# Plasma-derived candidate biomarkers for detection of gallbladder carcinoma

**DOI:** 10.1038/s41598-021-02923-7

**Published:** 2021-12-07

**Authors:** Ratna Priya, Vaishali Jain, Javed Akhtar, Geeta Chauhan, Puja Sakhuja, Surbhi Goyal, Anil Kumar Agarwal, Amit Javed, Ankit P. Jain, Ravindra Varma Polisetty, Ravi Sirdeshmukh, Sudeshna Kar, Poonam Gautam

**Affiliations:** 1 Laboratory of Molecular Oncology, ICMR-National Institute of Pathology, Safdarjung Hospital Campus, New Delhi, 110029 India; 2 Jamia Hamdard-Institute of Molecular Medicine, Jamia Hamdard, New Delhi, 110062 India; 3 Manipal Academy of Higher Education (MAHE), Manipal, 576104 India; 4 Department of Pathology, Govind Ballabh Pant Institute of Postgraduate Medical Education and Research (GIPMER), New Delhi, 110002 India; 5 Institute of Bioinformatics, International Tech Park, Bangalore, 560066 India; 6 Department of Biochemistry, Sri Venkateswara College, University of Delhi, New Delhi, 110021 India

**Keywords:** Gastrointestinal cancer, Tumour biomarkers, Biomarkers

## Abstract

Gallbladder carcinoma (GBC) is a major cancer of the gastrointestinal tract with poor prognosis. Reliable and affordable biomarker-based assays with high sensitivity and specificity for the detection of this cancer are a clinical need. With the aim of studying the potential of the plasma-derived extracellular vesicles (EVs), we carried out quantitative proteomic analysis of the EV proteins, using three types of controls and various stages of the disease, which led to the identification of 86 proteins with altered abundance. These include 29 proteins unique to early stage, 44 unique to the advanced stage and 13 proteins being common to both the stages. Many proteins are functionally relevant to the tumor condition or have been also known to be differentially expressed in GBC tissues. Several of them are also present in the plasma in free state. Clinical verification of three tumor-associated proteins with elevated levels in comparison to all the three control types—5′-nucleotidase isoform 2 (NT5E), aminopeptidase N (ANPEP) and neprilysin (MME) was carried out using individual plasma samples from early or advanced stage GBC. Sensitivity and specificity assessment based on receiver operating characteristic (ROC) analysis indicated a significant association of NT5E and ANPEP with advanced stage GBC and MME with early stage GBC. These and other proteins identified in the study may be potentially useful for developing new diagnostics for GBC.

## Introduction

Gallbladder carcinoma (GBC) is the fifth most common and aggressive malignancy of the gastrointestinal tract, with high prevalence and incidence rate in Latin America and Asian region, including northern and northeast India^1,2^. Gallstone disease (GSD) cases are at high risk, with about 60% GBC cases reported to have prior GSD^3^. GBC generally has poor survival outcome due to its anatomic position, absence of specific early stage symptoms and presentation at an advanced stage^4^. Inspite of standard treatment for GBC i.e. surgery followed by chemotherapy and/or radiotherapy, ~ 35% of the cases recur with a median time of 9.5 months^5^.

The tumor markers CEA and CA19-9 are presently in use for the detection of GBC^6–8^, however, there are continued efforts to identify novel circulatory biomarkers with improved sensitivity for detection of GBC. In an effort towards this, various groups have applied high throughput proteomics to study altered levels of proteins in serum/plasma and their applications in clinical diagnosis and prognosis of GBC. A study on altered level of proteins in serum from GBC patients led to the identification of 24 proteins, and suggested S100A10 and haptoglobin as potential molecular targets for early diagnosis for GBC^9^. Liu et al. analyzed serum levels of 49 inflammatory proteins using Luminex bead-based assay and reported significant association of eight proteins with overall survival of GBC patients^10^.

Extracellular vesicles (EVs) secreted by tumor cells carry tumor-associated proteins, mRNAs, miRNAs and could be accessed in various biological fluids such as blood plasma, urine and others^11,12^. They have been explored in several other cancers including cholangiocarcinoma^13^, pancreatic cancer^14^, colorectal cancer^15^ and ovarian cancer^16^. In the present study, we have analyzed plasma-derived EV proteins with altered levels in early and advanced stage GBC in comparison to the controls. The EV-derived proteins identified are suggestive of their tumor origin although some of them are also present in free state in plasma. Three proteins were verified in plasma samples directly, suggesting their potential for developing GBC diagnostics.

## Methodology

### Clinical sample collection, characterization and sample processing

Blood samples from adult patients diagnosed with GBC, GSD, xanthogranulomatous cholecystitis (XGC) and healthy individuals were collected from Govind Ballabh Pant Institute of Postgraduate Medical Education and Research (GIPMER), New Delhi, after approval from the Institutional Human Ethics Committee [Maulana Azad Medical College-Institutional Ethics Committee and ICMR-National Institute of Pathology-Institutional Ethics Committee, New Delhi]. All the methods were performed in accordance with the relevant guidelines and regulations. Cases include early stage GBC (stages I and II) and advanced stage GBC (stages III and IV). Tumor Staging was done on the basis of clinical data of patients, histopathological evaluation and imaging tools, as per American Joint Committee on Cancer (AJCC), 8th edition staging system^17^. GBC cases with ≥ 20 year age group and adenocarcinomas were included for the study. GBC cases with age < 20 years or having malignancy other than GBC or those who have already taken the treatment were excluded for the study. The tumor staging and histological grading for GBC cases is provided in the Supplementary Table S1. Controls include healthy individuals, GSD cases with no dysplasia and XGC cases. XGC is a benign, uncommon variant of chronic cholecystitis characterized by focal or diffuse destructive inflammation of the gall bladder^18^. The control group did not have any malignancy. Table 1 and Supplementary Table S1 includes the details of the clinico-pathological features of the samples used in the study.

**Table 1:** Clinical samples used for the study.

Subjects	Total number	Number of males	Number of females	Mean age (years)	Age range (years)
Total GBC cases	56	9	47	51.05	30–78
**Stages**					
GBC, Stage I	8	2	6	46.12	38–55
GBC, Stage II	5	0	5	50	34–65
GBC, Stage IIIA	13	0	13	51.76	30–66
GBC, Stage IIIB	4	1	3	52.25	45–62
GBC, Stage IVA	2	0	2	59	55–63
GBC, Stage IVB	24	6	18	51.66	38–78
Early stages (I and II)	13	2	11	47.61	34–65
Advanced stages (IIIA, IIIB, IVA and IVB)	43	7	36	52.09	30–78
**Histological grade**					
Well-differentiated (G1)	14	3	11	51.57	38–65
Moderately-differentiated (G2)	27	3	24	47.96	30–65
Poorly-differentiated (G3)	15	3	12	56.13	42–78
Total controls	57	15	42	44.61	22–71
GSD cases	23	6	17	44.27	22–70
Healthy group	25	4	21	42.52	25–59
XGC	9	5	4	50.77	26–71

*GBC* gallbladder carcinomas, *GSD* gallstone disease, *XGC* xanthogranulomatous cholecystitis.

Clinical parameters such as white cell count, liver enzymes (AST/ALT/ALP), bilirubin and co-morbidities (jaundice, pulmonary tuberculosis, asthma, diabetes melitus, hypertension, loss of appetite and loss of weight, thyroid disease) for the GBC patients and control groups as available (~ 70%) are provided in Supplementary Table S1. The co-morbidities were reported in both cases and controls among the subjects enrolled for discovery study (proteomics) and verification study (ELISA and IHC).

Peripheral blood (~ 5 ml) was collected from patients with early stage GBC (Stage I, n = 8, Stage II, n = 5), advanced stage GBC (stage III, n = 17 and stage IV, n = 26), GSD cases (n = 23), XGC cases (n = 9) before surgery and from healthy individuals (n = 25). The samples were processed within 30 min of collection for the separation of plasma. The samples were centrifuged at 2000 × *g* for 20 min at 4° C, clear plasma separated, aliquoted and stored at − 80 °C for further EV isolation, quantitative proteomic analysis and ELISA.

Formalin-fixed paraffin-embedded (FFPE) tissue samples from GBC cases [early and advanced stage] and controls [GSD and XGC cases] were drawn from GIPMER, New Delhi, India, after approval from the Institutional Human Ethics Committee and used for immunohistochemistry (IHC) analysis. The details of the in-house tissue microarray (TMA) preparation and samples used are described under “Immunohistochemistry analysis” section.

### EV isolation and characterization

#### EV isolation

Blood plasma was pooled for EV isolation and quantitative proteomic analysis experiments involving early stage GBC cases (Experiment I) and advanced stage GBC cases (Experiment II). For Experiment I, an equal volume of blood plasma was pooled from healthy individuals (n = 5) or GSD cases (n = 5) or XGC (n = 5), GBC stage I and II (n = 5) (age and gender matched) for EV isolation. For Experiment II, an equal volume of blood plasma was pooled from healthy individuals (n = 11) or GSD cases (n = 11) or GBC stage IIIA (n = 9) or GBC stage IVB (n = 11) (age and gender matched).

EVs were isolated using ultracentrifugation-based method as described earlier^19^ with minor modifications. Briefly, blood plasma was diluted 1:4 with 1× PBS and spun at 500 × *g* at 4 °C for 30 min to remove any cells. The supernatant was further centrifuged at 12,000 × *g* at 4 °C for 45 min to sediment any larger vesicles. Supernatant was then filtered through a 0.22 µm PVDF membrane filter (Millipore, Manchester, USA) to remove vesicles larger than 220 nm and enrich ‘exosomes’. EVs were pelleted by ultracentrifugation (Sorvall discovery M150 SE, Hitachi, UK) at 1,20,000 × *g* at 4 °C for 1.5 h. The pellet contained EVs and the supernatant was collected as ‘EV-depleted’ fraction. ‘EV pellet’ was washed in PBS by centrifugation at 1,20,000 × *g* at 4 °C for 1.5 h. A part of EVs were resuspended in 1× PBS for characterization and remaining was used for protein extraction.

#### Nanoparticle tracking system (NTA) analysis

EVs resuspended in 1× PBS were analyzed for size and concentration by NTA using a NanoSight LM20 system (Malvern, UK). Samples were introduced manually and the video images were recorded for 60 s using the NTA software (version 3.1) with camera level-16 and screen gain-10. Processing of images was performed with detection threshold 3 and screen gain 10. Each video was analyzed to obtain the mode vesicle size and the concentration. For all the samples, NTA acquisition settings were kept constant. Each experiment was carried out in duplicates. The NanoSight was calibrated with 20 nm, 60 nm and 120 nm latex beads.

#### Transmission electron microscopy (TEM)

EVs resuspended in 1× PBS was loaded on carbon-coated grids. The sample was washed with MQ water twice followed by negative staining performed using 2% phosphotungstic acid (PTA). Images of EVs were acquired using TEM (120 kV Hitachi TEM 7500, USA) at 1,04,000× magnification.

#### SDS-PAGE analysis

EV pellet was dissolved in modified RIPA buffer [25 mM Tris–Cl, pH 7.6 + 150 mM NaCl + 2% 3-{(3-cholamidopropyl) dimethylammonio]-1-propanesulfonate (CHAPS)} with 0.5% protease inhibitor cocktail (Sigma, USA)] followed by sonication (Biologics 3000MP, USA) with four bursts of 10 s each with 10 s of pause interval at 4 °C for protein extraction. Total EV protein was estimated by Bradford assay^20^. A total of 15 μg protein from EV-depleted fraction and EV fraction was loaded on SDS-PAGE. The gel was stained with Coomassie Brilliant Blue R250 to visualize the proteins. Image was acquired using imaging system (ChemidocMP, Bio-Rad, USA). Protein load from different groups of cases and controls was normalized based on total density and BSA as loading control.

### Quantitative proteomic analysis

#### iTRAQ labeling and SCX fractionation

Blood plasma-derived EV proteins from healthy individuals, XGC, GSD and early stage GBC (85 µg each group) were subjected to trypsin digestion and the peptides were labelled with iTRAQ reagents according to the manufacturer’s instructions (iTRAQ Reagents Multiplex kit; Applied Biosystems/MDS Sciex, CA, USA). EV protein digest from healthy individuals, GSD, XGC and early stage GBC was labeled with 114, 115, 116 and 117 tags respectively. All the four labelled peptide samples were pooled, vacuum-dried and subjected to strong cation exchange (SCX) chromatography as described previously^21^. A total of six SCX fractions were collected and then desalted using C18 cartridge (Pierce, Rockford, USA) as per the manufacturer’s instructions for further LC–MS/MS analysis.

Similarly, blood plasma-derived EV proteins from healthy individuals, GSD, GBC stage IIIA and GBC stage IVB (85 µg each group) were subjected to trypsin digestion and the peptides were labelled with iTRAQ reagents. 114, 115, 116 and 117 tags respectively. All the four labelled peptide samples were pooled, vacuum-dried and subjected to strong cation exchange (SCX) chromatography as described previously^21^. A total of eight SCX fractions were collected and then the fractions were desalted using C18 cartridge (Pierce, Rockford, USA) as per the manufacturer’s instructions for further LC–MS/MS analysis.

#### LC–MS/MS analysis

Nanoflow electrospray ionization tandem mass spectrometric analysis was carried out using QExactive plus (Thermo Scientific, Bremen, Germany) interfaced with Dinonex RS nanoLC 3000 nanoflow LC system. Peptides from each SCX fraction were enriched using a C18 trap column (75 μm × 2 cm) at a flow rate of 3 μl/min and fractionated on an analytical column (75 μm × 50 cm) at a flow rate of 300 nl/min using a linear gradient of 8–35% acetonitrile (ACN) over 85 min. Mass spectrometric analysis was performed in a data dependent manner using the Orbitrap mass analyzer at a mass resolution of 70,000 at m/z 200. For each MS cycle, 10 top most intense precursor ions were selected and subjected to MS/MS fragmentation and detected at a mass resolution of 35,000 at m/z 200. The fragmentation was carried out using higher-energy collision dissociation (HCD) mode. Normalized collision energy (CE) of 30% was used to obtain release of reporter ions from all peptides detected in the full scan. The ions selected for fragmentation were excluded for next 30 s. The automatic gain control for full FT MS and FT MS/MS was set to 3e^6^ ions and 1e^5^ ions respectively with a maximum time of accumulation of 50 ms for MS and 75 ms for MS/MS. The lock mass with 10 ppm error window option was enabled for accurate mass measurements^22^.

#### Identification and quantification of proteins

Protein identification, quantification and annotations of differentially abundant proteins were carried out as described earlier by Polisetty et al.^22^. The MS/MS data was analyzed using Proteome Discoverer (Thermo Fisher Scientific, version 1.4) with Mascot and Sequest HT search engine nodes using NCBI RefSeq database (release 81). Search parameters included trypsin as the enzyme with 1 missed cleavage allowed; precursor and fragment mass tolerance were set to 10 ppm and 0.1 Da, respectively; Methionine oxidation and deamidation of asparagines and glutamine amino acids was set as a dynamic modification while methylthio modification at cysteine and iTRAQ modification at N-terminus of the peptide and lysines were set as static modifications. The peptide and protein information were extracted using high peptide confidence and top one peptide rank filters. The FDR was calculated using percolator node in proteome discoverer 1.4. High confidence peptide identifications were obtained by setting a target FDR threshold of 1% at the peptide level. Relative quantitation of proteins was carried out based on the intensities of reporter ions released during MS/MS fragmentation of peptides. The average relative intensities of the two reporter ions for each of the unique peptide identifiers for a protein were used to determine relative quantity of a protein and percentage variability. Proteins with twofold-change or above in GBC were considered significant and used for further analysis^22^. P-value was calculated based on the intensity of PSMs. A volcano map was prepared by using log2 fold change and − log10 (p-value) as the co-ordinates and significant fold change ≥ 2.0 and p-value < 0.05 were considered to screen the proteins.

Mapping of proteins with altered levels in GBC was done for associated cellular components using the STRING (Search Tool for the Retrieval of Interacting Genes/Proteins) database^23^.

### Enzyme-linked immunosorbent assay (ELISA)

ELISA assays were carried out to measure the level of human 5′-nucleotidase isoform 2 (NT5E) and aminopeptidase N (ANPEP) directly in individual plasma samples after sonication to solubilize EVs. For verification, we used a total of 45 controls and 55 GBC cases including samples from the discovery set (proteomics study, 20 controls and 19 GBC cases) and an independent cohort (25 controls and 36 cases) (Supplementary Table S2A). ELISA quantitation kits (Abcam, USA) were used for the assays. The plasma level of human neprilysisn (MME) was measured in individual samples from 24 controls and 13 GBC cases including samples from the discovery set (proteomics study, 14 controls and 4 GBC cases) and an independent cohort (10 controls and 9 GBC cases) (Supplementary Table S2A) using ELISA quantitation kit (ThermoFisher Scientific, USA).

Concentrations of NT5E, ANPEP and MME are presented as scatter plot and statistical analysis was performed using GraphPad Prism 5^24^. Differences in protein levels between two independent groups was tested with Unpaired t-test (two-tailed) with confidence intervals of 95% and *p*-value less than 0.05 indicated statistical significance as described earlier by Akhtar et al*.*^25^. The receiver operating characteristic (ROC) analysis for NT5E and ANPEP for various groups of GBC [all GBC (early and advanced) vs all controls, early stage GBC (stages I and II) vs all controls; advanced stage GBC (stages III and IV) vs all controls] was performed leading to the estimates of area under the curve (AUC) with 95% confidence interval (CI) along with sensitivity and specificity. ROC analysis for MME for early stage GBC (stages I and II) vs all controls was performed leading to the estimates of area under the curve (AUC) with 95% confidence interval (CI) along with sensitivity and specificity^25^. The above analysis was performed for the samples from discovery cohort (D), independent cohort (IC) and combined cohort (D + IC). AUC value > 0.7 were considered significant.

### Immunohistochemistry analysis

IHC was performed on FFPE tissues using tissue microarray (TMA) and individual tissue sections from 23 controls and 47 GBC cases (Supplementary Table S2B) to analyze the expression of NT5E and MME protein. In-house TMAs were prepared as follows. An in-house TMA block was constructed using the FFPE blocks and included 6 controls (6 GSD cases) and 14 GBC cases (2 early stage and 12 advanced stage). Each TMA block consisted of tissue cores of 2 mm diameter and 4 µm sections were cut from the TMA block for carrying out IHC. Individual tissue sections (FFPE) of 33 GBC (11 early stage and 22 advanced stage) and 17 controls (10 GSD and 7 XGC cases) were also for IHC analysis. We used the serial sections from TMA and individual tissue blocks for IHC analysis of NT5E and MME. IHC analysis was performed as described earlier by Akhtar et al*.*^25^. In brief, after deparaffinization and rehydration of FFPE tissue sections, antigen retrieval was performed by immersing the slide in antigen retrieval buffer (20 mM Tris buffer, pH 9.0) at 90 °C for 20 min. Endogenous peroxidases were blocked with 0.03% hydrogen peroxide, and nonspecific binding was blocked with protein blocking reagent. Sections were then incubated for 1 h at RT with primary antibody against NT5E (dilution 1:400, Cat. No. ab91086, Abcam, USA) and MME (dilution 1:50, Cat. No. MA5-14050, Thermo, USA) followed by incubation with PolyExcel PolyHRP for 40 min at RT. Tissue sections were then incubated with Stunn DAB working solution for 5 min at RT (PathnSitu Biotechnologies, USA). Sections were counter stained with Mayer’s hematoxylin, dehydrated and images were taken under the microscope. The distribution of staining and staining intensity across the section was observed under the microscope. Scoring criteria were based on both staining intensities and distributions.

For NT5E, normal and non-neoplastic glands did not show apical expression; > 10% cytoplasmic/membranous/apical positivity, 2–3 + intensity were considered as ‘Positive’, while 1 + positivity was considered as ‘Negative’. For MME, normal and non-neoplastic glands showed apical expression; > 10% cytoplasmic/membranous positivity and 2–3 + intensity were considered as ‘Positive’, while 1 + positivity and apical expression were considered as ‘Negative’. IHC data analysis was done by two independent pathologists.

The statistical analysis (Fisher’s exact test) was performed using GraphPad Prism 5^24^ to study the correlation of NT5E and MME expression among cases and controls (early stage GBC vs controls; advanced stage vs controls; all GBC vs controls). The *p*-value less than 0.05 indicated statistical significance.

### Ethics approval and consent to participate

Clinical samples from participants visiting GIPMER, Delhi, were collected for the study after approval from the Institutional Human Ethics Committee [MAMC-IEC (No: F.1/IEC/MAMC (51/5/2015/No. 12) and NIP-IEC/21-12/04)]. All the participants provided informed consent to participate in the study and written informed consent was obtained.

## Results

**Figure 1:**
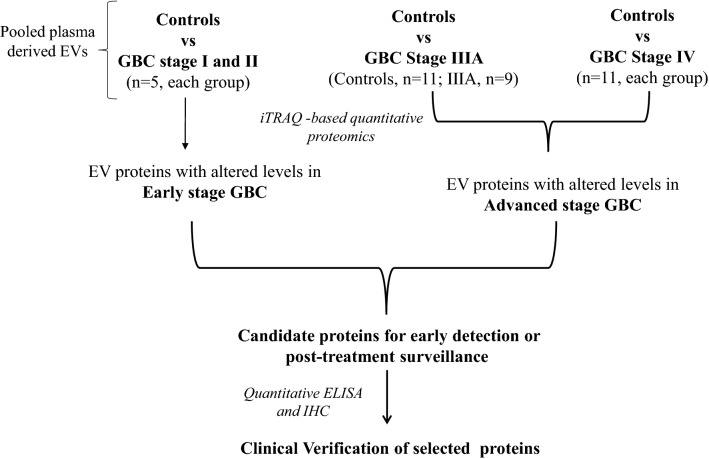
Overall workflow of the study. *GBC* gallbladder carcinoma, *EV* extracellular vesicles, *iTRAQ* isobaric tags for relative and absolute quantitation, *ELISA* enzyme-linked immunosorbent assay.

In the present study, using plasma-derived EVs, we investigated differentially abundant proteins in early and advanced stages of GBC. Three of the tumor-associated proteins, which are also present in the plasma in free state, were verified in individual plasma samples by quantitative ELISA. The overall workflow of the study is shown in Fig. 1.

### EV characterization

The size and particle distribution plots of EVs by NTA analysis showed an average size (mode) of 161 nm, suggesting enrichment of ‘exosomes’ in EV preparation (see Supplementary Fig. S1A). TEM analysis of blood plasma-derived EVs showed size ranging from 30 to 100 nm. The Supplementary Fig. S1B shows the representative transmission electron micrograph of plasma-derived EVs. For EV isolation, the plasma samples were passed through 0.22 µm cut off filters to obtain EVs enriched with ‘exosomes’. We have detected CD9, an exosomal marker, in the proteomics data with high confidence, however, the other exosome markers could not be detected due to technical limitations. Protein profile of EV and EV-depleted fraction from pooled plasma of cases (early and advanced stage GBC) and controls (healthy individuals, GSD cases, XGC cases) using SDS-PAGE analysis showed insignificant levels of serum albumin contamination (Supplementary Figs. S2, S3, S7).

Quantification of total EV protein obtained from equal volume of pooled plasma from different groups showed a significant increase in GBC cases in comparison to controls (healthy individuals or GSD cases or XGC cases). We also found a significant increase in EV proteins in advanced stages of GBC in comparison to early stage GBC (Supplementary Fig. S1C).

### Differential EV proteome in GBC

**Figure 2:**
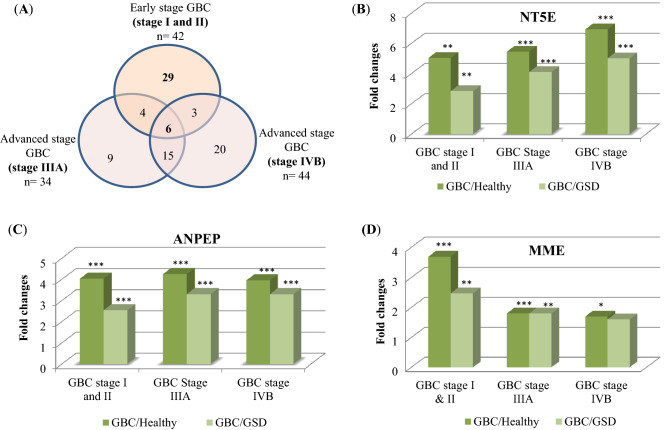
(**A**) Venn diagram showing EV proteins with altered levels in early and advanced stages of GBC. A total of 42, 34, 44 proteins were found to have altered levels in early stage GBC, GBC stage IIIA and stage IVB respectively in comparison to controls (healthy individuals/GSD/XGC). A total of 13 proteins are common among early and advanced stage GBC which may be associated with progression of the disease and a total of 20 proteins are with altered levels only in GBC Stage IVB which may be associated with metastasis. Proteins showing altered levels with ≥ twofold change were considered for the analysis. Altered levels of (**B**) NT5E (**C**) ANPEP and (**D**) MME in early and advanced stages of GBC as observed in quantitative proteomics data. The levels of NT5E and ANPEP were significantly altered (with ≥ twofold change and p value ≤ 0.05) in both early and advanced stage GBC, while MME were significantly altered in early stage GBC. The p values ≤ 0.05, ≤ 0.01, ≤ 0.001 are marked with ‘*’, ‘**’ and ‘***’ respectively. *GSD* gallstone disease, *XGC* xanthogranulomatous cholecystitis.

We performed two independent 4-plex iTRAQ experiments for the identification of differentially abundant proteins in early and advanced stage GBC. In experiment I, quantitative proteomic analysis of EVs derived from pooled plasma of early stage GBC patients vs healthy individuals, GSD, XGC patients used as controls, led to the identification of a total of 236 proteins, 42 of which were with altered levels (≥ twofold change) in GBC (Fig. 2A, Supplementary Table S3). Majority of these proteins (> 97%) showed significant p-value (< 0.05). Volcano plot analysis showing differentially expressed proteins in early stage GBC in comparison to each control type (healthy or GSD or XGC) is shown in Supplementary Fig. S4. The workflow for the quantitative proteomic analysis is shown in Supplementary Fig. S5 and the list of 42 proteins with corresponding peptides is shown in Supplementary Table S4. In experiment II, quantitative proteomic analysis of EVs derived from pooled plasma of GBC stage IIIA and stage IVB vs healthy individuals, GSD as controls led to the identification of a total of 426 proteins, 57 of which were with altered levels (≥ twofold change) (Fig. 2A, Supplementary Table S3). Majority of these proteins (> 98%) showed significant p-value (< 0.05). Volcano plot analysis showing differentially expressed proteins in advanced stage GBC in comparison to each control type (healthy or GSD) is shown in Supplementary Fig. S4. The workflow for the quantitative proteomic analysis is shown in Supplementary Fig. S6 and the list of 57 proteins with corresponding peptides is shown in Supplementary Table S5. A representative list of proteins with altered levels in early and/or advanced stage GBC is shown in Table 2.

**Table 2:** Representative list of plasma-derived EV proteins with altered levels in early and/or advanced stage GBC.

Gene symbol	Protein name	Altered levels in GBC (based on EV proteomics data)	Protein localization	Cancer association		Reported in GBC	Reported in EVs in other cancers
				HPA data	Literature survey		
ACTG1	Actin, cytoplasmic 2	Early stage and Advanced stage IIIA and IVB	Intracellular	–	Yes	No	Yes
ANPEP	Aminopeptidase N	Early stage and Advanced stage IIIA and IVB	Membrane	–	Yes	No	Yes
CRP	C-reactive protein	Early stage and Advanced stage IIIA and IVB	Intracellular, secreted	Yes	Yes	Yes-Serum, bile	No
FLNA	PREDICTED: filamin-A isoform X5	Early stage and Advanced stage IIIA and IVB	Intracellular	–	Yes	No	Yes
ITGA2B	Integrin alpha-iib	Early stage and Advanced stage IIIA and IVB	Intracellular, membrane	Yes	Yes	No	No
NT5E	5′-Nucleotidase isoform 2	Early stage and Advanced stage IIIA and IVB	Intracellular, membrane	–	Yes	Yes-Tissue	Yes
ITGA6	PREDICTED: integrin alpha-6 isoform X6	Early stage	Intracellular, membrane	–	Yes	Yes-Tissue	No
ITGB1	Integrin beta-1 isoform 1A	Early stage	Intracellular, membrane	Yes	Yes	No	Yes
MME	Neprilysin	Early stage	Intracellular, membrane	Yes	Yes	No	Yes
PKM	Pyruvate kinase PKM isoform d	Early stage	Intracellular	Yes	Yes	Yes-Tissue	Yes
SAA1	Serum amyloid A-1 protein	Early stage	secreted	–	Yes	No	No
SAA4	Serum amyloid A-4 protein	Early stage	secreted	–	Yes	No	No
CFL1	Cofilin-1	Advanced GBC stage IIIA and IVB	Intracellular	–	Yes	Yes-Tissue	Yes
ALPL	Alkaline phosphatase, tissue-nonspecific isozyme isoform 2	Advanced GBC stage IIIA and IVB	Intracellular, membrane	Yes	Yes	Yes-Serum	No
RAP1B	Ras-related protein Rap-1b isoform 4	Advanced GBC stage IIIA and IVB	Intracellular	–	Yes	No	Yes
DPP4	PREDICTED: dipeptidyl peptidase 4 isoform X1	Advanced GBC stage IIIA and IVB	Intracellular, membrane, secreted	–	Yes	No	Yes
TFG	PREDICTED: protein TFG isoform X2	Advanced GBC stage IIIA and IVB	Intracellular	Yes	Yes	No	No
PFN1	Profilin-1	Advanced GBC stage IIIA and IVB	Intracellular	–	Yes	No	Yes

The table shows protein localization (uniprot database), cancer association (HPA database and literature search), altered level in GBC tissue/blood plasma/serumand plasma-derived EVs in other cancers (based on literature). The non- redundant list of 86 plasma-derived EV proteins with altered levels (≥ 2.0 fold change) in early and/or advanced stage GBC are shown in Supplementary Table S2.

Overall, the study resulted in the identification of a total of ~ 500 proteins in EVs and 86 non-redundant proteins with altered levels in GBC (Fig. 2A, Supplementary Table S3). Gene Ontology annotations of these proteins using STRING database showed extracellular region, vesicle, secretory granule, cytoplasmic vesicle lumen, cytoplasmic vesicles as top ‘cellular components’ (Supplementary Table S6). Out of 86 proteins, 13 proteins were common to both early and advanced stage GBC. Some of the tumor-associated proteins with differential levels in comparison to all the control types include 5′-nucleotidase isoform 2 (NT5E), aminopeptidase N (ANPEP). A total of 29 proteins were detected only in early stage GBC including neprilysin (MME), serum amyloid A-1 protein (SAA1) that showed significantly increased levels in compared to all control types. A total of 44 proteins were detected only in advanced stage GBC cases. These include some of the tumor associated proteins with differential levels in both stage IIIA and IVB such as alkaline phosphatase, tissue-nonspecific isozyme isoform 1 preproprotein (ALPL), dipeptidyl peptidase 4 isoform X1 (DPP4), protein TFG isoform X2 (TFG) (Table 2, Supplementary Table S3).

### Clinical verification by ELISA

**Figure 3:**
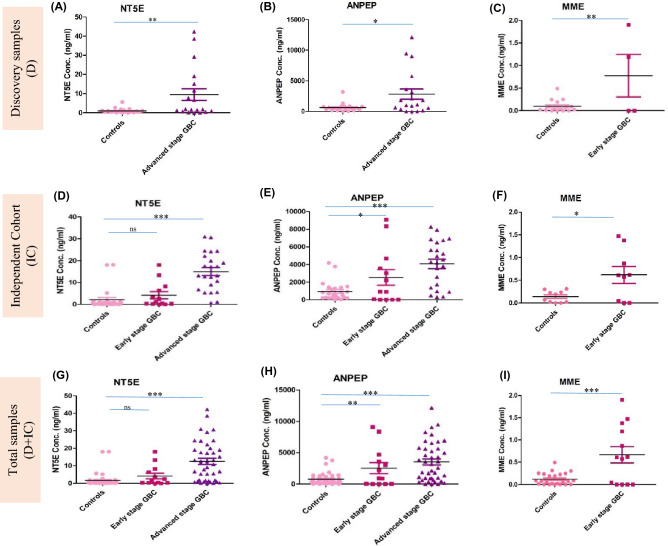
Protein concentration of NT5E, ANPEP and MME in controls and GBC cases using quantitative ELISA. Scatter plot showing NT5E, ANPEP and MME concentration in sonicated plasma samples from discovery cohort (**A**, **D**, **G**), an independent cohort (**B**, **E**, **H**) and combined cohort (discovery + Independent cohort) (**C**, **F**, **I**). Controls include healthy individuals, GSD, XGC cases. A significant increase in the levels of MME was observed in early stage GBC cases, NT5E in advanced stage GBC cases and ANPEP in both early and advanced stage GBC.

**Figure 4:**
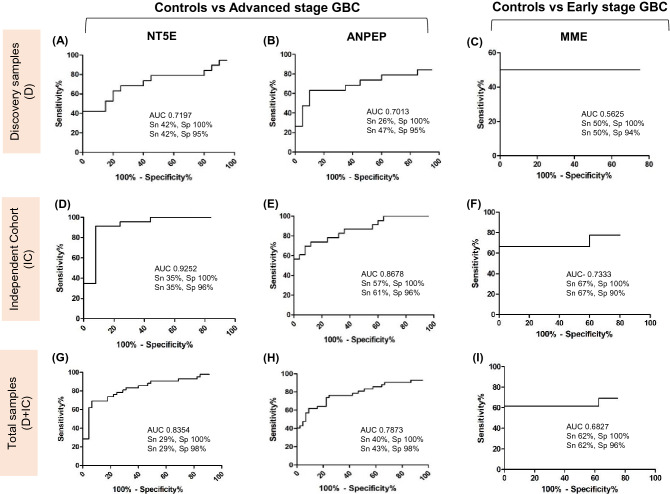
ROC curve representing sensitivity and specificity for NT5E, ANPEP and MME. ROC curve showing AUC, sensitivity and specificity for NT5E, ANPEP and MME in plasma samples from discovery cohort (**A**, **D**, **G**), an independent cohort (**B**, **E**, **H**) and combined cohort (discovery + Independent cohort) (**C**, **F**, **I**). AUC for independent cohort was increased for all the three proteins in comparison to the discovery cohort.

Three proteins, NT5E, ANPEP and MME, were selected for clinical verification based on differential abundance in GBC compared to all control types (healthy, GSD and XGC cases), their association with tumor state in other cancers and functional relevance to tumor condition. The fold changes for each protein, as observed in proteomic analysis, are shown in Fig. 2B–D and Supplementary Tables S4 and S5. We performed clinical verifications for NT5E, ANPEP and MME in individual samples from discovery cohort (proteomics) and an independent cohort using sonicated plasma and the results are represented as scatter plot in Fig. 3. The mean value of NT5E and ANPEP for early stage GBC (stage I and II), GBC stage III and IV and controls i.e. healthy individuals, GSD, XGC cases is shown in Supplementary Table S7. We observed significantly increased levels of NT5E in the advanced stage GBC cases in comparison to all controls (p value ≤ 0.0001), whereas there was no significant increase in the early stage GBC (Fig. 3A,D,G). An increased level of ANPEP was observed in both early and advanced stage GBC cases (p value = 0.0017 and < 0.0001 respectively) in comparison to all controls (Fig. 3B,E,H). Receiver operating characteristic (ROC) curve analysis for the discovery cohort, independent cohort and combined cohort for NT5E, ANPEP (for advanced stage GBC vs controls) is shown in Fig. 4 and Supplementary Table S8.

The mean value of MME for early stage GBC (stage I and II) healthy individuals and GSD is shown in Supplementary Table S7. Analysis of MME levels in early stage GBC in comparison to all controls showed a significant increase in early stage GBC (p value = 0.0004) (Fig. 3C,F,I). Receiver operating characteristic (ROC) curve analysis for discovery cohort, independent cohort and combined cohort for MME (for early stage GBC vs controls) is shown in Fig. 4 and Supplementary Table S8.

### IHC analysis

**Figure 5:**
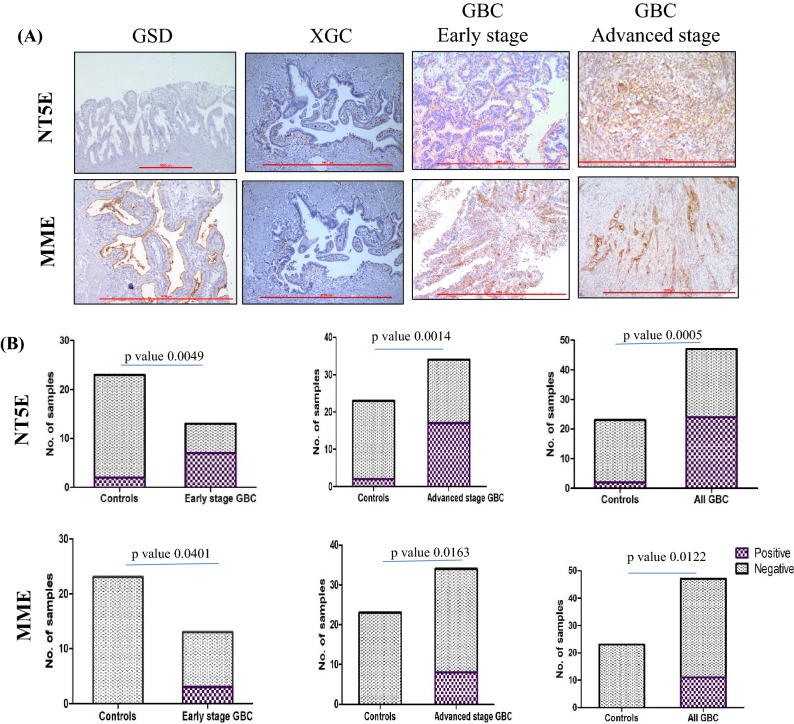
IHC analysis to study the expression of NT5E and MME in controls and GBC cases. (**A**) Representative IHC images showing the expression of NT5E and MME in controls and GBC cases. IHC was performed on formalin-fixed paraffin-embedded (FFPE) tissue microarray (TMA) and individual tissue sections. An in-house TMA block was constructed using the FFPE blocks and included 6 controls (6 GSD cases) and 14 GBC cases (2 early stage and 12 advanced stage). Each TMA block consisted of tissue cores of 2 mm diameter and 4 µm sections were cut from the TMA block for carrying out IHC. Individual tissue sections (FFPE) of 33 GBC (11 early stage and 22 advanced stage) and 17 controls (10 GSD and 7 XGC cases) were also for IHC analysis. IHC results showed positive expression for NT5E in 53.84% of early and 50% of advanced stage GBC cases while MME showed positive expression in 23.07% of early and 23.52% of advanced stage GBC cases. (**B**) The statistical analysis showed a significant difference between cases and controls for NT5E and MME for both early stage and advanced stage GBC. The scale bar is shown as red line. IHC scoring is shown in “Methodology” section “Immunohistochemistry analysis”.

We performed IHC analysis to study the expression of NT5E and MME in 23 controls (16 GSD cases and 7 XGC cases) and 47 GBC cases (13 early stage and 34 advanced stage GBC cases). We found ‘Positive’ expression levels in 51.06% of all GBC cases (53.84% in early stage and 50% in advanced stage) and 23.40% of all GBC cases (23.07% in early stage and 23.52% in advanced stage) for NT5E and MME respectively. The statistical analysis between cases and controls showed a significant difference for NT5E and MME for early stage GBC vs all controls and advanced stage GBC vs all controls. The controls (≥ 95%) showed ‘Negative’ expression levels (Fig. 5). We performed IHC analysis for ANPEP, however, the results were not clear due to technical reasons.

## Discussion

The present study aimed at identification of circulatory markers for the detection of GBC. In the first discovery stage, we pooled the plasma from each group of clinical samples and performed EV isolation using ultracentrifugation-based method, the specimen groups being controls (healthy individuals, GSD cases and XGC cases) and cases from early stage (stage I and II) and advanced stage GBC (stage IIIA and IVB). The proteins from the plasma-derived EVs were analyzed for differential abundance using iTRAQ-based high resolution mass spectrometry. The second part involved the verification of selected (representative) differentially abundant proteins by ELISA using individual plasma specimens. This would therefore include EV-derived proteins as well as the protein present in free state, if any.

The proteomic analysis of total EV proteins from pooled plasma of cases vs controls led to the identification of 86 proteins with altered levels in GBC (42 in early stage, 57 in advanced stage and 13 being common between the stages). Many of these proteins are reported to have significantly altered levels in blood plasma-derived EVs in other cancers. Out of 86 proteins, majority of them are reported in several cancers, implying their tumor-relevance (Supplementary Table S3).

Global analysis of early and advanced stage GBC may identify proteins associated with the malignancy and may be useful for differentiating GBC from other GB diseases. We found 13 proteins common in both the stages that includes C-reactive protein (CRP), 5′-nucleotidase isoform 2 (NT5E or CD73), aminopeptidase N (ANPEP). Elevated CRP levels have earlier been reported in GBC^26^. CRP is a well-known inflammatory marker. We analyzed the co-morbidities such as jaundice, pulmonary tuberculosis, hypertension, diabetes etc. reported in controls and cases used for the discovery study (proteomics). For the Discovery study, the plasma was pooled for the different groups. We found that the co-morbidities were reported in both controls and cases, suggesting that it contributes insignificant towards increased levels of GGT and CRP (Supplementary Table S1).

NT5E, an ecto-nucleotidase, is a component of purinergic signaling and plays an important role in tumor cell escape from immune system. It is involved in the catabolism of extracellular AMP to adenosine, which activates specific G-protein coupled receptor (GPCR) leading to an increased intracellular cAMP level, resulting in tumor cell metastasis and angiogenesis. Cancer exosomes are reported to express NT5E (or CD73) and CD39, leading to extracellular adenosine in tumor microenvironment and suppress T Cells^27^. NT5E is reported to be overexpressed in tumor tissue samples from various cancers including GBC. It has been reported to promote tumor progression and survival of GBC patients by regulating epithelial-mesenchymal transition (EMT) induced by transforming growth factor 1 (TGF-1)^28^. ANPEP or CD13, is a cell surface ectopeptidase which plays an important role in degradation of proteins and peptides with an N-terminal neutral amino acid and shown to be involved in degradation of extracellular matrix, cell invasion, migration, and angiogenesis. It is also reported in exosomes derived from microglial cells^29^. ANPEP is also found to be overexpressed in tumor tissue of several cancers contributing to tumor progression, proliferation, tumor invasion and angiogenesis. Elevated levels of circulating ANPEP is reported to be valuable in detection of breast, pancreatic and thyroid cancer, and linked with poor prognosis in colorectal cancer and non-small cell lung cancer (NSCLC)^30^.

Delayed detection of cancer makes treatment difficult because of progressive advancement of the disease stage and metastasis. Five-year survival rate for early stage is higher (70–90% in Stage I and 45–60% in Stage II, when treated with extended cholecystectomy) in comparison to advanced stage (≤ 20%)^4^. Therefore, early detection of GBC is critical to reduce morbidity and mortality. In the present study, a total of 29 proteins were with altered levels only in early stage disease, and may be useful for early detection of GBC. Some of the proteins with altered levels in GBC in comparison to all the three control types include MME and SAA1. We discuss these proteins with reference to their relevance to cancer. Neprilysin [Membrane metallo-endopeptidase (MME) or common acute lymphoblastic leukemia antigen (CALLA) or CD10], a predominantly membrane-bound zinc-dependent metalloproteinase, has been associated with cardiovascular disease and cancer. It is responsible for the breakdown of multiple endogenous vasoactive peptides including bradykinin, natriuretic peptides, and adrenomedullin. MME overexpression is reported in hematopoietic malignancies and solid tumors such as colorectal, hepatocellular, lung, cervix or breast cancer and indicate poor prognosis^31^, however, the expression levels of MME is not yet explored in GBC. SAA1 is an acute phase protein secreted by liver and has been reported to be elevated in serum of GBC patients^26^.

Three of these proteins, NT5E, ANPEP and MME observed with significantly high fold changes in GBC in comparison to all the three control types were selected for clinical verifications. NT5E, ANPEP and MME were initially verified in EVs from pooled plasma using ELISA. NT5E has signal sequence while the other two were found to have extracellular localization (as per GO classification) suggesting their presence in plasma in free state. We also checked for their presence in the pooled plasma using ELISA and observed them to be present in free state as well with significantly increased levels in the tumor plasma specimens (data not shown).

Thus, clinical verification of NT5E and ANPEP was performed in early and advanced stage GBC in comparison to all control types. NT5E level was significantly increased in advanced stage while ANPEP level was significantly increased in both early and advanced stage GBC cases (Fig. 3D,E). ROC curve analysis for NT5E, ANPEP and MME for discovery and independent cohort showed that AUC was higher for the independent cohort in comparison to the discovery cohort (Fig. 4). Here, we discuss the sensitivity and specificity for the three proteins using the combined cohort (Discovery + Independent cohort). For NT5E, we observed 28.57% sensitivity with a specificity of 100% or 61.90% sensitivity with a specificity of 95.56% whereas ANPEP was found to have 40.48% sensitivity with a specificity of 100% or 43% sensitivity with a specificity 98% (Fig. 4). Earlier, Wang et al. used 71 advanced stage GBC cases and 78 subjects each from healthy controls, benign GB and reported that serum CEA, CA19-9, CA242, CA125 had a sensitivity of 11.2%, 60.6%, 64.8% and 46.4% respectively with specificity of > 96% in GBC cases^7^. CA19-9, CA242, CA125 were also shown to be with higher levels in recurrent GBC in comparison to non-recurrent group. The combination of previously reported markers, CA19-9, CA242, CA125 alongwith NT5E and ANPEP, identified in the present study, needs to be explored further and may represent a high confidence panel of markers with improved sensitivity for the detection of advanced stage GBC.

We performed clinical verification of MME, one of the tumor-associated proteins with significantly high fold changes in proteomics data, in individual plasma samples from controls and early stage GBC cases and found significantly increased levels in early stage GBC cases (Fig. 3F). MME showed 61.54% sensitivity with 100% specificity (Fig. 4). Earlier, Wang et al. used 07 subjects of early stage GBC cases and reported that serum CEA, CA19-9, CA242, CA125 had a sensitivity of 14.2%, 42.8%, 57.1% and 28.5% respectively with specificity of > 96% in early stage GBC cases^7^. We believe that MME alongwith CA19-9 and CA242 as a panel might improve the sensitivity for detection of early stage GBC cases.

Although we evaluated only three proteins for their potential as candidate biomarkers for detection of GBC, particularly at early stage, other proteins revealed in the proteomic analysis also provide an additional portfolio of molecules to be explored so as to develop a reliable panel for GBC diagnostics. The verified proteins are not GBC-specific and have been reported in other cancers, however, a reliable molecular test may add value to the case evaluation alongwith radiological imaging (CE-US, PET scan). The two would give complementary information for diagnosis and determining tumor localization. The validation of proteins verified in the present study needs to be done in an independent cohort of samples to explore their potential for clinical applications.

## Conclusions

The present study identified differentially abundant EV proteome in different stages of GBC. Of the three clinically verified tumor-associated proteins, NT5E and ANPEP showed the potential for detection of advanced stage GBC and MME for early stage GBC. These and other proteins identified in the study needs to be further investigated in large cohort of samples to establish their potential as markers for the detection of GBC.

## Supplementary Information

Supplementary Information.

## Data Availability

All data generated or analysed during this study are included in this published article and its supplementary information files.

## Abbreviations

GBC: Gallbladder carcinoma
EVs: Extracellular vesicles
GSD: Gallstone disease
XGC: Xanthogranulomatous cholecystitis
iTRAQ: Isobaric tags for relative and absolute quantitation
ELISA: Enzyme-linked immunosorbent assay
MME: Neprilysin
NT5E: 5′-Nucleotidase isoform 2
ANPEP: Aminopeptidase N
ROC: Receiver operating characteristic curve
AUC: Area under the ROC curve
NTA: Nanoparticle tracking analysis
SCX: Strong cation exchange
LC–MS: Liquid chromatography–mass spectrometry
FDR: False discovery rate
PSMs: Peptide spectral matches
STRING: Search tool for the retrieval of interacting genes/proteins

## References

[1] Mahdavifar N, Pakzad R, Ghoncheh M, Gandomani HS, Salehiniya H (2017). Epidemiology, incidence, and mortality of gallbladder cancer and its relation with development in the world. Ann. Trop. Med. Public Health..

[2] Malhotra RK, Manoharan N, Shukla NK, Rath GK (2017). Gallbladder cancer incidence in Delhi urban: A 25-year trend analysis. Indian J. Cancer..

[3] Randi G, Franceschi S, La Vecchi C (2006). Gallbladder cancer worldwide: Geographical distribution and risk factors. Int. J. Cancer..

[4] Shah SH, Gupta N, Gupta G, Mehta A, Singh S (2017). Lymph node micrometastasis in gallbladder cancer. Indian J. Gastroenterol..

[5] Margonis GA (2016). Rates and patterns of recurrence after curative intent resection for gallbladder cancer: A multi-institution analysis from the US Extra-hepatic Biliary Malignancy Consortium. HPB (Oxford)..

[6] Guidelines for patients Gallbladder and Bile duct cancers 2020 in National Comprehensive Cancer Network (NCCN). https://www.nccn.org/patients/guidelines/content/PDF/gallandbile-hppatient.pdf. Accessed 5th March 2020.

[7] Wang YF (2014). Combined detection tumor markers for diagnosis and prognosis of gallbladder cancer. World J. Gastroenterol..

[8] Shukla VK, Sharma D, Dixit VK (2006). Diagnostic value of serum CA242, CA 19–9, CA 15–3 and CA 125 in patients with carcinoma of the gallbladder. Trop. Gastroenterol..

[9] Tan Y (2011). Proteomic-based analysis for identification of potential serum biomarkers in gallbladder cancer. Oncol. Rep..

[10] Liu Z (2018). Circulating levels of inflammatory proteins and survival in patients with gallbladder cancer. Sci. Rep..

[11] VanNiel G, D'Angelo G, Raposo G (2018). Shedding light on the cell biology of extracellular vesicles. Nat. Rev. Mol. Cell Biol..

[12] Martins VR, Dias MS, Hainaut P (2013). Tumor-cell-derived microvesicles as carriers of molecular information in cancer. Curr. Opin. Oncol..

[13] Arbelaiz A (2017). Serum extracellular vesicles contain protein biomarkers for primary sclerosing cholangitis and cholangiocarcinoma. Hepatology.

[14] An M (2017). Quantitative proteomic analysis of serum exosomes from patients with locally advanced pancreatic cancer undergoing chemoradiotherapy. J. Proteome Res..

[15] Zhong ME (2019). Serum extracellular vesicles contain SPARC and LRG1 as biomarkers of colon cancer and differ by tumour primary location. EBioMedicine.

[16] Zhang W, Ou X, Wu X (2019). Proteomics profiling of plasma exosomes in epithelial ovarian cancer: A potential role in the coagulation cascade, diagnosis and prognosis. Int. J. Oncol..

[17] Amin MB (2017). The eighth edition AJCC cancer staging manual: Continuing to build a bridge from a population-based to a more "personalized" approach to cancer staging. CA Cancer J. Clin..

[18] Agarwal AK (2013). Mass-forming xanthogranulomatous cholecystitis masquerading as gallbladder cancer. J. Gastrointest. Surg..

[19] Szajnik, M. *et al*. Exosomes in plasma of patients with ovarian carcinoma: Potential biomarkers of tumor progression and response to therapy. *GynecolObstet (Sunnyvale)*. (Suppl 4), 3 (2013). 10.4172/2161-0932.S4-003 PMC3899646 24466501

[20] Bradford MM (1976). A rapid and sensitive method for the quantitation of microgram quantities of protein utilizing the principle of protein-dye binding. Anal. Biochem..

[21] Gautam P (2012). Proteins with altered levels in plasma from glioblastoma patients as revealed by iTRAQ-based quantitative proteomic analysis. PLoS ONE.

[22] Polisetty R (2016). Microsomal membrane proteome of low grade diffuse astrocytomas: Differentially expressed proteins and candidate surveillance biomarkers. Sci. Rep..

[23] Szklarczyk D (2015). STRING v10: Protein–protein interaction networks, integrated over the tree of life. Nucleic Acids Res..

[24] Unpaired t-test and ROC anlaysis were done using GraphPad Prism software version 5.0. www.graphpad.com. Accessed January 2020.

[25] Akhtar J (2020). Immunoproteomics approach revealed elevated autoantibody levels against ANXA1 in early stage gallbladder carcinoma. BMC Cancer.

[26] Koshiol J (2016). Association of inflammatory and other immune markers with gallbladder cancer: Results from two independent case-control studies. Cytokine.

[27] Clayton A, Al-Taei S, Webber J, Mason MD, Tabi Z (2011). Cancer exosomes express CD39 and CD73, which suppress T cells through adenosine production. J. Immunol..

[28] Xiong L, Wen Y, Miao X, Yang Z (2014). NT5E and FcGBP as key regulators of TGF-1-induced epithelial-mesenchymal transition (EMT) are associated with tumor progression and survival of patients with gallbladder cancer. Cell Tissue Res..

[29] Potolicchio I (2005). Proteomic analysis of microglia-derived exosomes: Metabolic role of the aminopeptidase CD13 in neuropeptide catabolism. J. Immunol..

[30] Wickström M, Larsson R, Nygren P, Gullbo J (2011). Aminopeptidase N (CD13) as a target for cancer chemotherapy. Cancer Sci..

[31] Pavo N (2019). The circulating form of neprilysin is not a general biomarker for overall survival in treatment-naïve cancer patients. Sci. Rep..

