# Supplementary material for: Plasma-derived candidate biomarkers for detection of gallbladder carcinoma
Source: Sci Rep. 2021 Dec 7;11:23554. doi: 10.1038/s41598-021-02923-7 (PMC8651660; doi:10.1038/s41598-021-02923-7)
Supplement: Supplementary file 1 — Supplementary Information. [file 41598_2021_2923_MOESM1_ESM.pdf]

Plasma-derived Candidate Biomarkers for Detection of Gallbladder Carcinoma

Authors and Affiliations:

[1,2]Ratna Priya, [1,3]Vaishali Jain, [1,2]Javed Akhtar, [4]Geeta Chauhan, [4#]Puja Sakhuja,[4]Surbhi Goyal,[4]Anil Kumar Agarwal, [4]Amit Javed, [5]Ankit P. Jain, [6]Ravindra Varma Polisetty, [3,5]Ravi Sirdeshmukh, [2]Sudeshna Kar, [1#]Poonam Gautam

[1]Laboratory of Molecular Oncology, ICMR- National Institute of Pathology, New Delhi-110029, India;

[2]Jamia Hamdard- Institute of Molecular Medicine, Jamia Hamdard, New Delhi-110062, India;

[3] Manipal Academy of Higher Education (MAHE), Manipal-576104, India;

[4]Govind Ballabh Pant Institute of Postgraduate Medical Education and Research (GIPMER), New Delhi-110002, India;

[5]Institute of Bioinformatics, International Tech Park, Bangalore-560066, India;

[6]Department of Biochemistry, Sri Venkateswara College, University of Delhi, New Delhi-110021, India

#Correspondence:

Dr. Poonam Gautam

ICMR-National Institute of Pathology,

Safdarjung Hospital Campus, New Delhi-110029, India

Phone: 0091-8800845323 ; E-mail: gautam.poonam@gmail.com; poonamgautam.nip@gov.in

AND

Dr. Puja Sakhuja

Department of Pathology, Govind Ballabh Pant Institute of Postgraduate Medical Education and Research (GIPMER), New Delhi- 110002, India

Phone: 0091-9718599073; E-mail: pujasak@gmail.com

SUPPLEMENTARY FILE LEGENDS

Supplementary Fig. S1:

Characterization of blood plasma-derived EVs (A) Size and particle distribution plots of EVs from human blood plasma-derived EVs using nanoparticle tracking system showed peaks at 161 nm suggesting enrichment of exosomes in EV fraction. (B) Transmission electron micrographs of plasma derived EVs. EVs from pooled plasma of healthy individuals isolated by ultracentrifugation method were resuspended in PBS and loaded on 2% collodion coated grids. Negative staining was performed using 2% phosphotungstic acid (PTA). Images of EVs (30-100 nm) were acquired using 120 KV Hitachi TEM 7500 at 1,04,000× magnification, scale bar- 100 nm. (C) Bar diagram showing EV protein amount in controls (healthy individuals, XGC, GSD) and cases (GBC stage I and II, stage IIIA, stage IVB). An increase in EV proteins was observed in both early and advanced stages of GBC cases in comparison to controls. * *p value* ≤ 0.05** *p value* ≤ 0.01.

Supplementary Fig. S2:

SDS-PAGE analysis showing protein profiling of EV fraction of pooled plasma from healthy individuals or GSD or XGC or early stage GBC (stage I and II).A total of 15 μg EV protein isolated from different groups along with MW marker was loaded on SDS-PAGE (Precast criterion gel, 8-16% gradient gel, BIO RAD). The gel was stained with Coomassie Brilliant Blue R250. We found very low amount of an abundant proteins in blood plasma i.e. albumin (66kD) in EV fraction. Densitometric analysis showed that there was equal loading of EV proteins from different groups. The full-length gel image is presented in **Supplementary Fig. S7A**. EV- Extracellular vesicles, GSD- Gallstone, XGC- Xanthogranulomatous cholecystitis, GBC- Gallbladder carcinoma, BSA- Bovine Serum Albumin.

Supplementary Fig. S3:

SDS-PAGE analysis showing protein profiling of EV and EV-depleted fraction of pooled plasma from healthy individuals or GSD or GBC stage IIIA and GBC stage IVB. A total of 15 μg EV protein isolated from different groups along with MW marker was loaded on SDS-PAGE (10% gel, BIO RAD). The gel was stained with Coomassie Brilliant Blue R250. We found very low amount of an abundant proteins in blood plasma i.e. albumin (66kD) in EV fraction in comparison to EV-depleted fraction. Densitometric analysis showed that there was equal loading of EV proteins from different groups. The full-length gel image is presented in **Supplementary Fig. S7B**. EV- Extracellular vesicles, GSD- Gallstone, GBC- Gallbladder carcinoma, BSA- Bovine Serum Albumin.

Supplementary Fig. S4:

Volcano plot showing differentially abundant proteins in early and advanced stage GBC in comparison to different control types (healthy, GSD and XGC). The volcano map was prepared by using log2 fold change and -log10 (p-value) as the co-ordinates and significant fold change $\geq$ 2.0 and p-value <0.05 were considered to screen the proteins. Dots in red, green/blue and grey represents proteins that are overexpressed, downregulated and unchanged respectively. GBC- Gallbladder carcinoma; GSD- Gallstone disease; XGC- Xanthogranulomatous cholecystitis.

Supplementary Fig. S5:

Flow chart showing the experimental design for identification of altered levels of EV proteins in GBC cases with early stages. EV- Extracellular vesicles; GBC- Gallbladder carcinoma; GSD- Gallstone disease ; XGC- Xanthogranulomatous cholecystitis.

Supplementary Fig. S6: Flow chart showing the experimental design for identification of altered levels of EV proteins in GBC cases with advanced stages. EV- Extracellular vesicles ; GBC- Gallbladder carcinoma; GSD- Gallstone disease.

Supplementary Fig. S7:

The full-length blot images of Fig. S2 and Fig. S3. (A) The full-length gel images of **Fig. S2 (B)** The full-length gel image of **Fig. S3**. The cropping of the gel images is indicated with red dashed line.

Supplementary Table S1: Clinical details of cases (GBC) and controls (Healthy individuals, GSD, XGC) used in the study. GBC- Gallbladder carcinoma; GSD- Gallstone disease ; XGC- Xanthogranulomatous cholecystitis, DM- Diabetis milletus, HTN- Hypertension, TB- Tuberculosis, LOA- Loss of appetite,
LOW- Loss of weight;
Foot note: NA- Not applicable, (-) Not available

Supplementary Table S2:

(A) Number of samples used to study the expression of NT5E, ANPEP and MME by ELISA

(B)) No. of samples used to study the expression of NT5E and MME by IHC analysis

Supplementary Table S3:

Non-redundant list of 86 plasma-derived EV proteins with altered levels in early and/or advanced stage GBC. The table shows protein localization (uniprot database), cancer association (HPA database and literature search), altered level in GBC tissue/ blood plasma/serum (based on literature) and altered levels in plasma-derived EVs in other cancers (based on literature).The list includes non-redundant EV proteins (≥2.0 fold change) derived from 42 proteins from early stage GBC and 57 proteins from advanced stage GBC cases. The details of these proteins/ peptides identified by mass spectrometry and fold changes are shown in **Supplementary Tables S3 and S4** respectively.

Supplementary Table S4:

List of plasma-derived EV proteins with altered levels in early stage GBC. A total of 42 EV proteins (≥2.0 fold change) were identified as detailed in Methods section. The Table describes the total number of proteins and peptides identified in mass spectrometry runs along with their quantity values**.**

Supplementary Table S5:

List of plasma-derived EV proteins with altered levels in advanced stage GBC. A total of 57 EV proteins (≥2.0 fold change) were identified as detailed in Methods section. The Table describes the total number of proteins and peptides identified in mass spectrometry runs along with their quantity values.

Supplementary Table S6:

Localization of 86 non-redundant plasma-derived EV proteins with altered levels in GBC using STRING database.

Supplementary Table S7:

The concentration of NT5E and ANPEP and MME in GBC cases and controls as determined by quantitative ELISA.

Supplementary Table S8:

Statistical analysis of quantitative ELISA data showing sensitivity and specificity of NT5E, ANPEP and MME for detection of GBC. (A) The statistical analysis performed using samples from the discovery study (proteomics study) as available and (B) an independent cohort (C) Combined cohort (discovery + independent cohort). The details of the samples are shown in **Supplementary Table S2.**

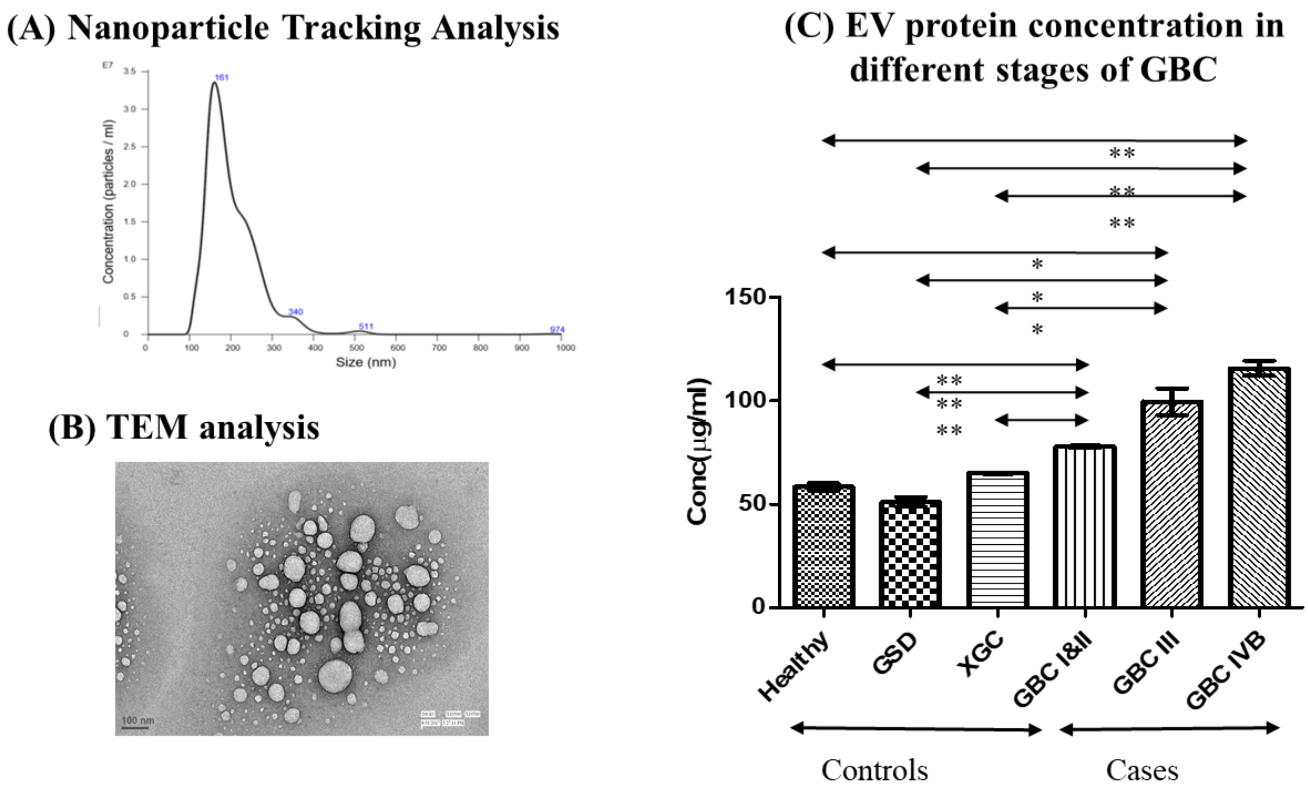

Supplementary Fig. S1:

Characterization of blood plasma-derived EVs (A) Size and particle distribution plots of EVs from human blood plasma-derived EVs using nanoparticle tracking system showed peaks at 161 nm suggesting enrichment of exosomes in EV fraction. (B) Transmission electron micrographs of plasma derived EVs. EVs from pooled plasma of healthy individuals isolated by ultracentrifugation method were resuspended in PBS and loaded on 2% collodion coated grids. Negative staining was performed using 2% phosphotungstic acid (PTA). Images of EVs (30-100 nm) were acquired using 120 KV Hitachi TEM 7500 at 1,04,000× magnification, scale bar- 100 nm. (C) Bar diagram showing EV protein amount in controls (healthy individuals, XGC, GSD) and cases (GBC stage I and II, stage IIIA, stage IVB). An increase in EV proteins was observed in both early and advanced stages of GBC cases in comparison to controls. * *p value* $\leq$ 0.05** *p value* $\leq$ 0.01.

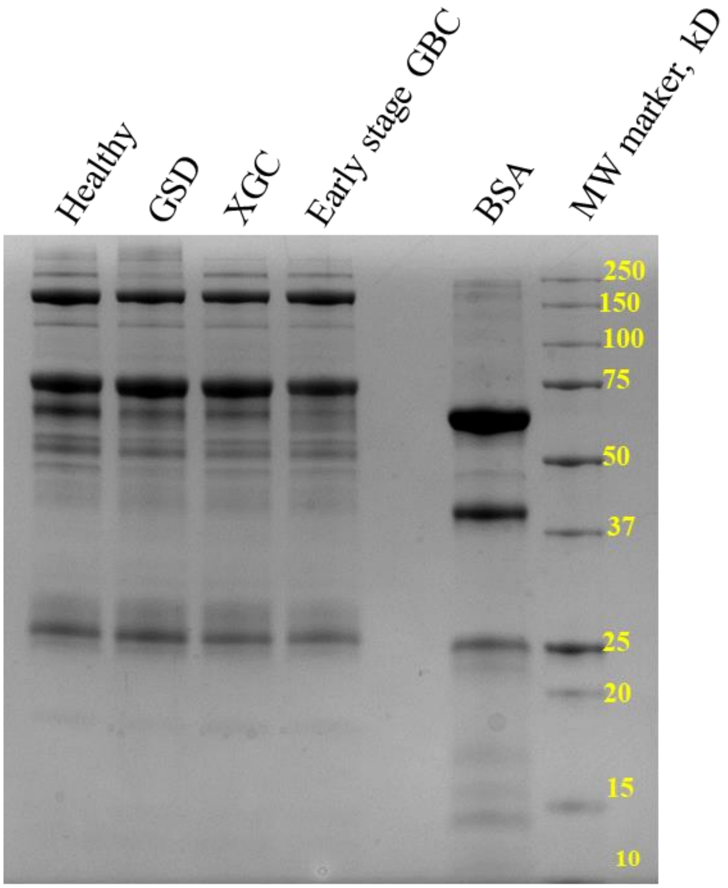

Supplementary Fig. S2:

SDS-PAGE analysis showing protein profiling of EV fraction of pooled plasma from healthy individuals or GSD or XGC or early stage GBC (stage I and II). A total of 15 µg EV protein isolated from different groups along with MW marker was loaded on SDS-PAGE (Precast criterion gel, 8-16% gradient gel, BIO RAD). The gel was stained with Coomassie Brilliant Blue R250. We found very low amount of an abundant proteins in blood plasma i.e. albumin (66kD) in EV fraction. Densitometric analysis showed that there was equal loading of EV proteins from different groups. The full-length gel image is presented in **Supplementary Fig. S7A**. EV- Extracellular vesicles, GSD- Gallstone, XGC- Xanthogranulomatous cholecystitis, GBC- Gallbladder carcinoma, BSA- Bovine Serum Albumin.

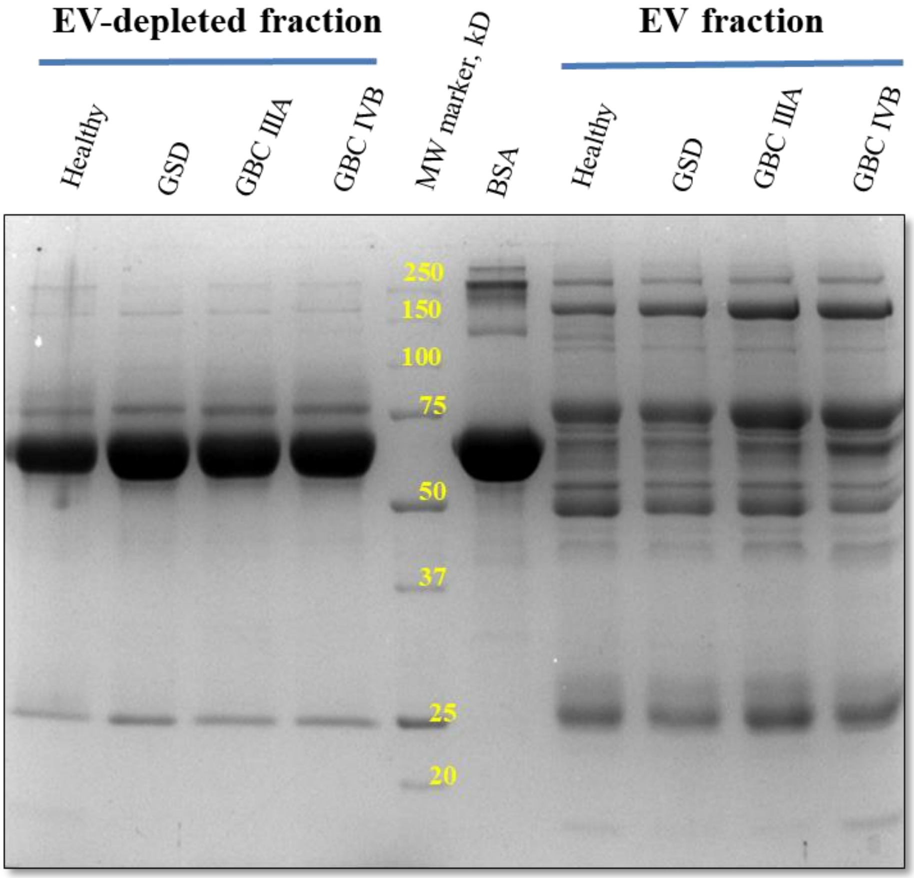

Supplementary Fig. S3:

SDS-PAGE analysis showing protein profiling of EV and EV-depleted fraction of pooled plasma from healthy individuals or GSD or GBC stage IIIA and GBC stage IVB. A total of 15 µg EV protein isolated from different groups along with MW marker was loaded on SDS-PAGE (10% gel, BIO RAD). The gel was stained with Coomassie Brilliant Blue R250. We found very low amount of an abundant proteins in blood plasma i.e. albumin (66kD) in EV fraction in comparison to EV-depleted fraction. Densitometric analysis showed that there was equal loading of EV proteins from different groups. The full-length gel image is presented in **Supplementary Fig. S7B**. EV- Extracellular vesicles, GSD- Gallstone, GBC- Gallbladder carcinoma, BSA- Bovine Serum Albumin.

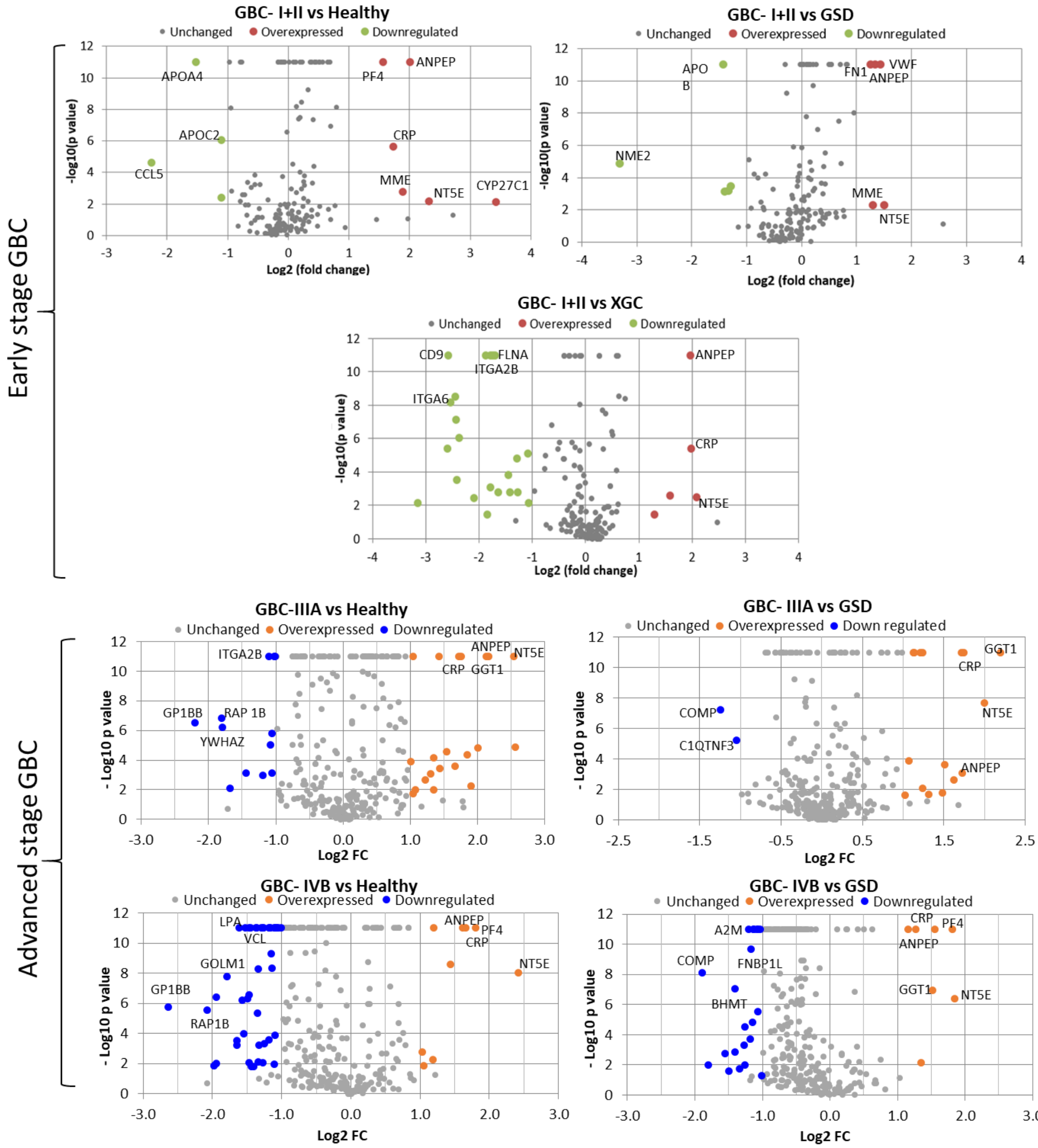

Supplementary Fig. S4:

Volcano plot showing differentially abundant proteins in early and advanced stage GBC in comparison to different control types (healthy, GSD and XGC). The volcano map was prepared by using log2 fold change and -log10 (p-value) as the co-ordinates and significant fold change ≥ 2.0 and p-value <0.05 were considered to screen the proteins. Dots in red, green/blue and grey represents proteins that are overexpressed, downregulated and unchanged respectively. GBC- Gallbladder carcinoma; GSD- Gallstone disease; XGC- Xanthogranulomatous cholecystitis.

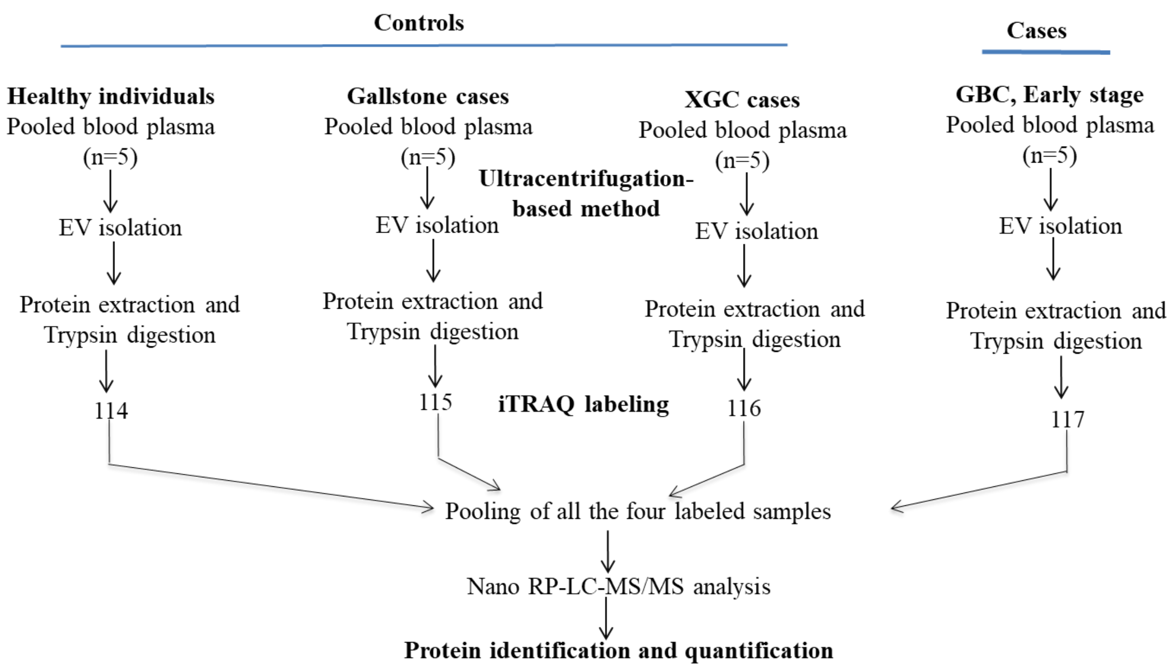

Supplementary Fig. S5:

Flow chart showing the experimental design for identification of altered levels of EV proteins in GBC cases with early stages. EV- Extracellular vesicles; GBC- Gallbladder carcinoma; GSD- Gallstone disease ; XGC- Xanthogranulomatous cholecystitis.

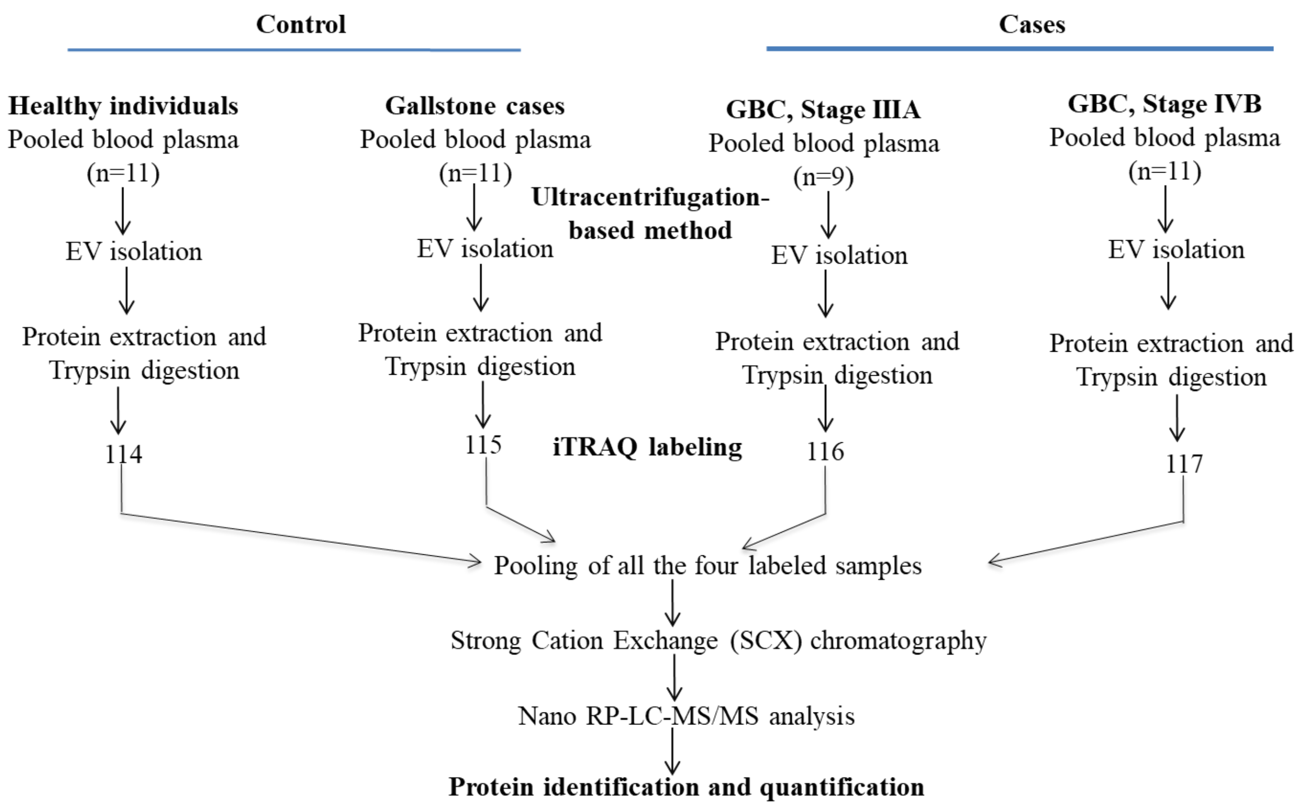

Supplementary Fig. S6: Flow chart showing the experimental design for identification of altered levels of EV proteins in GBC cases with advanced stages. EV- Extracellular vesicles ; GBC- Gallbladder carcinoma; GSD- Gallstone disease.

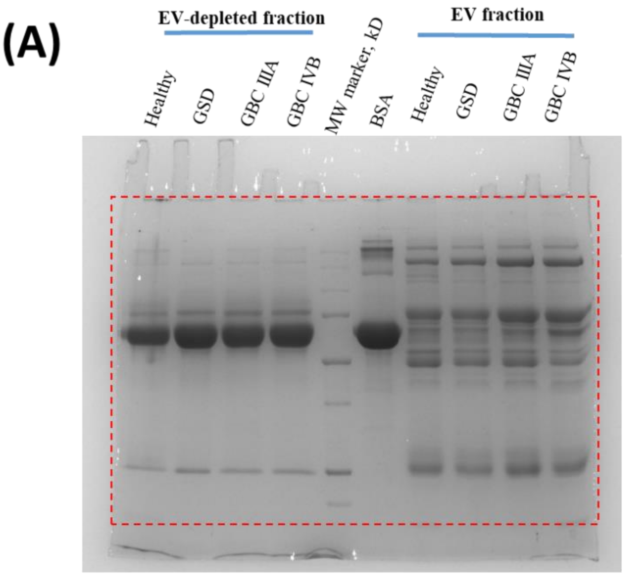

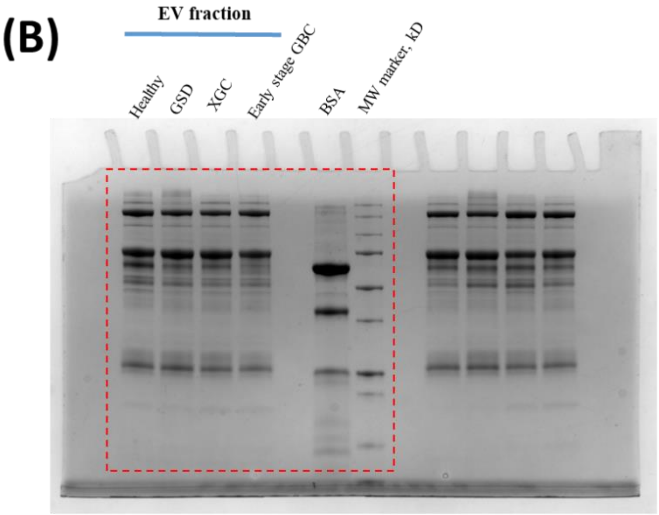

Supplementary Fig. S7:

The full-length blot images of Fig. S2 and Fig. S3. (A) The full-length gel images of **Fig. S2** (B) The full-length gel image of **Fig. S3**. The cropping of the gel images is indicated with red dashed line.

Supplementary Table S1: Clinical details of cases (GBC) and controls (Healthy individuals, GSD, XGC) used in the study. GBC- Gallbladder carcinoma; GSD- Gallstone disease ; XGC- Xanthogranulomatous cholecystitis, DM- Diabetis milletus, HTN- Hypertension, TB- Tuberculosis, LOA- Loss of appetite, LOW- Loss of weight; Foot note: NA- Not applicable, (-) Not available

S.No.	Patient Code	Sex	Age	Group	TNM	Stage	Grade	Discovery (proteomics) / Verification (ELISA/IHC)	TLC	DLC	Bilirubin	AST	ALT	ALP	Jaundice	Co-morbidities (DM, HTN,TB, Asthma, LOA, LOW, thyroid disease)	ELISA- ANPEP (Positive/ Negative)	ELISA- NT5E (Positive/ Negative)	ELISA- MME (Positive/ Negative)
1	GN001	Male	42	Early GBC	pT1N0M0	I	WDAC	Discovery and Verification	8400	60/30	1.6	20	22	181	YES	NO	Positive	Positive	Positive
2	GN002	Female	47	Early GBC	pT1bN0M0	I	WDAC	Verification only	9600	63/31	0.6	23	20	90	NO	NO	Negative	Negative	Positive
3	GN003	Female	38	Early GBC	pTis N0M0	I	WDAC	Verification only	7650	69/21	0.5	16	32	76	NO	NO	Negative	Negative	Positive
4	GN004	Female	45	Early GBC	pT1bN0M0	I	WDAC	Verification only	14700	96/1	0.3	55	48	230	NO	YES (Diabetis, on tab Glimperide 2mg OD)	Negative	Negative	Positive
5	GN005	Female	45	Early GBC	pT1bNxM0	I	WDAC	Verification only	–	–	–	–	–	–	–	–	Negative	Negative	Negative
6	GN006	Male	55	Early GBC	pT1bN0M0	I	WDAC	Verification only	–	–	–	–	–	–	–	–	Positive	Negative	Negative
7	GN007	Female	53	Early GBC	pT1bN0M0	I	WDAC	Verification only	–	–	–	–	–	–	–	–	Positive	Negative	Positive
8	GN008	Female	44	Early GBC	pTisN0M0	I	WDAC	Verification only	–	–	–	–	–	–	–	–	Negative	Negative	Positive
9	GN009	Female	60	Early GBC	pT2N0M0	II	WDAC	Discovery and Verification	15900	90/07	0.2	14	8	124	NO	YES (Diabetis, on tab Glimeperide 2mg OD)	Negative	Negative	Positive
10	GN010	Female	34	Early GBC	pT2AN0M0	II	MDAC	Discovery and Verification	9700	62/34	0.6	26	18	102	NO	NO	Negative	Negative	Negative
11	GN011	Female	55	Early GBC	pT2aN0M0	II	MDAC	Discovery and Verification	–	–	–	–	–	–	–	–	Negative	Negative	Negative
12	GN012	Female	65	Early GBC	pT2aN0M0	IIA	WDAC	Verification only	–	–	–	–	–	–	–	–	Negative	Negative	Negative
13	GN013	Female	36	Early GBC	pT2N0M0	IIA	MDAC	Verification only	–	–	–	–	–	–	–	–	Negative	Negative	Positive
14	GN014	Female	66	Advanced GBC	pT3N0M0	IIIA	PDAC	Discovery and Verification	17900	91/06	1.6	39	16	287	NO	YES (DM-HTN)	Negative	Negative	–
15	GN015	Female	65	Advanced GBC	pT3N0M0	IIIA	MDAC	Discovery and Verification									Positive	Negative	–
16	GN016	Female	30	Advanced GBC	pT3 N0M0	IIIA	MDAC	Discovery and Verification	5700	59/4	1	49	186	145	NO	YES {TB (+) pulmonary koch's, took ATT, anorexia or weight loss}.	Negative	Negative	–
17	GN017	Female	50	Advanced GBC	pT3N0M0	IIIA	PDAC	Discovery and Verification	11100	72/14	1.5	10	5	145	NO	YES (Weight loss)	Negative	Negative	–
18	GN018	Female	60	Advanced GBC	pT3N0M0	IIIA	MDAC	Discovery and Verification	–	–	–	–	–	–	–	–	Negative	Negative	–
19	GN019	Female	51	Advanced GBC	pT3N0M0	IIIA	MDAC	Discovery and Verification	–	–	–	–	–	–	–	–	Negative	Negative	–
20	GN020	Female	42	Advanced GBC	pT3N0M0	IIIA	MDAC	Discovery and Verification	–	–	–	–	–	–	–	–	Negative	Positive	–
21	GN021	Female	48	Advanced GBC	pT3N0M0	IIIA	MDAC	Discovery and Verification	–	–	–	–	–	–	–	–	Negative	Negative	–
22	GN022	Female	42	Advanced GBC	pT3N0M0	IIIA	MDAC	Discovery only	–	–	–	–	–	–	–	–			–
23	GN023	Female	48	Advanced GBC	pT3N0M0	IIIA	MDAC	Verification only	5100	72/22	0.4	43	18	148	NO	YES (DM on OAH (metformin), asthma on inhaler, on ATT for cervical lymphadenopathy)	Positive	Negative	–
24	GN024	Female	57	Advanced GBC	pT3N0M0	IIIA	PDAC	Verification only	4900	64/24	0.3	22	14	NA	NO	YES (LOA)	Positive	Negative	–
25	GN025	Female	65	Advanced GBC	pT3N0M0	IIIA	MDAC	Verification only	9400	66/22	0.3	16	8	81	NO	NO	Negative	Negative	–
26	GN026	Female	49	Advanced GBC	pT3N0M0	IIIA	MDAC	Verification only	8000	76/12	0.7	34	25	126	NO	YES (LOA, LOW)	Negative	Positive	–
27	GN027	Male	48	Advanced GBC	pT3N1Mx	IIIB	MDAC	Verification only	5800	66/19	0.2	18	5	68	NO	NO	Positive	Positive	–
28	GN028	Female	62	Advanced GBC	pT2aN1Mx	IIIB	WDAC	Verification only	7600	72/20	1	20	22	126	NO	YES (HTN)	Negative	Negative	–
29	GN029	Female	54	Advanced GBC	pT3N1Mx	IIIB	MDAC	Verification only	12300	70/24	10.8	138	81	324	YES	NO	Negative	Negative	–
30	GN030	Female	45	Advanced GBC	pT3N1Mx	IIIB	MDAC	Verification only	–	–	–	–	–	–	–	–	Positive	Negative	–
42	GN042	Female	55	Advanced GBC	pT4N0Mx	IVA	MDAC	Verification only	–	–	–	–	–	–	–	–	Positive	Positive	–
43	GN043	Female	63	Advanced GBC	pT4N0Mx	IVA	WDAC	Verification only	–	–	–	–	–	–	–	–	Positive	Positive	–
31	GN031	Female	45	Advanced GBC	pT2N2M0	IVB	MDAC	Discovery and Verification	6700	52/35	0.3	54	55	155	NO	YES (HTN)	Negative	Negative	–
32	GN032	Female	52	Advanced GBC	pT4N0M1	IVB	MDAC	Discovery and Verification	17100	76/16	0.4	9	13	178	NO	YES {2 episodes LOW (+), LOA (+). Asthma -(on inhaler and medication)}	Negative	Negative	–
33	GN033	Female	58	Advanced GBC	pTxNxM1	IVB	PDAC	Discovery and Verification	–	–	–	–	–	–	–	–	Positive	Negative	–
34	GN034	Female	55	Advanced GBC	pT2b N1 M1	IVB	MDAC	Discovery and Verification	8400	48/33	0.3	32	22	241	NO	NO	Negative	Negative	–
35	GN035	Female	78	Advanced GBC	pT3N1M1	IVB	PDAC	Discovery and Verification	–	–	–	–	–	–	–	–	Positive	Positive	–
36	GN036	Male	38	Advanced GBC	pT3N1M1	IVB	MDAC	Discovery and Verification	–	–	–	–	–	–	–	–	Negative	Negative	–
37	GN037	Female	60	Advanced GBC	pT3N0M1	IVB	PDAC	Discovery and Verification	–	–	–	–	–	–	–	–	Negative	Negative	–
38	GN038	Female	45	Advanced GBC	pTxNxM1	IVB	PDAC	Discovery and Verification	–	–	–	–	–	–	–	–	Positive	Positive	–

39	GN039	Female	45	Advanced GBC	pT4N1M1	IVB	MDAC	Discovery and Verification	10400	80/11	3.1	32	30	115	YES	NO	Negative	Positive	–
40	GN040	Female	52	Advanced GBC	pTxNxM1	IVB	PDAC	Discovery and Verification	7600	68/26	0.5	47	63	98	NO	YES (HTN)	Negative	Negative	–
41	GN041	Female	64	Advanced GBC	pT4N1M1	IVB	PDAC	Discovery and Verification	–	–	–	–	–	–	–	–	Negative	Negative	–
44	GN044	Female	42	Advanced GBC	pT4NxM1	IVB	MDAC	Verification only	5800	64/30	5.6	106	65	NA	YES	NO	Positive	Negative	–
45	GN045	Female	62	Advanced GBC	pT3N2Mx	IVB	MDAC	Verification only	8900	66/26	1.1	34	53	110	NO	YES (HTN)	Positive	Negative	–
46	GN046	Female	41	Advanced GBC	pT3NxM1	IVB	MDAC	Verification only	12600	59/36	2	44	18	174	NO	YES (LOA, LOW)	Positive	Negative	–
47	GN047	Female	53	Advanced GBC	pT3N1M1	IVB	PDAC	Verification only	10500	60/31	0.3	22	17	148	NO	NO	Positive	Negative	–
48	GN048	Male	54	Advanced GBC	pTxNxM1	IVB	WDAC	Verification only	2700	49/41	1	72	73	287	NO	YES (anorexia)	Negative	Negative	–
49	GN049	Male	42	Advanced GBC	pTxNxM2	IVB	PDAC	Verification only	6400	74/37	1.6	123	134	574	NO	NO	Negative	Negative	–
50	GN050	Female	49	Advanced GBC	pT3N2Mx	IVB	WDAC	Verification only	9800	66/20	0.2	10	7	NA	NO	YES {hypothyroid on T. Elthroxin 100mcg OD, seizure disorder on T. Levara 500mg BD}	Negative	Negative	–
51	GN051	Female	38	Advanced GBC	pT3NxM1	IVB	MDAC	Verification only	6900	73/21	0.3	25	31	99	NO	NO	Negative	Negative	–
52	GN052	Male	63	Advanced GBC	pTxNxM1	IVB	PDAC	Verification only	–	–	–	–	–	–	–	–	Positive	Positive	–
53	GN053	Male	54	Advanced GBC	pTxNxM1	IVB	PDAC	Verification only	–	–	–	–	–	–	–	–	Positive	Positive	–
54	GN054	Female	50	Advanced GBC	pTxNxM1	IVB	PDAC	Verification only	6400	62/26	1.7	28	16	61	NO	NO	Positive	Positive	–
55	GN055	Female	50	Advanced GBC	pTxNxM1	IVB	PDAC	Verification only	6400	74	1	123	134	76	NO	NO	Positive	Negative	–
56	GN056	Male	50	Advanced GBC	pT2aNxM1	IVB	MDAC	Verification only	12000	80/16	3	99	88	NA	NO	NO	Positive	Positive	–
57	GN057	Male	60	GSD	NA	NA	NA	Discovery only									–	–	–
58	GN058	Female	62	GSD	NA	NA	NA	Discovery and Verification	7200	82/28	0.2	21	27	68	NO	NO	Negative	Negative	–
59	GN059	Male	51	GSD	NA	NA	NA	Discovery and Verification	6400	72/24	0.4	14	17	174	NO	NO	Negative	Negative	–
60	GN060	Female	50	GSD	NA	NA	NA	Discovery and Verification	7800	55/32	0.8	24	26	118	NO	NO	Negative	Negative	Negative
61	GN061	Female	45	GSD	NA	NA	NA	Discovery and Verification	5800	58/26	0.3	28	27	302	NO	NO	Negative	Negative	Negative
62	GN062	Female	50	GSD	NA	NA	NA	Discovery and Verification	7200	60/33	0.3	17	27	124	NO	NO	Negative	Negative	Negative
63	GN063	Female	40	GSD	NA	NA	NA	Discovery and Verification	8300	66/30	0.7	30	27	132	YES	NO	Negative	Negative	–
64	GN064	Female	70	GSD	NA	NA	NA	Discovery and Verification	5100	60/29	0.5	24	12	74	NO	NO	Negative	Negative	–
65	GN065	Female	30	GSD	NA	NA	NA	Discovery and Verification	6500	74/21	0.5	18	7	63	NO	NO	Negative	Negative	Negative
66	GN066	Female	35	GSD	NA	NA	NA	Discovery and Verification									Negative	Negative	
67	GN067	Female	56	GSD	NA	NA	NA	Discovery only	9700	59/32	0.6	24	19	74	NO	YES {DM (taking tab metformin 500mg)}	–	–	–
68	GN068	Male	40	GSD	NA	NA	NA	Discovery only	4300	56/24	0.6	37	40	74	NO	NO	–	–	–
69	GN069	Male	60	GSD	NA	NA	NA	Discovery only	7700	68/19	0.6	19	30	68	NO	NO	–	–	–
70	GN070	Female	35	GSD	NA	NA	NA	Discovery only	7700	72/22	0.6	86	42	85	NO	NO	–	–	–
71	GN071	Female	39	GSD	NA	NA	NA	Discovery only									–	–	–
72	GN072	Female	43	GSD	NA	NA	NA	Verification only	8400	71/21	6.7	165	134	528	YES	NO	Negative	Negative	Negative
73	GN073	Female	40	GSD	NA	NA	NA	Verification only	9900	17/24	0.5	47	90	124	NO	NO	Negative	Negative	Negative
74	GN074	Female	37	GSD	NA	NA	NA	Verification only	9900	70/24	0.5	47	90	104	NO	NO	Negative	Negative	–
75	GN075	Female	40	GSD	NA	NA	NA	Verification only	8600	78/15	0.7	37	41	108	NO	NO	Negative	Negative	Negative
76	GN076	Female	30	GSD	NA	NA	NA	Verification only	–	–	–	–	–	–	–	–	Negative	Negative	Negative
77	GN077	Male	34	GSD	NA	NA	NA	Verification only	–	–	–	–	–	–	–	–	Negative	Negative	–
78	GN078	Male	54	GSD	NA	NA	NA	Verification only	–	–	–	–	–	–	–	–	Negative	Negative	–
79	GN079	Female	22	GSD	NA	NA	NA	Verification only	–	–	–	–	–	–	–	–	Positive	Negative	–
80	GN080	Male	35	XGC	NA	NA	NA	Discovery and Verification	7200	64/28	0.5	35	34	113	YES	YES {cardiac event (Mural Thrombus), on cardiac medications}	Negative	Negative	Negative
81	GN081	Female	63	XGC	NA	NA	NA	Discovery and Verification	6600	74/18	0.7	16	11	73	NO	YES [DM, is on OHA , HTN on hypertensives, clopidogrel & atovastatine, pulmonary TB, took ATT].	Negative	Negative	Negative
82	GN082	Female	55	XGC	NA	NA	NA	Discovery and Verification	–	–	–	–	–	–	–	–	Negative	Negative	Negative
83	GN083	Female	42	XGC	NA	NA	NA	Discovery and Verification	–	–	–	–	–	–	–	–	Negative	Negative	Negative
84	GN084	Male	71	XGC	NA	NA	NA	Discovery only	–	–	–	–	–	–	–	–	–	–	–
85	GN085	Male	40	XGC	NA	NA	NA	Verification only	7500	56/33	0.5	21	26	154	NO	NO	Negative	Negative	Negative
86	GN086	Female	26	XGC	NA	NA	NA	Verification only	7800	67/30	0.6	66	80	112	NO	NO	Negative	Negative	Negative
87	GN087	Male	62	XGC	NA	NA	NA	Verification only	5300	49/42	0.3	19	20	87	NO	NO	Negative	Negative	Negative
88	GN088	Male	63	XGC	NA	NA	NA	Verification only	9600	70/20	0.3	28	22	68	NO	NO	Negative	Negative	Negative
89	GN089	Female	42	Healthy	NA	NA	NA	Discovery and Verification	–	–	–	–	–	–	NO	NO	Negative	Negative	Positive
90	GN090	Female	29	Healthy	NA	NA	NA	Discovery and Verification	–	–	–	–	–	–	NO	NO	Negative	Negative	Negative
91	GN091	Male	39	Healthy	NA	NA	NA	Discovery and Verification	–	–	–	–	–	–	NO	YES (Hypothyrodism)	Negative	Negative	Negative
92	GN092	Female	48	Healthy	NA	NA	NA	Discovery and Verification	–	–	–	–	–	–	NO	NO	Negative	Negative	–

93	GN093	Female	53	Healthy	NA	NA	NA	Discovery and Verification	–	–	–	–	–	–	NO	NO	Negative	Negative	–
94	GN094	Female	37	Healthy	NA	NA	NA	Discovery and Verification	–	–	–	–	–	–	NO	NO	Negative	Negative	Negative
95	GN095	Female	46	Healthy	NA	NA	NA	Discovery and Verification	–	–	–	–	–	–	NO	NO	Negative	Negative	–
96	GN096	Female	46	Healthy	NA	NA	NA	Discovery and Verification	–	–	–	–	–	–	NO	NO	Negative	Negative	–
97	GN097	Male	59	Healthy	NA	NA	NA	Discovery and Verification	–	–	–	–	–	–	NO	NO	Negative	Negative	–
98	GN098	Female	42	Healthy	NA	NA	NA	Discovery and Verification	–	–	–	–	–	–	NO	NO	Negative	Negative	Negative
99	GN099	Female	46	Healthy	NA	NA	NA	Discovery and Verification	–	–	–	–	–	–	NO	NO	Negative	Negative	Negative
100	GN100	Female	38	Healthy	NA	NA	NA	Discovery only	–	–	–	–	–	–	NO	YES (Hypothyrodism)	–	–	–
101	GN101	Male	36	Healthy	NA	NA	NA	Discovery only	–	–	–	–	–	–	NO	NO	–	–	–
102	GN102	Female	48	Healthy	NA	NA	NA	Discovery only	–	–	–	–	–	–	NO	NO	–	–	–
103	GN103	Female	48	Healthy	NA	NA	NA	Discovery only	–	–	–	–	–	–	NO	NO	–	–	–
104	GN104	Female	56	Healthy	NA	NA	NA	Discovery only	–	–	–	–	–	–	NO	NO	–	–	–
105	GN105	Female	29	Healthy	NA	NA	NA	Verification only	–	–	–	–	–	–	NO	NO	Negative	Negative	Negative
106	GN106	Female	25	Healthy	NA	NA	NA	Verification only	–	–	–	–	–	–	NO	NO	Negative	Negative	Negative
107	GN107	Male	39	Healthy	NA	NA	NA	Verification only	–	–	–	–	–	–	NO	NO	Negative	Positive	–
108	GN108	Female	56	Healthy	NA	NA	NA	Verification only	–	–	–	–	–	–	NO	NO	Negative	Negative	–
109	GN109	Female	53	Healthy	NA	NA	NA	Verification only	–	–	–	–	–	–	NO	NO	Negative	Negative	–
110	GN110	Female	30	Healthy	NA	NA	NA	Verification only	–	–	–	–	–	–	NO	NO	Negative	Negative	–
111	GN111	Female	31	Healthy	NA	NA	NA	Verification only	–	–	–	–	–	–	NO	NO	Negative	Negative	–
112	GN112	Female	35	Healthy	NA	NA	NA	Verification only	–	–	–	–	–	–	NO	NO	Negative	Negative	–
113	GN113	Female	52	Healthy	NA	NA	NA	Verification only	–	–	–	–	–	–	NO	NO	Negative	Negative	–

Supplementary Table S2:

(A) Number of samples used to study the expression of NT5E, ANPEP and MME by ELISA

(i) Number of plasma samples used in ELISA for NT5E and ANPEP

	**Healthy**	**GSD**	**XGC**	**Early stage GBC** (Stage I and II)	**GBC III**	**GBC IV**	**Advanced stage GBC** (Stage III and IV)	**All GBC**	**All Controls** (Healthy, GSD, XGC)
**D**	11	9	-	-	8	11	19	19	20
**IC**	9	8	8	13	8	15	23	36	25
**Total (D+IC)**	20	17	8	13	16	26	42	55	45

(ii) Number of plasma samples used in ELISA for MME

	**Healthy**	**GSD**	**XGC**	**Early stage GBC** (Stage I and II)	**All GBC**	**All Controls** (Healthy, GSD, XGC)
**D**	6	4	4	4	4	14
**IC**	2	4	4	9	9	10
**Total (D + IC)**	8	8	8	13	13	24

D- The discovery study samples used for verification
IC- The independent cohort used for verification
GSD- Gallstone disease cases
XGC- Xanthogranulomatous cholecystitis

(B) No. of samples used to study the expression of NT5E and MME by IHC analysis

	Early stage GBC	Advanced stage GBC	All GBC	All Controls (GSD and XGC)
Total	13	34	47	23

Supplementary Table S3:

Non-redundant list of 86 plasma-derived EV proteins with altered levels in early and/or advanced stage GBC. The table shows protein localization (uniprot database), cancer association (HPA database and literature search), altered level in GBC tissue/ blood plasma/serum (based on literature) and altered levels in plasma-derived EVs in other cancers (based on literature).The list includes non-redundant EV proteins (≥2.0 fold change) derived from 42 proteins from early stage GBC and 57 proteins from advanced stage GBC cases. The details of these proteins/ peptides identified by mass spectrometry and fold changes are shown in **Supplementary Tables S4 and S5** respectively.

Gene Symbol	Protein name	Altered levels in GBC (based on EV proteomics data)	Protein localization	Cancer association		Reported in GBC	Reported in EVs in other cancers
				HPA data	literature survey		
ACTG1	Actin, cytoplasmic 2	Early stage and Advanced stage IIIA and IVB	Intracellular	-	Yes	No	Yes
ANPEP	Aminopeptidase N	Early stage and Advanced stage IIIA and IVB	Membrane	-	Yes	No	Yes
CRP	C-reactive protein	Early stage and Advanced stage IIIA and IVB	Intracellular, secreted	Yes	Yes	Yes-Serum, bile	No
FLNA	PREDICTED: filamin-A isoform X5	Early stage and Advanced stage IIIA and IVB	Intracellular	-	Yes	No	Yes
ITGA2B	Integrin alpha-iib	Early stage and Advanced stage IIIA and IVB	Intracellular, membrane	Yes	Yes	No	No
NT5E	5'-nucleotidase isoform 2	Early stage and Advanced stage IIIA and IVB	Intracellular, membrane	-	Yes	Yes- Tissue	Yes
ADAM10	PREDICTED: disintegrin and metalloproteinase domain-containing protein 10 isoform X1	Early stage	Intracellular, membrane	Yes	Yes	No	Yes
ALAD	Delta-aminolevulinic acid dehydratase isoform c	Early stage	Intracellular	Yes	Yes	No	Yes
ANGPTL6	Angiopoietin-related protein 6	Early stage	secreted	-	Yes	No	No
APOA4	Apolipoprotein A-IV	Early stage	secreted	-	Yes	No	Yes
APOC3	Apolipoprotein C-III	Early stage	secreted	Yes	Yes	No	Yes
APOM	Apolipoprotein M isoform 2	Early stage	Intracellular, secreted	-	Yes	No	No
B2M	Beta-2-microglobulin	Early stage	Intracellular	Yes	Yes	Yes- Tissue	Yes
C6orf25	Protein G6b isoform g6b-B	Early stage	Intracellular, membrane	-	Yes	No	No
CCL5	C-C motif chemokine 5 isoform 1	Early stage	Membrane, secreted	Yes	Yes	No	No
CD9	PREDICTED: CD9 antigen isoform X2	Early stage	Membrane	Yes	Yes	Yes- Tissue	Yes
CYP27C1	PREDICTED: cytochrome P450 27C1 isoform X1	Early stage	Intracellular	-	No	No	No
DCTN2	PREDICTED: dynactin subunit 2 isoform X1	Early stage	Intracellular	-	Yes	No	No
F11R	Junctional adhesion molecule A	Early stage	Membrane	-	Yes	No	Yes

FERMT3	PREDICTED: fermitin family homolog 3 isoform X2	Early stage	Intracellular	-	Yes	No	Yes
FKBP15	PREDICTED: FK506-binding protein 15 isoform X3	Early stage	Intracellular	-	No	No	No
FN1	Fibronectin isoform 6	Early stage	Intracellular, secreted	Yes	Yes	Yes- Urine	Yes
HSPA8	Heat shock cognate 71 kda protein isoform 1	Early stage	Intracellular	Yes	Yes		Yes
ITGA6	PREDICTED: integrin alpha-6 isoform X6	Early stage	Intracellular, membrane	-	Yes	Yes- Tissue	No
ITGB1	Integrin beta-1 isoform 1A	Early stage	Intracellular, membrane	Yes	Yes	No	Yes
MME	Neprilysin	Early stage	Intracellular, membrane	Yes	Yes	No	Yes
NME2	Nucleoside diphosphate kinase B isoform b	Early stage	Intracellular	Yes	Yes	No	Yes
PARVB	PREDICTED: beta-parvin isoform X2	Early stage	Intracellular, membrane	Yes	Yes	Yes- Tissue	Yes
PKM	Pyruvate kinase PKM isoform d	Early stage	Intracellular	Yes	Yes	Yes- Tissue	Yes
SAA1	Serum amyloid A-1 protein	Early stage	secreted	-	Yes	No	No
SAA4	Serum amyloid A-4 protein	Early stage	secreted	-	Yes	No	No
TLN1	Talin-1	Early stage	Intracellular	-	Yes	No	Yes
TUBB4A	Tubulin beta-4A chain isoform 4	Early stage	Intracellular	-	Yes	No	No
VCP	Transitional endoplasmic reticulum atpase	Early stage	Intracellular	-	Yes	No	No
YWHAB	PREDICTED: 14-3-3 protein beta/alpha isoform X2	Early stage	Intracellular	Yes	Yes	No	Yes
ABL1	Tyrosine-protein kinase ABL1 isoform a	Advanced GBC stage IVB	Intracellular	Yes	Yes	Yes- Tissue	No
ALB	Serum albumin	Advanced GBC stage IVB	Intracellular, secreted	Yes	Yes	Yes- Tissue	Yes
AZGP1	Zinc-alpha-2-glycoprotein	Advanced GBC stage IVB	Intracellular, secreted	Yes	Yes	No	Yes
C7	Complement component C7	Advanced GBC stage IVB	secreted	Yes	Yes	No	Yes
ECM1	Extracellular matrix protein 1 isoform 1	Advanced GBC stage IVB	secreted	-	Yes	Yes- Tissue	Yes
FGA	Fibrinogen alpha chain isoform alpha	Advanced GBC stage IVB	secreted	Yes	Yes	No	Yes
FGG	Fibrinogen gamma chain isoform gamma-A	Advanced GBC stage IVB	Intracellular, secreted	Yes	Yes	No	Yes
FTH1	Ferritin heavy chain	Advanced GBC stage IVB	Intracellular	Yes	Yes	No	Yes
FTL	Ferritin light chain	Advanced GBC stage IVB	Intracellular	Yes	Yes	No	Yes
GOLM1	Golgi membrane protein 1	Advanced GBC stage IVB	Intracellular	-	Yes	Yes- Tissue	No

NDST1	PREDICTED: bifunctional heparan sulfate N-deacetylase/N-sulfotransferase 1 isoform X8	Advanced GBC stage IVB	Intracellular, membrane	-	Yes	No	No
PIGR	Polymeric immunoglobulin receptor	Advanced GBC stage IVB	membrane	Yes	Yes	No	Yes
PSMA1	Proteasome subunit alpha type-1 isoform 2	Advanced GBC stage IVB	Intracellular	-	Yes	No	Yes
PSMA5	Proteasome subunit alpha type-5 isoform 1	Advanced GBC stage IVB	Intracellular	-	Yes	No	Yes
PSMB1	Proteasome subunit beta type-1	Advanced GBC stage IVB	Intracellular	-	Yes	No	Yes
RAC2	PREDICTED: ras-related C3 botulinum toxin substrate 2 isoform X1	Advanced GBC stage IVB	Intracellular	-	Yes	No	No
SAA2	Serum amyloid A-2 protein isoform a	Advanced GBC stage IVB	secreted	-	Yes	Yes- Serun and bile	No
TPM4	Tropomyosin alpha-4 chain isoform Tpm4.2cy	Advanced GBC stage IVB	Intracellular	Yes	Yes	No	Yes
VASP	PREDICTED: vasodilator-stimulated phosphoprotein isoform X3	Advanced GBC stage IVB	Intracellular	-	Yes	No	Yes
VCL	Vinculin isoform VCL	Advanced GBC stage IVB	Intracellular	-	Yes	No	Yes
ALDH1L1	PREDICTED: cytosolic 10-formyltetrahydro folate dehydrogenase isoform X3	Advanced GBC stage IIIA	Intracellular	-	Yes	No	No
HIST1H2B D	Histone H2B type 1-D	Advanced GBC stage IIIA	Intracellular	-	Yes	No	Yes
ILK	Integrin-linked protein kinase isoform 3	Advanced GBC stage IIIA	Intracellular	Yes	Yes	Yes- Tissue	Yes
HIST1H2AJ	Histone cluster 1, h2aj	Advanced GBC stage IIIA	Intracellular	-	No	No	No
HIST2H3D	Histone H3.2	Advanced GBC stage IIIA	Intracellular	-	Yes	No	No
HIST2H4A	Histone H4	Advanced GBC stage IIIA	Intracellular	-	No	No	No
HP	Haptoglobin isoform 1	Advanced GBC stage IIIA	Intracellular, secreted	Yes	Yes	Yes- Serum	Yes
PPBP	Platelet basic protein	Advanced GBC stage IIIA	secreted	-	Yes	No	No
XPNPEP2	Xaa-Pro aminopeptidase 2	Advanced GBC stage IIIA	Intracellular	-	Yes	No	No
BHMT	Betaine--homocysteine S-methyltransferase 1	Advanced GBC stage IIIA and IVB	Intracellular	-	Yes	No	No
CFL1	Cofilin-1	Advanced GBC stage IIIA and IVB	Intracellular	-	Yes	Yes- Tissue	Yes
COMP	Cartilage oligomeric matrix protein	Advanced GBC stage IIIA and IVB	Intracellular, secreted	-	Yes	No	No
ALPL	Alkaline phosphatase, tissue-nonspecific isozyme isoform 2	Advanced GBC stage IIIA and IVB	Intracellular, membrane	Yes	Yes	Yes- Serum	No
RAP1B	Ras-related protein Rap-1b isoform 4	Advanced GBC stage IIIA and IVB	Intracellular	-	Yes	No	Yes
DPP4	PREDICTED: dipeptidyl peptidase 4 isoform X1	Advanced GBC stage IIIA and IVB	Intracellular, membrane, secreted	-	Yes	No	Yes

GGT1	Gamma-glutamyltranspeptidase 1	Advanced GBC stage IIIA and IVB	Intracellular, membrane	-	Yes	Yes- Tissue	Yes
GP1BB	PlateletglycoproteinIb beta chain	Advanced GBC stage IIIA and IVB	Intracellular	-	No	No	No
MSN	PREDICTED: moesin isoform X3	Advanced GBC stage IIIA and IVB	Intracellular	Yes	Yes	No	Yes
PFN1	Profilin-1	Advanced GBC stage IIIA and IVB	Intracellular	-	Yes	No	Yes
SORD	Sorbitol dehydrogenase	Advanced GBC stage IIIA and IVB	Intracellular	-	Yes	No	Yes
TFG	PREDICTED: protein TFG isoform X2	Advanced GBC stage IIIA and IVB	Intracellular	Yes	Yes	No	No
THBS4	Thrombospondin-4 isoform b	Advanced GBC stage IIIA and IVB	Intracellular, secreted	Yes	Yes	No	No
UGP2	UTP--glucose-1-phosphate uridylyltransferase isoform a	Advanced GBC stage IIIA and IVB	Intracellular	-	Yes	Yes- Tissue	Yes
YWHAZ	PREDICTED: 14-3-3 protein zeta/delta isoform X1	Advanced GBC stage IIIA and IVB	Intracellular	-	Yes	No	No
ADH1B	Alcohol dehydrogenase 1B isoform 2	Early stage and Advanced stage IIIA	Intracellular	-	Yes	No	No
APOB	Apolipoprotein B-100	Early stage and Advanced stage IIIA	secreted	-	Yes	Yes- Serun	No
APOC2	Apolipoprotein C-II	Early stage and Advanced stage IIIA	secreted	-	Yes	No	No
ITGB3	Integrin beta-3	Early stage and Advanced stage IIIA	Intracellular, membrane	Yes	Yes	No	No
LPA	Apolipoprotein(a)	Early stage and Advanced stage IVB	Intracellular, secreted	-	Yes	No	No
PF4	Platelet factor 4	Early stage and Advanced stage IVB	secreted	Yes	Yes	No	No
VWF	Von Willebrand factor	Early stage and Advanced stage IVB	secreted	Yes	Yes	Yes- Tissue	No

Supplementary Table S4: List of plasma-derived EV proteins with altered levels in early stage GBC. A total of 42 EV proteins (≥2.0 fold change) were identified as detailed in Methods section. The Table describes the total number of proteins and peptides identified in

Protein Accession	Gene ID	Gene Symbol	Description	Unique Peptides	PSMs	Differential	ΣCoverage	Fold change GBC vs Healthy 117/114	p value (117/114)	Fold change GBC vs GSD 117/115	p value 117/115	Fold change GBC vs XGC 117/116	p value 117/116	Fold change XGC vs healthy 116/114	Fold change XGC vs GSD 116/115	GBC vs Healthy 117/114 Variability [%]	GBC vs GSD 117/115 Variability [%]	GBC vs XGC 117/116 Variability [%]	XGC vs healthy 116/114 Variability [%]	XGC vs GSD 116/115 Variability [%]	Sequence	Modifications	Quan Info	114	115	116	117	q-Value	PEP	XCorr	Ion Score	Exp Value	# Missed Cleavages	Charge	m/z [Da]	RT [min]	Spectrum File
4505733	5196	PF4	platelet factor 4	2	26	GBC vs Healthy, XGC	23.76	2.952	1.0E-12	0.861	2.1E-02	0.289	2.6E-31	9.672	3.122	44.7	40.2	35.1	69.0	18.1																	
4505733																					hITSLEVIk	N-Term(iTRAQ4plex); K9(iTRAQ4plex)	Unique	1.301E5	4.127E5	2.318E6	5.774E5	0	0.0176	3.16			0	3	443.27792	32.63	NIOP_Eset1_031019_Frac6.raw
4505733																					hITSLEVIk	N-Term(iTRAQ4plex); K9(iTRAQ4plex)	Unique	1.202E5	2.647E5	1.367E6	5.316E5	0	0.02181	3.27			0	3	443.27768	32.55	NIOP_Eset1_031019_Frac6.raw
4505733																					hITSLEVIk	N-Term(iTRAQ4plex); K9(iTRAQ4plex)	Unique	1.073E5	3.295E5	1.382E6	5.268E5	0	0.03299	3.13			0	3	443.27768	32.55	NIOP_Eset1_031019_Frac6.raw
4505733																					hITSLEVIk	N-Term(iTRAQ4plex); K9(iTRAQ4plex)	Unique	9.383E4	1.210E5	6.631E5	3.747E5	0.001	0.04915	2.79			0	3	443.27786	32.46	NIOP_Eset1_031019_Frac6.raw
4505733																					hITSLEVIk	N-Term(iTRAQ4plex); K9(iTRAQ4plex)	Unique	5.940E4	2.979E5	1.228E6	2.887E5	0.001	0.05576	3.13			0	3	443.27725	40.24	EV_set1_060319_050219_Frac3.raw
4505733																					hITSLEVIk	N-Term(iTRAQ4plex); K9(iTRAQ4plex)	Unique	8.069E4	8.088E4	4.692E5	2.669E5	0.001	0.05164	2.58			0	3	443.27780	32.45	NIOP_Eset1_031019_Frac6.raw
4505733																					hITSLEVIk	N-Term(iTRAQ4plex); K9(iTRAQ4plex)	Unique	6.130E4	1.800E5	1.129E6	2.539E5	0	0.00594	3.26			0	3	443.27744	32.80	NIOP_Eset1_031019_Frac6.raw
4505733																					hITSLEVIk	N-Term(iTRAQ4plex); K9(iTRAQ4plex)	Unique	1.060E5	1.363E5	4.492E5	1.393E5	0.002	0.08657	2.86			0	3	443.27725	40.25	EV_set1_060319_050219_Frac3.raw
4505733																					hITSLEVIk	N-Term(iTRAQ4plex); K9(iTRAQ4plex)	Unique	4.894E4	1.312E5	5.136E5	1.151E5	0	0.00219	3.70			0	2	664.41290	40.25	EV_set1_060319_050219_Frac3.raw
4505733																					hITSLEVIk	N-Term(iTRAQ4plex); K9(iTRAQ4plex)	Unique	3.483E4	6.923E4	2.084E5	8.752E4	0.001	0.05958	3.24			0	2	664.41400	40.26	EV_set1_060319_050219_Frac3.raw
4505733																					hITSLEVIk	N-Term(iTRAQ4plex); K9(iTRAQ4plex)	Unique	1.433E4	9.944E4	4.721E5	8.471E4	0	0.00408	3.68			0	2	664.41278	32.54	NIOP_Eset1_031019_Frac6.raw
4505733																					hITSLEVIk	N-Term(iTRAQ4plex); K9(iTRAQ4plex)	Unique	1.025E4	1.212E5	5.782E5	8.022E4	0	0.00525	3.60			0	2	664.41266	32.71	NIOP_Eset1_031019_Frac6.raw
4505733																					hITSLEVIk	N-Term(iTRAQ4plex); K9(iTRAQ4plex)	Unique	1.766E4	2.752E4	1.403E5	4.754E4	0.001	0.0449	2.44			0	3	443.27753	32.98	NIOP_Eset1_031019_Frac6.raw
4505733																					hITSLEVIk	N-Term(iTRAQ4plex); K9(iTRAQ4plex)	Unique	6.773E3	4.839E4	2.259E5	4.691E4	0	0.0112	3.22			0	3	443.27780	39.07	EV_set1_060319_050219_Frac4.raw
4505733																					hITSLEVIk	N-Term(iTRAQ4plex); K9(iTRAQ4plex)	Unique	7.903E3	5.613E4	2.010E5	4.303E4	0	0.01681	3.29			0	2	664.41351	32.89	NIOP_Eset1_031019_Frac6.raw
4505733																					hITSLEVIk	N-Term(iTRAQ4plex); K9(iTRAQ4plex)	Unique	7.839E3	3.987E4	2.136E5	3.337E4	0	0.02099	2.62			0	3	443.27774	39.07	EV_set1_060319_050219_Frac5.raw
4505733																					hITSLEVIk	N-Term(iTRAQ4plex); K9(iTRAQ4plex)	Unique	8.199E3	2.786E4	1.277E5	2.947E4	0	0.0078	3.20			0	2	664.41315	39.08	EV_set1_060319_050219_Frac4.raw
4505733																					hITSLEVIk	N-Term(iTRAQ4plex); K9(iTRAQ4plex)	Unique	1.043E4	2.096E4	9.446E4	2.889E4	0.001	0.04648	2.45			0	3	443.27780	39.07	EV_set1_060319_050219_Frac4.raw
4505733																					hITSLEVIk	N-Term(iTRAQ4plex); K9(iTRAQ4plex)	Unique	8.236E3	2.929E4	7.149E4	2.223E4	0	0.02032	2.63			0	2	664.41260	32.36	NIOP_Eset1_031019_Frac6.raw
4505733																					hITSLEVIk	N-Term(iTRAQ4plex); K9(iTRAQ4plex)	Unique	8.318E3	2.109E4	1.030E5	2.205E4	0	0.02188	2.84			0	3	443.27774	39.08	EV_set1_060319_050219_Frac5.raw
4505733																					hITSLEVIk	N-Term(iTRAQ4plex); K9(iTRAQ4plex)	Unique	5.731E3	1.691E4	7.220E4	2.061E4	0.001	0.05713	3.01			0	2	664.41290	39.11	EV_set1_060319_050219_Frac5.raw
4505733																					hITSLEVIk	N-Term(iTRAQ4plex); K9(iTRAQ4plex)	Unique	6.264E3	2.680E4	1.155E5	1.920E4	0	0.00812	3.39			0	2	664.41290	39.10	EV_set1_060319_050219_Frac5.raw
4505733																					hITSLEVIk	N-Term(iTRAQ4plex); K9(iTRAQ4plex)	Unique	1.249E4	1.496E4	5.557E4	1.889E4	0.005	0.1398	1.93			0	3	443.27774	39.08	EV_set1_060319_050219_Frac5.raw
4505733																					hITSLEVIk	N-Term(iTRAQ4plex); K9(iTRAQ4plex)	Unique	4.764E3	1.398E4	5.858E4	1.666E4	0.001	0.05637	2.60			0	2	664.41315	39.08	EV_set1_060319_050219_Frac4.raw
4505733																					hITSLEVIk	N-Term(iTRAQ4plex); K9(iTRAQ4plex)	Unique	3.650E3	9.885E3	4.645E4	1.490E4	0.006	0.1551	2.12			0	2	664.41290	39.14	EV_set1_060319_050219_Frac5.raw
4505733																					aGPHcPTAQLIATLk	N-Term(iTRAQ4plex); C5(Methylthio); K15(iTRAQ4plex)	Unique	2.475E3	5.217E3	1.532E4	3.034E3	0	0.00568	3.15			0	3	619.00989	76.86	NIOP_Eset1_031019_Frac6.raw
4557323	345	APOC3	apolipoprotein C-III	2	6	GBC vs Healthy	27.27	0.466	4.1E-03	0.662	8.9E-02	0.831	2.8E-02	0.648	0.730	17.8	21.1	32.6	17.7	25.3																	
4557323																					gWVTDGFSSLk	N-Term(iTRAQ4plex); K11(iTRAQ4plex)	Unique	2.403E4	1.988E4	2.061E4	1.577E4	0	0.00169	3.40			0	2	742.90399	63.88	EV_set1_060319_050219_Frac3.raw
4557323																					gWVTDGFSSLk	N-Term(iTRAQ4plex); K11(iTRAQ4plex)	Unique	1.994E4	1.138E4	1.138E4	1.132E4	0	0.02362	2.91			0	3	495.60425	63.87	EV_set1_060319_050219_Frac3.raw
4557323																					gWVTDGFSSLk	N-Term(iTRAQ4plex); K11(iTRAQ4plex)	Unique	2.195E4	1.281E4	1.792E4	1.106E4	0	0.02918	2.78			0	2	742.90436	63.91	EV_set1_060319_050219_Frac3.raw
4557323																					gWVTDGFSSLk	N-Term(iTRAQ4plex); K11(iTRAQ4plex)	Unique	1.815E4	1.630E4	1.775E4	1.021E4	0	0.02443	3.03			0	3	495.60425	63.87	EV_set1_060319_050219_Frac3.raw
4557323																					gWVTDGFSSLk	N-Term(iTRAQ4plex); K11(iTRAQ4plex)	Unique	1.048E4	6.955E3	1.127E4	8.744E3	0.001	0.05816	2.08			0	3	495.60464	63.93	EV_set1_060319_050219_Frac3.raw
4557323																					dALSSVQESQVAQQAR	N-Term(iTRAQ4plex)	Unique	1.703E3	2.004E3	1.315E3		0	0.00595	2.59			0	3	620.98871	40.36	EV_set1_060319_050219_Frac1.raw
4757826	567	B2M	beta-2-microglobulin	1	4	GBC vs XGC	8.40	1.213	8.4E-02	1.168	1.2E-01	0.478	7.4E-03	3.217	2.427	10.8	18.5	24.9	60.8	26.2																	
4757826																					vNHVTLSQPk	N-Term(iTRAQ4plex); K10(iTRAQ4plex)	Unique	1.825E4	1.531E4	5.250E4	2.743E4	0	0.01464	2.79			0	3	470.94827	15.25	NIOP_Eset1_031019_Frac6.raw
4757826																					vNHVTLSQPk	N-Term(iTRAQ4plex); K10(iTRAQ4plex)	Unique	3.677E3	6.061E3	2.206E4	8.284E3	0.004	0.1178	1.50			0	3	470.94809	22.11	EV_set1_060319_050219_Frac4.raw
4757826																					vNHVTLSQPk	N-Term(iTRAQ4plex); K10(iTRAQ4plex)	Unique	3.772E3	4.131E3	1.062E4	5.477E3	0.005	0.1387	1.36			0	3	470.94855	22.12	EV_set1_060319_050219_Frac4.raw

4757826																					vNHVTLSQPk	N-Term(iTRAQ4plex); K10(iTRAQ4plex)	Unique	1.414E3	1.081E3	1.153E4	1.837E3	0.006	0.1544	1.42			0	3	470.94846	22.27	EV_set1_060319_050219_Frac5.raw
5729877	3312	HSPA8	heat shock cognate 71 kDa protein isoform 1	2	2	GBC vs XGC	2.79	0.564	2.7E-01	0.554	6.2E-05	0.399	8.0E-02	1.447	1.350	21.8	0.1	12.3	34.7	12.2																	
5729877																					vcNPIITk	N-Term(iTRAQ4plex); C2(Methylthio); K8(iTRAQ4plex)	Unique	1.475E4	1.623E4	3.461E4	1.172E4	0.001	0.06715	2.02			0	2	611.35004	50.57	EV_set1_060319_050219_Frac4.raw
5729877																					mVNHFIAEFk	N-Term(iTRAQ4plex); M1(Oxidation); K10(iTRAQ4plex)	Unique	1.065E4	8.759E3	1.585E4	6.330E3	0.001	0.04838	1.96			0	3	513.94623	47.61	EV_set1_060319_050219_Frac5.raw
6005942	7415	VCP	transitional endoplasmic reticulum ATPase	5	24	GBC vs XGC	6.58	1.327	5.1E-04	0.780	1.1E-01	0.299	1.3E-18	4.609	3.211	50.5	70.1	81.3	59.8	27.5																	
6005942																					aIGVkPPR	N-Term(iTRAQ4plex); K5(iTRAQ4plex)	Unique	1.686E5	1.030E5	4.012E5	1.902E5	0.008	0.1766	2.51			0	3	375.91635	13.33	NIOP_Eset1_031019_Frac6.raw
6005942																					kYEMFAQTLQQSR	N-Term(iTRAQ4plex); K1(iTRAQ4plex)	Unique	6.229E4	3.641E4	7.479E4	3.704E4	0	0.01999	3.55			1	3	640.00409	45.58	NIOP_Eset1_031019_Frac6.raw
6005942																					kYEmFAQTLQQSR	N-Term(iTRAQ4plex); K1(iTRAQ4plex); M4(Oxidation)	Unique	1.046E4	1.310E4	4.186E4	2.382E4	0	0.01101	2.99			1	3	645.33978	36.82	NIOP_Eset1_031019_Frac6.raw
6005942																					gILLYGPPGTGk	N-Term(iTRAQ4plex); K12(iTRAQ4plex)	Unique	8.724E3	1.014E4	3.041E4	1.402E4	0	0.00433	3.29			0	2	730.93909	56.52	EV_set1_060319_050219_Frac3.raw
6005942																					kGDIFLVR	N-Term(iTRAQ4plex); K1(iTRAQ4plex)	Unique	4.572E3	5.143E3	3.325E4	1.195E4	0	0.02547	2.64			1	3	412.59482	34.22	NIOP_Eset1_031019_Frac6.raw
6005942																					kYEmFAQTLQQSR	N-Term(iTRAQ4plex); K1(iTRAQ4plex); M4(Oxidation)	Unique	7.046E3	7.221E3	3.377E4	9.260E3	0	6.5E-05	4.88			1	3	645.33966	41.14	EV_set1_060319_050219_Frac5.raw
6005942																					kYEmFAQTLQQSR	N-Term(iTRAQ4plex); K1(iTRAQ4plex); M4(Oxidation)	Unique	5.573E3	6.297E3	2.825E4	9.055E3	0	0.00043	3.91			1	3	645.33966	41.15	EV_set1_060319_050219_Frac5.raw
6005942																					gILLYGPPGTGk	N-Term(iTRAQ4plex); K12(iTRAQ4plex)	Unique	2.162E3	5.748E3	2.183E4	4.717E3	0	0.00257	3.20			0	3	487.62860	55.94	EV_set1_060319_050219_Frac5.raw
6005942																					gILLYGPPGTGk	N-Term(iTRAQ4plex); K12(iTRAQ4plex)	Unique	2.356E3	1.946E3	8.568E3	4.370E3	0	0.0109	2.52			0	3	487.62842	55.97	EV_set1_060319_050219_Frac5.raw
6005942																					gILLYGPPGTGk	N-Term(iTRAQ4plex); K12(iTRAQ4plex)	Unique	2.248E3	2.697E3	1.353E4	4.352E3	0	0.00913	2.56			0	3	487.62860	55.95	EV_set1_060319_050219_Frac5.raw
6005942																					gILLYGPPGTGk	N-Term(iTRAQ4plex); K12(iTRAQ4plex)	Unique	3.130E3	1.878E3	1.201E4	3.309E3	0.002	0.07816	2.40			0	2	730.93890	56.05	EV_set1_060319_050219_Frac5.raw
6005942																					gVLFYGPPGcGk	N-Term(iTRAQ4plex); C10(Methylthio); K12(iTRAQ4plex)	Unique	1.186E3	3.487E3	1.628E4	2.743E3	0	0.00401	2.60			0	3	510.26828	66.20	EV_set1_060319_050219_Frac5.raw
6005942																					gILLYGPPGTGk	N-Term(iTRAQ4plex); K12(iTRAQ4plex)	Unique	1.640E3	2.725E3	1.383E4	2.737E3	0	0.00618	2.62			0	2	730.93884	56.08	EV_set1_060319_050219_Frac4.raw
6005942																					gVLFYGPPGcGk	N-Term(iTRAQ4plex); C10(Methylthio); K12(iTRAQ4plex)	Unique	1.145E3	1.167E3	6.738E3	2.228E3	0.001	0.04156	1.99			0	3	510.26816	66.26	EV_set1_060319_050219_Frac5.raw
6005942																					gVLFYGPPGcGk	N-Term(iTRAQ4plex); C10(Methylthio); K12(iTRAQ4plex)	Unique	1.131E3	1.545E3	7.221E3	1.510E3	0	0.01936	1.96			0	3	510.26810	66.37	EV_set1_060319_050219_Frac4.raw
6005942																					gVLFYGPPGcGk	N-Term(iTRAQ4plex); C10(Methylthio); K12(iTRAQ4plex)	Unique	1.888E3	3.394E3	1.136E4	1.260E3	0	0.00176	2.55			0	2	764.89838	66.23	EV_set1_060319_050219_Frac5.raw
6005942																					gVLFYGPPGcGk	N-Term(iTRAQ4plex); C10(Methylthio); K12(iTRAQ4plex)	Unique	1.191E3	1.683E3	1.104E4	1.182E3	0	0.0217	2.27			0	3	510.26825	66.24	EV_set1_060319_050219_Frac5.raw
6005942																					gVLFYGPPGcGk	N-Term(iTRAQ4plex); C10(Methylthio); K12(iTRAQ4plex)	Unique	1.183E3	1.503E3	7.943E3	1.141E3	0	0.01275	2.07			0	3	510.26819	66.35	EV_set1_060319_050219_Frac4.raw
6005942																					gVLFYGPPGcGk	N-Term(iTRAQ4plex); C10(Methylthio); K12(iTRAQ4plex)	Unique		2.368E3	1.163E4	1.162E3	0	0.00669	2.57			0	3	510.26828	66.21	EV_set1_060319_050219_Frac5.raw
6005942																					gVLFYGPPGcGk	N-Term(iTRAQ4plex); C10(Methylthio); K12(iTRAQ4plex)	Unique		2.029E3	1.085E4	3.162E3	0	0.02206	2.50			0	2	764.89838	66.24	EV_set1_060319_050219_Frac5.raw
6005942																					gVLFYGPPGcGk	N-Term(iTRAQ4plex); C10(Methylthio); K12(iTRAQ4plex)	Unique		1.735E3	1.066E4	1.219E3	0	0.00336	2.41			0	2	764.89764	66.42	EV_set1_060319_050219_Frac4.raw
6005942																					gVLFYGPPGcGk	N-Term(iTRAQ4plex); C10(Methylthio); K12(iTRAQ4plex)	Unique		1.937E3	4.132E3	1.276E3	0	0.00847	2.26			0	3	510.26804	66.41	EV_set1_060319_050219_Frac4.raw
6005942																					gVLFYGPPGcGk	N-Term(iTRAQ4plex); C10(Methylthio); K12(iTRAQ4plex)	Unique		2.273E3	1.000E4	1.423E3	0	0.0355	2.24			0	2	764.89801	66.29	EV_set1_060319_050219_Frac5.raw
6005942																					gVLFYGPPGcGk	N-Term(iTRAQ4plex); C10(Methylthio); K12(iTRAQ4plex)	Unique			6.856E3	1.177E3	0	0.0343	2.05			0	2	764.89764	66.44	EV_set1_060319_050219_Frac4.raw
8393638	50848	F11R	junctional adhesion molecule A	1	2	GBC vs XGC	3.68	1.026	5.3E-01	0.621	8.5E-02	0.279	3.6E-02	3.769	2.166	36.2	4.4	6.7	43.7	2.3																	
8393638																					vTFLPTGITFk	N-Term(iTRAQ4plex); K11(iTRAQ4plex)	Unique	1.932E3	3.894E3	1.247E4	3.060E3	0	0.02509	1.93			0	3	504.64075	77.28	EV_set1_060319_050219_Frac3.raw
8393638																					vTFLPTGITFk	N-Term(iTRAQ4plex); K11(iTRAQ4plex)	Unique	2.974E3	3.518E3	1.092E4	2.934E3	0.001	0.0424	1.82			0	3	504.64063	77.31	EV_set1_060319_050219_Frac3.raw
19743813	3688	ITGB1	integrin beta-1 isoform 1A	1	4	GBC vs XGC	2.01	1.254	6.9E-03	1.292	2.3E-04	0.409	1.5E-05	3.210	3.149	18.8	22.9	9.7	24.4	12.9																	
19743813																					dkLPQPVQPDPVSHck	N-Term(iTRAQ4plex); K2(iTRAQ4plex); C15(Methylthio); K16(iTRAQ4plex)	Unique	5.273E4	6.098E4	2.750E5	8.933E4	0	0.00063	4.93			1	4	567.30621	36.31	NIOP_Eset1_031019_Frac6.raw

19743813																					dkLPQPVQPDPVSHck	N-Term(iTRAQ4plex);<br>K2(iTRAQ4plex);<br>C15(Methylthio);<br>K16(iTRAQ4plex)	Unique	4.322E4	3.576E4	1.971E5	7.644E4	0	0.00037	5.59			1	4	567.30713	36.34	NIOP_Eset1_031019_Frac6.raw
19743813																					dkLPQPVQPDPVSHck	N-Term(iTRAQ4plex);<br>K2(iTRAQ4plex);<br>C15(Methylthio);<br>K16(iTRAQ4plex)	Unique	3.256E4	3.133E4	1.227E5	4.478E4	0	0.00368	3.78			1	4	567.30646	36.14	NIOP_Eset1_031019_Frac6.raw
19743813																					dkLPQPVQPDPVSHck	N-Term(iTRAQ4plex);<br>K2(iTRAQ4plex);<br>C15(Methylthio);<br>K16(iTRAQ4plex)	Unique	3.168E4	2.051E4	9.553E4	3.974E4	0	0.00249	3.71			1	4	567.30646	36.15	NIOP_Eset1_031019_Frac6.raw
19913375	80739	C6orf25	protein G6b isoform G6b-B	3	4	GBC vs XGC	11.62	1.216	5.5E-01	0.592	4.1E-01	0.113	6.9E-03	7.827	4.171	37.7	53.8	16.0	123.8	110.9																	
19913375																					fAPLVk	N-Term(iTRAQ4plex);<br>K6(iTRAQ4plex)	Unique	4.401E3	7.127E3	6.643E4	7.223E3	0.004	0.07268		40	0.00233	0	2	481.81732	44.32	EV_set1_060319_050219_Frac4.raw
19913375																					vNLScGGVSHPIR	N-Term(iTRAQ4plex);<br>C5(Methylthio)	Unique	4.224E3	9.585E3	1.333E4	5.639E3	0.004	0.1304	2.10			0	3	510.26550	44.08	EV_set1_060319_050219_Frac4.raw
19913375																					tVLHVLGDR	N-Term(iTRAQ4plex)	Unique	2.136E3	4.011E3	5.308E4	4.660E3	0	0.02965	2.77			0	3	385.23245	39.31	EV_set1_060319_050219_Frac5.raw
19913375																					tVLHVLGDR	N-Term(iTRAQ4plex)	Unique	1.616E3	2.766E3	1.094E4	1.079E3	0.002	0.0909	1.87			0	3	385.23135	39.39	EV_set1_060319_050219_Frac5.raw
22538814	6352	CCL5	C-C motif chemokine 5 isoform 1	1	4	GBC vs Healthy, XGC	12.09	0.210	2.4E-05	1.263	5.1E-02	0.165	3.9E-06	1.231	7.469	19.0	7.5	5.1	10.2	32.4																	
22538814																					cSNPAVVFVTR	N-Term(iTRAQ4plex);<br>C1(Methylthio)	Unique	4.568E4	1.278E4	7.480E4	1.935E4	0	0.00391	3.15			0	2	691.85571	62.72	NIOP_Eset1_031019_Frac2.raw
22538814																					cSNPAVVFVTR	N-Term(iTRAQ4plex);<br>C1(Methylthio)	Unique	4.765E4	5.606E3	8.976E4	1.298E4	0	0.00237	3.26			0	2	691.85614	62.19	NIOP_Eset1_031019_Frac2.raw
22538814																					cSNPAVVFVTR	N-Term(iTRAQ4plex);<br>C1(Methylthio)	Unique	4.919E4	7.269E3	7.597E4	1.178E4	0	0.00212	3.41			0	2	691.85596	62.54	NIOP_Eset1_031019_Frac2.raw
22538814																					cSNPAVVFVTR	N-Term(iTRAQ4plex);<br>C1(Methylthio)	Unique	5.204E4	6.564E3	7.424E4	1.100E4	0	0.00024	3.93			0	2	691.85547	62.37	NIOP_Eset1_031019_Frac2.raw
32130518	344	APOC2	apolipoprotein C-II	2	6	GBC vs Healthy	17.82	0.467	9.9E-07	0.610	1.1E-01	0.902	2.0E-03	0.608	0.705	17.5	23.0	3.9	10.1	27.9																	
32130518																					tYLPAVDEk	N-Term(iTRAQ4plex);<br>K9(iTRAQ4plex)	Unique	7.649E4	6.944E4	6.041E4	4.914E4	0	0.01981	3.30			0	2	662.37231	43.12	EV_set1_060319_050219_Frac3.raw
32130518																					tYLPAVDEk	N-Term(iTRAQ4plex);<br>K9(iTRAQ4plex)	Unique	7.956E4	6.053E4	5.063E4	4.295E4	0	0.02458	3.13			0	2	662.37445	43.21	EV_set1_060319_050219_Frac3.raw
32130518																					tAAQNLYEk	N-Term(iTRAQ4plex);<br>K9(iTRAQ4plex)	Unique	2.928E4	2.239E4	2.349E4	1.997E4	0.002	0.07592	2.32			0	2	663.36993	31.72	EV_set1_060319_050219_Frac3.raw
32130518																					tAAQNLYEk	N-Term(iTRAQ4plex);<br>K9(iTRAQ4plex)	Unique	3.924E4	3.051E4	3.062E4	1.947E4	0	0.00091	3.27			0	2	663.36877	31.71	EV_set1_060319_050219_Frac3.raw
32130518																					tyLPAVDEk	N-Term(iTRAQ4plex);<br>Y2(iTRAQ4plex);<br>K9(iTRAQ4plex)	Unique	1.781E4	7.875E3	1.247E4	1.067E4	0	0.00784		31	0.08467	0	3	489.95337	33.92	EV_set1_060319_050219_Frac5.raw
32130518																					tyLPAVDEk	N-Term(iTRAQ4plex);<br>Y2(iTRAQ4plex);<br>K9(iTRAQ4plex)	Unique	1.252E4	6.610E3	1.105E4	6.357E3	0.008	0.1228		14	4.23373	0	3	489.95337	33.93	EV_set1_060319_050219_Frac5.raw
47078292	3690	ITGB3	integrin beta-3	3	11	GBC vs XGC	3.93	1.100	3.7E-01	0.664	1.1E-01	0.184	3.0E-09	5.192	3.452	23.9	26.2	75.7	82.4	46.5																	
47078292																					sFTIkPVGFk	N-Term(iTRAQ4plex);<br>K5(iTRAQ4plex);<br>K10(iTRAQ4plex)	Unique	3.981E4	8.410E4	5.696E5	6.122E4	0	0.00372	3.59			0	3	519.32416	44.08	NIOP_Eset1_031019_Frac6.raw
47078292																					sFTIkPVGFk	N-Term(iTRAQ4plex);<br>K5(iTRAQ4plex);<br>K10(iTRAQ4plex)	Unique	3.963E4	6.820E4	4.125E5	3.474E4	0	0.00226	3.99			0	3	519.32404	44.26	NIOP_Eset1_031019_Frac6.raw
47078292																					sFTIkPVGFk	N-Term(iTRAQ4plex);<br>K5(iTRAQ4plex);<br>K10(iTRAQ4plex)	Unique	1.440E4	2.123E4	9.653E4	1.928E4	0.004	0.1197	1.99			0	3	519.32501	43.87	NIOP_Eset1_031019_Frac6.raw
47078292																					sFTIkPVGFk	N-Term(iTRAQ4plex);<br>K5(iTRAQ4plex);<br>K10(iTRAQ4plex)	Unique	1.205E4	1.422E4	7.142E4	1.614E4	0.003	0.1105	1.96			0	3	519.32422	43.90	NIOP_Eset1_031019_Frac6.raw
47078292																					tTcLPmFGYk	N-Term(iTRAQ4plex);<br>C3(Methylthio);<br>M6(Oxidation);<br>K10(iTRAQ4plex)	Unique	1.470E4	1.410E4	3.678E4	1.458E4	0	0.02869	2.20			0	3	504.24924	61.95	EV_set1_060319_050219_Frac3.raw
47078292																					hVLTLTDQVTR	N-Term(iTRAQ4plex)	Unique	1.091E4	1.260E4	3.329E4	1.154E4	0	0.01347	2.76			0	3	476.27579	37.86	EV_set1_060319_050219_Frac4.raw
47078292																					hVLTLTDQVTR	N-Term(iTRAQ4plex)	Unique	9.594E3	1.382E4	3.222E4	1.096E4	0	0.02874	2.41			0	3	476.27621	37.90	EV_set1_060319_050219_Frac4.raw
47078292																					tTcLPMFGYk	N-Term(iTRAQ4plex);<br>C3(Methylthio);<br>K10(iTRAQ4plex)	Unique	1.824E3	5.047E3	3.562E4	4.691E3	0	0.0156	2.05			0	3	498.91812	71.73	EV_set1_060319_050219_Frac3.raw
47078292																					tTcLPMFGYk	N-Term(iTRAQ4plex);<br>C3(Methylthio);<br>K10(iTRAQ4plex)	Unique	2.938E3	7.097E3	2.582E4	4.369E3	0.001	0.06145	2.01			0	3	498.91812	71.73	EV_set1_060319_050219_Frac3.raw
47078292																					tTcLPMFGYk	N-Term(iTRAQ4plex);<br>C3(Methylthio);<br>K10(iTRAQ4plex)	Unique	2.741E3	4.899E3	2.987E4	4.244E3	0	0.01046	2.30			0	2	747.87396	71.74	EV_set1_060319_050219_Frac3.raw
47078292																					tTcLPMFGYk	N-Term(iTRAQ4plex);<br>C3(Methylthio);<br>K10(iTRAQ4plex)	Unique	2.393E3	2.758E3	1.436E4	1.230E3	0.008	0.1829	1.30			0	3	498.91812	71.74	EV_set1_060319_050219_Frac3.raw
47132549	2335	FN1	fibronectin isoform 6	36	289	GBC vs GSD	24.22	0.928	2.1E-19	2.397	1.8E-218	0.944	1.5E-32	1.001	2.465	21.6	51.3	15.6	20.7	55.1																	
47132549																					vFAVSHGR	N-Term(iTRAQ4plex)	Unique	7.128E5	1.689E5	8.460E5	7.293E5	0.004	0.1299	2.67			0	2	508.79181	13.67	NIOP_Eset1_031019_Frac6.raw
47132549																					iTGYIIk	N-Term(iTRAQ4plex);<br>K7(iTRAQ4plex)	Unique	4.868E5	1.481E5	9.425E5	6.901E5	0.001	0.06424	2.95			0	2	548.35492	49.96	EV_set1_060319_050219_Frac3.raw
47132549																					yEkPGSPPR	N-Term(iTRAQ4plex);<br>K3(iTRAQ4plex)	Unique	3.135E5	8.576E4	3.904E5	3.262E5	0.001	0.06523	2.85			0	3	440.25082	11.29	NIOP_Eset1_031019_Frac6.raw
47132549																					iTGYIIk	N-Term(iTRAQ4plex);<br>K7(iTRAQ4plex)	Unique	1.522E5	5.244E4	3.009E5	2.562E5	0.009	0.1862	2.19			0	3	365.90573	49.99	EV_set1_060319_050219_Frac3.raw

47132549																					iTGYIIk	N-Term(iTRAQ4plex); K7(iTRAQ4plex)	Unique	1.602E5	5.164E4	3.134E5	2.357E5	0.004	0.1273	2.16			0	3	365.90555	49.97	EV_set1_060319_050219_Frac3.raw
47132549																					vPGTSTSATLTGLTR	N-Term(iTRAQ4plex)	Unique	2.607E5	4.079E4	2.730E5	2.317E5	0	0.01004	3.60			0	2	803.44977	45.47	NIOP_Eset1_031019_Frac2.raw
47132549																					gDSPASSkPISINYR	N-Term(iTRAQ4plex); K8(iTRAQ4plex)	Unique	1.633E5	5.919E4	2.893E5	2.245E5	0	3.9E-05	6.06			0	3	627.34241	35.53	EV_set1_060319_050219_Frac3.raw
47132549																					iTGYIIk	N-Term(iTRAQ4plex); K7(iTRAQ4plex)	Unique	1.500E5	5.672E4	2.426E5	2.069E5	0.005	0.143	2.23			0	2	548.35492	49.97	EV_set1_060319_050219_Frac3.raw
47132549																					iScTIANR	N-Term(iTRAQ4plex); C3(Methylthio)	Unique	2.283E5	4.021E4	2.104E5	1.852E5	0.001	0.04333	2.87			0	2	534.27710	36.09	NIOP_Eset1_031019_Frac2.raw
47132549																					vPGTSTSATLTGLTR	N-Term(iTRAQ4plex)	Unique	2.103E5	3.807E4	2.259E5	1.769E5	0	0.01453	3.63			0	2	803.44995	45.81	NIOP_Eset1_031019_Frac2.raw
47132549																					vPGTSTSATLTGLTR	N-Term(iTRAQ4plex)	Unique	1.862E5	3.832E4	1.970E5	1.734E5	0	0.01719	3.67			0	2	803.44849	45.64	NIOP_Eset1_031019_Frac2.raw
47132549																					gDSPASSkPISINYR	N-Term(iTRAQ4plex); K8(iTRAQ4plex)	Unique	1.291E5	5.085E4	2.245E5	1.705E5	0	5.5E-06	6.31			0	3	627.34277	35.51	EV_set1_060319_050219_Frac3.raw
47132549																					iTGYIIk	N-Term(iTRAQ4plex); K7(iTRAQ4plex)	Unique	1.758E5	3.083E4	2.064E5	1.694E5	0	0.03435	2.77			0	2	548.35529	43.96	NIOP_Eset1_031019_Frac2.raw
47132549																					iScTIANR	N-Term(iTRAQ4plex); C3(Methylthio)	Unique	1.742E5	4.193E4	1.989E5	1.607E5	0	0.03811	2.70			0	2	534.27673	35.79	NIOP_Eset1_031019_Frac2.raw
47132549																					sYTITGLQPGTDYk	N-Term(iTRAQ4plex); K14(iTRAQ4plex)	Unique	1.350E5	5.323E4	1.698E5	1.491E5	0	0.0032	4.28			0	3	611.32776	51.54	EV_set1_060319_050219_Frac3.raw
47132549																					iTGYIIk	N-Term(iTRAQ4plex); K7(iTRAQ4plex)	Unique	1.233E5	2.956E4	1.596E5	1.377E5	0	0.03173	2.89			0	2	548.35498	43.79	NIOP_Eset1_031019_Frac2.raw
47132549																					sYTITGLQPGTDYk	N-Term(iTRAQ4plex); K14(iTRAQ4plex)	Unique	1.102E5	9.554E4	1.180E5	1.344E5	0	0.0214	2.84			0	3	611.32782	51.15	EV_set1_060319_050219_Frac5.raw
47132549																					gDSPASSkPISINYR	N-Term(iTRAQ4plex); K8(iTRAQ4plex)	Unique	1.101E5	3.874E4	1.602E5	1.320E5	0	0.01331	3.38			0	3	627.34241	35.53	EV_set1_060319_050219_Frac3.raw
47132549																					iScTIANR	N-Term(iTRAQ4plex); C3(Methylthio)	Unique	1.374E5	2.678E4	1.460E5	1.287E5	0	0.01478	3.01			0	2	534.27698	35.61	NIOP_Eset1_031019_Frac2.raw
47132549																					iScTIANR	N-Term(iTRAQ4plex); C3(Methylthio)	Unique	1.442E5	3.036E4	1.447E5	1.278E5	0	0.03196	2.86			0	2	534.27686	36.26	NIOP_Eset1_031019_Frac2.raw
47132549																					qYNVGPSVSk	N-Term(iTRAQ4plex); K10(iTRAQ4plex)	Unique	1.123E5	4.058E4	1.322E5	1.276E5	0	0.00545	3.38			0	3	456.25751	32.28	EV_set1_060319_050219_Frac3.raw
47132549																					gDSPASSkPISINYR	N-Term(iTRAQ4plex); K8(iTRAQ4plex)	Unique	9.316E4	6.270E4	1.377E5	1.272E5	0	0.00215	3.51			0	4	470.75848	35.60	EV_set1_060319_050219_Frac3.raw
47132549																					lGVRPSQGGEAPR	N-Term(iTRAQ4plex)	Unique	1.396E5	3.638E4	1.693E5	1.249E5	0	0.00136	4.75			0	3	489.94336	13.43	NIOP_Eset1_031019_Frac6.raw
47132549																					yEkPGSPPR	N-Term(iTRAQ4plex); K3(iTRAQ4plex)	Unique	9.889E4	2.534E4	1.192E5	1.106E5	0	0.03809	3.54			0	3	440.25067	19.52	EV_set1_060319_050219_Frac5.raw
47132549																					sYTITGLQPGTDYk	N-Term(iTRAQ4plex); K14(iTRAQ4plex)	Unique	9.022E4	3.307E4	1.195E5	1.068E5	0	0.00214	3.72			0	3	611.32776	51.54	EV_set1_060319_050219_Frac3.raw
47132549																					vPGTSTSATLTGLTR	N-Term(iTRAQ4plex)	Unique	1.112E5	1.931E4	1.200E5	9.787E4	0	0.00585	3.95			0	2	803.45001	45.30	NIOP_Eset1_031019_Frac2.raw
47132549																					vPGTSTSATLTGLTR	N-Term(iTRAQ4plex)	Unique	2.927E4	9.382E3	1.202E5	9.555E4	0.007	0.1087		26	0.31986	0	3	535.97003	45.17	NIOP_Eset1_031019_Frac2.raw
47132549																					gDSPASSkPISINYR	N-Term(iTRAQ4plex); K8(iTRAQ4plex)	Unique	7.920E4	4.426E4	9.803E4	9.418E4	0	0.00224	4.22			0	4	470.75888	35.61	EV_set1_060319_050219_Frac3.raw
47132549																					gDSPASSkPISINYR	N-Term(iTRAQ4plex); K8(iTRAQ4plex)	Unique	6.669E4	3.213E4	1.001E5	9.107E4	0	0.00541	3.79			0	3	627.34247	35.60	EV_set1_060319_050219_Frac3.raw
47132549																					qYNVGPSVSk	N-Term(iTRAQ4plex); K10(iTRAQ4plex)	Unique	7.670E4	2.103E4	1.115E5	8.805E4	0	0.00562	3.56			0	2	683.88287	32.26	EV_set1_060319_050219_Frac3.raw
47132549																					eSkPLTAQQTTk	N-Term(iTRAQ4plex); K3(iTRAQ4plex); K12(iTRAQ4plex)	Unique	7.937E4	1.645E4	1.016E5	8.789E4	0	5.6E-05	5.99			0	3	588.68024	20.06	EV_set1_060319_050219_Frac3.raw
47132549																					iTGYIIk	N-Term(iTRAQ4plex); K7(iTRAQ4plex)	Unique	6.899E4	2.050E4	9.297E4	8.621E4	0	0.02885	2.80			0	2	548.35492	44.03	NIOP_Eset1_031019_Frac6.raw
47132549																					eATIPGHLNSYTIk	N-Term(iTRAQ4plex); K14(iTRAQ4plex)	Unique	6.394E4	3.901E4	8.170E4	8.062E4	0	0.00072	4.52			0	3	611.34393	43.51	EV_set1_060319_050219_Frac3.raw
47132549																					wSRPQAPITGYR	N-Term(iTRAQ4plex)	Unique	5.722E4	3.677E4	1.060E5	7.999E4	0	0.00119	4.45			0	3	525.95490	34.15	NIOP_Eset1_031019_Frac6.raw
47132549																					iScTIANR	N-Term(iTRAQ4plex); C3(Methylthio)	Unique	9.339E4	1.806E4	1.096E5	7.996E4	0.006	0.1533	2.45			0	2	534.27667	35.44	NIOP_Eset1_031019_Frac2.raw
47132549																					vFAVSHGR	N-Term(iTRAQ4plex)	Unique	8.192E4	2.196E4	8.490E4	7.975E4	0	0.03195	2.65			0	2	508.79175	13.85	NIOP_Eset1_031019_Frac6.raw
47132549																					wSRPQAPITGYR	N-Term(iTRAQ4plex)	Unique	5.968E4	4.441E4	8.989E4	7.701E4	0	0.03761	2.84			0	3	525.95538	33.97	NIOP_Eset1_031019_Frac6.raw
47132549																					iTGYIIk	N-Term(iTRAQ4plex); K7(iTRAQ4plex)	Unique	4.824E4	1.264E4	7.541E4	7.417E4	0.001	0.05198	2.73			0	2	548.35455	43.84	NIOP_Eset1_031019_Frac6.raw
47132549																					qYNVGPSVSk	N-Term(iTRAQ4plex); K10(iTRAQ4plex)	Unique	6.727E4	3.102E4	8.110E4	7.355E4	0	0.00834	3.37			0	3	456.25751	32.28	EV_set1_060319_050219_Frac3.raw
47132549																					sYTITGLQPGTDYk	N-Term(iTRAQ4plex); K14(iTRAQ4plex)	Unique	5.520E4	2.589E4	7.721E4	7.261E4	0	0.00376	4.40			0	3	611.32776	51.54	EV_set1_060319_050219_Frac3.raw
47132549																					iTGYIIk	N-Term(iTRAQ4plex); K7(iTRAQ4plex)	Unique	6.721E4	3.052E4	7.733E4	7.092E4	0.002	0.08957	2.80			0	2	548.35480	50.49	EV_set1_060319_050219_Frac3.raw
47132549																					vPGTSTSATLTGLTR	N-Term(iTRAQ4plex)	Unique	8.866E4	1.179E4	9.836E4	6.852E4	0	0.00139	3.86			0	2	803.44983	45.98	NIOP_Eset1_031019_Frac2.raw
47132549																					sYTITGLQPGTDYk	N-Term(iTRAQ4plex); K14(iTRAQ4plex)	Unique	5.443E4	3.699E4	5.847E4	6.841E4	0	0.01232	3.33			0	3	611.32782	51.15	EV_set1_060319_050219_Frac5.raw
47132549																					iTGYIIk	N-Term(iTRAQ4plex); K7(iTRAQ4plex)	Unique	6.678E4	1.327E4	7.227E4	6.538E4	0.001	0.04549	2.70			0	2	548.35529	44.13	NIOP_Eset1_031019_Frac2.raw
47132549																					vPGTSTSATLTGLTR	N-Term(iTRAQ4plex)	Unique	1.196E5	5.850E4	9.374E4	6.453E4	0.001	0.05079	2.59			0	3	535.96960	46.55	NIOP_Eset1_031019_Frac2.raw
47132549																					vPGTSTSATLTGLTR	N-Term(iTRAQ4plex)	Unique	5.682E4	7.915E3	6.911E4	6.209E4	0	0.00384	3.83			0	2	803.45020	46.15	NIOP_Eset1_031019_Frac2.raw
47132549																					sYTITGLQPGTDYk	N-Term(iTRAQ4plex); K14(iTRAQ4plex)	Unique	5.517E4	8.235E3	6.439E4	5.931E4	0	5.2E-05	4.30			0	2	916.48926	51.60	EV_set1_060319_050219_Frac3.raw
47132549																					iTGYIIk	N-Term(iTRAQ4plex); K7(iTRAQ4plex)	Unique	5.838E4	1.515E4	7.618E4	5.926E4	0	0.03192	2.94			0	2	548.35522	43.61	NIOP_Eset1_031019_Frac2.raw
47132549																					qYNVGPSVSk	N-Term(iTRAQ4plex); K10(iTRAQ4plex)	Unique	5.154E4	2.966E4	5.203E4	5.909E4	0	0.02289	2.59			0	3	456.25748	32.31	EV_set1_060319_050219_Frac3.raw

47132549																					eSkPLTAQQTTk	N-Term(iTRAQ4plex); K3(iTRAQ4plex); K12(iTRAQ4plex)	Unique	5.122E4	1.298E4	6.540E4	5.895E4	0	0.00027	5.22			0	3	588.68024	20.06	EV_set1_060319_050219_Frac3.raw
47132549																					qYNVGPSVSk	N-Term(iTRAQ4plex); K10(iTRAQ4plex)	Unique	4.612E4	1.578E4	6.321E4	5.820E4	0	0.01577	3.14			0	2	683.88287	32.26	EV_set1_060319_050219_Frac3.raw
47132549																					sTTPDITGyR	N-Term(iTRAQ4plex); Y9(iTRAQ4plex)	Unique	4.448E4	2.130E4	6.159E4	5.762E4	0.006	0.09341		10	10.1128	0	3	466.92102	29.97	EV_set1_060319_050219_Frac3.raw
47132549																					dLQFVEVTDVk	N-Term(iTRAQ4plex); K11(iTRAQ4plex)	Unique	6.328E4	1.708E4	6.126E4	5.736E4	0	0.00221	3.04			0	3	527.63202	61.51	EV_set1_060319_050219_Frac3.raw
47132549																					iyLYTLNDNAR	N-Term(iTRAQ4plex); Y2(iTRAQ4plex)	Unique	5.383E4	1.876E4	6.280E4	5.664E4	0.001	0.02976		16	2.71498	0	3	548.63831	42.26	NIOP_Eset1_031019_Frac6.raw
47132549																					iyLYTLNDNAR	N-Term(iTRAQ4plex); Y2(iTRAQ4plex)	Unique	4.766E4	7.552E3	6.650E4	5.590E4	0	0.00096		27	0.21041	0	3	548.63940	42.09	NIOP_Eset1_031019_Frac6.raw
47132549																					sYTITGLQPGTDYk	N-Term(iTRAQ4plex); K14(iTRAQ4plex)	Unique	4.409E4	3.429E4	4.116E4	5.556E4	0.001	0.05459	2.77			0	3	611.32782	51.16	EV_set1_060319_050219_Frac5.raw
47132549																					eATIPGHLNSYTIk	N-Term(iTRAQ4plex); K14(iTRAQ4plex)	Unique	4.848E4	3.589E4	5.062E4	5.478E4	0	0.03091	2.95			0	3	611.34406	43.54	EV_set1_060319_050219_Frac3.raw
47132549																					eATIPGHLNSYTIk	N-Term(iTRAQ4plex); K14(iTRAQ4plex)	Unique	4.325E4	2.693E4	5.174E4	5.455E4	0	0.00272	3.81			0	3	611.34393	43.51	EV_set1_060319_050219_Frac3.raw
47132549																					syTITGLQPGTDYk	N-Term(iTRAQ4plex); Y2(iTRAQ4plex); K14(iTRAQ4plex)	Unique	4.355E4	1.687E4	5.349E4	5.368E4	0.002	0.03542		10	14.7077	0	3	659.36255	36.99	NIOP_Eset1_031019_Frac6.raw
47132549																					vPGTSTSATLTGLTR	N-Term(iTRAQ4plex)	Unique	6.442E4	7.549E3	6.582E4	5.179E4	0	0.00625	3.74			0	2	803.45020	46.32	NIOP_Eset1_031019_Frac2.raw
47132549																					iTYGETGGNSPVQEFTVPGSk	N-Term(iTRAQ4plex); K21(iTRAQ4plex)	Unique	4.464E4	9.677E3	5.766E4	5.000E4	0	1.8E-05	5.38			0	3	819.42340	60.83	EV_set1_060319_050219_Frac3.raw
47132549																					yEVSVYALk	N-Term(iTRAQ4plex); K9(iTRAQ4plex)	Unique	5.016E4	1.641E4	6.023E4	4.751E4	0	0.00133	3.62			0	2	680.39294	55.96	EV_set1_060319_050219_Frac3.raw
47132549																					wLPSSSPVTGyR	N-Term(iTRAQ4plex); Y11(iTRAQ4plex)	Unique	3.747E4	9.091E3	5.196E4	4.561E4	0.003	0.06252		27	0.28123	0	3	546.63507	41.50	NIOP_Eset1_031019_Frac6.raw
47132549																					iScTIANR	N-Term(iTRAQ4plex); C3(Methylthio)	Unique	4.269E4	1.065E4	5.306E4	4.540E4	0.002	0.08006	2.72			0	2	534.27704	46.59	EV_set1_060319_050219_Frac1.raw
47132549																					ySFcTDHTVLVQTR	N-Term(iTRAQ4plex); C4(Methylthio)	Unique	4.942E4	1.531E4	4.894E4	4.534E4	0	0.00049	4.52			0	3	620.63434	57.60	EV_set1_060319_050219_Frac3.raw
47132549																					gDSPASSkPISINYR	N-Term(iTRAQ4plex); K8(iTRAQ4plex)	Unique	2.546E4	2.359E4	4.062E4	4.460E4	0	0.02502	2.66			0	4	470.75830	34.46	EV_set1_060319_050219_Frac5.raw
47132549																					vPGTSTSATLTGLTR	N-Term(iTRAQ4plex)	Unique	4.404E4	7.126E3	4.860E4	4.446E4	0	0.03078	3.01			0	2	803.44983	45.12	NIOP_Eset1_031019_Frac2.raw
47132549																					wLPSSSPVTGYR	N-Term(iTRAQ4plex)	Unique	4.451E4	1.545E4	5.076E4	4.368E4	0.008	0.1765	1.99			0	2	747.39691	62.10	NIOP_Eset1_031019_Frac2.raw
47132549																					iYLYTLNDNAR	N-Term(iTRAQ4plex)	Unique	3.046E4	4.022E3	4.018E4	4.367E4	0	0.00428	2.79			0	2	750.40125	58.26	NIOP_Eset1_031019_Frac6.raw
47132549																					gDSPASSkPISINYR	N-Term(iTRAQ4plex); K8(iTRAQ4plex)	Unique	3.215E4	8.790E3	5.755E4	4.281E4	0	0.00101	4.17			0	3	627.34235	34.34	EV_set1_060319_050219_Frac5.raw
47132549																					cHEGGQSYk	N-Term(iTRAQ4plex); C1(Methylthio); K9(iTRAQ4plex)	Unique	7.722E3	1.263E4	4.639E4	4.247E4	0	0.01136	2.77			0	3	448.20807	10.56	NIOP_Eset1_031019_Frac6.raw
47132549																					qYNVGPSVSk	N-Term(iTRAQ4plex); K10(iTRAQ4plex)	Unique	3.460E4	1.628E4	4.240E4	4.247E4	0.001	0.04282	2.70			0	2	683.88287	32.29	EV_set1_060319_050219_Frac3.raw
47132549																					wLPSSSPVTGYR	N-Term(iTRAQ4plex)	Unique	4.322E4	1.454E4	4.680E4	4.243E4	0.003	0.05357		37	0.02313	0	2	747.39746	61.92	NIOP_Eset1_031019_Frac2.raw
47132549																					vFAVSHGR	N-Term(iTRAQ4plex)	Unique	3.350E4	1.579E4	3.735E4	4.238E4	0.001	0.06629	2.76			0	2	508.79285	28.99	EV_set1_060319_050219_Frac3.raw
47132549																					lGVRPSqGGEAPR	N-Term(iTRAQ4plex); Q7(Deamidated)	Unique	3.688E4	2.022E4	5.062E4	4.165E4	0	0.00115	4.35			0	3	490.27158	28.53	EV_set1_060319_050219_Frac3.raw
47132549																					eATIPGHLNSyTIk	N-Term(iTRAQ4plex); Y11(iTRAQ4plex); K14(iTRAQ4plex)	Unique	2.989E4	1.392E4	4.682E4	4.163E4	0	0.00238		13	4.52482	0	3	659.37732	31.97	NIOP_Eset1_031019_Frac6.raw
47132549																					sYTITGLQPGTDyk	N-Term(iTRAQ4plex); Y13(iTRAQ4plex); K14(iTRAQ4plex)	Unique	2.572E4	5.228E3	4.593E4	4.067E4	0	0.0061		14	5.91643	0	4	494.77362	40.72	EV_set1_060319_050219_Frac5.raw
47132549																					syTITGLQPGTDYk	N-Term(iTRAQ4plex); Y2(iTRAQ4plex); K14(iTRAQ4plex)	Unique	3.171E4	8.389E3	5.208E4	4.041E4	0.001	0.01172		17	3.14818	0	4	494.77362	40.71	EV_set1_060319_050219_Frac5.raw
47132549																					sYTITGLQPGTDYk	N-Term(iTRAQ4plex); K14(iTRAQ4plex)	Unique	3.237E4	1.965E4	3.462E4	4.027E4	0	0.00682	3.43			0	3	611.32776	51.54	EV_set1_060319_050219_Frac3.raw
47132549																					wLPSSSPVTGYR	N-Term(iTRAQ4plex)	Unique	3.402E4	1.730E4	4.126E4	4.017E4	0.004	0.07377		23	0.5239	0	2	747.39716	63.30	NIOP_Eset1_031019_Frac2.raw
47132549																					wLPSSSPVTGYR	N-Term(iTRAQ4plex)	Unique	3.743E4	9.299E3	4.414E4	3.999E4	0.005	0.1479	2.30			0	2	747.39642	57.15	EV_set1_060319_050219_Frac3.raw
47132549																					lGVRPSqGGEAPR	N-Term(iTRAQ4plex); Q7(Deamidated)	Unique	3.349E4	1.128E4	4.129E4	3.950E4	0.004	0.1258	2.55			0	3	490.27158	28.53	EV_set1_060319_050219_Frac3.raw
47132549																					vPGTSTSATLTGLTR	N-Term(iTRAQ4plex)	Unique	3.917E4	9.932E3	5.061E4	3.892E4	0	0.01072	3.24			0	2	803.45026	46.49	NIOP_Eset1_031019_Frac2.raw
47132549																					yEVSVYALk	N-Term(iTRAQ4plex); K9(iTRAQ4plex)	Unique	3.201E4	1.613E4	4.519E4	3.764E4	0.001	0.05752	2.27			0	3	453.93048	55.90	EV_set1_060319_050219_Frac3.raw
47132549																					iTYGETGGNSPVQEFTVPGSk	N-Term(iTRAQ4plex); K21(iTRAQ4plex)	Unique	2.307E4	7.359E3	4.702E4	3.701E4	0	0.00014	4.98			0	3	819.42340	60.82	EV_set1_060319_050219_Frac3.raw
47132549																					syTITGLQPGTDYk	N-Term(iTRAQ4plex); Y2(iTRAQ4plex); K14(iTRAQ4plex)	Unique	2.110E4	3.838E3	3.569E4	3.633E4	0	0.00629		12	8.94956	0	3	659.36243	40.83	EV_set1_060319_050219_Frac5.raw
47132549																					vGDTYERPk	N-Term(iTRAQ4plex); K9(iTRAQ4plex)	Unique	2.615E4	8.213E3	3.859E4	3.548E4	0.002	0.08339	2.03			0	3	451.58563	11.59	NIOP_Eset1_031019_Frac6.raw
47132549																					yEVSVYALk	N-Term(iTRAQ4plex); K9(iTRAQ4plex)	Unique	3.408E4	1.566E4	3.513E4	3.523E4	0	0.02576	3.20			0	2	680.39258	55.98	EV_set1_060319_050219_Frac3.raw
47132549																					wLPSSSPVTGYR	N-Term(iTRAQ4plex)	Unique	4.013E4	1.375E4	3.763E4	3.499E4	0.004	0.1229	2.08			0	2	747.39667	62.67	NIOP_Eset1_031019_Frac2.raw
47132549																					sYTITGLQPGTDYk	N-Term(iTRAQ4plex); K14(iTRAQ4plex)	Unique	3.307E4	5.449E3	3.512E4	3.468E4	0	0.0047	3.31			0	2	916.48926	51.60	EV_set1_060319_050219_Frac3.raw
47132549																					vPGTSTSATLTGLTR	N-Term(iTRAQ4plex)	Unique	2.568E4	1.429E4	3.888E4	3.390E4	0	0.00535	4.60			0	3	535.96936	45.81	NIOP_Eset1_031019_Frac2.raw

47132549																					vPGTSTSATLTGLTR	N-Term(iTRAQ4plex)	Unique	2.886E4	1.035E4	3.969E4	3.362E4	0	0.00169	4.79			0	3	535.96979	45.69	NIOP_Eset1_031019_Frac2.raw
47132549																					qYNVGPSVSk	N-Term(iTRAQ4plex); K10(iTRAQ4plex)	Unique	3.420E4	6.379E3	3.915E4	3.335E4	0	0.0034	3.22			0	2	683.88263	20.81	NIOP_Eset1_031019_Frac2.raw
47132549																					yEVSVYALk	N-Term(iTRAQ4plex); K9(iTRAQ4plex)	Unique	2.897E4	1.839E4	3.887E4	3.299E4	0.001	0.05581	1.95			0	3	453.93048	55.91	EV_set1_060319_050219_Frac3.raw
47132549																					ySFcTDHTVLVQTR	N-Term(iTRAQ4plex); C4(Methylthio)	Unique	3.878E4	1.429E4	3.361E4	3.298E4	0	0.00333	3.61			0	3	620.63434	57.60	EV_set1_060319_050219_Frac3.raw
47132549																					ySFcTDHTVLVQTR	N-Term(iTRAQ4plex); C4(Methylthio)	Unique	4.149E4	2.000E4	3.149E4	3.294E4	0	0.00384	3.56			0	3	620.63464	57.61	EV_set1_060319_050219_Frac3.raw
47132549																					wLPSSSPVTGYR	N-Term(iTRAQ4plex)	Unique	4.109E4	1.071E4	5.225E4	3.288E4	0.004	0.1299	2.03			0	2	747.39734	61.56	NIOP_Eset1_031019_Frac2.raw
47132549																					lGVRPSQGGEAPR	N-Term(iTRAQ4plex)	Unique	2.948E4	1.040E4	3.507E4	3.231E4	0.002	0.09627	2.69			0	3	489.94308	13.61	NIOP_Eset1_031019_Frac6.raw
47132549																					cDPHEATcYDDGk	N-Term(iTRAQ4plex); C1(Methylthio); C8(Methylthio); K13(iTRAQ4plex)	Unique	4.364E4	8.220E3	3.652E4	3.214E4	0	9.6E-05	3.77			0	3	611.91028	38.36	EV_set1_060319_050219_Frac5.raw
47132549																					sYTITGLQPGTDYk	N-Term(iTRAQ4plex); K14(iTRAQ4plex)	Unique	2.817E4	1.907E4	3.080E4	3.187E4	0.008	0.1787	2.31			0	3	611.32806	51.22	EV_set1_060319_050219_Frac5.raw
47132549																					vFAVSHGR	N-Term(iTRAQ4plex)	Unique	2.728E4	1.478E4	3.350E4	3.120E4	0.008	0.1774	2.52			0	2	508.79285	29.00	EV_set1_060319_050219_Frac3.raw
47132549																					hTSVQTTSSGSGPFTDVR	N-Term(iTRAQ4plex)	Unique	3.235E4	2.500E4	3.148E4	3.118E4	0	0.0042	4.04			0	3	670.00067	32.90	EV_set1_060319_050219_Frac3.raw
47132549																					eATIPGHLNSYTIk	N-Term(iTRAQ4plex); K14(iTRAQ4plex)	Unique	3.930E4	1.091E4	4.487E4	3.107E4	0	0.00012	4.28			0	3	611.34442	42.71	EV_set1_060319_050219_Frac4.raw
47132549																					vPGTSTSATLTGLTR	N-Term(iTRAQ4plex)	Unique	1.765E4	5.703E3	3.892E4	3.101E4	0.001	0.05651	2.69			0	3	535.96948	45.29	NIOP_Eset1_031019_Frac2.raw
47132549																					hTSVQTTSSGSGPFTDVR	N-Term(iTRAQ4plex)	Unique	4.455E4	2.331E4	3.056E4	3.018E4	0	1.4E-05	5.21			0	3	670.00055	32.88	EV_set1_060319_050219_Frac3.raw
47132549																					vPGTSTSATLTGLTR	N-Term(iTRAQ4plex)	Unique	2.203E4	4.186E3	3.536E4	2.981E4	0	0.01643	3.91			0	3	535.96973	45.39	NIOP_Eset1_031019_Frac2.raw
47132549																					syTITGLQPGTDYk	N-Term(iTRAQ4plex); Y2(iTRAQ4plex); K14(iTRAQ4plex)	Unique	1.881E4	3.369E3	3.138E4	2.947E4	0.001	0.01182		14	5.09857	0	3	659.36322	40.70	EV_set1_060319_050219_Frac5.raw
47132549																					syTITGLQPGTDYk	N-Term(iTRAQ4plex); Y2(iTRAQ4plex); K14(iTRAQ4plex)	Unique	2.402E4	4.189E3	4.228E4	2.945E4	0	0.00066		19	1.75569	0	3	659.36322	40.70	EV_set1_060319_050219_Frac5.raw
47132549																					gDSPASSkPISINYR	N-Term(iTRAQ4plex); K8(iTRAQ4plex)	Unique	1.872E4	1.010E4	4.208E4	2.917E4	0	0.00304	3.46			0	4	470.75876	34.37	EV_set1_060319_050219_Frac5.raw
47132549																					gDSPASSkPISINYR	N-Term(iTRAQ4plex); K8(iTRAQ4plex)	Unique	2.302E4	8.289E3	3.839E4	2.893E4	0	0.00987	2.96			0	3	627.34271	34.24	EV_set1_060319_050219_Frac4.raw
47132549																					iTYGETGGNSPVQEFTVPGSk	N-Term(iTRAQ4plex); K21(iTRAQ4plex)	Unique	2.182E4	4.096E3	2.690E4	2.790E4	0	5.2E-05	5.29			0	3	819.42340	60.83	EV_set1_060319_050219_Frac3.raw
47132549																					sYTITGLQPGTDyk	N-Term(iTRAQ4plex); Y13(iTRAQ4plex); K14(iTRAQ4plex)	Unique	2.406E4	8.001E3	3.336E4	2.769E4	0	0.00564		14	6.12683	0	4	494.77356	40.70	EV_set1_060319_050219_Frac4.raw
47132549																					vPGTSTSATLTGLTR	N-Term(iTRAQ4plex)	Unique	2.918E4	6.234E3	3.132E4	2.722E4	0	0.01115	3.19			0	2	803.44983	46.66	NIOP_Eset1_031019_Frac2.raw
47132549																					cDPHEATcYDDGk	N-Term(iTRAQ4plex); C1(Methylthio); C8(Methylthio); K13(iTRAQ4plex)	Unique	2.614E4	6.375E3	2.713E4	2.717E4	0	0.00149	2.91			0	3	611.91028	38.36	EV_set1_060319_050219_Frac5.raw
47132549																					dLQFVEVTDVk	N-Term(iTRAQ4plex); K11(iTRAQ4plex)	Unique	3.303E4	1.309E4	3.115E4	2.710E4	0	0.00897	2.89			0	3	527.63208	61.53	EV_set1_060319_050219_Frac3.raw
47132549																					tEIDkPSQmQVTDVQDNSISVk	N-Term(iTRAQ4plex); K5(iTRAQ4plex); M9(Oxidation); K22(iTRAQ4plex)	Unique	2.942E4	8.734E3	3.922E4	2.693E4	0	1.5E-05	4.69			0	4	728.38306	40.80	EV_set1_060319_050219_Frac5.raw
47132549																					hTSVQTTSSGSGPFTDVR	N-Term(iTRAQ4plex)	Unique	3.597E4	1.920E4	3.023E4	2.653E4	0	5E-05	4.99			0	3	670.00055	32.88	EV_set1_060319_050219_Frac3.raw
47132549																					eATIPGHLNSYTIk	N-Term(iTRAQ4plex); K14(iTRAQ4plex)	Unique	2.287E4	1.272E4	3.434E4	2.635E4	0	0.00059	3.62			0	3	611.34418	42.52	EV_set1_060319_050219_Frac4.raw
47132549																					dAPIVNk	N-Term(iTRAQ4plex); K7(iTRAQ4plex)	Unique	2.546E4	1.157E4	2.955E4	2.609E4	0.007	0.1691	2.28			0	2	522.81915	29.15	EV_set1_060319_050219_Frac3.raw
47132549																					tEIDkPSQmQVTDVQDNSISVk	N-Term(iTRAQ4plex); K5(iTRAQ4plex); M9(Oxidation); K22(iTRAQ4plex)	Unique	2.323E4	9.064E3	3.202E4	2.596E4	0	0.00023	4.60			0	4	728.38306	40.80	EV_set1_060319_050219_Frac5.raw
47132549																					tEIDkPSQmQVTDVQDNSISVk	N-Term(iTRAQ4plex); K5(iTRAQ4plex); M9(Oxidation); K22(iTRAQ4plex)	Unique	2.298E4	1.129E4	2.964E4	2.589E4	0	8.5E-05	4.19			0	4	728.38306	40.80	EV_set1_060319_050219_Frac5.raw
47132549																					vPGTSTSATLTGLTR	N-Term(iTRAQ4plex)	Unique	2.637E4	7.728E3	3.093E4	2.549E4	0	0.00651	4.32			0	3	535.96924	45.62	NIOP_Eset1_031019_Frac2.raw
47132549																					eATIPGHLNSYTIk	N-Term(iTRAQ4plex); K14(iTRAQ4plex)	Unique	1.964E4	5.293E3	2.979E4	2.519E4	0	0.01283	2.99			0	4	458.75977	42.59	EV_set1_060319_050219_Frac5.raw
47132549																					dLQFVEVTDVk	N-Term(iTRAQ4plex); K11(iTRAQ4plex)	Unique	1.689E4	7.712E3	2.151E4	2.493E4	0	0.00875	2.79			0	3	527.63123	61.36	EV_set1_060319_050219_Frac3.raw
47132549																					dLQFVEVTDVk	N-Term(iTRAQ4plex); K11(iTRAQ4plex)	Unique	1.915E4	9.017E3	2.480E4	2.488E4	0	0.00178	2.79			0	3	527.63110	61.33	EV_set1_060319_050219_Frac3.raw
47132549																					iyLYTLnDNAR	N-Term(iTRAQ4plex); Y2(iTRAQ4plex); N7(Deamidated)	Unique	2.188E4	6.660E3	2.304E4	2.487E4	0	0.00042		42	0.00687	0	3	548.96674	49.80	EV_set1_060319_050219_Frac5.raw
47132549																					gDSPASSkPISINYR	N-Term(iTRAQ4plex); K8(iTRAQ4plex)	Unique	1.719E4	9.238E3	2.738E4	2.466E4	0	0.00319	3.46			0	4	470.75876	34.37	EV_set1_060319_050219_Frac5.raw
47132549																					ySFcTDHTVLVQTR	N-Term(iTRAQ4plex); C4(Methylthio)	Unique	3.180E4	2.770E4	2.520E4	2.448E4	0	0.0299	2.54			0	3	620.63464	57.63	EV_set1_060319_050219_Frac3.raw
47132549																					gDSPASSkPISINYR	N-Term(iTRAQ4plex); K8(iTRAQ4plex)	Unique	2.067E4	1.161E4	2.950E4	2.408E4	0	0.01551	3.21			0	4	470.75839	34.28	EV_set1_060319_050219_Frac4.raw
47132549																					sYTITGLQPGTDYk	N-Term(iTRAQ4plex); K14(iTRAQ4plex)	Unique	1.897E4	1.478E3	2.262E4	2.405E4	0	0.00167	2.95			0	2	916.48767	51.25	EV_set1_060319_050219_Frac5.raw

47132549																					lGVRPSQGGEAPR	N-Term(iTRAQ4plex)	Unique	1.859E4	1.130E4	1.982E4	2.341E4	0.008	0.1791	1.82			0	4	367.70932	20.44	EV_set1_060319_050219_Frac4.raw
47132549																					eATIPGHLNSYTIk	N-Term(iTRAQ4plex); K14(iTRAQ4plex)	Unique	1.652E4	7.146E3	2.799E4	2.314E4	0	0.00316	3.32			0	3	611.34406	42.55	EV_set1_060319_050219_Frac5.raw
47132549																					vFAVSHGR	N-Term(iTRAQ4plex)	Unique	2.205E4	8.189E3	2.705E4	2.274E4	0.002	0.09718	2.45			0	2	508.79187	21.31	EV_set1_060319_050219_Frac4.raw
47132549																					eATIPGHLNSYTIk	N-Term(iTRAQ4plex); K14(iTRAQ4plex)	Unique	2.221E4	1.382E4	2.960E4	2.267E4	0	0.00871	3.33			0	3	611.34430	42.54	EV_set1_060319_050219_Frac4.raw
47132549																					sYTITGLQPGTDYk	N-Term(iTRAQ4plex); K14(iTRAQ4plex)	Unique	2.265E4	1.250E4	2.660E4	2.252E4	0	0.00508	2.96			0	3	611.32776	51.27	EV_set1_060319_050219_Frac4.raw
47132549																					syTITGLQPGTDYk	N-Term(iTRAQ4plex); Y2(iTRAQ4plex); K14(iTRAQ4plex)	Unique	1.770E4	3.763E3	2.267E4	2.248E4	0.001	0.02909		12	9.31591	0	3	659.36285	41.43	EV_set1_060319_050219_Frac5.raw
47132549																					gDSPASSkPISINYR	N-Term(iTRAQ4plex); K8(iTRAQ4plex)	Unique	1.787E4	9.674E3	3.547E4	2.230E4	0	0.01208	3.48			0	3	627.34229	34.20	EV_set1_060319_050219_Frac4.raw
47132549																					gDSPASSkPISINYR	N-Term(iTRAQ4plex); K8(iTRAQ4plex)	Unique	1.683E4	1.361E4	3.090E4	2.227E4	0	0.01076	3.00			0	4	470.75876	34.37	EV_set1_060319_050219_Frac5.raw
47132549																					gDSPASSkPISINYR	N-Term(iTRAQ4plex); K8(iTRAQ4plex)	Unique	1.541E4	1.435E4	2.237E4	2.226E4	0	0.00665	2.92			0	4	470.75888	34.26	EV_set1_060319_050219_Frac4.raw
47132549																					eATIPGHLnSYTIk	N-Term(iTRAQ4plex); N9(Deamidated); K14(iTRAQ4plex)	Unique	2.273E4	8.297E3	2.663E4	2.211E4	0	0.00232	3.50			0	3	611.67212	45.15	EV_set1_060319_050219_Frac5.raw
47132549																					eATIPGHLnSYTIk	N-Term(iTRAQ4plex); N9(Deamidated); K14(iTRAQ4plex)	Unique	1.776E4	8.608E3	2.375E4	2.201E4	0	0.00021	3.71			0	3	611.67206	45.79	EV_set1_060319_050219_Frac5.raw
47132549																					dLQFVEVTDVk	N-Term(iTRAQ4plex); K11(iTRAQ4plex)	Unique	2.045E4	4.585E3	2.633E4	2.194E4	0	0.00011	3.68			0	2	790.94318	61.37	EV_set1_060319_050219_Frac3.raw
47132549																					eATIPGHLNSYTIk	N-Term(iTRAQ4plex); K14(iTRAQ4plex)	Unique	1.969E4	5.498E3	2.606E4	2.161E4	0	0.01099	3.00			0	4	458.75977	42.58	EV_set1_060319_050219_Frac5.raw
47132549																					hTSVQTTSSGSGPFTDVR	N-Term(iTRAQ4plex)	Unique	2.198E4	1.670E4	1.994E4	2.146E4	0	0.01697	2.97			0	3	670.00037	32.99	EV_set1_060319_050219_Frac3.raw
47132549																					qYNVGPSVSk	N-Term(iTRAQ4plex); K10(iTRAQ4plex)	Unique	2.168E4	2.538E3	2.727E4	2.145E4	0	0.01988	2.53			0	2	683.88306	20.98	NIOP_Eset1_031019_Frac2.raw
47132549																					lGVRPSQGGEAPR	N-Term(iTRAQ4plex)	Unique	1.684E4	3.190E3	2.330E4	2.127E4	0	0.00143	3.51			0	3	489.94351	20.85	EV_set1_060319_050219_Frac5.raw
47132549																					gDSPASSkPISINYR	N-Term(iTRAQ4plex); K8(iTRAQ4plex)	Unique	1.458E4	1.152E4	2.460E4	2.125E4	0.003	0.1117	1.92			0	4	470.75888	34.26	EV_set1_060319_050219_Frac4.raw
47132549																					vPGTSTSATLTGLTR	N-Term(iTRAQ4plex)	Unique	2.044E4	7.164E3	3.031E4	2.123E4	0	0.00063	4.35			0	3	535.96887	46.03	NIOP_Eset1_031019_Frac2.raw
47132549																					vPGTSTSATLTGLTR	N-Term(iTRAQ4plex)	Unique	2.278E4	4.019E3	3.156E4	2.121E4	0	0.00228	4.43			0	3	535.96942	45.52	NIOP_Eset1_031019_Frac2.raw
47132549																					eATIPGHLNSYTIk	N-Term(iTRAQ4plex); K14(iTRAQ4plex)	Unique	1.905E4	9.712E3	2.129E4	2.110E4	0	0.01459	3.13			0	3	611.34424	42.58	EV_set1_060319_050219_Frac5.raw
47132549																					iYLYTLNDNAR	N-Term(iTRAQ4plex)	Unique	1.404E4	5.214E3	2.000E4	2.084E4	0	0.01044	2.37			0	2	750.40308	61.00	NIOP_Eset1_031019_Frac2.raw
47132549																					syTITGLQPGTDYk	N-Term(iTRAQ4plex); Y2(iTRAQ4plex); K14(iTRAQ4plex)	Unique	1.544E4	5.205E3	2.312E4	2.058E4	0	0.00685		11	13.0383	0	4	494.77347	41.49	EV_set1_060319_050219_Frac5.raw
47132549																					lGVRPSQGGEAPR	N-Term(iTRAQ4plex)	Unique	2.158E4	6.536E3	2.297E4	2.027E4	0	0.00405	3.90			0	3	489.94339	27.40	EV_set1_060319_050219_Frac3.raw
47132549																					syTITGLQPGTDYk	N-Term(iTRAQ4plex); Y2(iTRAQ4plex); K14(iTRAQ4plex)	Unique	1.379E4	2.821E3	1.862E4	2.016E4	0.001	0.02007		14	6.40125	0	3	659.36267	41.52	EV_set1_060319_050219_Frac4.raw
47132549																					vPGTSTSATLTGLTR	N-Term(iTRAQ4plex)	Unique	1.561E4	4.177E3	3.154E4	2.007E4	0	0.00337	4.14			0	3	535.96967	45.35	NIOP_Eset1_031019_Frac2.raw
47132549																					tFyScTTEGR	N-Term(iTRAQ4plex); Y3(iTRAQ4plex); C5(Methylthio)	Unique	1.946E4	1.651E3	2.314E4	2.005E4	0	0.00221		29	0.04006	0	3	500.23557	35.11	EV_set1_060319_050219_Frac5.raw
47132549																					tEIDkPSQmQVTDVQDNSISVk	N-Term(iTRAQ4plex); K5(iTRAQ4plex); M9(Oxidation); K22(iTRAQ4plex)	Unique	1.664E4	8.023E3	2.474E4	1.954E4	0	0.00045	3.08			0	4	728.38336	40.82	EV_set1_060319_050219_Frac5.raw
47132549																					dLQFVEVTDVk	N-Term(iTRAQ4plex); K11(iTRAQ4plex)	Unique	1.460E4	8.154E3	1.911E4	1.948E4	0	0.00357	3.28			0	3	527.63110	61.34	EV_set1_060319_050219_Frac3.raw
47132549																					gDSPASSkPISINYR	N-Term(iTRAQ4plex); K8(iTRAQ4plex)	Unique	1.165E4	7.447E3	2.136E4	1.935E4	0.003	0.107	2.37			0	3	627.34229	34.19	EV_set1_060319_050219_Frac4.raw
47132549																					eATIPGHLNSYTIk	N-Term(iTRAQ4plex); K14(iTRAQ4plex)	Unique	1.937E4	8.604E3	2.960E4	1.933E4	0	0.01189	3.78			0	4	458.75977	42.62	EV_set1_060319_050219_Frac5.raw
47132549																					cDPHEATcYDDGk	N-Term(iTRAQ4plex); C1(Methylthio); C8(Methylthio); K13(iTRAQ4plex)	Unique	1.965E4	7.255E3	1.926E4	1.930E4	0	0.0084	2.52			0	3	611.91034	38.39	EV_set1_060319_050219_Frac5.raw
47132549																					eATIPGHLNSYTIk	N-Term(iTRAQ4plex); K14(iTRAQ4plex)	Unique	1.389E4	5.556E3	1.866E4	1.892E4	0	0.00378	3.27			0	3	611.34406	42.56	EV_set1_060319_050219_Frac5.raw
47132549																					yEVSVYALk	N-Term(iTRAQ4plex); K9(iTRAQ4plex)	Unique	1.443E4	1.072E4	2.018E4	1.890E4	0.002	0.072	2.06			0	3	453.93094	55.99	EV_set1_060319_050219_Frac3.raw
47132549																					lGVRPSQGGEAPR	N-Term(iTRAQ4plex)	Unique	1.564E4	1.001E4	1.838E4	1.882E4	0	0.03015	2.39			0	4	367.70932	20.44	EV_set1_060319_050219_Frac4.raw
47132549																					iYLYTLNDNAR	N-Term(iTRAQ4plex)	Unique	1.488E4	4.074E3	2.150E4	1.850E4	0.002	0.09462	2.26			0	2	750.40234	60.49	EV_set1_060319_050219_Frac5.raw
47132549																					nLQPASEyTVSLVAIk	N-Term(iTRAQ4plex); Y8(iTRAQ4plex); K16(iTRAQ4plex)	Unique	1.372E4	5.829E3	1.035E4	1.776E4	0	0.00289		15	1.57248	0	4	542.06915	54.77	EV_set1_060319_050219_Frac4.raw
47132549																					eATIPGHLNSYTIk	N-Term(iTRAQ4plex); K14(iTRAQ4plex)	Unique	1.284E4	5.946E3	1.770E4	1.726E4	0.004	0.1192	2.41			0	3	611.34424	42.59	EV_set1_060319_050219_Frac5.raw
47132549																					nLQPASEyTVSLVAIk	N-Term(iTRAQ4plex); Y8(iTRAQ4plex); K16(iTRAQ4plex)	Unique	8.419E3	4.083E3	8.682E3	1.709E4	0	0.00591		11	3.72894	0	4	542.06915	54.76	EV_set1_060319_050219_Frac4.raw
47132549																					vPGTSTSATLTGLTR	N-Term(iTRAQ4plex)	Unique	9.451E3	4.291E3	2.441E4	1.702E4	0.008	0.1217		17	2.42428	0	3	535.96948	31.03	NIOP_Eset1_031019_Frac2.raw

47132549																					sYTITGLQPGTDYk	N-Term(iTRAQ4plex); K14(iTRAQ4plex)	Unique	1.177E4	1.215E3	1.979E4	1.678E4	0	0.01722	2.67			0	2	916.48767	51.25	EV_set1_060319_050219_Frac5.raw
47132549																					dLQFVEVTDVk	N-Term(iTRAQ4plex); K11(iTRAQ4plex)	Unique	1.836E4	6.890E3	1.393E4	1.675E4	0	0.00437	2.73			0	2	790.94476	61.52	EV_set1_060319_050219_Frac3.raw
47132549																					iTGYIIk	N-Term(iTRAQ4plex); K7(iTRAQ4plex)	Unique	1.119E4	3.414E3	2.214E4	1.665E4	0.003	0.1095	2.34			0	2	548.35522	50.41	EV_set1_060319_050219_Frac1.raw
47132549																					vPGTSTSATLTGLTR	N-Term(iTRAQ4plex)	Unique	1.717E4	5.979E3	2.606E4	1.661E4	0	0.00297	4.10			0	3	535.96893	45.86	NIOP_Eset1_031019_Frac2.raw
47132549																					iYLYTLNDNAR	N-Term(iTRAQ4plex)	Unique	1.273E4	4.942E3	2.115E4	1.637E4	0.001	0.04259	2.43			0	2	750.40375	58.07	NIOP_Eset1_031019_Frac6.raw
47132549																					eATIPGHLnSyTIk	N-Term(iTRAQ4plex); N9(Deamidated); Y11(iTRAQ4plex); K14(iTRAQ4plex)	Unique	7.484E3	4.036E3	1.860E4	1.618E4	0.003	0.06941		12	7.9198	0	4	495.02972	39.63	EV_set1_060319_050219_Frac5.raw
47132549																					iYLYTLNDNAR	N-Term(iTRAQ4plex)	Unique	1.446E4	2.958E3	2.038E4	1.614E4	0	0.01847	2.49			0	2	750.40320	60.45	EV_set1_060319_050219_Frac5.raw
47132549																					eATIPGHLNSYTIk	N-Term(iTRAQ4plex); K14(iTRAQ4plex)	Unique	1.726E4	6.289E3	1.928E4	1.601E4	0	0.00774	3.06			0	3	611.34418	42.52	EV_set1_060319_050219_Frac4.raw
47132549																					dLQFVEVTDVk	N-Term(iTRAQ4plex); K11(iTRAQ4plex)	Unique	1.521E4	3.105E3	2.108E4	1.600E4	0	0.00332	3.45			0	2	790.94305	61.39	EV_set1_060319_050219_Frac3.raw
47132549																					lGVRPSQGGEAPR	N-Term(iTRAQ4plex)	Unique	1.493E4	7.797E3	1.986E4	1.593E4	0.004	0.1287	2.55			0	3	489.94339	27.41	EV_set1_060319_050219_Frac3.raw
47132549																					wSRPQAPITGYR	N-Term(iTRAQ4plex)	Unique	1.779E4	8.144E3	2.593E4	1.581E4	0	0.00038	3.84			0	3	525.95532	39.03	EV_set1_060319_050219_Frac5.raw
47132549																					eATIPGHLnSyTIk	N-Term(iTRAQ4plex); N9(Deamidated); Y11(iTRAQ4plex); K14(iTRAQ4plex)	Unique	9.710E3	3.961E3	1.641E4	1.568E4	0.001	0.02751		14	4.18518	0	4	495.02972	39.62	EV_set1_060319_050219_Frac5.raw
47132549																					tFYScTTEGR	N-Term(iTRAQ4plex); C5(Methylthio)	Unique	1.337E4	3.834E3	1.836E4	1.561E4	0.001	0.05911	2.36			0	2	677.79779	46.58	EV_set1_060319_050219_Frac5.raw
47132549																					hTSVQTTSSGSGPFTDVR	N-Term(iTRAQ4plex)	Unique	1.535E4	1.065E4	1.690E4	1.525E4	0	0.00878	3.25			0	3	670.00012	33.40	EV_set1_060319_050219_Frac3.raw
47132549																					sYTITGLQPGTDYk	N-Term(iTRAQ4plex); K14(iTRAQ4plex)	Unique	1.435E4	5.494E3	1.675E4	1.515E4	0	0.00906	3.06			0	3	611.32776	51.27	EV_set1_060319_050219_Frac4.raw
47132549																					iYLYTLNDNAR	N-Term(iTRAQ4plex)	Unique	1.686E4	2.019E3	1.831E4	1.507E4	0.001	0.05684	2.24			0	2	750.40234	61.21	NIOP_Eset1_031019_Frac2.raw
47132549																					vPGTSTSATLTGLTR	N-Term(iTRAQ4plex)	Unique	1.359E4	4.096E3	1.846E4	1.504E4	0	0.00886	3.67			0	3	535.96967	46.20	NIOP_Eset1_031019_Frac2.raw
47132549																					dLQFVEVTDVk	N-Term(iTRAQ4plex); K11(iTRAQ4plex)	Unique	1.051E4	5.583E3	1.476E4	1.492E4	0.002	0.0807	2.32			0	3	527.63153	61.39	EV_set1_060319_050219_Frac3.raw
47132549																					vPGTSTSATLTGLTR	N-Term(iTRAQ4plex)	Unique	1.399E4	4.007E3	2.103E4	1.465E4	0.004	0.1169	1.93			0	2	803.44971	46.83	NIOP_Eset1_031019_Frac2.raw
47132549																					iTYGETGGNSPVQEFTVPGSk	N-Term(iTRAQ4plex); K21(iTRAQ4plex)	Unique	1.227E4	4.283E3	1.680E4	1.464E4	0	0.00115	4.41			0	3	819.42340	60.83	EV_set1_060319_050219_Frac3.raw
47132549																					tYHVGEQWQk	N-Term(iTRAQ4plex); K10(iTRAQ4plex)	Unique	4.913E3	3.710E3	1.450E4	1.464E4	0.004	0.1137	1.87			0	3	521.94452	22.64	NIOP_Eset1_031019_Frac6.raw
47132549																					tYHVGEQWQk	N-Term(iTRAQ4plex); K10(iTRAQ4plex)	Unique	4.437E3	6.932E3	1.624E4	1.445E4	0	0.0257	2.35			0	3	521.94470	22.82	NIOP_Eset1_031019_Frac6.raw
47132549																					lGVRPSQGGEAPR	N-Term(iTRAQ4plex)	Unique	1.589E4	5.810E3	1.561E4	1.440E4	0.004	0.1277	2.61			0	3	489.94357	20.31	EV_set1_060319_050219_Frac4.raw
47132549																					vDVIPVNLPGEHGQR	N-Term(iTRAQ4plex)	Unique	1.295E4	4.711E3	1.649E4	1.418E4	0	0.00041	3.61			0	3	591.99591	52.06	EV_set1_060319_050219_Frac5.raw
47132549																					vPGTSTSATLTGLTR	N-Term(iTRAQ4plex)	Unique	1.294E4	5.729E3	1.686E4	1.403E4	0.004	0.1158	2.66			0	3	535.96991	46.05	NIOP_Eset1_031019_Frac2.raw
47132549																					eATIPGHLnSYTIk	N-Term(iTRAQ4plex); N9(Deamidated); K14(iTRAQ4plex)	Unique	1.038E4	7.290E3	1.464E4	1.394E4	0	0.01946	2.94			0	3	611.67267	45.17	EV_set1_060319_050219_Frac4.raw
47132549																					vDVIPVNLPGEHGQR	N-Term(iTRAQ4plex)	Unique	1.440E4	3.978E3	1.952E4	1.355E4	0	0.00019	4.21			0	3	591.99591	52.06	EV_set1_060319_050219_Frac5.raw
47132549																					vPGTSTSATLTGLTR	N-Term(iTRAQ4plex)	Unique	1.824E4	1.678E3	1.632E4	1.351E4	0.001	0.02289		32	0.07471	0	2	803.45007	47.00	NIOP_Eset1_031019_Frac2.raw
47132549																					eATIPGHLnSYTIk	N-Term(iTRAQ4plex); N9(Deamidated); K14(iTRAQ4plex)	Unique	1.069E4	7.884E3	1.899E4	1.341E4	0	0.00525	3.13			0	3	611.67206	45.80	EV_set1_060319_050219_Frac5.raw
47132549																					vFAVSHGR	N-Term(iTRAQ4plex)	Unique	1.316E4	2.783E3	1.771E4	1.340E4	0.002	0.09613	2.31			0	2	508.79208	21.39	EV_set1_060319_050219_Frac5.raw
47132549																					vPGTSTSATLTGLTR	N-Term(iTRAQ4plex)	Unique	2.239E4	7.764E3	2.282E4	1.304E4	0.002	0.075	3.12			0	3	535.96936	46.37	NIOP_Eset1_031019_Frac2.raw
47132549																					dLQFVEVTDVk	N-Term(iTRAQ4plex); K11(iTRAQ4plex)	Unique	9.129E3	3.740E3	1.053E4	1.285E4	0.001	0.04654	2.27			0	2	790.94354	61.42	EV_set1_060319_050219_Frac3.raw
47132549																					lGVRPSQGGEAPR	N-Term(iTRAQ4plex)	Unique	1.089E4	6.024E3	1.645E4	1.279E4	0	0.01133	2.90			0	4	367.70944	27.47	EV_set1_060319_050219_Frac3.raw
47132549																					tEIDkPSQmQVTDVQDNSISVk	N-Term(iTRAQ4plex); K5(iTRAQ4plex); M9(Oxidation); K22(iTRAQ4plex)	Unique	8.891E3	3.075E3	1.140E4	1.252E4	0	0.00217	2.79			0	5	582.90863	41.06	EV_set1_060319_050219_Frac5.raw
47132549																					tEIDkPSQmqVTDVQDNSISVk	N-Term(iTRAQ4plex); K5(iTRAQ4plex); M9(Oxidation); Q10(Deamidated); K22(iTRAQ4plex)	Unique	8.719E3	5.666E3	1.444E4	1.244E4	0	0.00069	3.19			0	4	728.63037	42.67	EV_set1_060319_050219_Frac5.raw
47132549																					eSkPLTAqQTTk	N-Term(iTRAQ4plex); K3(iTRAQ4plex); Q8(Deamidated); K12(iTRAQ4plex)	Unique	1.025E4	3.809E3	1.405E4	1.243E4	0.006	0.09693		10	9.63269	0	3	589.00861	19.51	EV_set1_060319_050219_Frac4.raw
47132549																					eSkPLTAQQTTk	N-Term(iTRAQ4plex); K3(iTRAQ4plex); K12(iTRAQ4plex)	Unique	1.173E4	5.444E3	1.518E4	1.230E4	0	0.00977	2.62			0	3	588.67987	19.43	EV_set1_060319_050219_Frac4.raw
47132549																					gDSPASSkPISInYR	N-Term(iTRAQ4plex); K8(iTRAQ4plex); N13(Deamidated)	Unique	8.259E3	2.393E3	1.289E4	1.223E4	0.002	0.08687	1.62			0	3	627.67053	36.00	EV_set1_060319_050219_Frac5.raw
47132549																					nLQPASEYTVSLVAIk	N-Term(iTRAQ4plex); K16(iTRAQ4plex)	Unique	1.125E4	5.202E3	1.312E4	1.206E4	0	0.00211	3.52			0	3	674.38959	67.84	EV_set1_060319_050219_Frac3.raw

47132549																					hTSVQTTSSGSGPFTDVR	N-Term(iTRAQ4plex)	Unique	1.482E4	9.774E3	1.321E4	1.199E4	0	0.00125	3.39			0	3	670.00012	33.40	EV_set1_060319_050219_Frac3.raw
47132549																					lGVRPSQGGEAPR	N-Term(iTRAQ4plex)	Unique	1.431E4	3.912E3	1.852E4	1.190E4	0.002	0.07883	2.63			0	3	489.94348	22.88	EV_set1_060319_050219_Frac5.raw
47132549																					eATIPGHLNSYTlk	N-Term(iTRAQ4plex); K14(iTRAQ4plex)	Unique	1.333E4	5.128E3	1.415E4	1.172E4	0.001	0.0495	2.37			0	4	458.75980	42.64	EV_set1_060319_050219_Frac5.raw
47132549																					tEIDkPSQmQVTDVQDNSISVk	N-Term(iTRAQ4plex); K5(iTRAQ4plex); M9(Oxidation); K22(iTRAQ4plex)	Unique	1.054E4	5.676E3	1.294E4	1.164E4	0	0.00199	2.66			0	4	728.38312	40.98	EV_set1_060319_050219_Frac5.raw
47132549																					nLQPASEYTVSLVAlk	N-Term(iTRAQ4plex); K16(iTRAQ4plex)	Unique	1.149E4	5.017E3	1.427E4	1.159E4	0	0.00141	3.54			0	3	674.38959	67.85	EV_set1_060319_050219_Frac3.raw
47132549																					yEVSVYALk	N-Term(iTRAQ4plex); K9(iTRAQ4plex)	Unique	6.527E3	1.868E3	1.399E4	1.107E4	0	0.0313	1.86			0	3	453.93042	55.48	EV_set1_060319_050219_Frac5.raw
47132549																					eATIPGHLnSYTlk	N-Term(iTRAQ4plex); N9(Deamidated); K14(iTRAQ4plex)	Unique	1.343E4	5.596E3	1.552E4	1.100E4	0	0.02115	3.29			0	3	611.67249	45.19	EV_set1_060319_050219_Frac5.raw
47132549																					iYLYTLnDNAR	N-Term(iTRAQ4plex); N7(Deamidated)	Unique	8.464E3	2.584E3	1.219E4	1.091E4	0	0.01953	2.51			0	2	750.89508	65.37	EV_set1_060319_050219_Frac3.raw
47132549																					eATIPGHLNSYTlk	N-Term(iTRAQ4plex); K14(iTRAQ4plex)	Unique	1.152E4	3.322E3	1.390E4	1.054E4	0	0.00567	2.74			0	2	916.51312	42.68	EV_set1_060319_050219_Frac5.raw
47132549																					iYLYTLnDNAR	N-Term(iTRAQ4plex); N7(Deamidated)	Unique	7.036E3	1.532E3	9.016E3	1.027E4	0.003	0.1074	1.66			0	2	750.89465	64.97	EV_set1_060319_050219_Frac5.raw
47132549																					gDSPASSkPISInYR	N-Term(iTRAQ4plex); K8(iTRAQ4plex); N13(Deamidated)	Unique	9.038E3	2.591E3	1.419E4	1.022E4	0	0.01949	2.47			0	3	627.67053	36.00	EV_set1_060319_050219_Frac5.raw
47132549																					eATIPGHLNSYTlk	N-Term(iTRAQ4plex); K14(iTRAQ4plex)	Unique	9.104E3	5.434E3	1.147E4	9.973E3	0.009	0.1875	2.01			0	3	611.34436	42.64	EV_set1_060319_050219_Frac5.raw
47132549																					vTImWTPPESAVTGyR	N-Term(iTRAQ4plex); M4(Oxidation); Y15(iTRAQ4plex)	Unique	6.772E3	2.788E3	8.243E3	9.784E3	0	0.00614		15	4.61498	0	4	528.78217	55.20	EV_set1_060319_050219_Frac5.raw
47132549																					eATIPGHLnSYTlk	N-Term(iTRAQ4plex); N9(Deamidated); K14(iTRAQ4plex)	Unique	8.333E3	3.914E3	8.944E3	9.744E3	0	0.01037	2.82			0	3	611.67212	45.15	EV_set1_060319_050219_Frac5.raw
47132549																					dDkESVPISDTIIPAVPPPTDLR	N-Term(iTRAQ4plex); K3(iTRAQ4plex)	Unique	7.353E3	6.309E3	8.960E3	9.461E3	0	1.8E-05	6.01			1	4	691.63141	68.34	EV_set1_060319_050219_Frac5.raw
47132549																					qYNVGPSVSk	N-Term(iTRAQ4plex); K10(iTRAQ4plex)	Unique	3.371E3	5.171E3	9.657E3	9.289E3	0.001	0.04064	2.07			0	3	456.25778	20.78	NIOP_Eset1_031019_Frac2.raw
47132549																					iTyGETGGNSPVQEFTVPGSk	N-Term(iTRAQ4plex); Y3(iTRAQ4plex); K21(iTRAQ4plex)	Unique	1.012E4	6.170E3	1.135E4	9.289E3	0	0.002		26	0.33247	0	4	650.84375	49.24	EV_set1_060319_050219_Frac4.raw
47132549																					sYTITGLqPGTDYk	N-Term(iTRAQ4plex); Q8(Deamidated); K14(iTRAQ4plex)	Unique	7.466E3	3.558E3	1.248E4	9.138E3	0	0.00955	2.91			0	3	611.65576	53.96	EV_set1_060319_050219_Frac5.raw
47132549																					gDSPASSkPISINYR	N-Term(iTRAQ4plex); K8(iTRAQ4plex)	Unique	7.210E3	2.254E3	1.226E4	9.037E3	0	0.00708	3.56			0	2	940.51025	34.33	EV_set1_060319_050219_Frac4.raw
47132549																					nLQPASEYTVSLVAlk	N-Term(iTRAQ4plex); K16(iTRAQ4plex)	Unique	7.612E3	4.053E3	1.088E4	8.710E3	0	0.00582	3.07			0	3	674.38928	67.87	EV_set1_060319_050219_Frac3.raw
47132549																					gDSPASSkPISINYR	N-Term(iTRAQ4plex); K8(iTRAQ4plex)	Unique	8.227E3	2.720E3	1.150E4	8.644E3	0	0.00311	3.44			0	2	940.50891	34.37	EV_set1_060319_050219_Frac5.raw
47132549																					nLQPASEYTVSLVAlk	N-Term(iTRAQ4plex); K16(iTRAQ4plex)	Unique	4.318E3	9.726E2	8.576E3	8.612E3	0	0.0032	2.42			0	3	674.38928	67.44	EV_set1_060319_050219_Frac5.raw
47132549																					gDSPASSkPISINYR	N-Term(iTRAQ4plex); K8(iTRAQ4plex)	Unique	5.181E3	1.287E3	9.093E3	8.534E3	0.003	0.1064	1.71			0	3	627.34235	34.34	EV_set1_060319_050219_Frac5.raw
47132549																					tEIDkPSqmQVTDVQDNSISVk	N-Term(iTRAQ4plex); K5(iTRAQ4plex); Q8(Deamidated); M9(Oxidation); K22(iTRAQ4plex)	Unique	8.009E3	2.954E3	8.480E3	8.437E3	0	0.01066	2.88			0	4	728.63104	42.93	EV_set1_060319_050219_Frac5.raw
47132549																					sYTITGLqPGTDYk	N-Term(iTRAQ4plex); Q8(Deamidated); K14(iTRAQ4plex)	Unique	8.249E3	3.257E3	1.224E4	8.397E3	0	0.01531	2.27			0	3	611.65576	53.96	EV_set1_060319_050219_Frac5.raw
47132549																					vDVIPVNLPGEHGQR	N-Term(iTRAQ4plex)	Unique	7.710E3	6.015E3	9.892E3	8.343E3	0	0.02764	2.69			0	3	591.99597	52.11	EV_set1_060319_050219_Frac5.raw
47132549																					tEIDkPSQmQVTDVQDNSISVk	N-Term(iTRAQ4plex); K5(iTRAQ4plex); M9(Oxidation); K22(iTRAQ4plex)	Unique	5.268E3	2.231E3	5.890E3	8.261E3	0.001	0.04371	1.12			0	5	582.90796	40.93	EV_set1_060319_050219_Frac5.raw
47132549																					eATIPGHLnSYTlk	N-Term(iTRAQ4plex); N9(Deamidated); K14(iTRAQ4plex)	Unique	9.182E3	5.325E3	1.181E4	8.237E3	0.001	0.04252	2.60			0	3	611.67267	45.18	EV_set1_060319_050219_Frac4.raw
47132549																					vDVIPVNLPGEHGQR	N-Term(iTRAQ4plex)	Unique	9.262E3	5.114E3	1.175E4	8.147E3	0	0.00204	3.24			0	3	591.99615	52.08	EV_set1_060319_050219_Frac5.raw
47132549																					vPGTSTSATLTGLTR	N-Term(iTRAQ4plex)	Unique	4.822E3	2.838E3	7.483E3	7.849E3	0	0.01176	2.97			0	3	535.96960	49.34	EV_set1_060319_050219_Frac5.raw
47132549																					dDkESVPISDTIIPAVPPPTDLR	N-Term(iTRAQ4plex); K3(iTRAQ4plex)	Unique	6.665E3	3.720E3	9.451E3	7.501E3	0	4E-05	5.31			1	4	691.63141	68.34	EV_set1_060319_050219_Frac5.raw
47132549																					dDkESVPISDTIIPAVPPPTDLR	N-Term(iTRAQ4plex); K3(iTRAQ4plex)	Unique	6.830E3	2.448E3	7.955E3	7.401E3	0	0.00699	3.91			1	4	691.63141	68.34	EV_set1_060319_050219_Frac5.raw
47132549																					eATIPGHLNSYTlk	N-Term(iTRAQ4plex); K14(iTRAQ4plex)	Unique	9.538E3	1.990E3	9.296E3	7.338E3	0	0.01207	2.77			0	2	916.51312	42.68	EV_set1_060319_050219_Frac5.raw
47132549																					iTYGETGGNSPVQEFTVPGSk	N-Term(iTRAQ4plex); K21(iTRAQ4plex)	Unique	6.861E3	2.739E3	1.083E4	7.214E3	0.004	0.1354	2.03			0	3	819.42358	60.86	EV_set1_060319_050219_Frac3.raw
47132549																					dDkESVPISDTIIPAVPPPTDLR	N-Term(iTRAQ4plex); K3(iTRAQ4plex)	Unique	5.876E3	2.548E3	1.054E4	7.104E3	0	0.00283	4.43			1	4	691.63141	68.34	EV_set1_060319_050219_Frac5.raw
47132549																					vPGTSTSATLTGLTR	N-Term(iTRAQ4plex)	Unique	8.063E3	1.658E3	1.008E4	7.074E3	0.002	0.04328		33	0.06387	0	2	803.44934	49.76	EV_set1_060319_050219_Frac1.raw

47132549																					nLQPASEYTVSLVAIk	N-Term(iTRAQ4plex); K16(iTRAQ4plex)	Unique	6.745E3	3.427E3	8.457E3	6.788E3	0.002	0.08193	1.89			0	3	674.38947	67.91	EV_set1_060319_050219_Frac3.raw
47132549																					vPGTSTSATLTGLTR	N-Term(iTRAQ4plex)	Unique	6.210E3	1.729E3	7.223E3	6.775E3	0	0.01181	3.19			0	3	535.96967	49.77	EV_set1_060319_050219_Frac1.raw
47132549																					IGVRPSQGGEAPR	N-Term(iTRAQ4plex)	Unique	5.472E3	2.380E3	1.031E4	6.633E3	0.005	0.1475	2.65			0	2	734.41150	27.44	EV_set1_060319_050219_Frac3.raw
47132549																					vPGTSTSATLTGLTR	N-Term(iTRAQ4plex)	Unique	9.335E3	1.296E3	1.143E4	6.596E3	0	0.03659	3.26			0	3	535.96954	33.33	NIOP_Eset1_031019_Frac2.raw
47132549																					dDkESVPISDTIIPAVPPPTDLR	N-Term(iTRAQ4plex); K3(iTRAQ4plex)	Unique	7.111E3	1.905E3	8.110E3	6.391E3	0	0.0001	5.61			1	4	691.63129	68.36	EV_set1_060319_050219_Frac5.raw
47132549																					eATIPGHLnSYTIk	N-Term(iTRAQ4plex); N9(Deamidated); K14(iTRAQ4plex)	Unique	6.132E3	1.948E3	7.103E3	6.336E3	0.008	0.1768	1.41			0	3	611.67212	45.92	EV_set1_060319_050219_Frac5.raw
47132549																					iYLYTLnDNAR	N-Term(iTRAQ4plex); N7(Deamidated)	Unique	6.446E3	1.634E3	9.298E3	5.890E3	0.004	0.1357	1.59			0	2	750.89508	65.38	EV_set1_060319_050219_Frac3.raw
47132549																					vDVIPVnLPGEHGQR	N-Term(iTRAQ4plex); N7(Deamidated)	Unique	3.689E3	1.726E3	4.650E3	5.814E3	0	0.03048	2.20			0	3	592.32410	54.44	EV_set1_060319_050219_Frac5.raw
47132549																					eSkPLTAQqTTk	N-Term(iTRAQ4plex); K3(iTRAQ4plex); Q9(Deamidated); K12(iTRAQ4plex)	Unique	4.813E3	1.586E3	7.991E3	5.727E3	0.001	0.04402	2.03			0	3	589.00793	19.63	EV_set1_060319_050219_Frac5.raw
47132549																					gDSPASSkPISINYR	N-Term(iTRAQ4plex); K8(iTRAQ4plex)	Unique	6.551E3	2.564E3	9.825E3	5.617E3	0.001	0.05796	2.35			0	2	940.50891	34.38	EV_set1_060319_050219_Frac5.raw
47132549																					IGVRPSqGGEAPR	N-Term(iTRAQ4plex); Q7(Deamidated)	Unique	3.456E3	1.386E3	6.997E3	5.598E3	0.004	0.1356	2.36			0	2	734.90381	28.58	EV_set1_060319_050219_Frac3.raw
47132549																					eSkPLTAQqTTk	N-Term(iTRAQ4plex); K3(iTRAQ4plex); Q9(Deamidated); K12(iTRAQ4plex)	Unique	4.181E3	1.581E3	4.516E3	5.331E3	0	0.03838	1.75			0	3	589.00812	21.65	EV_set1_060319_050219_Frac3.raw
47132549																					iTYGETGGnSPVQEFTVPGSk	N-Term(iTRAQ4plex); N9(Deamidated); K21(iTRAQ4plex)	Unique	4.732E3	2.613E3	6.003E3	5.299E3	0.002	0.08184	2.28			0	3	819.75287	64.47	EV_set1_060319_050219_Frac3.raw
47132549																					nTFAEVTGLSPGVTYyFk	N-Term(iTRAQ4plex); Y16(iTRAQ4plex); K18(iTRAQ4plex)	Unique	4.694E3	1.969E3	5.455E3	4.880E3	0	0.00112		22	0.73463	0	4	607.33002	69.02	EV_set1_060319_050219_Frac5.raw
47132549																					iTYGETGGnSPVQEFTVPGSk	N-Term(iTRAQ4plex); N9(Deamidated); K21(iTRAQ4plex)	Unique	7.005E3	1.401E3	4.634E3	4.872E3	0	0.00436	2.54			0	3	819.75195	62.58	EV_set1_060319_050219_Frac3.raw
47132549																					gATYNIIVEALk	N-Term(iTRAQ4plex); K12(iTRAQ4plex)	Unique	4.655E3	2.474E3	8.120E3	4.833E3	0	0.01416	2.29			0	3	527.31488	74.62	EV_set1_060319_050219_Frac3.raw
47132549																					IGVRPSQGGEAPR	N-Term(iTRAQ4plex)	Unique	5.549E3	1.314E3	6.743E3	4.614E3	0.001	0.06421	2.22			0	4	367.70947	20.45	EV_set1_060319_050219_Frac5.raw
47132549																					nLQPASEYTVSLVAIk	N-Term(iTRAQ4plex); K16(iTRAQ4plex)	Unique	2.656E3	1.257E3	4.429E3	4.517E3	0.001	0.04689	1.78			0	3	674.38922	67.47	EV_set1_060319_050219_Frac5.raw
47132549																					tEIDkPSQmQVTDVQDNSISVk	N-Term(iTRAQ4plex); K5(iTRAQ4plex); M9(Oxidation); K22(iTRAQ4plex)	Unique	7.025E3	7.927E2	9.626E3	4.507E3	0	0.00717	2.32			0	3	970.84198	41.09	EV_set1_060319_050219_Frac5.raw
47132549																					vTImWTPPESAVTGYR	N-Term(iTRAQ4plex); M4(Oxidation)	Unique	2.899E3	9.262E2	3.817E3	4.301E3	0	0.03694	2.34			0	3	656.67230	67.29	EV_set1_060319_050219_Frac5.raw
47132549																					vTImWTPPESAVTGYR	N-Term(iTRAQ4plex); M4(Oxidation)	Unique	3.563E3	1.326E3	4.327E3	4.177E3	0	0.00381	2.94			0	3	656.67261	67.26	EV_set1_060319_050219_Frac5.raw
47132549																					gATYNIIVEALk	N-Term(iTRAQ4plex); K12(iTRAQ4plex)	Unique	2.816E3	2.567E3	5.721E3	3.902E3	0.006	0.1576	1.43			0	3	527.31488	74.63	EV_set1_060319_050219_Frac3.raw
47132549																					vDVIPVnLPGEHGQR	N-Term(iTRAQ4plex); N7(Deamidated)	Unique	4.279E3	1.289E3	4.102E3	3.736E3	0.003	0.106	2.02			0	3	592.32410	54.43	EV_set1_060319_050219_Frac5.raw
47132549																					nLQPASEYTVSLVAIk	N-Term(iTRAQ4plex); K16(iTRAQ4plex)	Unique	3.983E3	2.035E3	6.176E3	3.595E3	0	0.01733	2.29			0	3	674.38922	67.44	EV_set1_060319_050219_Frac5.raw
47132549																					gLkPGVVYEGQLISIQQYGHQEVTR	N-Term(iTRAQ4plex); K3(iTRAQ4plex)	Unique	3.691E3	1.390E3	2.342E3	3.535E3	0	7.5E-06	4.47			0	4	772.67639	56.88	EV_set1_060319_050219_Frac5.raw
47132549																					vPGTSTSATLTGLTR	N-Term(iTRAQ4plex)	Unique	2.874E3	2.281E3	4.534E3	3.498E3	0.002	0.07052	2.55			0	3	535.96967	49.78	EV_set1_060319_050219_Frac1.raw
47132549																					vPGTSTSATLTGLTR	N-Term(iTRAQ4plex)	Unique	4.406E3	2.046E3	4.689E3	3.201E3	0.001	0.03955	2.80			0	3	535.96869	32.99	NIOP_Eset1_031019_Frac2.raw
47132549																					gLkPGVVYEGQLISIQQYGHQEVTR	N-Term(iTRAQ4plex); K3(iTRAQ4plex)	Unique	1.776E3	2.436E3	2.564E3	2.964E3	0	0.00229	3.45			0	4	772.67639	56.88	EV_set1_060319_050219_Frac5.raw
47132549																					fLATTPNSLLVSWQPPR	N-Term(iTRAQ4plex)	Unique	3.214E3	2.332E3	3.698E3	2.770E3	0.003	0.09948	2.27			0	3	691.05310	85.83	EV_set1_060319_050219_Frac3.raw
47132549																					fLATTPNSLLVSWQPPR	N-Term(iTRAQ4plex)	Unique	3.380E3	1.845E3	4.459E3	2.547E3	0	0.00437	2.72			0	3	691.05353	85.61	EV_set1_060319_050219_Frac3.raw
47132549																					iTYGETGGNSPVQEFTVPGSk	N-Term(iTRAQ4plex); K21(iTRAQ4plex)	Unique	2.244E3	9.274E2	4.155E3	2.242E3	0.001	0.05857	1.60			0	3	819.42310	60.44	EV_set1_060319_050219_Frac5.raw
47132549																					fLATTPNSLLVSWQPPR	N-Term(iTRAQ4plex)	Unique	2.684E3	2.101E3	4.062E3	1.346E3	0.006	0.1573	1.32			0	3	691.05353	85.61	EV_set1_060319_050219_Frac3.raw
47132549																					gLkPGVVYEGQLISIQQYGHQEVTR	N-Term(iTRAQ4plex); K3(iTRAQ4plex)	Unique	3.095E3	1.266E3	2.356E3		0	7.9E-05	4.45			0	4	772.67639	56.88	EV_set1_060319_050219_Frac5.raw
47132549																					tEIDkPSQmQVTDVQDNSISVk	N-Term(iTRAQ4plex); K5(iTRAQ4plex); M9(Oxidation); K22(iTRAQ4plex)	Unique	9.490E3		1.244E4	1.115E4	0	0.00012	2.85			0	3	970.84198	41.10	EV_set1_060319_050219_Frac5.raw
47132549																					iTGYIIk	N-Term(iTRAQ4plex); K7(iTRAQ4plex)	Unique	6.833E3		8.924E3	6.030E3	0.008	0.1719	1.81			0	1	1095.70081	43.79	NIOP_Eset1_031019_Frac2.raw
47132549																					dDkESVPISDTIIPAVPPPTDLR	N-Term(iTRAQ4plex); K3(iTRAQ4plex)	Unique	6.596E3		9.440E3	7.215E3	0	1.7E-05	6.41			1	4	691.63159	68.49	EV_set1_060319_050219_Frac5.raw
47132549																					vPGTSTSATLTGLTR	N-Term(iTRAQ4plex)	Unique	4.861E3		6.813E3	3.750E3	0.004	0.1286	2.00			0	3	535.96954	33.51	NIOP_Eset1_031019_Frac2.raw
47132549																					vPGTSTSATLTGLTR	N-Term(iTRAQ4plex)	Unique	4.522E3		9.471E3	6.476E3	0.005	0.1453	2.47			0	3	535.96930	33.16	NIOP_Eset1_031019_Frac2.raw

47132549																					iTYGETGGNSPVQEFTVPGSk	N-Term(iTRAQ4plex); K21(iTRAQ4plex)	Unique	3.883E3		4.827E3	4.829E3	0	0.01381	2.31			0	3	819.42310	60.40	EV_set1_060319_050219_Frac5.raw
47132549																					fLATTPNSLLVSWQPPR	N-Term(iTRAQ4plex)	Unique	3.707E3		9.836E3	3.938E3	0.001	0.04835	2.22			0	2	1036.07751	86.02	EV_set1_060319_050219_Frac3.raw
47132549																					vTImWTPPESAVTGYR	N-Term(iTRAQ4plex); M4(Oxidation)	Unique	2.971E3		4.672E3	5.265E3	0	0.02481	2.32			0	3	656.67261	67.26	EV_set1_060319_050219_Frac5.raw
47132549																					nTFAEVTGLSPGVTyYFk	N-Term(iTRAQ4plex); Y15(iTRAQ4plex); K18(iTRAQ4plex)	Unique	2.924E3		4.868E3	4.065E3	0	0.00222		24	0.47477	0	4	607.33038	69.04	EV_set1_060319_050219_Frac5.raw
47132549																					rPGGEPSPEGTTGQSYNQYSQR	N-Term(iTRAQ4plex)	Unique	1.978E3		1.875E3	1.972E3	0	0.00498	3.92			0	3	847.40216	29.10	EV_set1_060319_050219_Frac3.raw
47132549																					dDkESVPISDTIIPAVPPPTDLR	N-Term(iTRAQ4plex); K3(iTRAQ4plex)	Unique			1.805E3	2.603E3	0	0.00195	3.74			1	3	921.83997	68.29	EV_set1_060319_050219_Frac5.raw
47132549																					dDkESVPISDTIIPAVPPPTDLR	N-Term(iTRAQ4plex); K3(iTRAQ4plex)	Unique			2.255E3	1.207E3	0	0.00396	3.38			1	3	921.84021	68.27	EV_set1_060319_050219_Frac5.raw
55770842	1401	CRP	C-reactive protein	3	6	GBC vs Healthy, XGC	10.71	3.328	2.4E-06	1.661	1.5E-05	3.967	4.2E-06	0.810	0.380	45.0	7.0	59.7	17.6	70.1																	
55770842																					aFVFPk	N-Term(iTRAQ4plex); K6(iTRAQ4plex)	Unique	1.054E5	3.110E5	1.006E5	5.907E5	0.008	0.1735	2.70			0	2	498.80835	50.06	EV_set1_060319_050219_Frac5.raw
55770842																					aFVFPk	N-Term(iTRAQ4plex); K6(iTRAQ4plex)	Unique	5.741E4	1.864E5	5.888E4	4.172E5	0.008	0.1785	2.39			0	2	498.80930	50.41	EV_set1_060319_050219_Frac3.raw
55770842																					aPLTkPLk	N-Term(iTRAQ4plex); K5(iTRAQ4plex); K8(iTRAQ4plex)	Unique	4.301E4	7.142E4	3.902E4	1.316E5	0.007	0.171	2.16			0	3	433.96198	18.72	NIOP_Eset1_031019_Frac6.raw
55770842																					gYSIFSYATk	N-Term(iTRAQ4plex); K10(iTRAQ4plex)	Unique	8.497E3	1.617E4	9.076E3	3.586E4	0	0.0043	3.55			0	2	712.88556	59.89	EV_set1_060319_050219_Frac3.raw
55770842																					gYSIFSYATk	N-Term(iTRAQ4plex); K10(iTRAQ4plex)	Unique	6.286E3	1.156E4	8.776E3	2.446E4	0	0.0352	2.23			0	3	475.59323	59.94	EV_set1_060319_050219_Frac3.raw
55770842																					gYSIFSYATk	N-Term(iTRAQ4plex); K10(iTRAQ4plex)	Unique	4.449E3	5.810E3	9.053E3	1.338E4	0.002	0.07659	1.85			0	3	475.59323	59.95	EV_set1_060319_050219_Frac3.raw
71773110	337	APOA4	apolipoprotein A-IV	8	40	GBC vs Healthy	23.48	0.349	3.3E-16	0.720	1.1E-01	0.749	6.0E-12	0.518	0.930	66.9	35.7	22.8	37.8	20.3																	
71773110																					eAVEHLQk	N-Term(iTRAQ4plex); K8(iTRAQ4plex)	Unique	7.308E3	2.594E3	6.610E3	4.031E3	0.009	0.1874	1.49			0	3	414.57568	19.80	EV_set1_060319_050219_Frac5.raw
71773110																					lGEVNTYAGDLQk	N-Term(iTRAQ4plex); K13(iTRAQ4plex)	Unique	4.124E4	2.736E4	4.104E4	3.147E4	0	0.01472	3.13			0	3	565.97833	50.17	EV_set1_060319_050219_Frac3.raw
71773110																					lGEVNTYAGDLQk	N-Term(iTRAQ4plex); K13(iTRAQ4plex)	Unique	5.080E4	2.457E4	4.050E4	3.060E4	0	0.00216	3.34			0	3	565.97736	50.18	EV_set1_060319_050219_Frac3.raw
71773110																					lkEEIGk	N-Term(iTRAQ4plex); K2(iTRAQ4plex); K7(iTRAQ4plex)	Unique	9.220E4	3.720E4	5.046E4	3.656E4	0.006	0.1523	2.71			1	3	416.93491	17.25	NIOP_Eset1_031019_Frac6.raw
71773110																					lLPHANEVSQk	N-Term(iTRAQ4plex); K11(iTRAQ4plex)	Unique	9.223E4	4.906E4	6.511E4	6.158E4	0	0.00881	3.53			0	3	508.63190	33.94	EV_set1_060319_050219_Frac3.raw
71773110																					lLPHANEVSQk	N-Term(iTRAQ4plex); K11(iTRAQ4plex)	Unique	1.014E5	5.476E4	7.003E4	5.820E4	0	0.01141	3.36			0	3	508.63181	33.92	EV_set1_060319_050219_Frac3.raw
71773110																					lLPHANEVSQk	N-Term(iTRAQ4plex); K11(iTRAQ4plex)	Unique	6.488E4	4.460E4	5.505E4	5.780E4	0.001	0.04895	2.87			0	3	508.63217	34.02	EV_set1_060319_050219_Frac3.raw
71773110																					lLPHANEVSQk	N-Term(iTRAQ4plex); K11(iTRAQ4plex)	Unique	1.283E5	4.195E4	5.942E4	3.183E4	0	0.00052	4.20			0	3	508.63266	23.00	NIOP_Eset1_031019_Frac6.raw
71773110																					lLPHANEVSQk	N-Term(iTRAQ4plex); K11(iTRAQ4plex)	Unique	7.084E4	2.303E4	3.575E4	1.744E4	0	0.00258	3.79			0	3	508.63300	22.83	NIOP_Eset1_031019_Frac6.raw
71773110																					lLPHANEVSQk	N-Term(iTRAQ4plex); K11(iTRAQ4plex)	Unique	5.854E4	1.924E4	3.059E4	1.273E4	0	0.00074	3.73			0	3	508.63248	23.17	NIOP_Eset1_031019_Frac6.raw
71773110																					lLPHANEVSQk	N-Term(iTRAQ4plex); K11(iTRAQ4plex)	Unique	1.686E4	7.546E3	1.255E4	1.188E4	0	0.03579	2.31			0	3	508.63205	30.75	EV_set1_060319_050219_Frac4.raw
71773110																					lLPHANEVSQk	N-Term(iTRAQ4plex); K11(iTRAQ4plex)	Unique	2.491E4	1.026E4	1.452E4	1.001E4	0.003	0.1075	2.54			0	3	508.63254	32.68	EV_set1_060319_050219_Frac5.raw
71773110																					lLPHANEVSQk	N-Term(iTRAQ4plex); K11(iTRAQ4plex)	Unique	1.731E4	9.979E3	8.880E3	7.117E3	0.003	0.1003	1.98			0	3	508.63229	32.61	EV_set1_060319_050219_Frac5.raw
71773110																					lLPHANEVSQk	N-Term(iTRAQ4plex); K11(iTRAQ4plex)	Unique	1.594E4	8.778E3	9.792E3	6.290E3	0	0.01189	2.38			0	3	508.63220	32.60	EV_set1_060319_050219_Frac5.raw
71773110																					lLPHANEVSqk	N-Term(iTRAQ4plex); Q10(Deamidated); K11(iTRAQ4plex)	Unique	1.689E4	5.319E3	8.514E3	5.597E3	0	0.00789	2.58			0	3	508.96008	33.70	EV_set1_060319_050219_Frac5.raw
71773110																					lLPHANEVSQk	N-Term(iTRAQ4plex); K11(iTRAQ4plex)	Unique	1.217E4	4.126E3	8.724E3	4.732E3	0	0.01839	2.33			0	3	508.63199	23.34	NIOP_Eset1_031019_Frac6.raw
71773110																					lLPHANEVSQk	N-Term(iTRAQ4plex); K11(iTRAQ4plex)	Unique	1.042E4	5.729E3	8.737E3	4.555E3	0.004	0.1291	1.78			0	3	508.63293	22.65	NIOP_Eset1_031019_Frac6.raw
71773110																					lNHQLEGLTFQmk	N-Term(iTRAQ4plex); M12(Oxidation); K13(iTRAQ4plex)	Unique	2.964E4	2.569E4	3.921E4	2.702E4	0	0.00315	3.70			0	3	621.67279	46.94	EV_set1_060319_050219_Frac4.raw
71773110																					lNHQLEGLTFQmk	N-Term(iTRAQ4plex); M12(Oxidation); K13(iTRAQ4plex)	Unique	1.963E4	2.491E4	3.121E4	2.366E4	0	0.01465	3.04			0	3	621.67242	45.19	NIOP_Eset1_031019_Frac6.raw
71773110																					lNHQLEGLTFQmk	N-Term(iTRAQ4plex); M12(Oxidation); K13(iTRAQ4plex)	Unique	1.118E4	1.343E4	1.774E4	1.196E4	0	0.00161	3.14			0	3	621.67310	46.90	EV_set1_060319_050219_Frac4.raw
71773110																					lNHQLEGLTFQmk	N-Term(iTRAQ4plex); M12(Oxidation); K13(iTRAQ4plex)	Unique	7.477E3	7.593E3	1.206E4	8.579E3	0	0.00594	2.66			0	3	621.67310	46.91	EV_set1_060319_050219_Frac4.raw
71773110																					lNHQLEGLTFQmk	N-Term(iTRAQ4plex); M12(Oxidation); K13(iTRAQ4plex)	Unique	6.560E3	9.543E3	1.115E4	6.458E3	0.001	0.05873	1.65			0	4	466.50665	46.93	EV_set1_060319_050219_Frac4.raw
71773110																					lNHQLEGLTFQmk	N-Term(iTRAQ4plex); M12(Oxidation); K13(iTRAQ4plex)	Unique	7.083E3	6.033E3	1.064E4	4.783E3	0	0.0127	2.34			0	4	466.50641	46.92	EV_set1_060319_050219_Frac4.raw

71773110																					lNHQLEGLTFQmk	N-Term(iTRAQ4plex); M12(Oxidation); K13(iTRAQ4plex)	Unique	6.472E3	7.581E3	5.683E3	4.066E3	0	0.02114	1.73			0	4	466.50620	47.05	EV_set1_060319_050219_Frac5.raw
71773110																					lTPYADEFk	N-Term(iTRAQ4plex); K9(iTRAQ4plex)	Unique	7.548E4	3.011E4	4.209E4	2.981E4	0	0.02855	2.74			0	2	686.37500	51.86	EV_set1_060319_050219_Frac3.raw
71773110																					rVEPYGENFNk	N-Term(iTRAQ4plex); K11(iTRAQ4plex)	Unique	5.052E4	3.208E4	4.320E4	4.081E4	0	0.00888	3.56			1	3	547.62677	32.61	EV_set1_060319_050219_Frac3.raw
71773110																					rVEPYGENFNk	N-Term(iTRAQ4plex); K11(iTRAQ4plex)	Unique	3.104E4	2.278E4	3.924E4	3.055E4	0.009	0.1878	2.23			1	3	547.62665	32.62	EV_set1_060319_050219_Frac3.raw
71773110																					rVEPYGENFNk	N-Term(iTRAQ4plex); K11(iTRAQ4plex)	Unique	2.319E4	1.428E4	1.907E4	1.757E4	0	0.00354	4.32			1	3	547.62622	20.82	NIOP_Eset1_031019_Frac6.raw
71773110																					vNSFFSTFk	N-Term(iTRAQ4plex); K9(iTRAQ4plex)	Unique	5.780E4	2.178E4	3.242E4	2.234E4	0.001	0.04387	2.74			0	2	682.87750	66.03	EV_set1_060319_050219_Frac3.raw
71773110																					vNSFFSTFk	N-Term(iTRAQ4plex); K9(iTRAQ4plex)	Unique	1.254E5	3.843E4	4.853E4	2.197E4	0	0.03632	2.94			0	3	455.58771	65.73	EV_set1_060319_050219_Frac4.raw
71773110																					vNSFFSTFk	N-Term(iTRAQ4plex); K9(iTRAQ4plex)	Unique	6.090E4	2.622E4	3.441E4	1.902E4	0	0.00294	3.15			0	2	682.87750	66.02	EV_set1_060319_050219_Frac3.raw
71773110																					vNSFFSTFk	N-Term(iTRAQ4plex); K9(iTRAQ4plex)	Unique	1.149E5	3.135E4	3.484E4	1.663E4	0	0.00475	3.49			0	2	682.87762	65.76	EV_set1_060319_050219_Frac4.raw
71773110																					vNSFFSTFk	N-Term(iTRAQ4plex); K9(iTRAQ4plex)	Unique	1.012E5	3.559E4	3.263E4	1.554E4	0	0.03541	3.11			0	3	455.58771	65.73	EV_set1_060319_050219_Frac4.raw
71773110																					vNSFFSTFk	N-Term(iTRAQ4plex); K9(iTRAQ4plex)	Unique	3.926E4	1.732E4	2.505E4	1.482E4	0.008	0.1779	1.82			0	2	682.87726	66.06	EV_set1_060319_050219_Frac3.raw
71773110																					vNSFFSTFk	N-Term(iTRAQ4plex); K9(iTRAQ4plex)	Unique	3.756E4	1.827E4	2.090E4	1.477E4	0.002	0.08234	2.08			0	3	455.58740	66.02	EV_set1_060319_050219_Frac3.raw
71773110																					vNSFFSTFk	N-Term(iTRAQ4plex); K9(iTRAQ4plex)	Unique	2.340E4	9.152E3	1.416E4	1.040E4	0.001	0.04848	2.52			0	3	455.58722	66.06	EV_set1_060319_050219_Frac3.raw
71773110																					vNSFFSTFk	N-Term(iTRAQ4plex); K9(iTRAQ4plex)	Unique	1.889E4	1.246E4	1.418E4	8.561E3	0.003	0.1055	2.32			0	3	455.58740	66.02	EV_set1_060319_050219_Frac3.raw
71773110																					vNSFFSTFk	N-Term(iTRAQ4plex); K9(iTRAQ4plex)	Unique	1.708E4	9.522E3	8.604E3	6.943E3	0.003	0.1099	1.88			0	3	455.58719	65.58	EV_set1_060319_050219_Frac4.raw
71773110																					vNSFFSTFk	N-Term(iTRAQ4plex); K9(iTRAQ4plex)	Unique	2.445E4	1.173E4	1.583E4	6.761E3	0	0.01407	2.35			0	2	682.87689	65.58	EV_set1_060319_050219_Frac4.raw
71773110																					vNSFFSTFk	N-Term(iTRAQ4plex); K9(iTRAQ4plex)	Unique	1.334E4	5.924E3	6.861E3	5.499E3	0.005	0.1404	1.87			0	3	455.58725	65.60	EV_set1_060319_050219_Frac4.raw
89191868	7450	VWF	von Willebrand factor	10	24	GBC vs GSD	4.80	1.150	3.4E-08	2.710	4.4E-20	1.064	3.0E-01	1.113	2.524	27.4	52.1	10.9	35.8	81.2																	
89191868																					iLVGNk	N-Term(iTRAQ4plex); K6(iTRAQ4plex)	Unique	1.476E5	4.174E4	2.408E5	2.172E5	0.008	0.1252		36	0.00358	0	2	466.31277	34.33	EV_set1_060319_050219_Frac3.raw
89191868																					sLScRPPMVk	N-Term(iTRAQ4plex); C4(Methylthio); K10(iTRAQ4plex)	Unique	9.121E4	1.342E4	1.242E5	1.200E5	0	0.00518	3.40			0	3	484.59772	38.51	NIOP_Eset1_031019_Frac6.raw
89191868																					iLVGNk	N-Term(iTRAQ4plex); K6(iTRAQ4plex)	Unique	7.428E4	3.488E4	1.112E5	1.152E5	0.008	0.1754	2.48			0	2	466.31277	34.33	EV_set1_060319_050219_Frac3.raw
89191868																					sLScRPPMVk	N-Term(iTRAQ4plex); C4(Methylthio); K10(iTRAQ4plex)	Unique	6.412E4	1.299E4	8.005E4	8.352E4	0	0.01036	2.74			0	3	484.59766	38.69	NIOP_Eset1_031019_Frac6.raw
89191868																					iLVGNk	N-Term(iTRAQ4plex); K6(iTRAQ4plex)	Unique	3.148E4	1.558E4	5.723E4	5.506E4	0.003	0.0603		43	0.0007	0	2	466.31287	31.87	EV_set1_060319_050219_Frac4.raw
89191868																					sLScRPPMVk	N-Term(iTRAQ4plex); C4(Methylthio); K10(iTRAQ4plex)	Unique	3.921E4	1.260E4	6.086E4	5.459E4	0.001	0.04863	2.48			0	3	484.59796	38.34	NIOP_Eset1_031019_Frac6.raw
89191868																					sLScRPPMVk	N-Term(iTRAQ4plex); C4(Methylthio); K10(iTRAQ4plex)	Unique	5.254E4	7.473E3	5.491E4	4.589E4	0.001	0.05137	2.09			0	3	484.59723	38.87	NIOP_Eset1_031019_Frac6.raw
89191868																					hIVTFDGQNFk	N-Term(iTRAQ4plex); K11(iTRAQ4plex)	Unique	2.454E4	1.149E4	3.468E4	3.457E4	0.001	0.05862	2.39			0	3	531.95972	38.09	NIOP_Eset1_031019_Frac6.raw
89191868																					gGQImTLk	N-Term(iTRAQ4plex); M5(Oxidation); K8(iTRAQ4plex)	Unique	1.966E4	9.232E3	1.638E4	3.239E4	0.006	0.09987		24	0.50241	0	2	576.33978	30.34	EV_set1_060319_050219_Frac3.raw
89191868																					hIVTFDGQNFk	N-Term(iTRAQ4plex); K11(iTRAQ4plex)	Unique	2.043E4	5.278E3	2.416E4	2.360E4	0.001	0.05667	1.95			0	3	531.96008	42.43	EV_set1_060319_050219_Frac5.raw
89191868																					hIVTFDGQNFk	N-Term(iTRAQ4plex); K11(iTRAQ4plex)	Unique	1.523E4	3.945E3	1.722E4	1.799E4	0.003	0.1093	1.74			0	3	531.96008	42.45	EV_set1_060319_050219_Frac5.raw
89191868																					tTcNPcPLGYk	N-Term(iTRAQ4plex); C3(Methylthio); C6(Methylthio); K11(iTRAQ4plex)	Unique	1.482E4	5.245E3	1.156E4	1.332E4	0.001	0.06357	1.96			0	3	526.24780	61.79	EV_set1_060319_050219_Frac3.raw
89191868																					vTVFPIGIGDR	N-Term(iTRAQ4plex)	Unique	1.252E4	3.087E3	1.425E4	1.328E4	0.001	0.04817	2.45			0	2	659.38611	69.35	EV_set1_060319_050219_Frac3.raw
89191868																					aHLLSLVDVmQR	N-Term(iTRAQ4plex); M10(Oxidation)	Unique	2.224E3	2.708E3	1.177E4	1.290E4	0	0.0159	2.40			0	3	514.62427	53.06	EV_set1_060319_050219_Frac5.raw
89191868																					yTLFQIFSk	N-Term(iTRAQ4plex); K9(iTRAQ4plex)	Unique	7.570E3	4.596E2	1.181E4	1.093E4	0	0.02842	2.06			0	2	717.91614	83.86	EV_set1_060319_050219_Frac3.raw
89191868																					hIVTFDGQNFk	N-Term(iTRAQ4plex); K11(iTRAQ4plex)	Unique	9.112E3	4.422E3	8.842E3	1.047E4	0.007	0.1693	1.43			0	3	531.95990	42.51	EV_set1_060319_050219_Frac5.raw
89191868																					aHLLSLVDVmQR	N-Term(iTRAQ4plex); M10(Oxidation)	Unique	3.285E3	3.141E3	1.054E4	1.002E4	0.004	0.1297	1.67			0	3	514.62445	53.20	EV_set1_060319_050219_Frac4.raw
89191868																					aHLLSLVDVmQR	N-Term(iTRAQ4plex); M10(Oxidation)	Unique	2.530E3	4.550E3	7.420E3	9.321E3	0.001	0.03382		22	0.62184	0	3	514.62427	53.06	EV_set1_060319_050219_Frac5.raw
89191868																					aHLLSLVDVmQR	N-Term(iTRAQ4plex); M10(Oxidation)	Unique	3.399E3	3.432E3	6.195E3	8.652E3	0.006	0.1611	1.38			0	3	514.62445	53.12	EV_set1_060319_050219_Frac5.raw
89191868																					aHLLSLVDVmQR	N-Term(iTRAQ4plex); M10(Oxidation)	Unique	3.416E3	3.805E3	8.651E3	8.503E3	0.008	0.1733	1.56			0	3	514.62476	53.31	EV_set1_060319_050219_Frac4.raw

89191868																					gLRPScPnSQSPVk	N-Term(iTRAQ4plex); C6(Methylthio); N8(Deamidated); K14(iTRAQ4plex)	Unique	6.363E3	7.741E3	9.305E3	8.143E3	0.001	0.04298	1.94			0	3	602.31476	36.94	EV_set1_060319_050219_Frac5.raw
89191868																					yLSDHSFLVSQGDR	N-Term(iTRAQ4plex)	Unique	6.382E3	3.909E3	4.058E3	6.843E3	0	0.0349	2.23			0	3	589.96484	49.59	EV_set1_060319_050219_Frac4.raw
89191868																					yTLFQIFSk	N-Term(iTRAQ4plex); K9(iTRAQ4plex)	Unique	4.475E3	1.449E3	6.091E3	5.336E3	0	0.0357	1.74			0	3	478.94650	83.79	EV_set1_060319_050219_Frac3.raw
89191868																					yTLFQIFSk	N-Term(iTRAQ4plex); K9(iTRAQ4plex)	Unique	3.575E3		5.863E3	5.395E3	0.003	0.1099	1.50			0	3	478.94638	83.81	EV_set1_060319_050219_Frac3.raw
105990532	338	APOB	apolipoprotein B-100	56	315	GBC vs GSD	12.58	1.490	8.0E-95	0.375	8.6E-161	1.239	1.8E-08	1.232	0.298	30.7	30.4	25.6	30.5	35.2																	
105990532																					lHVAGNLk	N-Term(iTRAQ4plex); K8(iTRAQ4plex)	Unique	4.233E4	5.463E5	2.049E5	3.249E5	0	0.03848	3.23			0	3	380.57504	18.81	NIOP_Eset1_031019_Frac6.raw
105990532																					lHVAGNLk	N-Term(iTRAQ4plex); K8(iTRAQ4plex)	Unique	2.738E4	5.148E5	1.682E5	2.963E5	0.001	0.0416	3.64			0	3	380.57489	18.82	NIOP_Eset1_031019_Frac6.raw
105990532																					iNPLALk	N-Term(iTRAQ4plex); K7(iTRAQ4plex)	Unique	7.079E4	3.203E5	1.362E5	2.030E5	0.002	0.06852	2.83			0	2	528.85358	52.10	EV_set1_060319_050219_Frac3.raw
105990532																					ikDYFEk	N-Term(iTRAQ4plex); K2(iTRAQ4plex); K7(iTRAQ4plex)	Unique	7.584E4	4.498E5	1.562E5	1.946E5	0.001	0.06424	3.16			1	3	458.93619	34.32	NIOP_Eset1_031019_Frac6.raw
105990532																					kLNELSFk	N-Term(iTRAQ4plex); K1(iTRAQ4plex); K8(iTRAQ4plex)	Unique	9.813E4	5.087E5	1.781E5	1.781E5	0	0.0072	4.41			1	3	470.95966	32.54	NIOP_Eset1_031019_Frac6.raw
105990532																					kLNELSFk	N-Term(iTRAQ4plex); K1(iTRAQ4plex); K8(iTRAQ4plex)	Unique	8.365E4	4.306E5	1.340E5	1.641E5	0	0.00805	4.39			1	3	470.95972	32.36	NIOP_Eset1_031019_Frac6.raw
105990532																					aIQSLk	N-Term(iTRAQ4plex); K6(iTRAQ4plex)	Unique	7.943E4	2.777E5	1.085E5	1.504E5	0.009	0.1858	2.74			0	2	474.30859	32.20	EV_set1_060319_050219_Frac3.raw
105990532																					hIQNIDIQHLAGk	N-Term(iTRAQ4plex); K13(iTRAQ4plex)	Unique	6.906E4	4.215E5	1.166E5	1.465E5	0	8.3E-05	5.12			0	4	444.50937	36.03	NIOP_Eset1_031019_Frac6.raw
105990532																					lVELAHQYk	N-Term(iTRAQ4plex); K9(iTRAQ4plex)	Unique	6.863E4	2.899E5	1.314E5	1.417E5	0	0.01082	3.93			0	3	463.60858	36.01	NIOP_Eset1_031019_Frac6.raw
105990532																					iNPLALk	N-Term(iTRAQ4plex); K7(iTRAQ4plex)	Unique	6.549E4	1.778E5	1.113E5	1.414E5	0.004	0.1176	2.83			0	2	528.85358	52.10	EV_set1_060319_050219_Frac3.raw
105990532																					vSSFYAk	N-Term(iTRAQ4plex); K7(iTRAQ4plex)	Unique	6.676E4	2.345E5	9.332E4	1.283E5	0.009	0.1866	2.60			0	2	545.30859	37.45	EV_set1_060319_050219_Frac3.raw
105990532																					vSTAFVYTk	N-Term(iTRAQ4plex); K9(iTRAQ4plex)	Unique	6.976E4	2.921E5	1.115E5	1.252E5	0	0.00115	3.92			0	2	652.37836	45.81	EV_set1_060319_050219_Frac3.raw
105990532																					vSTAFVYTk	N-Term(iTRAQ4plex); K9(iTRAQ4plex)	Unique	6.843E4	2.394E5	1.265E5	1.201E5	0	0.0069	3.83			0	2	652.37762	45.83	EV_set1_060319_050219_Frac3.raw
105990532																					lVELAHQYk	N-Term(iTRAQ4plex); K9(iTRAQ4plex)	Unique	6.630E4	2.335E5	1.157E5	1.164E5	0	0.0006	4.44			0	3	463.60901	36.19	NIOP_Eset1_031019_Frac6.raw
105990532																					iISDYHQQFR	N-Term(iTRAQ4plex)	Unique	8.465E4	1.180E5	1.010E5	1.136E5	0	0.00494	3.73			0	3	484.25641	39.46	EV_set1_060319_050219_Frac3.raw
105990532																					lATALSLSNk	N-Term(iTRAQ4plex); K10(iTRAQ4plex)	Unique	3.965E4	2.077E5	6.367E4	1.098E5	0	0.03275	3.10			0	3	435.93665	49.99	EV_set1_060319_050219_Frac3.raw
105990532																					nYQLYk	N-Term(iTRAQ4plex); K6(iTRAQ4plex)	Unique	5.640E4	1.961E5	9.212E4	1.062E5	0.009	0.1893	2.55			0	2	558.81677	35.09	EV_set1_060319_050219_Frac3.raw
105990532																					lSNVLQQVk	N-Term(iTRAQ4plex); K9(iTRAQ4plex)	Unique	3.963E4	2.403E5	8.962E4	1.045E5	0	0.00231	4.39			0	2	658.90918	51.60	EV_set1_060319_050219_Frac3.raw
105990532																					lATALSLSNk	N-Term(iTRAQ4plex); K10(iTRAQ4plex)	Unique	3.723E4	2.115E5	6.603E4	9.493E4	0	0.00275	4.28			0	2	653.40149	50.07	EV_set1_060319_050219_Frac3.raw
105990532																					hIQNIDIQHLAGk	N-Term(iTRAQ4plex); K13(iTRAQ4plex)	Unique	6.396E4	1.471E5	8.672E4	9.363E4	0	0.00018	4.95			0	3	592.34290	35.86	NIOP_Eset1_031019_Frac6.raw
105990532																					vLVDHFGYTk	N-Term(iTRAQ4plex); K10(iTRAQ4plex)	Unique	5.025E4	1.828E5	8.227E4	9.338E4	0	0.02566	2.95			0	3	489.61218	44.55	EV_set1_060319_050219_Frac3.raw
105990532																					sVGFHLPSR	N-Term(iTRAQ4plex)	Unique	4.121E4	3.276E5	6.324E4	9.293E4	0.001	0.0397	3.32			0	3	381.88409	29.58	NIOP_Eset1_031019_Frac6.raw
105990532																					hIQNIDIQHLAGk	N-Term(iTRAQ4plex); K13(iTRAQ4plex)	Unique	8.893E4	5.195E4	4.194E4	9.226E4	0.005	0.1443	1.35			0	4	444.50922	39.83	EV_set1_060319_050219_Frac5.raw
105990532																					lSNVLQQVk	N-Term(iTRAQ4plex); K9(iTRAQ4plex)	Unique	4.320E4	1.899E5	8.650E4	9.139E4	0	0.00998	3.86			0	2	658.90918	51.62	EV_set1_060319_050219_Frac3.raw
105990532																					hIQNIDIQHLAGk	N-Term(iTRAQ4plex); K13(iTRAQ4plex)	Unique	5.235E4	1.163E5	7.884E4	9.067E4	0	3.8E-05	5.13			0	3	592.34332	36.04	NIOP_Eset1_031019_Frac6.raw
105990532																					lVELAHQYk	N-Term(iTRAQ4plex); K9(iTRAQ4plex)	Unique	4.505E4	1.599E5	9.334E4	8.991E4	0	0.00346	3.67			0	3	463.60883	35.83	NIOP_Eset1_031019_Frac6.raw
105990532																					hINIDQFVR	N-Term(iTRAQ4plex)	Unique	6.997E4	1.699E5	7.983E4	8.970E4	0	0.01538	3.36			0	3	429.24225	46.43	EV_set1_060319_050219_Frac3.raw
105990532																					fIIPSPk	N-Term(iTRAQ4plex); K7(iTRAQ4plex)	Unique	4.191E4	2.004E5	6.436E4	8.846E4	0.005	0.1401	2.39			0	2	545.34778	52.62	EV_set1_060319_050219_Frac5.raw
105990532																					hIQNIDIQHLAGk	N-Term(iTRAQ4plex); K13(iTRAQ4plex)	Unique	5.981E4	1.266E5	8.747E4	8.838E4	0	0.00012	4.58			0	3	592.34387	35.69	NIOP_Eset1_031019_Frac6.raw
105990532																					iISDYHQQFR	N-Term(iTRAQ4plex)	Unique	7.178E4	8.572E4	8.406E4	8.818E4	0.001	0.05547	2.46			0	3	484.25641	39.47	EV_set1_060319_050219_Frac3.raw
105990532																					vSTAFVYTk	N-Term(iTRAQ4plex); K9(iTRAQ4plex)	Unique	4.174E4	2.022E5	6.351E4	8.611E4	0.002	0.09607	2.46			0	3	435.25397	45.83	EV_set1_060319_050219_Frac3.raw
105990532																					hIQNIDIQHLAGk	N-Term(iTRAQ4plex); K13(iTRAQ4plex)	Unique	7.885E4	8.385E4	4.540E4	8.602E4	0.001	0.06042	2.15			0	4	444.50922	39.82	EV_set1_060319_050219_Frac5.raw
105990532																					kLNELSFk	N-Term(iTRAQ4plex); K1(iTRAQ4plex); K8(iTRAQ4plex)	Unique	3.483E4	1.862E5	7.330E4	8.428E4	0	0.01007	3.54			1	3	470.95978	32.19	NIOP_Eset1_031019_Frac6.raw
105990532																					lATALSLSNk	N-Term(iTRAQ4plex); K10(iTRAQ4plex)	Unique	5.255E4	1.229E5	6.844E4	8.203E4	0	0.00809	3.60			0	2	653.40149	50.08	EV_set1_060319_050219_Frac3.raw
105990532																					hIQNIDIQHLAGk	N-Term(iTRAQ4plex); K13(iTRAQ4plex)	Unique	6.755E4	9.240E4	4.398E4	8.088E4	0	0.02247	3.13			0	4	444.50922	39.82	EV_set1_060319_050219_Frac5.raw

105990532																					vLVDHFGYTk	N-Term(iTRAQ4plex); K10(iTRAQ4plex)	Unique	3.781E4	1.167E5	5.770E4	7.854E4	0	0.02034	3.05			0	3	489.61218	44.55	EV_set1_060319_050219_Frac3.raw
105990532																					hIQNIDIQHLAGk	N-Term(iTRAQ4plex); K13(iTRAQ4plex)	Unique	5.648E4	5.553E4	7.523E4	7.721E4	0.001	0.05066	2.85			0	3	592.34448	35.50	NIOP_Eset1_031019_Frac6.raw
105990532																					lATALSLSNk	N-Term(iTRAQ4plex); K10(iTRAQ4plex)	Unique	3.633E4	1.330E5	6.651E4	7.598E4	0	0.02904	3.15			0	3	435.93665	50.00	EV_set1_060319_050219_Frac3.raw
105990532																					nIFNFk	N-Term(iTRAQ4plex); K6(iTRAQ4plex)	Unique	2.785E4	1.336E5	4.818E4	7.528E4	0.008	0.1764	2.56			0	2	535.81409	54.43	EV_set1_060319_050219_Frac3.raw
105990532																					vSTAFVYTk	N-Term(iTRAQ4plex); K9(iTRAQ4plex)	Unique	5.597E4	1.141E5	7.494E4	7.508E4	0.001	0.05131	3.19			0	2	652.37726	45.86	EV_set1_060319_050219_Frac3.raw
105990532																					hVAEAIck	N-Term(iTRAQ4plex); C7(Methylthio); K8(iTRAQ4plex)	Unique	4.017E4	1.054E5	5.858E4	7.268E4	0	0.01502	2.90			0	3	402.21826	20.85	NIOP_Eset1_031019_Frac6.raw
105990532																					lSNVLQQVk	N-Term(iTRAQ4plex); K9(iTRAQ4plex)	Unique	4.023E4	1.448E5	8.456E4	7.255E4	0.001	0.06288	2.78			0	2	658.90918	51.64	EV_set1_060319_050219_Frac3.raw
105990532																					aAIQALR	N-Term(iTRAQ4plex)	Unique	5.936E4	1.770E5	6.375E4	7.194E4	0.009	0.132		33	0.00605	0	2	443.78235	19.57	NIOP_Eset1_031019_Frac2.raw
105990532																					lPYTIITTPPLk	N-Term(iTRAQ4plex); K12(iTRAQ4plex)	Unique	2.633E4	1.860E5	4.615E4	7.150E4	0	0.01824	3.19			0	3	549.01080	73.77	EV_set1_060319_050219_Frac3.raw
105990532																					lLSGGNTLHLVSTTk	N-Term(iTRAQ4plex); K15(iTRAQ4plex)	Unique	3.601E4	1.543E5	6.424E4	6.852E4	0	0.00014	4.23			0	3	610.36176	51.41	EV_set1_060319_050219_Frac3.raw
105990532																					hVAEAIck	N-Term(iTRAQ4plex); C7(Methylthio); K8(iTRAQ4plex)	Unique	4.045E4	1.068E5	3.965E4	6.779E4	0	0.01705	2.34			0	3	402.21844	34.30	EV_set1_060319_050219_Frac3.raw
105990532																					lVELAHQYk	N-Term(iTRAQ4plex); K9(iTRAQ4plex)	Unique	3.379E4	1.070E5	6.668E4	6.489E4	0	0.03126	2.77			0	3	463.60995	35.65	NIOP_Eset1_031019_Frac6.raw
105990532																					iNPLALk	N-Term(iTRAQ4plex); K7(iTRAQ4plex)	Unique	2.060E4	8.346E4	3.850E4	5.961E4	0.001	0.0431	2.56			0	2	528.85327	51.71	EV_set1_060319_050219_Frac5.raw
105990532																					lLSGGNTLHLVSTTk	N-Term(iTRAQ4plex); K15(iTRAQ4plex)	Unique	4.112E4	4.437E4	6.198E4	5.908E4	0.007	0.1622	2.05			0	3	610.36157	51.48	EV_set1_060319_050219_Frac3.raw
105990532																					ikDYFEk	N-Term(iTRAQ4plex); K2(iTRAQ4plex); K7(iTRAQ4plex)	Unique	4.000E4	1.258E5	5.224E4	5.875E4	0.008	0.1753	2.86			1	3	458.93564	34.50	NIOP_Eset1_031019_Frac6.raw
105990532																					vLVDHFGYTk	N-Term(iTRAQ4plex); K10(iTRAQ4plex)	Unique	3.836E4	8.830E4	6.235E4	5.862E4	0	0.03376	2.80			0	3	489.61243	44.60	EV_set1_060319_050219_Frac3.raw
105990532																					tSQcTLk	N-Term(iTRAQ4plex); C4(Methylthio); K7(iTRAQ4plex)	Unique	3.020E4	9.634E4	4.083E4	5.753E4	0.002	0.08671	2.35			0	2	557.79449	33.61	EV_set1_060319_050219_Frac3.raw
105990532																					lATALSLSNk	N-Term(iTRAQ4plex); K10(iTRAQ4plex)	Unique	3.002E4	7.780E4	4.716E4	5.641E4	0.005	0.1416	2.30			0	3	435.93665	50.06	EV_set1_060319_050219_Frac3.raw
105990532																					lSNVLQqVk	N-Term(iTRAQ4plex); Q7(Deamidated); K9(iTRAQ4plex)	Unique	4.398E4	1.065E5	4.353E4	5.635E4	0	0.00071	3.99			0	2	659.40039	55.22	EV_set1_060319_050219_Frac3.raw
105990532																					vSTAFVYTk	N-Term(iTRAQ4plex); K9(iTRAQ4plex)	Unique	3.665E4	1.099E5	4.929E4	5.542E4	0.001	0.05223	2.84			0	3	435.25397	45.84	EV_set1_060319_050219_Frac3.raw
105990532																					fIIPGLk	N-Term(iTRAQ4plex); K7(iTRAQ4plex)	Unique	2.055E4	1.309E5	3.591E4	5.498E4	0.004	0.1259	2.53			0	2	538.35791	66.41	NIOP_Eset1_031019_Frac6.raw
105990532																					sPAFTDLHLR	N-Term(iTRAQ4plex)	Unique	2.611E4	1.233E5	4.793E4	5.400E4	0	0.01997	4.02			0	3	434.24200	46.08	EV_set1_060319_050219_Frac3.raw
105990532																					lSNVLQQVk	N-Term(iTRAQ4plex); K9(iTRAQ4plex)	Unique	3.252E4	1.137E5	5.305E4	5.373E4	0.001	0.05271	2.74			0	3	439.60855	51.65	EV_set1_060319_050219_Frac3.raw
105990532																					vLVDHFGYTk	N-Term(iTRAQ4plex); K10(iTRAQ4plex)	Unique	3.244E4	1.571E5	6.643E4	5.317E4	0	0.00368	3.53			0	3	489.61255	38.87	NIOP_Eset1_031019_Frac6.raw
105990532																					lSNVLQQVk	N-Term(iTRAQ4plex); K9(iTRAQ4plex)	Unique	2.684E4	1.242E5	4.319E4	5.269E4	0.001	0.05056	2.57			0	3	439.60870	51.63	EV_set1_060319_050219_Frac3.raw
105990532																					qHLFVk	N-Term(iTRAQ4plex); K6(iTRAQ4plex)	Unique	3.495E3	1.241E5	4.245E4	5.196E4	0.003	0.1107	2.27			0	3	353.88910	16.88	NIOP_Eset1_031019_Frac6.raw
105990532																					ikDYFEk	N-Term(iTRAQ4plex); K2(iTRAQ4plex); K7(iTRAQ4plex)	Unique	1.999E4	1.136E5	4.051E4	5.194E4	0.004	0.1322	2.84			1	3	458.93661	40.43	EV_set1_060319_050219_Frac5.raw
105990532																					sPAFTDLHLR	N-Term(iTRAQ4plex)	Unique	3.003E4	8.586E4	4.419E4	5.038E4	0	0.01422	3.87			0	3	434.24200	46.09	EV_set1_060319_050219_Frac3.raw
105990532																					kLNELSFk	N-Term(iTRAQ4plex); K1(iTRAQ4plex); K8(iTRAQ4plex)	Unique	3.176E4	9.903E4	5.301E4	4.751E4	0	0.01247	3.60			1	3	470.95947	32.71	NIOP_Eset1_031019_Frac6.raw
105990532																					iNPLALk	N-Term(iTRAQ4plex); K7(iTRAQ4plex)	Unique	1.314E4	6.120E4	3.435E4	4.560E4	0	0.03202	2.71			0	2	528.85339	51.78	EV_set1_060319_050219_Frac4.raw
105990532																					ikDYFEk	N-Term(iTRAQ4plex); K2(iTRAQ4plex); K7(iTRAQ4plex)	Unique	2.239E4	7.461E4	3.264E4	4.546E4	0.003	0.06929		29	0.13874	1	3	458.93640	40.41	EV_set1_060319_050219_Frac4.raw
105990532																					tPALHFk	N-Term(iTRAQ4plex); K7(iTRAQ4plex)	Unique	1.690E4	1.099E5	5.864E4	4.505E4	0.005	0.144	2.61			0	3	367.89273	23.71	NIOP_Eset1_031019_Frac6.raw
105990532																					fIIPGLk	N-Term(iTRAQ4plex); K7(iTRAQ4plex)	Unique	2.209E4	1.044E5	3.626E4	4.499E4	0.004	0.1252	2.22			0	2	538.35803	66.75	NIOP_Eset1_031019_Frac6.raw
105990532																					lLSGGNTLHLVSTTk	N-Term(iTRAQ4plex); K15(iTRAQ4plex)	Unique	2.985E4	7.681E4	3.758E4	4.419E4	0	0.00014	4.26			0	3	610.36176	51.40	EV_set1_060319_050219_Frac3.raw
105990532																					kLNELSFk	N-Term(iTRAQ4plex); K1(iTRAQ4plex); K8(iTRAQ4plex)	Unique	2.028E4	6.045E4	3.207E4	4.327E4	0	0.03799	2.87			1	3	470.95984	32.01	NIOP_Eset1_031019_Frac6.raw
105990532																					fIIPSPk	N-Term(iTRAQ4plex); K7(iTRAQ4plex)	Unique	2.708E4	8.959E4	3.946E4	4.288E4	0.001	0.04698	2.43			0	2	545.34778	47.57	NIOP_Eset1_031019_Frac6.raw
105990532																					lVELAHQYk	N-Term(iTRAQ4plex); K9(iTRAQ4plex)	Unique	2.349E4	1.004E5	2.683E4	4.263E4	0	0.01437	3.06			0	3	463.60880	41.18	EV_set1_060319_050219_Frac4.raw
105990532																					lLSGGNTLHLVSTTk	N-Term(iTRAQ4plex); K15(iTRAQ4plex)	Unique	3.019E4	7.653E4	4.683E4	4.216E4	0	0.00076	4.50			0	3	610.36194	51.44	EV_set1_060319_050219_Frac3.raw

105990532																					fIIPSPk	N-Term(iTRAQ4plex); K7(iTRAQ4plex)	Unique	2.510E4	8.448E4	3.513E4	4.162E4	0.004	0.1216	2.38			0	2	545.34808	47.21	NIOP_Eset1_031019_Frac6.raw
105990532																					fIIPSPk	N-Term(iTRAQ4plex); K7(iTRAQ4plex)	Unique	2.482E4	9.163E4	3.753E4	4.137E4	0.002	0.07998	2.18			0	2	545.34802	47.39	NIOP_Eset1_031019_Frac6.raw
105990532																					iISDYHQQFR	N-Term(iTRAQ4plex)	Unique	1.910E4	8.472E4	2.958E4	4.134E4	0	0.00257	4.02			0	3	484.25641	38.24	EV_set1_060319_050219_Frac5.raw
105990532																					fIIPSPk	N-Term(iTRAQ4plex); K7(iTRAQ4plex)	Unique	1.828E4	1.038E5	2.812E4	4.085E4	0.006	0.09559		26	0.18784	0	3	363.90036	53.12	EV_set1_060319_050219_Frac3.raw
105990532																					fIIPGLk	N-Term(iTRAQ4plex); K7(iTRAQ4plex)	Unique	2.184E4	1.135E5	3.004E4	4.012E4	0.002	0.08055	2.22			0	3	359.24127	67.26	EV_set1_060319_050219_Frac4.raw
105990532																					vLVDHFGYTk	N-Term(iTRAQ4plex); K10(iTRAQ4plex)	Unique	1.768E4	1.035E5	3.785E4	3.983E4	0	0.01594	3.19			0	3	489.61252	43.57	EV_set1_060319_050219_Frac5.raw
105990532																					lSNVLQqVk	N-Term(iTRAQ4plex); Q7(Deamidated); K9(iTRAQ4plex)	Unique	3.041E4	6.081E4	3.361E4	3.976E4	0.001	0.0661	3.20			0	2	659.40039	55.22	EV_set1_060319_050219_Frac3.raw
105990532																					fIIPGLk	N-Term(iTRAQ4plex); K7(iTRAQ4plex)	Unique	1.906E4	9.682E4	2.268E4	3.825E4	0.004	0.1306	2.32			0	2	538.35828	67.24	EV_set1_060319_050219_Frac5.raw
105990532																					fIIPGLk	N-Term(iTRAQ4plex); K7(iTRAQ4plex)	Unique	1.231E4	9.563E4	2.650E4	3.748E4	0.004	0.1262	2.42			0	2	538.35767	66.58	NIOP_Eset1_031019_Frac6.raw
105990532																					hIYAISSAALSASYk	N-Term(iTRAQ4plex); K15(iTRAQ4plex)	Unique	2.533E4	3.686E4	2.808E4	3.738E4	0.002	0.09622	1.46			0	4	468.26270	49.88	EV_set1_060319_050219_Frac4.raw
105990532																					sPAFTDLHLR	N-Term(iTRAQ4plex)	Unique	2.977E4	5.190E4	3.293E4	3.688E4	0.006	0.152	2.67			0	3	434.24203	46.15	EV_set1_060319_050219_Frac3.raw
105990532																					fIIPSPk	N-Term(iTRAQ4plex); K7(iTRAQ4plex)	Unique	1.641E4	9.427E4	2.466E4	3.665E4	0.009	0.1845	2.24			0	3	363.90073	52.78	EV_set1_060319_050219_Frac4.raw
105990532																					sISAALEHk	N-Term(iTRAQ4plex); K9(iTRAQ4plex)	Unique	2.111E4	7.045E4	2.954E4	3.664E4	0	0.01069	3.48			0	3	415.24542	33.61	EV_set1_060319_050219_Frac3.raw
105990532																					kLTISEQNIQR	N-Term(iTRAQ4plex); K1(iTRAQ4plex)	Unique	1.999E4	1.072E5	3.495E4	3.593E4	0	0.00011	5.06			1	3	539.98883	32.42	EV_set1_060319_050219_Frac5.raw
105990532																					nIFNFk	N-Term(iTRAQ4plex); K6(iTRAQ4plex)	Unique	1.039E4	7.549E4	2.111E4	3.586E4	0.007	0.1053		18	1.42894	0	3	357.54517	54.56	EV_set1_060319_050219_Frac3.raw
105990532																					fPEVDVLTk	N-Term(iTRAQ4plex); K9(iTRAQ4plex)	Unique	1.665E4	6.989E4	2.916E4	3.490E4	0	0.01724	2.96			0	2	668.39026	62.48	EV_set1_060319_050219_Frac3.raw
105990532																					lSNVLQQVk	N-Term(iTRAQ4plex); K9(iTRAQ4plex)	Unique	2.912E4	3.792E4	2.599E4	3.419E4	0.002	0.07351	2.53			0	2	658.90936	48.37	NIOP_Eset1_031019_Frac2.raw
105990532																					aNLFNk	N-Term(iTRAQ4plex); K6(iTRAQ4plex)	Unique	2.071E4	6.205E4	2.280E4	3.397E4	0.009	0.1329		39	0.01212	0	2	497.79807	35.59	EV_set1_060319_050219_Frac4.raw
105990532																					sISAALEHk	N-Term(iTRAQ4plex); K9(iTRAQ4plex)	Unique	2.614E4	5.180E4	2.757E4	3.357E4	0	0.031	2.92			0	3	415.24548	33.63	EV_set1_060319_050219_Frac3.raw
105990532																					hIYAISSAALSASYk	N-Term(iTRAQ4plex); K15(iTRAQ4plex)	Unique	2.097E4	3.960E4	2.160E4	3.316E4	0	0.02228	2.14			0	4	468.26270	49.87	EV_set1_060319_050219_Frac4.raw
105990532																					vLVDHFGYTk	N-Term(iTRAQ4plex); K10(iTRAQ4plex)	Unique	1.692E4	8.275E4	4.474E4	3.312E4	0	0.02774	3.11			0	3	489.61252	38.70	NIOP_Eset1_031019_Frac6.raw
105990532																					sVGFHLPSR	N-Term(iTRAQ4plex)	Unique	1.140E4	8.728E4	3.176E4	3.306E4	0.001	0.04629	2.76			0	3	381.88409	29.41	NIOP_Eset1_031019_Frac6.raw
105990532																					fIIPSPk	N-Term(iTRAQ4plex); K7(iTRAQ4plex)	Unique	1.563E4	9.011E4	3.230E4	3.288E4	0.004	0.1248	2.40			0	2	545.34778	52.62	EV_set1_060319_050219_Frac5.raw
105990532																					sVGFHLPSR	N-Term(iTRAQ4plex)	Unique	1.962E4	1.150E5	2.817E4	3.178E4	0.001	0.05207	2.58			0	3	381.88388	29.76	NIOP_Eset1_031019_Frac6.raw
105990532																					lHVAGNLk	N-Term(iTRAQ4plex); K8(iTRAQ4plex)	Unique	5.983E3	6.193E4	2.222E4	3.173E4	0.002	0.07451	2.14			0	2	570.35913	18.76	NIOP_Eset1_031019_Frac6.raw
105990532																					iISDYHQQFR	N-Term(iTRAQ4plex)	Unique	1.644E4	6.578E4	2.769E4	3.158E4	0	0.01544	3.52			0	3	484.25641	38.24	EV_set1_060319_050219_Frac5.raw
105990532																					sPAFTDLHLR	N-Term(iTRAQ4plex)	Unique	1.398E4	9.014E4	1.968E4	3.103E4	0	0.01807	4.14			0	3	434.24240	45.46	EV_set1_060319_050219_Frac5.raw
105990532																					vLVDHFGYTk	N-Term(iTRAQ4plex); K10(iTRAQ4plex)	Unique	1.173E4	5.421E4	2.453E4	3.079E4	0	0.0103	3.06			0	3	489.61261	43.59	EV_set1_060319_050219_Frac5.raw
105990532																					iPSVQINFk	N-Term(iTRAQ4plex); K9(iTRAQ4plex)	Unique	1.568E4	4.980E4	2.737E4	3.052E4	0.001	0.05515	2.48			0	2	667.40674	61.25	EV_set1_060319_050219_Frac3.raw
105990532																					sFDYHQFVDETNDk	N-Term(iTRAQ4plex); K14(iTRAQ4plex)	Unique	8.802E3	5.337E4	3.001E4	3.048E4	0	0.00245	3.30			0	3	678.32037	51.38	EV_set1_060319_050219_Frac5.raw
105990532																					vLVDHFGYTk	N-Term(iTRAQ4plex); K10(iTRAQ4plex)	Unique	1.930E4	5.635E4	2.979E4	3.029E4	0	0.03652	2.38			0	3	489.61227	43.55	EV_set1_060319_050219_Frac4.raw
105990532																					vLVDHFGYTk	N-Term(iTRAQ4plex); K10(iTRAQ4plex)	Unique	1.580E4	6.616E4	3.237E4	2.993E4	0	0.00859	3.42			0	3	489.61230	39.05	NIOP_Eset1_031019_Frac6.raw
105990532																					fIIPSPk	N-Term(iTRAQ4plex); K7(iTRAQ4plex)	Unique	1.745E4	4.485E4	2.627E4	2.984E4	0.004	0.1275	2.35			0	2	545.34833	47.04	NIOP_Eset1_031019_Frac6.raw
105990532																					nLLVALk	N-Term(iTRAQ4plex); K7(iTRAQ4plex)	Unique	2.674E4	4.041E4	1.818E4	2.934E4	0.004	0.1154	2.41			0	2	529.86517	57.07	EV_set1_060319_050219_Frac3.raw
105990532																					iPSVQINFk	N-Term(iTRAQ4plex); K9(iTRAQ4plex)	Unique	1.548E4	7.500E4	2.089E4	2.925E4	0.004	0.1321	2.69			0	3	445.27362	61.36	EV_set1_060319_050219_Frac3.raw
105990532																					vLVDHFGYTk	N-Term(iTRAQ4plex); K10(iTRAQ4plex)	Unique	1.751E4	6.008E4	2.484E4	2.901E4	0	0.03187	2.74			0	3	489.61252	43.57	EV_set1_060319_050219_Frac5.raw
105990532																					lVELAHQYk	N-Term(iTRAQ4plex); K9(iTRAQ4plex)	Unique	1.210E4	5.657E4	2.248E4	2.798E4	0	0.03439	2.94			0	3	463.60876	41.08	EV_set1_060319_050219_Frac5.raw
105990532																					iISDYHQQFR	N-Term(iTRAQ4plex)	Unique	1.865E4	2.935E4	2.174E4	2.783E4	0.005	0.1492	1.52			0	3	484.25665	38.13	EV_set1_060319_050219_Frac4.raw
105990532																					aTGVLYDYVNk	N-Term(iTRAQ4plex); K11(iTRAQ4plex)	Unique	1.584E4	3.083E4	1.817E4	2.732E4	0	0.02167	2.58			0	3	510.95087	54.19	EV_set1_060319_050219_Frac3.raw
105990532																					iNPLALk	N-Term(iTRAQ4plex); K7(iTRAQ4plex)	Unique	8.321E3	4.243E4	1.764E4	2.634E4	0.004	0.1189	1.93			0	3	352.90457	51.84	EV_set1_060319_050219_Frac4.raw
105990532																					sVGFHLPSR	N-Term(iTRAQ4plex)	Unique	1.759E4	5.820E4	2.151E4	2.632E4	0.001	0.06013	2.46			0	3	381.88452	39.01	EV_set1_060319_050219_Frac3.raw

105990532																					hVAEAIck	N-Term(iTRAQ4plex); C7(Methylthio); K8(iTRAQ4plex)	Unique	1.209E4	4.552E4	1.815E4	2.607E4	0	0.01099	2.40			0	3	402.21802	32.92	EV_set1_060319_050219_Frac5.raw
105990532																					lVELAHQYk	N-Term(iTRAQ4plex); K9(iTRAQ4plex)	Unique	8.063E3	5.383E4	1.335E4	2.579E4	0	0.01783	2.89			0	3	463.60858	41.06	EV_set1_060319_050219_Frac5.raw
105990532																					fIIPGLk	N-Term(iTRAQ4plex); K7(iTRAQ4plex)	Unique	1.189E4	6.702E4	1.890E4	2.566E4	0.002	0.0832	2.06			0	3	359.24207	67.04	EV_set1_060319_050219_Frac4.raw
105990532																					hIYAISSAALSASYk	N-Term(iTRAQ4plex); K15(iTRAQ4plex)	Unique	1.774E4	3.882E4	1.758E4	2.544E4	0	0.01066	2.31			0	4	468.26270	49.87	EV_set1_060319_050219_Frac4.raw
105990532																					hIYAISSAALSASYk	N-Term(iTRAQ4plex); K15(iTRAQ4plex)	Unique	1.697E4	3.974E4	3.077E4	2.531E4	0	0.01108	2.72			0	3	624.01416	49.78	EV_set1_060319_050219_Frac5.raw
105990532																					fIIPSPk	N-Term(iTRAQ4plex); K7(iTRAQ4plex)	Unique	1.457E4	6.464E4	2.193E4	2.530E4	0.009	0.1873	1.68			0	2	545.34814	53.14	EV_set1_060319_050219_Frac4.raw
105990532																					vLVDHFGYTk	N-Term(iTRAQ4plex); K10(iTRAQ4plex)	Unique	1.194E4	5.372E4	2.223E4	2.518E4	0	0.02316	2.84			0	3	489.61267	43.64	EV_set1_060319_050219_Frac5.raw
105990532																					iPSVQINFk	N-Term(iTRAQ4plex); K9(iTRAQ4plex)	Unique	8.761E3	6.798E4	1.887E4	2.501E4	0.004	0.1208	2.62			0	3	445.27347	60.96	EV_set1_060319_050219_Frac4.raw
105990532																					lVELAHQYk	N-Term(iTRAQ4plex); K9(iTRAQ4plex)	Unique	1.834E4	4.768E4	2.691E4	2.497E4	0	0.00517	3.55			0	2	694.90991	36.03	NIOP_Eset1_031019_Frac6.raw
105990532																					vLVDHFGYTk	N-Term(iTRAQ4plex); K10(iTRAQ4plex)	Unique	1.750E4	3.909E4	2.398E4	2.460E4	0	0.02424	2.66			0	3	489.61227	43.55	EV_set1_060319_050219_Frac4.raw
105990532																					fPEVDVLTk	N-Term(iTRAQ4plex); K9(iTRAQ4plex)	Unique	1.424E4	4.578E4	1.997E4	2.427E4	0.008	0.1743	2.55			0	2	668.39026	62.49	EV_set1_060319_050219_Frac3.raw
105990532																					kLTISEQNIQR	N-Term(iTRAQ4plex); K1(iTRAQ4plex)	Unique	1.292E4	8.500E4	2.161E4	2.411E4	0	8.8E-05	5.03			1	3	539.98889	32.42	EV_set1_060319_050219_Frac5.raw
105990532																					sFDYHQFVDETNDk	N-Term(iTRAQ4plex); K14(iTRAQ4plex)	Unique	7.035E3	3.629E4	2.453E4	2.404E4	0	0.00276	2.65			0	3	678.32037	51.38	EV_set1_060319_050219_Frac5.raw
105990532																					vLVDHFGYTk	N-Term(iTRAQ4plex); K10(iTRAQ4plex)	Unique	1.321E4	4.470E4	2.463E4	2.366E4	0	0.02093	2.69			0	3	489.61234	43.60	EV_set1_060319_050219_Frac4.raw
105990532																					kLTISEQNIQR	N-Term(iTRAQ4plex); K1(iTRAQ4plex)	Unique	9.054E3	5.921E4	2.291E4	2.349E4	0	6.4E-05	5.68			1	3	539.98840	22.97	NIOP_Eset1_031019_Frac6.raw
105990532																					sVGFHLPSR	N-Term(iTRAQ4plex)	Unique	2.050E4	4.797E4	2.418E4	2.343E4	0.001	0.04448	2.64			0	3	381.88428	38.91	EV_set1_060319_050219_Frac3.raw
105990532																					vLVDHFGYTk	N-Term(iTRAQ4plex); K10(iTRAQ4plex)	Unique	1.325E4	6.175E4	2.124E4	2.309E4	0	0.00122	3.73			0	2	733.91455	43.65	EV_set1_060319_050219_Frac5.raw
105990532																					sVGFHLPSR	N-Term(iTRAQ4plex)	Unique	1.375E4	7.471E4	1.813E4	2.235E4	0	0.02208	3.20			0	3	381.88409	38.01	EV_set1_060319_050219_Frac5.raw
105990532																					sPAFTDLHLR	N-Term(iTRAQ4plex)	Unique	1.120E4	6.300E4	1.980E4	2.204E4	0	0.03572	3.80			0	3	434.24240	45.46	EV_set1_060319_050219_Frac5.raw
105990532																					lPYTIITTPPLk	N-Term(iTRAQ4plex); K12(iTRAQ4plex)	Unique	9.736E3	6.977E4	1.610E4	2.204E4	0	0.00594	3.04			0	3	549.01062	73.69	EV_set1_060319_050219_Frac3.raw
105990532																					sVGFHLPSR	N-Term(iTRAQ4plex)	Unique	1.141E4	4.687E4	1.535E4	2.178E4	0	0.01917	3.29			0	2	572.32269	37.63	EV_set1_060319_050219_Frac5.raw
105990532																					fIIPSPk	N-Term(iTRAQ4plex); K7(iTRAQ4plex)	Unique	7.301E3	4.539E4	1.935E4	2.153E4	0.009	0.186	2.02			0	3	363.90085	52.66	EV_set1_060319_050219_Frac5.raw
105990532																					hINIDQFVR	N-Term(iTRAQ4plex)	Unique	1.320E4	5.752E4	1.530E4	2.149E4	0	0.01722	3.23			0	3	429.24240	45.77	EV_set1_060319_050219_Frac5.raw
105990532																					lVELAHQYk	N-Term(iTRAQ4plex); K9(iTRAQ4plex)	Unique	1.213E4	5.163E4	1.743E4	2.130E4	0	0.00283	3.68			0	2	694.90948	41.14	EV_set1_060319_050219_Frac5.raw
105990532																					vLVDHFGYTk	N-Term(iTRAQ4plex); K10(iTRAQ4plex)	Unique	1.721E4	2.620E4	2.447E4	2.116E4	0.002	0.08095	2.12			0	3	489.61301	38.51	NIOP_Eset1_031019_Frac6.raw
105990532																					sPAFTDLHLR	N-Term(iTRAQ4plex)	Unique	1.339E4	6.247E4	1.663E4	2.112E4	0	0.00871	4.01			0	3	434.24200	45.49	EV_set1_060319_050219_Frac4.raw
105990532																					sISAALEHk	N-Term(iTRAQ4plex); K9(iTRAQ4plex)	Unique	8.663E3	5.872E4	1.354E4	2.097E4	0	0.03876	2.78			0	3	415.24539	30.00	EV_set1_060319_050219_Frac4.raw
105990532																					yGMVAQVTQTLk	N-Term(iTRAQ4plex); K12(iTRAQ4plex)	Unique	7.268E3	3.636E4	1.581E4	2.093E4	0	0.00176	3.23			0	3	542.97510	60.14	EV_set1_060319_050219_Frac3.raw
105990532																					fIIPGLk	N-Term(iTRAQ4plex); K7(iTRAQ4plex)	Unique	8.617E3	5.870E4	1.838E4	2.063E4	0.004	0.1307	2.31			0	3	359.24109	66.83	EV_set1_060319_050219_Frac5.raw
105990532																					fIIPGLk	N-Term(iTRAQ4plex); K7(iTRAQ4plex)	Unique	8.469E3	3.832E4	1.334E4	2.060E4	0.004	0.1332	1.99			0	2	538.35828	67.33	EV_set1_060319_050219_Frac3.raw
105990532																					lPYTIITTPPLk	N-Term(iTRAQ4plex); K12(iTRAQ4plex)	Unique	9.656E3	5.672E4	1.290E4	2.016E4	0	0.03614	2.42			0	2	823.01135	73.88	EV_set1_060319_050219_Frac3.raw
105990532																					hINIDQFVR	N-Term(iTRAQ4plex)	Unique	1.245E4	4.953E4	2.128E4	2.007E4	0	0.02237	2.98			0	3	429.24240	45.78	EV_set1_060319_050219_Frac5.raw
105990532																					lVELAHQYk	N-Term(iTRAQ4plex); K9(iTRAQ4plex)	Unique	1.013E4	3.354E4	1.477E4	1.992E4	0.001	0.05907	2.17			0	3	463.60806	41.03	EV_set1_060319_050219_Frac4.raw
105990532																					hIYAISSAALSASYk	N-Term(iTRAQ4plex); K15(iTRAQ4plex)	Unique	7.574E3	4.682E4	1.043E4	1.986E4	0	0.00299	3.85			0	4	468.26254	49.85	EV_set1_060319_050219_Frac5.raw
105990532																					hIYAISSAALSASYk	N-Term(iTRAQ4plex); K15(iTRAQ4plex)	Unique	1.180E4	2.057E4	2.090E4	1.974E4	0	0.01447	3.15			0	3	624.01434	49.81	EV_set1_060319_050219_Frac5.raw
105990532																					sVGFHLPSR	N-Term(iTRAQ4plex)	Unique	1.623E4	3.267E4	1.741E4	1.971E4	0.004	0.1233	2.31			0	3	381.88437	39.14	EV_set1_060319_050219_Frac3.raw
105990532																					qHLFVk	N-Term(iTRAQ4plex); K6(iTRAQ4plex)	Unique	3.510E3	4.037E4	1.876E4	1.926E4	0.008	0.1246		16	0.88442	0	3	353.88879	17.05	NIOP_Eset1_031019_Frac6.raw
105990532																					lVELAHQYk	N-Term(iTRAQ4plex); K9(iTRAQ4plex)	Unique	9.723E3	2.869E4	1.696E4	1.861E4	0.001	0.06305	2.48			0	3	463.60876	41.09	EV_set1_060319_050219_Frac5.raw
105990532																					iISDYHQQFR	N-Term(iTRAQ4plex)	Unique	1.315E4	5.198E4	1.616E4	1.858E4	0	0.00711	3.22			0	2	725.88104	38.27	EV_set1_060319_050219_Frac5.raw
105990532																					fIIPGLk	N-Term(iTRAQ4plex); K7(iTRAQ4plex)	Unique	6.716E3	4.257E4	1.055E4	1.850E4	0.007	0.1705	1.70			0	2	538.35748	66.24	NIOP_Eset1_031019_Frac6.raw

105990532																					kHVAEAIck	N-Term(iTRAQ4plex); K1(iTRAQ4plex); C8(Methylthio); K9(iTRAQ4plex)	Unique	2.783E3	3.497E4	1.756E4	1.843E4	0	0.00905	2.99			1	3	492.95065	16.95	NIOP_Eset1_031019_Frac6.raw
105990532																					lLSGGNTLHLVSTTk	N-Term(iTRAQ4plex); K15(iTRAQ4plex)	Unique	8.791E3	3.928E4	1.776E4	1.824E4	0	0.0001	3.85			0	3	610.36176	50.87	EV_set1_060319_050219_Frac5.raw
105990532																					yGMVAQVTQTLk	N-Term(iTRAQ4plex); K12(iTRAQ4plex)	Unique	9.649E3	3.128E4	1.669E4	1.816E4	0	0.00632	2.81			0	3	542.97510	60.15	EV_set1_060319_050219_Frac3.raw
105990532																					sPAFTDLHLR	N-Term(iTRAQ4plex)	Unique	6.806E3	4.467E4	1.591E4	1.781E4	0	0.02946	3.57			0	3	434.24216	45.51	EV_set1_060319_050219_Frac4.raw
105990532																					hIYAISSAALSASYk	N-Term(iTRAQ4plex); K15(iTRAQ4plex)	Unique	1.132E4	2.080E4	1.626E4	1.780E4	0.001	0.03973	2.25			0	3	624.01447	49.79	EV_set1_060319_050219_Frac4.raw
105990532																					lLSGGNTLHLVSTTk	N-Term(iTRAQ4plex); K15(iTRAQ4plex)	Unique	1.140E4	5.352E4	1.669E4	1.768E4	0	0.0003	3.90			0	4	458.02319	50.98	EV_set1_060319_050219_Frac5.raw
105990532																					lLSGGNTLHLVSTTk	N-Term(iTRAQ4plex); K15(iTRAQ4plex)	Unique	1.148E4	2.978E4	1.488E4	1.737E4	0	0.01389	3.31			0	3	610.36194	50.93	EV_set1_060319_050219_Frac5.raw
105990532																					iISDYHQQFR	N-Term(iTRAQ4plex)	Unique	8.586E3	3.234E4	1.471E4	1.717E4	0.003	0.1102	2.34			0	2	725.88086	38.31	EV_set1_060319_050219_Frac5.raw
105990532																					lVELAHqYk	N-Term(iTRAQ4plex); Q7(Deamidated); K9(iTRAQ4plex)	Unique	4.900E3	2.252E4	8.760E3	1.699E4	0	0.02648	2.28			0	3	463.93692	43.46	EV_set1_060319_050219_Frac5.raw
105990532																					tGISPLALIk	N-Term(iTRAQ4plex); K10(iTRAQ4plex)	Unique	1.131E4	4.814E4	1.941E4	1.682E4	0	0.01391	3.24			0	2	650.92450	67.09	EV_set1_060319_050219_Frac3.raw
105990532																					hIYAISSAALSASYk	N-Term(iTRAQ4plex); K15(iTRAQ4plex)	Unique	4.557E3	3.690E4	1.131E4	1.672E4	0	0.00185	4.03			0	4	468.26254	49.86	EV_set1_060319_050219_Frac5.raw
105990532																					sVGFHLPSR	N-Term(iTRAQ4plex)	Unique	7.343E3	5.129E4	1.952E4	1.668E4	0.003	0.1099	2.41			0	3	381.88397	37.56	EV_set1_060319_050219_Frac5.raw
105990532																					tGISPLALIk	N-Term(iTRAQ4plex); K10(iTRAQ4plex)	Unique	9.043E3	3.201E4	1.907E4	1.630E4	0	0.03838	3.15			0	2	650.92450	67.09	EV_set1_060319_050219_Frac3.raw
105990532																					iNDVLEHVk	N-Term(iTRAQ4plex); K9(iTRAQ4plex)	Unique	7.329E3	2.318E4	9.393E3	1.615E4	0	0.02116	2.74			0	3	452.26816	41.48	NIOP_Eset1_031019_Frac6.raw
105990532																					nIFNFk	N-Term(iTRAQ4plex); K6(iTRAQ4plex)	Unique	4.306E3	3.265E4	1.150E4	1.609E4	0.006	0.1001		15	2.67348	0	3	357.54483	54.23	EV_set1_060319_050219_Frac4.raw
105990532																					lVELAHQYk	N-Term(iTRAQ4plex); K9(iTRAQ4plex)	Unique	6.897E3	2.416E4	1.116E4	1.577E4	0	0.00292	2.88			0	2	694.91016	35.85	NIOP_Eset1_031019_Frac6.raw
105990532																					iPSVQINFk	N-Term(iTRAQ4plex); K9(iTRAQ4plex)	Unique	9.269E3	3.555E4	1.766E4	1.576E4	0.002	0.06999	2.85			0	3	445.27362	61.36	EV_set1_060319_050219_Frac3.raw
105990532																					vLVDHFGYTk	N-Term(iTRAQ4plex); K10(iTRAQ4plex)	Unique	8.751E3	3.276E4	1.317E4	1.567E4	0	0.00691	3.03			0	2	733.91455	43.65	EV_set1_060319_050219_Frac5.raw
105990532																					lGNNPVSk	N-Term(iTRAQ4plex); K8(iTRAQ4plex)	Unique	6.582E3	3.194E4	1.387E4	1.560E4	0.009	0.1835	1.93			0	2	558.83362	29.39	EV_set1_060319_050219_Frac3.raw
105990532																					lPYTIITTPPLk	N-Term(iTRAQ4plex); K12(iTRAQ4plex)	Unique	7.307E3	4.262E4	1.218E4	1.560E4	0	0.01918	3.02			0	3	549.01031	73.65	EV_set1_060319_050219_Frac3.raw
105990532																					aTLYALSHAVnNYHk	N-Term(iTRAQ4plex); N11(Deamidated); K15(iTRAQ4plex)	Unique	2.136E4	2.282E4	2.237E4	1.526E4	0	0.02476	2.86			0	4	498.51923	48.14	EV_set1_060319_050219_Frac5.raw
105990532																					fIIPGLk	N-Term(iTRAQ4plex); K7(iTRAQ4plex)	Unique	6.852E3	3.060E4	9.754E3	1.502E4	0.003	0.1079	2.16			0	2	538.35803	66.73	EV_set1_060319_050219_Frac5.raw
105990532																					sFDYHQFVDETNDk	N-Term(iTRAQ4plex); K14(iTRAQ4plex)	Unique	6.404E3	2.245E4	1.679E4	1.473E4	0	0.03524	1.78			0	3	678.32037	51.38	EV_set1_060319_050219_Frac5.raw
105990532																					hINIDQFVR	N-Term(iTRAQ4plex)	Unique	7.195E3	3.197E4	1.244E4	1.459E4	0	0.03237	2.79			0	3	429.24225	45.77	EV_set1_060319_050219_Frac4.raw
105990532																					sPAFTDLHLR	N-Term(iTRAQ4plex)	Unique	9.033E3	4.060E4	1.313E4	1.452E4	0.003	0.0992	3.21			0	3	434.24219	45.50	EV_set1_060319_050219_Frac5.raw
105990532																					kHVAEAIck	N-Term(iTRAQ4plex); K1(iTRAQ4plex); C8(Methylthio); K9(iTRAQ4plex)	Unique	3.360E3	3.242E4	1.644E4	1.451E4	0.002	0.07702	3.10			1	3	492.94986	16.77	NIOP_Eset1_031019_Frac6.raw
105990532																					kGNVATEISTER	N-Term(iTRAQ4plex); K1(iTRAQ4plex)	Unique	1.037E4	1.682E4	1.933E4	1.449E4	0.002	0.08747	3.26			1	3	531.63153	15.46	NIOP_Eset1_031019_Frac6.raw
105990532																					hIYAISSAALSASYk	N-Term(iTRAQ4plex); K15(iTRAQ4plex)	Unique	6.625E3	2.734E4	7.940E3	1.441E4	0	0.00049	3.84			0	3	624.01416	49.78	EV_set1_060319_050219_Frac5.raw
105990532																					hVAEAIck	N-Term(iTRAQ4plex); C7(Methylthio); K8(iTRAQ4plex)	Unique	4.934E3	2.430E4	1.007E4	1.431E4	0.006	0.09382		41	0.00701	0	2	602.82391	32.98	EV_set1_060319_050219_Frac5.raw
105990532																					hVAEAIck	N-Term(iTRAQ4plex); C7(Methylthio); K8(iTRAQ4plex)	Unique	7.406E3	3.120E4	9.768E3	1.412E4	0.001	0.054	2.01			0	3	402.21848	32.94	EV_set1_060319_050219_Frac5.raw
105990532																					iPSVQINFk	N-Term(iTRAQ4plex); K9(iTRAQ4plex)	Unique	6.340E3	3.123E4	1.037E4	1.398E4	0.006	0.1617	2.22			0	2	667.40576	60.80	EV_set1_060319_050219_Frac4.raw
105990532																					fIIPSPk	N-Term(iTRAQ4plex); K7(iTRAQ4plex)	Unique	7.862E3	3.131E4	1.649E4	1.398E4	0.007	0.1095		35	0.02084	0	2	545.34808	52.63	EV_set1_060319_050219_Frac4.raw
105990532																					fIIPSPk	N-Term(iTRAQ4plex); K7(iTRAQ4plex)	Unique	2.634E3	2.838E4	1.340E4	1.396E4	0.009	0.1293		10	5.79271	0	3	363.90079	52.74	EV_set1_060319_050219_Frac5.raw
105990532																					hINIDQFVR	N-Term(iTRAQ4plex)	Unique	9.676E3	2.574E4	1.275E4	1.369E4	0.001	0.05442	2.61			0	3	429.24219	45.81	EV_set1_060319_050219_Frac5.raw
105990532																					vSTAFVYTk	N-Term(iTRAQ4plex); K9(iTRAQ4plex)	Unique	9.634E3	2.760E4	1.257E4	1.349E4	0	0.02192	2.67			0	2	652.37982	39.39	NIOP_Eset1_031019_Frac2.raw
105990532																					hIYAISSAALSASYk	N-Term(iTRAQ4plex); K15(iTRAQ4plex)	Unique	9.374E3	1.433E4	1.164E4	1.345E4	0.001	0.06735	2.05			0	3	624.01447	49.83	EV_set1_060319_050219_Frac4.raw
105990532																					yGMVAQVTQTLk	N-Term(iTRAQ4plex); K12(iTRAQ4plex)	Unique	8.803E3	1.872E4	1.332E4	1.332E4	0.001	0.05698	2.34			0	3	542.97540	60.16	EV_set1_060319_050219_Frac3.raw
105990532																					lVELAHQYk	N-Term(iTRAQ4plex); K9(iTRAQ4plex)	Unique	9.094E3	3.108E4	1.701E4	1.331E4	0	0.01487	3.31			0	2	694.90948	41.15	EV_set1_060319_050219_Frac5.raw

105990532																					eQHLFLPFSYk	N-Term(iTRAQ4plex); K11(iTRAQ4plex)	Unique	2.005E4	2.790E4	2.212E4	1.324E4	0	0.00679	2.69			0	3	566.31439	62.30	EV_set1_060319_050219_Frac3.raw
105990532																					iPSVQINFk	N-Term(iTRAQ4plex); K9(iTRAQ4plex)	Unique	5.160E3	3.528E4	1.176E4	1.321E4	0.004	0.1287	2.53			0	3	445.27347	60.96	EV_set1_060319_050219_Frac4.raw
105990532																					yGMVAQVTQTLk	N-Term(iTRAQ4plex); K12(iTRAQ4plex)	Unique	8.477E3	1.082E4	9.814E3	1.292E4	0.004	0.1156	1.84			0	3	542.97540	60.18	EV_set1_060319_050219_Frac3.raw
105990532																					vSTAFVYTk	N-Term(iTRAQ4plex); K9(iTRAQ4plex)	Unique	3.869E3	2.465E4	8.990E3	1.288E4	0	0.0332	2.71			0	2	652.37933	39.08	NIOP_Eset1_031019_Frac2.raw
105990532																					vTQEFHmk	N-Term(iTRAQ4plex); M7(Oxidation); K8(iTRAQ4plex)	Unique	7.544E3	1.920E4	8.964E3	1.255E4	0.009	0.1338		24	0.42126	0	3	441.90317	20.21	EV_set1_060319_050219_Frac4.raw
105990532																					lVELAHqYk	N-Term(iTRAQ4plex); Q7(Deamidated); K9(iTRAQ4plex)	Unique	7.003E3	2.219E4	1.008E4	1.252E4	0	0.02281	2.49			0	3	463.93692	43.44	EV_set1_060319_050219_Frac5.raw
105990532																					aTLYALSHAVnNYHk	N-Term(iTRAQ4plex); N11(Deamidated); K15(iTRAQ4plex)	Unique	1.410E4	1.964E4	1.610E4	1.244E4	0	0.03082	2.87			0	4	498.51923	48.13	EV_set1_060319_050219_Frac5.raw
105990532																					hINIDQFVR	N-Term(iTRAQ4plex)	Unique	7.849E3	2.578E4	1.350E4	1.236E4	0	0.03117	1.95			0	3	429.24225	41.73	NIOP_Eset1_031019_Frac6.raw
105990532																					fIIPGLk	N-Term(iTRAQ4plex); K7(iTRAQ4plex)	Unique	5.025E3	3.026E4	1.178E4	1.192E4	0.002	0.07576	2.02			0	2	538.35840	66.93	EV_set1_060319_050219_Frac4.raw
105990532																					sPAFTDLHLR	N-Term(iTRAQ4plex)	Unique	7.684E3	2.438E4	1.266E4	1.191E4	0.002	0.07913	2.87			0	3	434.24216	45.52	EV_set1_060319_050219_Frac4.raw
105990532																					sISAALEHk	N-Term(iTRAQ4plex); K9(iTRAQ4plex)	Unique	4.963E3	2.031E4	1.004E4	1.189E4	0.001	0.05084	2.47			0	3	415.24548	32.34	EV_set1_060319_050219_Frac5.raw
105990532																					sISAALEHk	N-Term(iTRAQ4plex); K9(iTRAQ4plex)	Unique	4.733E3	2.036E4	8.472E3	1.185E4	0.001	0.04241	2.30			0	3	415.24533	32.31	EV_set1_060319_050219_Frac5.raw
105990532																					aTLYALSHAVnNYHk	N-Term(iTRAQ4plex); N11(Deamidated); K15(iTRAQ4plex)	Unique	6.839E3	2.449E4	8.175E3	1.172E4	0	0.00183	3.80			0	4	498.51959	43.53	EV_set1_060319_050219_Frac5.raw
105990532																					kGNVATEISTER	N-Term(iTRAQ4plex); K1(iTRAQ4plex)	Unique	5.122E3	1.290E4	8.528E3	1.166E4	0	0.01037	3.48			1	3	531.63245	28.90	EV_set1_060319_050219_Frac3.raw
105990532																					lLSGGNTLHLVSTTk	N-Term(iTRAQ4plex); K15(iTRAQ4plex)	Unique	5.284E3	3.279E4	1.361E4	1.160E4	0	0.00072	3.70			0	4	458.02319	50.98	EV_set1_060319_050219_Frac5.raw
105990532																					fIIPGLk	N-Term(iTRAQ4plex); K7(iTRAQ4plex)	Unique	3.804E3	2.866E4	1.027E4	1.160E4	0.001	0.05988	2.14			0	3	359.24207	67.04	EV_set1_060319_050219_Frac4.raw
105990532																					lPYTIITTPPLk	N-Term(iTRAQ4plex); K12(iTRAQ4plex)	Unique	6.803E3	3.560E4	8.099E3	1.141E4	0	0.00448	2.85			0	3	549.01031	73.38	EV_set1_060319_050219_Frac5.raw
105990532																					aTLYALSHAVnNYHk	N-Term(iTRAQ4plex); N11(Deamidated); K15(iTRAQ4plex)	Unique	5.527E3	2.039E4	8.998E3	1.130E4	0	0.00129	3.81			0	3	664.35583	43.54	EV_set1_060319_050219_Frac5.raw
105990532																					iNDVLEHVk	N-Term(iTRAQ4plex); K9(iTRAQ4plex)	Unique	8.223E3	1.127E4	8.043E3	1.126E4	0.004	0.1135	1.77			0	3	452.26871	41.30	NIOP_Eset1_031019_Frac6.raw
105990532																					yGMVAQVTQTLk	N-Term(iTRAQ4plex); K12(iTRAQ4plex)	Unique	6.892E3	2.303E4	7.764E3	1.120E4	0	0.01698	2.32			0	2	813.95953	60.19	EV_set1_060319_050219_Frac3.raw
105990532																					hINIDQFVR	N-Term(iTRAQ4plex)	Unique	5.229E3	2.319E4	8.380E3	1.107E4	0	0.01669	2.50			0	3	429.24188	45.74	EV_set1_060319_050219_Frac4.raw
105990532																					hIQNIDIQHLAGk	N-Term(iTRAQ4plex); K13(iTRAQ4plex)	Unique	1.502E4	1.646E4	6.612E3	1.095E4	0	0.00352	2.60			0	3	592.34235	39.88	EV_set1_060319_050219_Frac5.raw
105990532																					eQHLFLPFSYk	N-Term(iTRAQ4plex); K11(iTRAQ4plex)	Unique	1.678E4	2.114E4	1.851E4	1.084E4	0	0.01284	2.57			0	3	566.31500	62.34	EV_set1_060319_050219_Frac3.raw
105990532																					lLSGGNTLHLVSTTk	N-Term(iTRAQ4plex); K15(iTRAQ4plex)	Unique	6.666E3	2.839E4	1.062E4	1.083E4	0	0.00386	3.90			0	3	610.36176	50.87	EV_set1_060319_050219_Frac5.raw
105990532																					aLVEQGFTVPEIk	N-Term(iTRAQ4plex); K13(iTRAQ4plex)	Unique	8.124E3	2.346E4	9.294E3	1.076E4	0	0.00366	3.08			0	3	573.66882	66.46	EV_set1_060319_050219_Frac3.raw
105990532																					kLTISEQNIQR	N-Term(iTRAQ4plex); K1(iTRAQ4plex)	Unique	6.181E3	1.506E4	1.248E4	1.072E4	0.007	0.163	2.34			1	3	539.98834	30.39	EV_set1_060319_050219_Frac4.raw
105990532																					aLVEQGFTVPEIk	N-Term(iTRAQ4plex); K13(iTRAQ4plex)	Unique	4.911E3	1.506E4	3.714E3	1.054E4	0	0.00361	2.83			0	3	573.66852	66.48	EV_set1_060319_050219_Frac3.raw
105990532																					kLTISEQNIQR	N-Term(iTRAQ4plex); K1(iTRAQ4plex)	Unique	7.615E3	1.185E4	7.650E3	1.040E4	0.005	0.08281		23	0.34087	1	3	539.98828	30.40	EV_set1_060319_050219_Frac4.raw
105990532																					eQHLFLPFSYk	N-Term(iTRAQ4plex); K11(iTRAQ4plex)	Unique	1.636E4	1.519E4	1.611E4	9.643E3	0.006	0.1545	1.27			0	3	566.31500	62.35	EV_set1_060319_050219_Frac3.raw
105990532																					hIYAISSAALSASYk	N-Term(iTRAQ4plex); K15(iTRAQ4plex)	Unique	4.745E3	1.928E4	6.280E3	9.575E3	0	0.00324	3.45			0	4	468.26254	49.86	EV_set1_060319_050219_Frac5.raw
105990532																					tGISPLALIk	N-Term(iTRAQ4plex); K10(iTRAQ4plex)	Unique	8.030E3	2.251E4	1.717E4	9.463E3	0.008	0.173	2.23			0	2	650.92438	67.13	EV_set1_060319_050219_Frac3.raw
105990532																					aTLYALSHAVnNYHk	N-Term(iTRAQ4plex); N11(Deamidated); K15(iTRAQ4plex)	Unique	5.771E3	1.430E4	8.718E3	9.390E3	0	0.00483	3.17			0	4	498.51971	43.47	EV_set1_060319_050219_Frac5.raw
105990532																					tGISPLALIk	N-Term(iTRAQ4plex); K10(iTRAQ4plex)	Unique	4.748E3	1.443E4	6.681E3	9.389E3	0.009	0.1288		48	0.00021	0	2	650.92358	88.09	NIOP_Eset1_031019_Frac2.raw
105990532																					lLSGGNTLHLVSTTk	N-Term(iTRAQ4plex); K15(iTRAQ4plex)	Unique	5.786E3	1.828E4	9.004E3	9.104E3	0	0.03695	3.00			0	3	610.36176	50.88	EV_set1_060319_050219_Frac5.raw
105990532																					hVAEAIck	N-Term(iTRAQ4plex); C7(Methylthio); K8(iTRAQ4plex)	Unique	5.756E3	1.726E4	6.544E3	9.068E3	0.001	0.05359	1.58			0	3	402.21802	30.50	EV_set1_060319_050219_Frac4.raw
105990532																					sLHMYANR	N-Term(iTRAQ4plex)	Unique	4.911E3	2.175E4	9.192E3	8.971E3	0.001	0.05598	2.36			0	3	379.19733	28.36	EV_set1_060319_050219_Frac3.raw
105990532																					sVGFHLPSR	N-Term(iTRAQ4plex)	Unique	7.013E3	1.310E4	9.651E3	8.925E3	0.001	0.06528	2.36			0	3	381.88361	29.23	NIOP_Eset1_031019_Frac6.raw
105990532																					lPYTIITTPPLk	N-Term(iTRAQ4plex); K12(iTRAQ4plex)	Unique	3.795E3	2.112E4	5.578E3	8.793E3	0	0.01377	2.76			0	3	549.01031	73.50	EV_set1_060319_050219_Frac4.raw
105990532																					lnGEIQALELPQk	N-Term(iTRAQ4plex); N2(Deamidated); K13(iTRAQ4plex)	Unique	6.998E3	1.074E4	7.128E3	8.777E3	0	0.00835	2.86			0	3	581.33496	66.37	EV_set1_060319_050219_Frac3.raw

105990532																					ILSGGNTLHLVSTTk	N-Term(iTRAQ4plex); K15(iTRAQ4plex)	Unique	4.549E3	1.952E4	6.860E3	8.769E3	0	0.00019	3.86			0	3	610.36212	50.93	EV_set1_060319_050219_Frac4.raw
105990532																					ILSGGNTLHLVSTTk	N-Term(iTRAQ4plex); K15(iTRAQ4plex)	Unique	3.478E3	9.230E3	1.326E4	8.765E3	0.004	0.1158	1.93			0	3	610.36182	50.99	EV_set1_060319_050219_Frac4.raw
105990532																					hINIDQFVR	N-Term(iTRAQ4plex)	Unique	4.586E3	1.429E4	1.360E4	8.754E3	0.002	0.08021	1.53			0	3	429.24216	41.55	NIOP_Eset1_031019_Frac6.raw
105990532																					ILSGGNTLHLVSTTk	N-Term(iTRAQ4plex); K15(iTRAQ4plex)	Unique	4.124E3	2.538E4	8.032E3	8.744E3	0	0.00089	3.13			0	4	458.02301	51.04	EV_set1_060319_050219_Frac4.raw
105990532																					hIQNIDIQHLAGk	N-Term(iTRAQ4plex); K13(iTRAQ4plex)	Unique	8.156E3	1.351E4	8.480E3	8.716E3	0	0.01367	2.72			0	3	592.34235	39.88	EV_set1_060319_050219_Frac5.raw
105990532																					IPYTIITTPPLk	N-Term(iTRAQ4plex); K12(iTRAQ4plex)	Unique	8.916E3	1.379E4	9.884E3	8.635E3	0	0.01942	2.77			0	3	549.01111	88.77	NIOP_Eset1_031019_Frac2.raw
105990532																					aTLYALSHAVNnYHk	N-Term(iTRAQ4plex); N12(Deamidated); K15(iTRAQ4plex)	Unique	1.076E4	9.440E3	8.187E3	8.576E3	0.001	0.04881	2.00			0	4	498.51959	48.20	EV_set1_060319_050219_Frac5.raw
105990532																					kLTISEQNIQR	N-Term(iTRAQ4plex); K1(iTRAQ4plex)	Unique	4.984E3	1.921E4	6.489E3	8.553E3	0	0.01206	3.74			1	3	539.98834	23.14	NIOP_Eset1_031019_Frac6.raw
105990532																					sISAALEHk	N-Term(iTRAQ4plex); K9(iTRAQ4plex)	Unique	4.131E3	1.361E4	5.298E3	8.549E3	0.002	0.08163	2.24			0	3	415.24576	32.36	EV_set1_060319_050219_Frac5.raw
105990532																					vLVDHFGYTk	N-Term(iTRAQ4plex); K10(iTRAQ4plex)	Unique	6.288E3	1.779E4	7.589E3	8.525E3	0	0.019	2.41			0	2	733.91522	43.69	EV_set1_060319_050219_Frac4.raw
105990532																					aTLYALSHAVnNYHk	N-Term(iTRAQ4plex); N11(Deamidated); K15(iTRAQ4plex)	Unique	4.974E3	1.900E4	8.376E3	8.457E3	0	0.00099	3.65			0	4	498.51971	43.48	EV_set1_060319_050219_Frac5.raw
105990532																					sVGFHLPSR	N-Term(iTRAQ4plex)	Unique	5.717E3	2.718E4	1.573E4	8.351E3	0.006	0.1609	1.86			0	3	381.88419	37.60	EV_set1_060319_050219_Frac5.raw
105990532																					IPYTIITTPPLk	N-Term(iTRAQ4plex); K12(iTRAQ4plex)	Unique	3.185E3	2.234E4	5.894E3	8.336E3	0	0.02632	2.66			0	3	549.01031	73.51	EV_set1_060319_050219_Frac4.raw
105990532																					ILSGGNTLHLVSTTk	N-Term(iTRAQ4plex); K15(iTRAQ4plex)	Unique	7.466E3	1.432E4	1.491E4	8.303E3	0.001	0.06483	2.78			0	3	610.36188	50.96	EV_set1_060319_050219_Frac4.raw
105990532																					IPYTIITTPPLk	N-Term(iTRAQ4plex); K12(iTRAQ4plex)	Unique	5.396E3	2.312E4	5.091E3	8.027E3	0	0.01152	2.88			0	3	549.01031	73.41	EV_set1_060319_050219_Frac5.raw
105990532																					fIIPGLk	N-Term(iTRAQ4plex); K7(iTRAQ4plex)	Unique	4.326E3	1.985E4	5.739E3	8.009E3	0.006	0.1568	1.70			0	2	538.35834	67.43	EV_set1_060319_050219_Frac4.raw
105990532																					yGMVAQVTQTLk	N-Term(iTRAQ4plex); K12(iTRAQ4plex)	Unique	4.027E3	2.142E4	7.365E3	7.903E3	0	0.01092	2.56			0	2	813.95941	60.22	EV_set1_060319_050219_Frac3.raw
105990532																					tGISPLALIk	N-Term(iTRAQ4plex); K10(iTRAQ4plex)	Unique	2.153E3	1.634E4	6.631E3	7.713E3	0	0.03071	2.23			0	3	434.28552	67.08	EV_set1_060319_050219_Frac3.raw
105990532																					kLTISEQNIQR	N-Term(iTRAQ4plex); K1(iTRAQ4plex)	Unique	2.218E3	1.569E4	4.814E3	7.552E3	0.004	0.1374	2.49			1	3	539.98901	32.28	EV_set1_060319_050219_Frac5.raw
105990532																					vTQEFHmk	N-Term(iTRAQ4plex); M7(Oxidation); K8(iTRAQ4plex)	Unique	5.120E3	1.872E4	7.736E3	7.368E3	0.006	0.09839		19	1.27399	0	3	441.90311	20.21	EV_set1_060319_050219_Frac5.raw
105990532																					eFQVPTFTIPk	N-Term(iTRAQ4plex); K11(iTRAQ4plex)	Unique	3.794E3	1.399E4	9.033E3	7.343E3	0.001	0.04095	1.43			0	3	532.30676	72.65	EV_set1_060319_050219_Frac3.raw
105990532																					IPYTIITTPPLk	N-Term(iTRAQ4plex); K12(iTRAQ4plex)	Unique	4.541E3	1.831E4	7.424E3	7.276E3	0	0.03787	2.49			0	3	549.01038	74.07	EV_set1_060319_050219_Frac3.raw
105990532																					ILSGGNTLHLVSTTk	N-Term(iTRAQ4plex); K15(iTRAQ4plex)	Unique	5.817E3	2.106E4	7.332E3	7.221E3	0.009	0.1877	2.28			0	4	458.02301	51.05	EV_set1_060319_050219_Frac4.raw
105990532																					eQHLFLPFSYk	N-Term(iTRAQ4plex); K11(iTRAQ4plex)	Unique	4.063E3	1.763E4	8.577E3	7.186E3	0	0.0044	2.74			0	3	566.31445	61.55	EV_set1_060319_050219_Frac5.raw
105990532																					sVGFHLPSR	N-Term(iTRAQ4plex)	Unique	8.419E3	1.190E4	7.270E3	7.141E3	0.002	0.09561	1.81			0	3	381.88400	37.95	EV_set1_060319_050219_Frac4.raw
105990532																					ILSGGNTLHLVSTTk	N-Term(iTRAQ4plex); K15(iTRAQ4plex)	Unique	4.056E3	1.767E4	1.100E4	7.135E3	0	0.02057	2.90			0	3	610.36212	50.93	EV_set1_060319_050219_Frac4.raw
105990532																					sLHMYANR	N-Term(iTRAQ4plex)	Unique	4.855E3	1.653E4	9.337E3	7.073E3	0.006	0.1584	2.10			0	3	379.19736	28.39	EV_set1_060319_050219_Frac3.raw
105990532																					vQGVEFSHR	N-Term(iTRAQ4plex)	Unique	2.482E3	1.344E4	3.984E3	7.028E3	0.001	0.05205	2.41			0	3	401.55078	29.44	EV_set1_060319_050219_Frac5.raw
105990532																					hVAEAIck	N-Term(iTRAQ4plex); C7(Methylthio); K8(iTRAQ4plex)	Unique	5.944E3	1.415E4	6.903E3	6.793E3	0.005	0.145	1.65			0	3	402.21835	33.04	EV_set1_060319_050219_Frac5.raw
105990532																					InGEIQALELPQk	N-Term(iTRAQ4plex); N2(Deamidated); K13(iTRAQ4plex)	Unique	4.032E3	8.802E3	6.430E3	6.771E3	0.001	0.0638	2.19			0	3	581.33496	66.37	EV_set1_060319_050219_Frac3.raw
105990532																					tGISPLALIk	N-Term(iTRAQ4plex); K10(iTRAQ4plex)	Unique	2.759E3	1.682E4	8.356E3	6.767E3	0	0.01373	2.50			0	3	434.28537	67.06	EV_set1_060319_050219_Frac3.raw
105990532																					sFDYHQFVDETNDk	N-Term(iTRAQ4plex); K14(iTRAQ4plex)	Unique	1.920E3	6.975E3	4.645E3	6.704E3	0	0.01926	2.02			0	4	508.99210	51.43	EV_set1_060319_050219_Frac5.raw
105990532																					aLVEQGFTVPEIk	N-Term(iTRAQ4plex); K13(iTRAQ4plex)	Unique	4.115E3	1.189E4	6.295E3	6.511E3	0	0.01547	2.39			0	3	573.66895	66.54	EV_set1_060319_050219_Frac3.raw
105990532																					vQGVEFSHR	N-Term(iTRAQ4plex)	Unique	3.375E3	1.062E4	4.331E3	6.473E3	0	0.03875	2.14			0	3	401.55093	24.27	EV_set1_060319_050219_Frac4.raw
105990532																					sVGFHLPSR	N-Term(iTRAQ4plex)	Unique	6.972E3	2.358E4	7.378E3	6.352E3	0.001	0.06732	2.38			0	3	381.88431	29.93	NIOP_Eset1_031019_Frac6.raw
105990532																					ILSGGNTLHLVSTTk	N-Term(iTRAQ4plex); K15(iTRAQ4plex)	Unique	5.383E3	2.035E4	9.996E3	6.305E3	0.008	0.1764	3.07			0	4	458.02319	50.99	EV_set1_060319_050219_Frac5.raw
105990532																					tGISPLALIk	N-Term(iTRAQ4plex); K10(iTRAQ4plex)	Unique	1.443E3	1.642E4	4.790E3	6.088E3	0	0.00619	3.07			0	2	650.92456	66.83	EV_set1_060319_050219_Frac4.raw
105990532																					IPYTIITTPPLk	N-Term(iTRAQ4plex); K12(iTRAQ4plex)	Unique	2.504E3	1.305E4	4.907E3	5.977E3	0.002	0.08869	2.12			0	2	823.01001	88.79	NIOP_Eset1_031019_Frac2.raw
105990532																					sVGFHLPSR	N-Term(iTRAQ4plex)	Unique	3.262E3	1.193E4	3.538E3	5.850E3	0.002	0.07217	1.90			0	3	381.88416	37.42	EV_set1_060319_050219_Frac4.raw
105990532																					tGISPLALIk	N-Term(iTRAQ4plex); K10(iTRAQ4plex)	Unique	2.220E3	1.421E4	6.340E3	5.775E3	0	0.02354	2.76			0	2	650.92426	66.84	EV_set1_060319_050219_Frac4.raw

105990532																					vSTAFVYTk	N-Term(iTRAQ4plex); K9(iTRAQ4plex)	Unique	3.709E3	1.659E4	5.091E3	5.767E3	0.009	0.1905	1.39			0	2	652.37854	38.91	NIOP_Eset1_031019_Frac2.raw
105990532																					hINIDQFVR	N-Term(iTRAQ4plex)	Unique	2.897E3	8.682E3	5.044E3	5.717E3	0.002	0.08696	2.09			0	3	429.24228	45.80	EV_set1_060319_050219_Frac4.raw
105990532																					hIYAISSAALSASYk	N-Term(iTRAQ4plex); K15(iTRAQ4plex)	Unique	2.697E3	1.472E4	7.173E3	5.640E3	0	0.00147	3.34			0	3	624.01447	49.79	EV_set1_060319_050219_Frac4.raw
105990532																					sISAALEHk	N-Term(iTRAQ4plex); K9(iTRAQ4plex)	Unique	4.128E3	1.304E4	4.772E3	5.576E3	0.001	0.06506	1.95			0	3	415.24716	29.86	EV_set1_060319_050219_Frac4.raw
105990532																					kLTISEQNIQR	N-Term(iTRAQ4plex); K1(iTRAQ4plex)	Unique	2.821E3	1.277E4	5.750E3	5.516E3	0.002	0.07486	2.92			1	3	539.98883	32.30	EV_set1_060319_050219_Frac5.raw
105990532																					lPYTIITTPPLk	N-Term(iTRAQ4plex); K12(iTRAQ4plex)	Unique	3.792E3	6.134E3	6.374E3	5.373E3	0.002	0.08496	1.83			0	3	549.01025	73.56	EV_set1_060319_050219_Frac3.raw
105990532																					tGISPLALIk	N-Term(iTRAQ4plex); K10(iTRAQ4plex)	Unique	4.276E3	1.601E4	9.321E3	5.222E3	0.001	0.05488	2.10			0	3	434.28549	67.12	EV_set1_060319_050219_Frac3.raw
105990532																					eFQVPTFTIPk	N-Term(iTRAQ4plex); K11(iTRAQ4plex)	Unique	3.274E3	8.629E3	3.553E3	5.146E3	0.001	0.06451	1.62			0	3	532.30676	72.65	EV_set1_060319_050219_Frac3.raw
105990532																					qHIEAIDVR	N-Term(iTRAQ4plex)	Unique	3.263E3	6.856E3	5.179E3	5.047E3	0.003	0.09919	1.92			0	3	408.89804	25.55	EV_set1_060319_050219_Frac4.raw
105990532																					sVSDGIAALDLNAVANk	N-Term(iTRAQ4plex); K17(iTRAQ4plex)	Unique	4.371E3	9.996E3	6.207E3	5.039E3	0	0.0232	2.13			0	3	649.36420	68.41	EV_set1_060319_050219_Frac3.raw
105990532																					lPYTIITTPPLk	N-Term(iTRAQ4plex); K12(iTRAQ4plex)	Unique	4.986E3	8.929E3	2.739E3	5.019E3	0.004	0.1313	1.54			0	3	549.01056	73.60	EV_set1_060319_050219_Frac3.raw
105990532																					eQHLFLPFSYk	N-Term(iTRAQ4plex); K11(iTRAQ4plex)	Unique	5.093E3	5.731E3	2.500E3	4.876E3	0	0.01397	2.23			0	3	566.31403	61.76	EV_set1_060319_050219_Frac4.raw
105990532																					sVGFHLPSR	N-Term(iTRAQ4plex)	Unique	2.869E3	1.668E4	6.064E3	4.773E3	0	0.0275	2.34			0	3	381.88409	37.47	EV_set1_060319_050219_Frac5.raw
105990532																					tGISPLALIk	N-Term(iTRAQ4plex); K10(iTRAQ4plex)	Unique	1.281E3	1.161E4	3.116E3	4.768E3	0.003	0.1111	2.06			0	3	434.28531	66.82	EV_set1_060319_050219_Frac4.raw
105990532																					kGNVATEISTER	N-Term(iTRAQ4plex); K1(iTRAQ4plex)	Unique	4.242E3	2.881E3	6.078E3	4.712E3	0.006	0.1517	1.96			1	3	531.63232	22.24	EV_set1_060319_050219_Frac4.raw
105990532																					lPYTIITTPPLk	N-Term(iTRAQ4plex); K12(iTRAQ4plex)	Unique	2.352E3	7.440E3	3.549E3	4.703E3	0	0.02463	2.30			0	3	549.01031	73.22	EV_set1_060319_050219_Frac5.raw
105990532																					lIVAmSSWLQk	N-Term(iTRAQ4plex); M5(Oxidation); K11(iTRAQ4plex)	Unique	2.067E3	1.333E4	4.732E3	4.678E3	0.002	0.08764	1.77			0	2	790.45844	64.73	EV_set1_060319_050219_Frac4.raw
105990532																					eQHLFLPFSYk	N-Term(iTRAQ4plex); K11(iTRAQ4plex)	Unique	2.690E3	8.261E3	3.998E3	4.476E3	0	0.00136	2.90			0	3	566.31403	61.76	EV_set1_060319_050219_Frac4.raw
105990532																					lPYTIITTPPLk	N-Term(iTRAQ4plex); K12(iTRAQ4plex)	Unique	1.897E3	1.343E4	5.658E3	4.392E3	0.008	0.1814	1.21			0	2	823.01166	73.67	EV_set1_060319_050219_Frac3.raw
105990532																					sISAALEHk	N-Term(iTRAQ4plex); K9(iTRAQ4plex)	Unique	3.609E3	9.505E3	5.191E3	4.351E3	0.001	0.0425	2.49			0	3	415.24518	29.88	EV_set1_060319_050219_Frac4.raw
105990532																					eQHLFLPFSYk	N-Term(iTRAQ4plex); K11(iTRAQ4plex)	Unique	2.800E3	1.428E4	6.056E3	4.340E3	0	0.00539	3.16			0	3	566.31403	61.57	EV_set1_060319_050219_Frac5.raw
105990532																					kGNVATEISTER	N-Term(iTRAQ4plex); K1(iTRAQ4plex)	Unique	2.453E3	7.444E3	5.239E3	4.331E3	0.001	0.06376	2.63			1	3	531.63251	22.22	EV_set1_060319_050219_Frac4.raw
105990532																					eQHLFLPFSYk	N-Term(iTRAQ4plex); K11(iTRAQ4plex)	Unique	3.076E3	9.384E3	3.581E3	4.211E3	0	0.02996	2.13			0	3	566.31415	61.61	EV_set1_060319_050219_Frac5.raw
105990532																					iEIPLPFGGk	N-Term(iTRAQ4plex); K10(iTRAQ4plex)	Unique	3.481E3	1.210E4	5.445E3	4.210E3	0.003	0.1099	1.57			0	3	453.61325	77.05	EV_set1_060319_050219_Frac3.raw
105990532																					lIVAmSSWLQk	N-Term(iTRAQ4plex); M5(Oxidation); K11(iTRAQ4plex)	Unique	8.836E2	1.118E4	3.359E3	4.145E3	0	0.00871	2.40			0	3	527.30811	64.65	EV_set1_060319_050219_Frac4.raw
105990532																					lIDVISMYR	N-Term(iTRAQ4plex)	Unique	2.901E3	9.947E3	3.090E3	4.140E3	0.007	0.1087		23	0.54542	0	2	627.35614	78.10	EV_set1_060319_050219_Frac3.raw
105990532																					lPYTIITTPPLk	N-Term(iTRAQ4plex); K12(iTRAQ4plex)	Unique	4.198E3	7.125E3	3.379E3	3.967E3	0.003	0.105	1.73			0	3	549.01086	73.76	EV_set1_060319_050219_Frac3.raw
105990532																					fIIPGLk	N-Term(iTRAQ4plex); K7(iTRAQ4plex)	Unique	1.111E3	1.103E4	5.010E3	3.637E3	0.008	0.1773	1.80			0	3	359.24146	67.06	EV_set1_060319_050219_Frac4.raw
105990532																					sVGFHLPSR	N-Term(iTRAQ4plex)	Unique	2.891E3	1.027E4	4.130E3	3.379E3	0.006	0.1549	1.94			0	3	381.88391	37.45	EV_set1_060319_050219_Frac4.raw
105990532																					lPYTIITTPPLk	N-Term(iTRAQ4plex); K12(iTRAQ4plex)	Unique	1.654E3	7.314E3	2.701E3	3.334E3	0.001	0.06434	1.87			0	3	549.01031	73.22	EV_set1_060319_050219_Frac5.raw
105990532																					iEIPLPFGGk	N-Term(iTRAQ4plex); K10(iTRAQ4plex)	Unique	8.932E2	7.647E3	4.022E3	2.937E3	0.007	0.1623	1.54			0	3	453.61343	77.10	EV_set1_060319_050219_Frac3.raw
105990532																					lIVAmSSWLQk	N-Term(iTRAQ4plex); M5(Oxidation); K11(iTRAQ4plex)	Unique	9.644E2	6.805E3	2.945E3	2.845E3	0	0.01113	2.47			0	3	527.30811	64.66	EV_set1_060319_050219_Frac4.raw
105990532																					tGISPLALIk	N-Term(iTRAQ4plex); K10(iTRAQ4plex)	Unique	1.211E3	1.406E4	3.943E3	2.758E3	0.001	0.05359	2.27			0	3	434.28543	66.80	EV_set1_060319_050219_Frac4.raw
105990532																					vQGVEFSHR	N-Term(iTRAQ4plex)	Unique	2.123E3	5.512E3	3.732E3	2.345E3	0.004	0.1191	1.59			0	3	401.55072	29.48	EV_set1_060319_050219_Frac5.raw
105990532																					kGNVATEISTER	N-Term(iTRAQ4plex); K1(iTRAQ4plex)	Unique	1.117E3	2.981E3	1.190E3	1.169E3	0	0.01798	2.59			1	3	531.63251	22.40	EV_set1_060319_050219_Frac5.raw
105990532																					lPYTIITTPPLk	N-Term(iTRAQ4plex); K12(iTRAQ4plex)	Unique	4.604E3	7.731E3	3.927E3		0.002	0.0696	2.36			0	3	549.01025	88.59	NIOP_Eset1_031019_Frac2.raw
105990532																					lPYTIITTPPLk	N-Term(iTRAQ4plex); K12(iTRAQ4plex)	Unique	3.041E3	3.946E3	3.219E3		0	0.01733	2.39			0	3	549.01135	88.42	NIOP_Eset1_031019_Frac2.raw
105990532																					lIVAmSSWLQk	N-Term(iTRAQ4plex); M5(Oxidation); K11(iTRAQ4plex)	Unique		8.574E3	3.876E3	1.537E3	0.001	0.06569	1.67			0	3	527.30817	64.70	EV_set1_060319_050219_Frac4.raw
105990532																					lPYTIITTPPLk	N-Term(iTRAQ4plex); K12(iTRAQ4plex)	Unique		3.349E3	2.219E3	1.820E3	0.002	0.06961	1.68			0	3	549.01019	73.39	EV_set1_060319_050219_Frac4.raw
105990532																					lPYTIITTPPLk	N-Term(iTRAQ4plex); K12(iTRAQ4plex)	Unique		8.370E3	2.108E3	2.845E3	0.003	0.1052	1.55			0	4	412.00912	73.81	EV_set1_060319_050219_Frac3.raw

105990532																					kYTYNYEAESSSG<br>VPGTADSR	N-Term(iTRAQ4plex);<br>K1(iTRAQ4plex)	Unique		2.604E3	1.967E3	1.369E3	0.004	0.1348	2.24			1	3	857.41138	35.72	EV_set1_060319_0<br>50219_Frac5.raw
105990532																					lPYTIITTPPLk	N-Term(iTRAQ4plex);<br>K12(iTRAQ4plex)	Unique		2.579E3		1.587E3	0.007	0.1671	1.26			0	3	549.01031	73.51	EV_set1_060319_0<br>50219_Frac4.raw
105990532																					kGNVATEISTER	N-Term(iTRAQ4plex);<br>K1(iTRAQ4plex)	Unique		2.963E3	1.218E3		0.008	0.1733	1.95			1	3	531.63220	22.43	EV_set1_060319_0<br>50219_Frac5.raw
105990532																					kGNVATEISTER	N-Term(iTRAQ4plex);<br>K1(iTRAQ4plex)	Unique		1.228E3	1.506E3		0.006	0.0975		33	0.06112	1	3	531.63226	26.57	EV_set1_060319_0<br>50219_Frac5.raw
116256327	4311	MME	neprilysin	1	3	GBC vs Healthy,<br>GSD, XGC	0.93	3.696	1.9E-03	2.471	5.3E-03	3.002	2.6E-03	1.246	0.700	31.6	25.3	4.9	6.6	20.3																	
116256327																					lkPILTk	N-Term(iTRAQ4plex);<br>K2(iTRAQ4plex);<br>K7(iTRAQ4plex)	Unique	2.775E4	5.104E4	4.466E4	1.956E5	0.001	0.04819	2.95			0	3	415.62698	28.29	NIOP_Eset1_03101<br>9_Frac6.raw
116256327																					lkPILTk	N-Term(iTRAQ4plex);<br>K2(iTRAQ4plex);<br>K7(iTRAQ4plex)	Unique	3.655E4	4.146E4	4.835E4	1.336E5	0.002	0.07865	2.76			0	3	415.62622	28.46	NIOP_Eset1_03101<br>9_Frac6.raw
116256327																					lkPILTk	N-Term(iTRAQ4plex);<br>K2(iTRAQ4plex);<br>K7(iTRAQ4plex)	Unique	1.113E4	1.838E4	1.873E4	5.007E4	0.009	0.189	2.05			0	3	415.62689	28.11	NIOP_Eset1_03101<br>9_Frac6.raw
116292750	4018	LPA	apolipoprotein(a)	2	3	GBC vs GSD	1.23	1.111	4.7E-01	0.454	1.2E-01	1.119	7.0E-01	1.013	0.395	23.1	7.6	23.7	0.6	31.7																	
116292750																					tPAYYPNAGLIk	N-Term(iTRAQ4plex);<br>K12(iTRAQ4plex)	Unique	2.813E4	7.884E4	3.679E4	4.437E4	0.001	0.01842		12	6.04714	0	3	532.64014	52.09	EV_set1_060319_0<br>50219_Frac3.raw
116292750																					tPAYYPNAGLIk	N-Term(iTRAQ4plex);<br>K12(iTRAQ4plex)	Unique	2.859E4	6.527E4	3.753E4	3.866E4	0.001	0.04526	2.74			0	3	532.63971	52.06	EV_set1_060319_0<br>50219_Frac3.raw
116292750																					rIPLYYPnAGLTR	N-Term(iTRAQ4plex);<br>N8(Deamidated)	Unique	3.857E3	3.744E3	4.312E3	3.030E3	0.001	0.0176		23	0.84888	1	3	560.31635	54.43	EV_set1_060319_0<br>50219_Frac5.raw
157266300	290	ANPEP	aminopeptidase N	11	38	GBC vs Healthy,<br>GSD, XGC	11.79	4.031	4.3E-29	2.535	1.8E-25	3.918	9.5E-28	1.051	0.597	76.7	39.6	76.8	29.6	47.2																	
157266300																					gFYISk	N-Term(iTRAQ4plex);<br>K6(iTRAQ4plex)	Unique	7.735E4	1.517E5	1.200E5	7.417E5	0.005	0.1481	2.79			0	2	501.79663	41.23	EV_set1_060319_0<br>50219_Frac3.raw
157266300																					gLYVFk	N-Term(iTRAQ4plex);<br>K6(iTRAQ4plex)	Unique	4.074E4	7.291E4	7.082E4	3.576E5	0.002	0.09338	2.51			0	2	507.81479	51.28	EV_set1_060319_0<br>50219_Frac3.raw
157266300																					lPNTLkPDSYR	N-Term(iTRAQ4plex);<br>K6(iTRAQ4plex)	Unique	3.389E4	6.358E4	5.081E4	3.166E5	0	0.0039	3.29			0	3	531.30652	32.16	NIOP_Eset1_03101<br>9_Frac6.raw
157266300																					lPNTLkPDSYR	N-Term(iTRAQ4plex);<br>K6(iTRAQ4plex)	Unique	3.483E4	7.249E4	4.925E4	2.798E5	0	0.00697	3.47			0	3	531.30634	31.99	NIOP_Eset1_03101<br>9_Frac6.raw
157266300																					sIQLPTTVR	N-Term(iTRAQ4plex)	Unique	1.822E4	4.825E4	3.049E4	2.275E5	0	0.02911	2.80			0	2	579.85168	36.31	NIOP_Eset1_03101<br>9_Frac2.raw
157266300																					lPNTLkPDSYR	N-Term(iTRAQ4plex);<br>K6(iTRAQ4plex)	Unique	2.834E4	5.199E4	3.482E4	2.260E5	0	0.02049	3.21			0	3	531.30652	32.34	NIOP_Eset1_03101<br>9_Frac6.raw
157266300																					lPNTLkPDSYR	N-Term(iTRAQ4plex);<br>K6(iTRAQ4plex)	Unique	2.561E4	4.773E4	3.350E4	2.048E5	0	0.0073	3.30			0	3	531.30640	38.27	EV_set1_060319_0<br>50219_Frac5.raw
157266300																					sIQLPTTVR	N-Term(iTRAQ4plex)	Unique	1.544E4	3.594E4	2.101E4	1.677E5	0.003	0.1025	2.62			0	2	579.85248	36.14	NIOP_Eset1_03101<br>9_Frac2.raw
157266300																					vTLRPYLTPNDR	N-Term(iTRAQ4plex)	Unique	5.069E4	6.015E4	1.058E5	1.422E5	0	0.00068	4.51			0	3	530.30298	44.96	EV_set1_060319_0<br>50219_Frac3.raw
157266300																					lPNTLkPDSYR	N-Term(iTRAQ4plex);<br>K6(iTRAQ4plex)	Unique	2.708E4	4.569E4	3.705E4	1.358E5	0.001	0.04464	2.71			0	3	531.30597	31.82	NIOP_Eset1_03101<br>9_Frac6.raw
157266300																					lPNTLkPDSYR	N-Term(iTRAQ4plex);<br>K6(iTRAQ4plex)	Unique	1.838E4	2.708E4	1.894E4	1.086E5	0	0.02422	2.79			0	3	531.30640	38.27	EV_set1_060319_0<br>50219_Frac5.raw
157266300																					lPNTLkPDSYR	N-Term(iTRAQ4plex);<br>K6(iTRAQ4plex)	Unique	2.390E4	3.273E4	2.953E4	9.692E4	0	0.03871	2.70			0	3	531.30591	38.20	EV_set1_060319_0<br>50219_Frac4.raw
157266300																					vTLRPYLTPNDR	N-Term(iTRAQ4plex)	Unique	6.149E4	5.349E4	7.050E4	9.393E4	0.005	0.1409	2.36			0	3	530.30255	45.00	EV_set1_060319_0<br>50219_Frac3.raw
157266300																					sIQLPTTVR	N-Term(iTRAQ4plex)	Unique	8.019E3	1.762E4	9.285E3	8.031E4	0.001	0.0598	2.46			0	2	579.85156	36.49	NIOP_Eset1_03101<br>9_Frac2.raw
157266300																					vTLRPYLTPNDR	N-Term(iTRAQ4plex)	Unique	8.865E3	2.021E4	1.564E4	6.844E4	0	0.00151	3.62			0	3	530.30261	44.04	EV_set1_060319_0<br>50219_Frac5.raw
157266300																					kIQTQLQR	N-Term(iTRAQ4plex);<br>K1(iTRAQ4plex)	Unique	3.372E4	2.028E4	3.590E4	6.541E4	0.002	0.08959	2.58			1	3	434.94107	12.49	NIOP_Eset1_03101<br>9_Frac6.raw
157266300																					vTLRPYLTPNDR	N-Term(iTRAQ4plex)	Unique	1.189E4	1.384E4	1.315E4	5.054E4	0	0.00433	3.30			0	3	530.30267	44.00	EV_set1_060319_0<br>50219_Frac4.raw
157266300																					vTLRPYLTPNDR	N-Term(iTRAQ4plex)	Unique	7.177E3	1.534E4	1.396E4	4.953E4	0.004	0.1206	2.26			0	3	530.30255	44.07	EV_set1_060319_0<br>50219_Frac5.raw
157266300																					sIQLPTTVR	N-Term(iTRAQ4plex)	Unique	4.468E3	9.603E3	5.619E3	4.726E4	0.001	0.0524	2.75			0	2	579.85168	45.75	EV_set1_060319_0<br>50219_Frac1.raw
157266300																					sIQLPTTVR	N-Term(iTRAQ4plex)	Unique	5.762E3	1.137E4	9.235E3	4.178E4	0.002	0.09777	2.36			0	2	579.85242	35.97	NIOP_Eset1_03101<br>9_Frac2.raw
157266300																					vTLRPYLTPnDR	N-Term(iTRAQ4plex);<br>N10(Deamidated)	Unique	7.688E3	9.669E3	9.511E3	2.370E4	0	0.01548	2.58			0	3	530.63062	46.59	EV_set1_060319_0<br>50219_Frac5.raw
157266300																					qVTPLFIHFR	N-Term(iTRAQ4plex)	Unique	4.299E3	8.055E3	4.460E3	2.065E4	0	0.00551	3.33			0	3	467.94211	59.12	EV_set1_060319_0<br>50219_Frac5.raw
157266300																					eNSLLFDPLSSSSS<br>Nk	N-Term(iTRAQ4plex);<br>K16(iTRAQ4plex)	Unique	4.907E3	3.726E3	6.337E3	2.000E4	0	0.00018	3.48			0	3	671.68427	66.54	EV_set1_060319_0<br>50219_Frac3.raw
157266300																					sFPcFDEPAmk	N-Term(iTRAQ4plex);<br>C4(Methylthio);<br>M10(Oxidation);<br>K11(iTRAQ4plex)	Unique	9.137E3	4.943E3	7.664E3	1.565E4	0.005	0.1396	1.27			0	3	541.24841	62.52	EV_set1_060319_0<br>50219_Frac3.raw
157266300																					vTLRPYLTPnDR	N-Term(iTRAQ4plex);<br>N10(Deamidated)	Unique	6.667E3	5.655E3	3.694E3	1.402E4	0.003	0.0986	1.64			0	3	530.63043	48.09	EV_set1_060319_0<br>50219_Frac4.raw
157266300																					eNSLLFDPLSSSSS<br>Nk	N-Term(iTRAQ4plex);<br>K16(iTRAQ4plex)	Unique	6.103E3	4.499E3	8.121E3	1.367E4	0	0.00951	2.78			0	3	671.68494	66.55	EV_set1_060319_0<br>50219_Frac3.raw
157266300																					qVTPLFIHFR	N-Term(iTRAQ4plex)	Unique	2.234E3	4.661E3	3.827E3	1.314E4	0	0.00785	3.09			0	3	467.94211	59.12	EV_set1_060319_0<br>50219_Frac5.raw
157266300																					aQIINDAFNLASAHk	N-Term(iTRAQ4plex);<br>K15(iTRAQ4plex)	Unique	3.296E3	5.175E3	4.334E3	1.289E4	0	0.00035	3.64			0	3	634.35364	61.44	EV_set1_060319_0<br>50219_Frac5.raw

157266300																					aQIINDAFNLASAHk	N-Term(iTRAQ4plex);<br>K15(iTRAQ4plex)	Unique	3.426E3	5.301E3	5.902E3	1.236E4	0	0.00692	2.96			0	3	634.35364	61.44	EV_set1_060319_0<br>50219_Frac5.raw
157266300																					aQIINDAFNLASAHk	N-Term(iTRAQ4plex);<br>K15(iTRAQ4plex)	Unique	1.579E3	9.692E2	2.177E3	1.146E4	0	0.00143	2.90			0	4	476.01752	61.49	EV_set1_060319_0<br>50219_Frac5.raw
157266300																					dHSAIPVINR	N-Term(iTRAQ4plex)	Unique	1.671E3	2.806E3	3.434E3	1.035E4	0	0.02318	2.30			0	3	422.57404	30.97	EV_set1_060319_0<br>50219_Frac4.raw
157266300																					qVTPLFIHFR	N-Term(iTRAQ4plex)	Unique	2.474E3	2.535E3	3.348E3	1.029E4	0	0.0271	2.48			0	3	467.94229	59.14	EV_set1_060319_0<br>50219_Frac5.raw
157266300																					dHSAIPVINR	N-Term(iTRAQ4plex)	Unique	3.313E3	3.736E3	5.418E3	1.010E4	0.004	0.1183	2.25			0	3	422.57397	31.13	EV_set1_060319_0<br>50219_Frac4.raw
157266300																					aQIINDAFNLASAHk	N-Term(iTRAQ4plex);<br>K15(iTRAQ4plex)	Unique	1.924E3	3.575E3	1.205E3	9.889E3	0	0.02456	1.87			0	4	476.01746	61.51	EV_set1_060319_0<br>50219_Frac5.raw
157266300																					dHSAIPVINR	N-Term(iTRAQ4plex)	Unique	3.084E3	3.312E3	5.625E3	9.635E3	0	0.02109	2.63			0	3	422.57379	31.02	EV_set1_060319_0<br>50219_Frac4.raw
157266300																					aQIINDAFNLASAHk	N-Term(iTRAQ4plex);<br>K15(iTRAQ4plex)	Unique	2.165E3	4.051E3	4.083E3	9.190E3	0	0.00417	2.45			0	3	634.35388	61.73	EV_set1_060319_0<br>50219_Frac4.raw
157266300																					aQIINDAFNLASAHk	N-Term(iTRAQ4plex);<br>K15(iTRAQ4plex)	Unique	1.868E3	2.115E3	3.206E3	7.642E3	0.001	0.06409	1.75			0	3	634.35345	61.47	EV_set1_060319_0<br>50219_Frac5.raw
157266300																					aQIINDAFNLASAHk	N-Term(iTRAQ4plex);<br>K15(iTRAQ4plex)	Unique	3.655E3	4.276E3	2.027E3	5.948E3	0	0.01637	2.26			0	3	634.35388	61.74	EV_set1_060319_0<br>50219_Frac4.raw
223029410	7094	TLN1	talin-1	2	5	GBC vs XGC	1.06	1.099	7.9E-02	0.609	1.3E-01	0.470	7.8E-06	2.747	1.489	3.9	54.5	38.2	45.8	11.5																	
223029410																					aSAGPQPLLVQSck	N-Term(iTRAQ4plex);<br>C13(Methylthio);<br>K14(iTRAQ4plex)	Unique	1.361E4	2.281E4	4.070E4	1.813E4	0	0.00023	3.72			0	3	578.31531	54.85	EV_set1_060319_0<br>50219_Frac3.raw
223029410																					aSAGPQPLLVQSck	N-Term(iTRAQ4plex);<br>C13(Methylthio);<br>K14(iTRAQ4plex)	Unique	1.125E4	2.671E4	5.358E4	1.506E4	0	0.00033	3.29			0	3	578.31488	54.83	EV_set1_060319_0<br>50219_Frac3.raw
223029410																					vSHVLAALQAGNR	N-Term(iTRAQ4plex)	Unique	6.850E3	6.466E3	1.596E4	8.863E3	0	0.00022	4.02			0	3	493.95523	46.22	EV_set1_060319_0<br>50219_Frac4.raw
223029410																					vSHVLAALQAGNR	N-Term(iTRAQ4plex)	Unique	5.051E3	1.271E4	2.754E4	6.937E3	0	5.1E-05	4.69			0	3	493.95517	46.23	EV_set1_060319_0<br>50219_Frac5.raw
223029410																					vSHVLAALQAGNR	N-Term(iTRAQ4plex)	Unique	3.868E3	5.912E3	1.372E4	5.941E3	0	0.00931	3.45			0	3	493.95517	46.23	EV_set1_060319_0<br>50219_Frac5.raw
295821193	6288	SAA1	serum amyloid A-1 protein	2	3	GBC vs Healthy, GSD, XGC	27.05	3.928	9.6E-02	5.999	8.1E-02	5.536	9.8E-02	0.859	1.024	39.1	8.6	12.9	7.7	4.3																	
295821193																					dPNHFRPAGLPEk<br>Y	N-Term(iTRAQ4plex);<br>K13(iTRAQ4plex)	Unique	3.616E4	2.211E4	3.394E4	1.730E5	0.004	0.1236	2.89			1	4	483.01147	33.30	NIOP_Eset1_03101<br>9_Frac6.raw
295821193																					dPNHFRPAGLPEk<br>Y	N-Term(iTRAQ4plex);<br>K13(iTRAQ4plex)	Unique	2.612E4	1.944E4	2.899E4	1.612E5	0.008	0.1733	2.96			1	4	483.01111	33.48	NIOP_Eset1_03101<br>9_Frac6.raw
295821193																					rGPGGAWAAEVIS<br>DAR	N-Term(iTRAQ4plex)	Unique	7.213E3	9.460E3	8.431E3	9.311E3	0	0.00304		35	0.03829	1	3	586.31226	50.47	EV_set1_060319_0<br>50219_Frac5.raw
310923127	4831	NME2	nucleoside diphosphate kinase B isoform b	1	3	GBC vs GSD	14.63	0.897	2.5E-01	0.102	1.4E-05	0.816	3.4E-01	1.156	0.125	13.5	19.8	4.1	17.6	17.2																	
310923127																					tFIAIkPDGVQR	N-Term(iTRAQ4plex);<br>K6(iTRAQ4plex)	Unique	4.945E3	4.958E4	1.009E4	1.014E4	0.002	0.08962	2.25			0	3	544.99023	42.69	EV_set1_060319_0<br>50219_Frac4.raw
310923127																					tFIAIkPDGVQR	N-Term(iTRAQ4plex);<br>K6(iTRAQ4plex)	Unique	7.732E3	6.366E4	1.154E4	8.441E3	0.005	0.1482	2.31			0	3	544.99268	42.73	EV_set1_060319_0<br>50219_Frac5.raw
310923127																					tFIAIkPDGVQR	N-Term(iTRAQ4plex);<br>K6(iTRAQ4plex)	Unique	8.362E3	7.179E4	1.110E4	8.341E3	0	0.02761	2.74			0	3	544.99268	42.72	EV_set1_060319_0<br>50219_Frac5.raw
315075333	6291	SAA4	serum amyloid A-4 protein	2	5	GBC vs GSD	17.69	1.051	9.3E-02	0.399	7.1E-04	1.230	2.8E-01	0.791	0.343	17.0	9.0	22.8	22.0	25.5																	
315075333																					fRPDGLPk	N-Term(iTRAQ4plex);<br>K8(iTRAQ4plex)	Unique	7.014E4	1.640E5	7.168E4	8.117E4	0	0.00361	3.33			0	3	406.57922	23.89	NIOP_Eset1_03101<br>9_Frac6.raw
315075333																					fRPDGLPk	N-Term(iTRAQ4plex);<br>K8(iTRAQ4plex)	Unique	1.925E4	4.738E4	1.741E4	2.463E4	0.004	0.1291	2.44			0	3	406.57889	24.06	NIOP_Eset1_03101<br>9_Frac6.raw
315075333																					aYWDImISNHQNS<br>NR	N-Term(iTRAQ4plex);<br>M6(Oxidation)	Unique	4.746E3	7.399E3	5.617E3	6.803E3	0	0.01204	2.73			0	3	670.31848	45.97	EV_set1_060319_0<br>50219_Frac4.raw
315075333																					aYWDImISNHQNS<br>NR	N-Term(iTRAQ4plex);<br>M6(Oxidation)	Unique	8.031E3	1.337E4	6.685E3	6.501E3	0	0.00063	3.24			0	3	670.31848	45.97	EV_set1_060319_0<br>50219_Frac4.raw
315075333																					aYWDImISNHQNS<br>NR	N-Term(iTRAQ4plex);<br>M6(Oxidation)	Unique	3.186E3	9.179E3	5.434E3	5.071E3	0.008	0.1832	1.33			0	3	670.31848	46.00	EV_set1_060319_0<br>50219_Frac4.raw
316659409	71	ACTG1	actin, cytoplasmic 2	3	10	GBC vs XGC	12.27	1.042	3.5E-01	0.867	6.3E-01	0.186	7.7E-08	7.168	4.432	30.2	68.0	173.9	164.9	58.2																	
316659409																					hQGVMVGMGQk	N-Term(iTRAQ4plex);<br>K11(iTRAQ4plex)	Unique	1.451E4	2.099E4	6.283E4	3.318E4	0	0.00861	3.15			0	3	487.26276	16.10	NIOP_Eset1_03101<br>9_Frac6.raw
316659409																					hQGVmVGMGQk	N-Term(iTRAQ4plex);<br>M5(Oxidation);<br>K11(iTRAQ4plex)	Unique	1.385E4	9.671E3	4.181E4	1.265E4	0	0.00383	3.16			0	3	492.59457	12.50	NIOP_Eset1_03101<br>9_Frac6.raw
316659409																					hQGVmVGMGQk	N-Term(iTRAQ4plex);<br>M5(Oxidation);<br>K11(iTRAQ4plex)	Unique	9.583E3	8.713E3	2.927E4	1.219E4	0	0.02225	2.56			0	3	492.59460	12.32	NIOP_Eset1_03101<br>9_Frac6.raw
316659409																					hQGVMVGmGQk	N-Term(iTRAQ4plex);<br>M8(Oxidation);<br>K11(iTRAQ4plex)	Unique	8.042E3	5.893E3	2.739E4	8.839E3	0.001	0.04245	2.22			0	3	492.59467	10.72	NIOP_Eset1_03101<br>9_Frac6.raw
316659409																					aVFPSIVGRPR	N-Term(iTRAQ4plex)	Unique	5.554E3	1.198E4	1.222E5	8.241E3	0	0.00121	3.96			0	3	448.27386	40.45	NIOP_Eset1_03101<br>9_Frac6.raw
316659409																					hQGVmVGmGQk	N-Term(iTRAQ4plex);<br>M5(Oxidation);<br>M8(Oxidation);<br>K11(iTRAQ4plex)	Unique	4.293E3	4.368E3	3.163E4	8.177E3	0	0.0024	2.39			0	3	497.92624	9.51	NIOP_Eset1_03101<br>9_Frac6.raw
316659409																					aVFPSIVGRPR	N-Term(iTRAQ4plex)	Unique	5.050E3	1.200E4	1.026E5	6.391E3	0	0.00698	3.71			0	3	448.27377	40.62	NIOP_Eset1_03101<br>9_Frac6.raw
316659409																					aVFPSIVGRPR	N-Term(iTRAQ4plex)	Unique	4.347E3	5.845E3	5.056E4	5.710E3	0	0.01824	3.13			0	3	448.27411	40.79	NIOP_Eset1_03101<br>9_Frac6.raw
316659409																					gILTLk	N-Term(iTRAQ4plex);<br>K6(iTRAQ4plex)	Unique	5.641E3	1.153E4	6.624E4	5.618E3	0.002	0.07151	2.17			0	2	466.82278	45.79	EV_set1_060319_0<br>50219_Frac4.raw
316659409																					aVFPSIVGRPR	N-Term(iTRAQ4plex)	Unique	4.232E3	7.808E3	8.356E4	2.024E3	0	0.00281	3.94			0	3	448.27359	40.27	NIOP_Eset1_03101<br>9_Frac6.raw

325651886	4907	NT5E	5'-nucleotidase isoform 2	1	3	GBC vs Healthy, GSD, XGC	1.72	5.026	7.1E-03	2.842	5.6E-03	4.245	3.4E-03	1.212	0.628	23.9	3.0	43.7	18.4	5.4																	
325651886																					fPILSANIk	N-Term(iTRAQ4plex); K9(iTRAQ4plex)	Unique	8.821E3	1.744E4	1.595E4	3.989E4	0.002	0.07445	2.33			0	2	645.90442	63.71	EV_set1_060319_050219_Frac3.raw
325651886																					fPILSANIk	N-Term(iTRAQ4plex); K9(iTRAQ4plex)	Unique	4.714E3	7.782E3	7.382E3	2.885E4	0.009	0.1948	1.68			0	3	430.93890	63.66	EV_set1_060319_050219_Frac3.raw
325651886																					fPILSANIk	N-Term(iTRAQ4plex); K9(iTRAQ4plex)	Unique	2.356E3	4.466E3	3.263E3	1.690E4	0.004	0.1151	1.75			0	3	430.93872	63.23	EV_set1_060319_050219_Frac4.raw
332164777	5315	PKM	pyruvate kinase PKM isoform d	1	3	GBC vs XGC	1.75	1.185	2.2E-01	0.946	2.1E-01	0.290	8.2E-04	4.181	3.523	19.7	46.6	65.6	31.9	15.7																	
332164777																					gIFPVLck	N-Term(iTRAQ4plex); C7(Methylthio); K8(iTRAQ4plex)	Unique	2.782E3	1.464E3	1.218E4	5.139E3	0.002	0.06888	2.06			0	2	605.85010	75.66	EV_set1_060319_050219_Frac5.raw
332164777																					gIFPVLck	N-Term(iTRAQ4plex); C7(Methylthio); K8(iTRAQ4plex)	Unique	3.008E3	3.519E3	1.624E4	4.340E3	0.001	0.04375	2.13			0	2	605.85028	75.62	EV_set1_060319_050219_Frac5.raw
332164777																					gIFPVLck	N-Term(iTRAQ4plex); C7(Methylthio); K8(iTRAQ4plex)	Unique	1.741E3	2.407E3	1.234E4	2.202E3	0.001	0.06668	1.49			0	3	404.23553	75.75	EV_set1_060319_050219_Frac5.raw
371873523	55937	APOM	apolipoprotein M isoform 2	1	4	GBC vs GSD	6.03	0.580	7.4E-03	0.410	3.8E-04	1.095	7.8E-01	0.544	0.368	22.9	15.3	11.7	23.1	24.6																	
371873523																					aFLLTPR	N-Term(iTRAQ4plex)	Unique	3.397E4	5.135E4	3.461E4	2.788E4	0.003	0.1121	2.31			0	2	481.30103	43.73	NIOP_Eset1_031019_Frac2.raw
371873523																					aFLLTPR	N-Term(iTRAQ4plex)	Unique	3.188E4	3.682E4	1.791E4	1.939E4	0.002	0.09835	2.15			0	2	481.30206	43.55	NIOP_Eset1_031019_Frac2.raw
371873523																					aFLLTPR	N-Term(iTRAQ4plex)	Unique	1.579E4	2.491E4	1.018E4	9.554E3	0.004	0.07214		30	0.22945	0	2	481.30136	43.91	NIOP_Eset1_031019_Frac2.raw
371873523																					aFLLTPR	N-Term(iTRAQ4plex)	Unique	8.836E3	1.146E4	6.749E3	7.415E3	0.006	0.09997		23	1.23222	0	2	481.30130	52.60	EV_set1_060319_050219_Frac1.raw
530420068	29780	PARVB	PREDICTED: beta-parvin isoform X2	2	5	GBC vs GSD, XGC	5.13	0.773	7.7E-01	0.380	8.0E-04	0.193	8.9E-07	5.149	1.919	27.5	39.3	39.3	38.4	1.2																	
530420068																					sLITFVNk	N-Term(iTRAQ4plex); K8(iTRAQ4plex)	Unique	2.146E4	3.786E4	1.058E5	2.425E4	0	0.03622	2.92			0	2	605.37531	55.91	EV_set1_060319_050219_Frac3.raw
530420068																					sLITFVNk	N-Term(iTRAQ4plex); K8(iTRAQ4plex)	Unique	8.745E3	1.436E4	3.548E4	8.079E3	0.003	0.09887	1.76			0	3	403.91928	55.98	EV_set1_060319_050219_Frac3.raw
530420068																					vLYNLFTk	N-Term(iTRAQ4plex); K8(iTRAQ4plex)	Unique	4.942E3	1.352E4	3.776E4	6.704E3	0.002	0.09502	2.28			0	2	643.39148	69.92	EV_set1_060319_050219_Frac3.raw
530420068																					vLYNLFTk	N-Term(iTRAQ4plex); K8(iTRAQ4plex)	Unique	6.972E3	2.149E4	5.954E4	6.567E3	0	0.00797	2.62			0	2	643.39136	69.90	EV_set1_060319_050219_Frac3.raw
530420068																					vLYNLFTk	N-Term(iTRAQ4plex); K8(iTRAQ4plex)	Unique	3.014E3	5.316E3	2.004E4	1.283E3	0.002	0.07578	1.80			0	3	429.26370	69.96	EV_set1_060319_050219_Frac3.raw
557786227	125	ADH1B	alcohol dehydrogenase 1B isoform 2	1	3	GBC vs XGC	3.28	1.406	5.2E-02	0.512	1.1E-01	0.375	1.6E-03	3.844	1.370		32.0	4.3		27.3																	
557786227																					vIPLFTPQcGk	N-Term(iTRAQ4plex); C9(Methylthio); K11(iTRAQ4plex)	Unique	2.045E3	4.250E3	1.015E4	3.503E3	0.001	0.05427	2.02			0	3	512.95551	74.69	EV_set1_060319_050219_Frac3.raw
557786227																					vIPLFTPQcGk	N-Term(iTRAQ4plex); C9(Methylthio); K11(iTRAQ4plex)	Unique		4.335E3	8.642E3	2.895E3	0.001	0.04488	2.01			0	3	512.95520	74.16	EV_set1_060319_050219_Frac5.raw
557786227																					vIPLFTPQcGk	N-Term(iTRAQ4plex); C9(Methylthio); K11(iTRAQ4plex)	Unique		6.753E3	1.010E4	3.596E3	0.002	0.07936	1.66			0	3	512.95520	74.16	EV_set1_060319_050219_Frac5.raw
574584818	10382	TUBB4A	tubulin beta-4A chain isoform 4	2	5	GBC vs XGC	5.91	1.406	2.6E-01	1.352	3.4E-02	0.187	2.9E-04	7.052	5.152	42.2	18.8	46.6	62.1	69.4																	
574584818																					lAVNMVPFPR	N-Term(iTRAQ4plex)	Unique	3.059E3	3.901E3	2.786E4	6.881E3	0.002	0.08646	2.43			0	2	644.37152	68.97	EV_set1_060319_050219_Frac3.raw
574584818																					lAVNMVPFPR	N-Term(iTRAQ4plex)	Unique	3.296E3	2.840E3	4.986E4	6.375E3	0	0.01295	2.93			0	2	644.37152	68.97	EV_set1_060319_050219_Frac3.raw
574584818																					imNTFSVVPSPk	N-Term(iTRAQ4plex); M2(Oxidation); K12(iTRAQ4plex)	Unique	4.466E3	2.776E3	1.058E4	4.319E3	0.001	0.04968	1.98			0	3	541.97192	52.94	EV_set1_060319_050219_Frac4.raw
574584818																					lAVNMVPFPR	N-Term(iTRAQ4plex)	Unique	1.395E3	1.266E3	1.449E4	2.388E3	0.001	0.05058	2.03			0	3	429.91672	68.99	EV_set1_060319_050219_Frac3.raw
574584818																					lAVNMVPFPR	N-Term(iTRAQ4plex)	Unique	1.453E3	1.201E3	9.005E3	1.553E3	0	0.03882	2.11			0	3	429.91690	69.00	EV_set1_060319_050219_Frac3.raw
578804287	3655	ITGA6	PREDICTED: integrin alpha-6 isoform X6	4	9	GBC vs XGC	5.01	1.122	6.1E-01	0.783	5.2E-01	0.173	6.6E-09	6.231	6.087	11.1	28.4	50.6	35.2	61.7																	
578804287																					lLLVGAPR	N-Term(iTRAQ4plex)	Unique	3.788E4	3.263E4	2.931E5	5.574E4	0.007	0.1696	2.76			0	2	491.83011	55.47	EV_set1_060319_050219_Frac3.raw
578804287																					lLLVGAPR	N-Term(iTRAQ4plex)	Unique	3.861E4	5.790E4	6.767E5	5.351E4	0.002	0.0815	2.74			0	2	491.83011	55.47	EV_set1_060319_050219_Frac3.raw
578804287																					fSYLPIQk	N-Term(iTRAQ4plex); K8(iTRAQ4plex)	Unique	3.410E4	3.899E4	3.455E5	3.982E4	0.001	0.06142	2.72			0	2	642.38367	57.60	EV_set1_060319_050219_Frac3.raw
578804287																					fSYLPIqk	N-Term(iTRAQ4plex); Q7(Deamidated); K8(iTRAQ4plex)	Unique	1.089E4	1.644E4	8.752E4	1.392E4	0.002	0.07224	2.17			0	2	642.87463	59.79	EV_set1_060319_050219_Frac3.raw
578804287																					sRPVINIQk	N-Term(iTRAQ4plex); K9(iTRAQ4plex)	Unique	1.621E4	1.005E4	5.184E4	1.349E4	0.001	0.06045	2.17			0	3	448.28467	20.49	NIOP_Eset1_031019_Frac6.raw
578804287																					fSYLPIQk	N-Term(iTRAQ4plex); K8(iTRAQ4plex)	Unique	4.615E3	1.202E4	2.010E5	9.701E3	0.002	0.07976	2.47			0	3	428.59152	57.59	EV_set1_060319_050219_Frac3.raw
578804287																					fSYLPIqk	N-Term(iTRAQ4plex); Q7(Deamidated); K8(iTRAQ4plex)	Unique	3.371E3	4.490E3	2.713E4	4.604E3	0.001	0.05436	1.65			0	3	428.91925	59.86	EV_set1_060319_050219_Frac3.raw
578804287																					tAHIDVHFLk	N-Term(iTRAQ4plex); K10(iTRAQ4plex)	Unique		2.019E3	2.743E4		0.001	0.04394	2.20			0	4	367.96844	35.18	NIOP_Eset1_031019_Frac6.raw
578804287																					tAHIDVHFLk	N-Term(iTRAQ4plex); K10(iTRAQ4plex)	Unique		2.396E3	1.867E4		0.004	0.1264	1.58			0	4	367.96841	35.43	NIOP_Eset1_031019_Frac6.raw
767971525	928	CD9	PREDICTED: CD9 antigen isoform X2	1	19	GBC vs XGC	6.29	1.150	1.0E-01	0.777	2.4E-01	0.168	4.5E-15	7.037	5.078	35.5	29.6	86.9	80.9	61.1																	

767971525																					kDVLETFTVk	N-Term(iTRAQ4plex); K1(iTRAQ4plex); K10(iTRAQ4plex)	Unique	1.252E4	2.087E4	1.951E5	1.814E4	0	7.3E-05	4.81			1	3	537.99408	44.28	NIOP_Eset1_031019_Frac6.raw
767971525																					kDVLETFTVk	N-Term(iTRAQ4plex); K1(iTRAQ4plex); K10(iTRAQ4plex)	Unique	9.280E3	1.799E4	2.422E5	1.672E4	0	0.00013	5.28			1	3	537.99402	44.45	NIOP_Eset1_031019_Frac6.raw
767971525																					kDVLETFTVk	N-Term(iTRAQ4plex); K1(iTRAQ4plex); K10(iTRAQ4plex)	Unique	8.020E3	1.317E4	1.154E5	1.299E4	0	0.00053	4.51			1	3	537.99390	44.63	NIOP_Eset1_031019_Frac6.raw
767971525																					kDVLETFTVk	N-Term(iTRAQ4plex); K1(iTRAQ4plex); K10(iTRAQ4plex)	Unique	5.712E3	5.300E3	2.024E4	1.170E4	0	0.02007	2.74			1	3	537.99469	44.11	NIOP_Eset1_031019_Frac6.raw
767971525																					kDVLETFTVk	N-Term(iTRAQ4plex); K1(iTRAQ4plex); K10(iTRAQ4plex)	Unique	7.078E3	5.822E3	2.260E4	9.908E3	0	0.02107	2.71			1	3	537.99426	47.86	EV_set1_060319_050219_Frac4.raw
767971525																					kDVLETFTVk	N-Term(iTRAQ4plex); K1(iTRAQ4plex); K10(iTRAQ4plex)	Unique	8.750E3	1.014E4	4.927E4	9.710E3	0	0.00072	4.07			1	3	537.99414	44.81	NIOP_Eset1_031019_Frac6.raw
767971525																					kDVLETFTVk	N-Term(iTRAQ4plex); K1(iTRAQ4plex); K10(iTRAQ4plex)	Unique	8.162E3	7.797E3	1.653E4	9.631E3	0.002	0.07069	2.00			1	3	537.99475	45.46	NIOP_Eset1_031019_Frac6.raw
767971525																					kDVLETFTVk	N-Term(iTRAQ4plex); K1(iTRAQ4plex); K10(iTRAQ4plex)	Unique	5.776E3	7.123E3	5.908E4	8.778E3	0	0.00084	4.09			1	3	537.99426	47.85	EV_set1_060319_050219_Frac4.raw
767971525																					kDVLETFTVk	N-Term(iTRAQ4plex); K1(iTRAQ4plex); K10(iTRAQ4plex)	Unique	9.077E3	7.422E3	5.485E4	8.241E3	0	0.00199	3.49			1	3	537.99426	47.85	EV_set1_060319_050219_Frac4.raw
767971525																					kDVLETFTVk	N-Term(iTRAQ4plex); K1(iTRAQ4plex); K10(iTRAQ4plex)	Unique	5.424E3	3.535E3	2.081E4	7.702E3	0.001	0.06525	2.45			1	3	537.99445	47.90	EV_set1_060319_050219_Frac5.raw
767971525																					kDVLETFTVk	N-Term(iTRAQ4plex); K1(iTRAQ4plex); K10(iTRAQ4plex)	Unique	5.060E3	3.235E3	3.242E4	6.311E3	0	0.01388	2.97			1	4	403.74731	47.96	EV_set1_060319_050219_Frac5.raw
767971525																					kDVLETFTVk	N-Term(iTRAQ4plex); K1(iTRAQ4plex); K10(iTRAQ4plex)	Unique	7.055E3	6.817E3	1.983E4	6.203E3	0	0.02535	2.41			1	3	537.99469	44.99	NIOP_Eset1_031019_Frac6.raw
767971525																					kDVLETFTVk	N-Term(iTRAQ4plex); K1(iTRAQ4plex); K10(iTRAQ4plex)	Unique	2.466E3	5.911E3	2.987E4	6.163E3	0	0.01264	3.28			1	3	537.99432	47.88	EV_set1_060319_050219_Frac5.raw
767971525																					kDVLETFTVk	N-Term(iTRAQ4plex); K1(iTRAQ4plex); K10(iTRAQ4plex)	Unique	5.877E3	3.644E3	3.976E4	6.134E3	0.001	0.0397	2.40			1	2	806.48773	44.29	NIOP_Eset1_031019_Frac6.raw
767971525																					kDVLETFTVk	N-Term(iTRAQ4plex); K1(iTRAQ4plex); K10(iTRAQ4plex)	Unique	2.252E3	5.973E3	2.964E4	6.053E3	0	0.00343	3.13			1	3	537.99451	47.84	EV_set1_060319_050219_Frac5.raw
767971525																					kDVLETFTVk	N-Term(iTRAQ4plex); K1(iTRAQ4plex); K10(iTRAQ4plex)	Unique	3.671E3	8.121E3	5.930E4	5.260E3	0	0.00912	3.15			1	4	403.74731	47.96	EV_set1_060319_050219_Frac5.raw
767971525																					kDVLETFTVk	N-Term(iTRAQ4plex); K1(iTRAQ4plex); K10(iTRAQ4plex)	Unique	3.749E3	4.011E3	3.408E4	3.650E3	0	0.00589	3.10			1	3	537.99451	47.83	EV_set1_060319_050219_Frac5.raw
767971525																					kDVLETFTVk	N-Term(iTRAQ4plex); K1(iTRAQ4plex); K10(iTRAQ4plex)	Unique	3.053E3	5.493E3	7.389E4	3.544E3	0	0.00388	3.33			1	4	403.74731	47.95	EV_set1_060319_050219_Frac5.raw
767971525																					kDVLETFTVk	N-Term(iTRAQ4plex); K1(iTRAQ4plex); K10(iTRAQ4plex)	Unique	3.819E3	5.593E3	4.457E4	2.640E3	0.008	0.1778	2.01			1	2	806.48743	44.47	NIOP_Eset1_031019_Frac6.raw
767972841	10540	DCTN2	PREDICTED: dynactin subunit 2 isoform X1	1	2	GBC vs XGC	6.69	1.714	1.4E-02	1.543	1.6E-02	0.414	1.7E-03	4.240	3.630	20.0	3.0	8.7	11.1	11.8																	
767972841																					ITPVLLAk	N-Term(iTRAQ4plex); K8(iTRAQ4plex)	Unique	1.635E4	1.976E4	9.647E4	3.899E4	0.002	0.08008	2.35			0	2	571.89105	56.96	EV_set1_060319_050219_Frac3.raw
767972841																					ITPVLLAk	N-Term(iTRAQ4plex); K8(iTRAQ4plex)	Unique	6.828E3	6.069E3	3.470E4	1.247E4	0.004	0.1284	1.69			0	2	571.89105	56.49	EV_set1_060319_050219_Frac4.raw
767994628	3674	ITGA2B	PREDICTED: integrin alpha-IIb isoform X2	8	30	GBC vs XGC	9.20	0.954	4.6E-01	0.733	4.6E-01	0.272	2.1E-18	3.271	2.465	19.9	45.0	93.9	86.9	49.1																	
767994628																					vAIVVGAPR	N-Term(iTRAQ4plex)	Unique	5.670E4	6.302E4	1.392E5	6.739E4	0.002	0.06841	2.71			0	2	513.33368	43.66	EV_set1_060319_050219_Frac3.raw
767994628																					vAIVVGAPR	N-Term(iTRAQ4plex)	Unique	5.541E4	6.060E4	2.174E5	5.662E4	0.001	0.04389	2.87			0	2	513.33356	43.65	EV_set1_060319_050219_Frac3.raw
767994628																					scVLPQTk	N-Term(iTRAQ4plex); C2(Methylthio); K8(iTRAQ4plex)	Unique	2.750E4	2.634E4	7.974E4	4.868E4	0.005	0.1507	2.06			0	2	605.33270	40.87	EV_set1_060319_050219_Frac3.raw
767994628																					scVLPQTk	N-Term(iTRAQ4plex); C2(Methylthio); K8(iTRAQ4plex)	Unique	3.014E4	4.789E4	2.223E5	4.177E4	0.001	0.05978	2.59			0	2	605.33295	40.83	EV_set1_060319_050219_Frac3.raw
767994628																					scVLPQTk	N-Term(iTRAQ4plex); C2(Methylthio); K8(iTRAQ4plex)	Unique	2.343E4	2.651E4	1.185E5	4.061E4	0.004	0.1136	2.38			0	2	605.33295	40.83	EV_set1_060319_050219_Frac3.raw
767994628																					nVGSQTLQTFk	N-Term(iTRAQ4plex); K11(iTRAQ4plex)	Unique	2.818E4	2.475E4	1.037E5	3.494E4	0	0.00831	3.50			0	3	504.28748	42.03	EV_set1_060319_050219_Frac3.raw
767994628																					nVGSQTLQTFk	N-Term(iTRAQ4plex); K11(iTRAQ4plex)	Unique	2.399E4	2.251E4	5.939E4	3.185E4	0.001	0.04787	2.68			0	3	504.28702	42.05	EV_set1_060319_050219_Frac3.raw
767994628																					nVGSQTLQTFk	N-Term(iTRAQ4plex); K11(iTRAQ4plex)	Unique	2.670E4	2.922E4	1.215E5	3.039E4	0	0.00772	3.25			0	3	504.28748	41.98	EV_set1_060319_050219_Frac3.raw
767994628																					scVLPQTk	N-Term(iTRAQ4plex); C2(Methylthio); K8(iTRAQ4plex)	Unique	2.325E4	2.501E4	8.833E4	2.994E4	0.002	0.07545	2.44			0	3	403.89063	40.89	EV_set1_060319_050219_Frac3.raw
767994628																					scVLPQTk	N-Term(iTRAQ4plex); C2(Methylthio); K8(iTRAQ4plex)	Unique	2.616E4	4.135E4	1.486E5	2.707E4	0.003	0.1039	2.29			0	3	403.89063	40.88	EV_set1_060319_050219_Frac3.raw

767994628																					nVGSQTLQTFk	N-Term(iTRAQ4plex); K11(iTRAQ4plex)	Unique	1.001E4	1.554E4	6.814E4	1.320E4	0	0.02719	2.87			0	2	755.92761	42.03	EV_set1_060319_050219_Frac3.raw
767994628																					nVGSQTLQTFk	N-Term(iTRAQ4plex); K11(iTRAQ4plex)	Unique	1.281E4	8.128E3	2.778E4	1.268E4	0	0.02496	1.94			0	3	504.28708	34.20	NIOP_Eset1_031019_Frac2.raw
767994628																					vAIVVGAPR	N-Term(iTRAQ4plex)	Unique	1.244E4	2.113E4	1.037E5	1.233E4	0.003	0.1102	2.26			0	2	513.33301	33.55	NIOP_Eset1_031019_Frac2.raw
767994628																					nVGSQTLQTFk	N-Term(iTRAQ4plex); K11(iTRAQ4plex)	Unique	1.061E4	2.069E4	1.093E5	1.121E4	0	0.001	3.61			0	2	755.92761	42.03	EV_set1_060319_050219_Frac3.raw
767994628																					vAIVVGAPR	N-Term(iTRAQ4plex)	Unique	1.509E4	2.099E4	1.188E5	1.049E4	0.007	0.1632	2.33			0	2	513.33313	33.66	NIOP_Eset1_031019_Frac2.raw
767994628																					vAIVVGAPR	N-Term(iTRAQ4plex)	Unique	7.370E3	1.088E4	5.346E4	1.041E4	0.005	0.1427	2.16			0	2	513.33307	33.84	NIOP_Eset1_031019_Frac2.raw
767994628																					vYLFLQPR	N-Term(iTRAQ4plex)	Unique	8.195E3	1.233E4	4.092E4	9.661E3	0.003	0.1114	2.34			0	2	590.35406	68.09	EV_set1_060319_050219_Frac3.raw
767994628																					rAEYSPcR	N-Term(iTRAQ4plex); C7(Methylthio)	Unique	7.786E3	7.869E3	1.498E4	9.047E3	0.002	0.07678	2.33			1	3	391.18686	29.35	EV_set1_060319_050219_Frac3.raw
767994628																					hDLLVGAPLYmESR	N-Term(iTRAQ4plex); M11(Oxidation)	Unique	5.770E3	6.818E3	1.097E4	8.711E3	0.008	0.1754	1.48			0	3	587.64148	50.77	EV_set1_060319_050219_Frac5.raw
767994628																					hDLLVGAPLYmESR	N-Term(iTRAQ4plex); M11(Oxidation)	Unique	8.330E3	7.809E3	1.984E4	4.551E3	0	0.02028	2.78			0	3	587.64148	50.76	EV_set1_060319_050219_Frac5.raw
767994628																					vAIVVGAPR	N-Term(iTRAQ4plex)	Unique	4.565E3	5.974E3	2.766E4	4.373E3	0	0.03036	2.46			0	2	513.33350	43.30	EV_set1_060319_050219_Frac1.raw
767994628																					gQVLVFLGQSEGLR	N-Term(iTRAQ4plex)	Unique	3.842E3	2.215E3	4.495E3	4.015E3	0.002	0.06858	1.63			0	3	549.65100	72.81	EV_set1_060319_050219_Frac3.raw
767994628																					gPHALGAPSLLLTGTQLYGR	N-Term(iTRAQ4plex)	Unique	1.664E3	1.717E3	3.482E3	1.984E3	0	0.00133	3.21			0	3	722.74304	66.77	EV_set1_060319_050219_Frac5.raw
767994628																					vYLFLQPR	N-Term(iTRAQ4plex)	Unique	1.641E3	2.568E3	1.018E4	1.504E3	0.002	0.06777	1.72			0	3	393.90509	68.23	EV_set1_060319_050219_Frac3.raw
767994628																					vYLFLQPR	N-Term(iTRAQ4plex)	Unique	1.625E3	3.238E3	2.060E4	6.067E2	0	0.03554	1.94			0	3	393.90509	68.23	EV_set1_060319_050219_Frac3.raw
767994628																					nVGSQTLQTFk	N-Term(iTRAQ4plex); K11(iTRAQ4plex)	Unique	2.236E3	5.768E3	1.456E4		0.002	0.07411	1.91			0	3	504.28656	34.02	NIOP_Eset1_031019_Frac2.raw
767994628																					gPHALGAPSLLLTGTQLYGR	N-Term(iTRAQ4plex)	Unique	1.229E3	1.928E3	3.259E3		0	2.1E-05	4.73			0	3	722.74335	66.28	EV_set1_060319_050219_Frac5.raw
767994628																					gPHALGAPSLLLTGTQLYGR	N-Term(iTRAQ4plex)	Unique	1.254E3		3.542E3		0	0.03157	2.57			0	3	722.74335	66.29	EV_set1_060319_050219_Frac5.raw
767994628																					gPHALGAPSLLLTGTQLYGR	N-Term(iTRAQ4plex)	Unique		1.115E3	1.051E3	1.374E3	0	0.00951	2.49			0	3	722.74323	66.26	EV_set1_060319_050219_Frac5.raw
767994628																					nVGSQTLQTFk	N-Term(iTRAQ4plex); K11(iTRAQ4plex)	Unique		4.612E3	2.859E4	4.183E3	0.002	0.0807	1.92			0	2	755.92700	34.04	NIOP_Eset1_031019_Frac2.raw
768039830	2316	FLNA	PREDICTED: filamin-A isoform X5	9	19	GBC vs XGC	4.15	1.042	1.1E-03	1.040	2.9E-03	0.308	2.8E-15	3.557	3.227	34.4	26.5	48.2	52.4	59.8																	
768039830																					iVGPSGAAVPck	N-Term(iTRAQ4plex); C11(Methylthio); K12(iTRAQ4plex)	Unique	1.735E4	2.312E4	8.337E4	3.386E4	0.001	0.05943	3.12			0	3	478.26709	51.32	EV_set1_060319_050219_Frac3.raw
768039830																					iVGPSGAAVPck	N-Term(iTRAQ4plex); C11(Methylthio); K12(iTRAQ4plex)	Unique	1.646E4	1.540E4	4.562E4	2.408E4	0	0.03303	2.94			0	3	478.26709	51.32	EV_set1_060319_050219_Frac3.raw
768039830																					eTGEHLVHVk	N-Term(iTRAQ4plex); K10(iTRAQ4plex)	Unique	1.526E4	1.197E4	5.028E4	1.936E4	0.008	0.1795	1.91			0	4	359.95755	13.20	NIOP_Eset1_031019_Frac6.raw
768039830																					fnGTHIPGSPFk	N-Term(iTRAQ4plex); N2(Deamidated); K12(iTRAQ4plex)	Unique	1.698E4	1.201E4	3.622E4	1.913E4	0	0.0236	2.90			0	3	530.95599	47.84	NIOP_Eset1_031019_Frac6.raw
768039830																					lkPGAPLRPk	N-Term(iTRAQ4plex); K2(iTRAQ4plex); K10(iTRAQ4plex)	Unique	1.540E4	1.027E4	7.073E4	1.617E4	0.003	0.1009	2.11			0	4	378.00531	16.15	NIOP_Eset1_031019_Frac6.raw
768039830																					vTVLFAGQHIAk	N-Term(iTRAQ4plex); K12(iTRAQ4plex)	Unique	7.049E3	8.476E3	3.548E4	1.006E4	0	0.0136	2.48			0	3	524.65515	48.87	EV_set1_060319_050219_Frac5.raw
768039830																					vTYTPmAPGSYLISIk	N-Term(iTRAQ4plex); M6(Oxidation); K16(iTRAQ4plex)	Unique	8.181E3	9.840E3	2.080E4	8.427E3	0.004	0.1171	1.89			0	3	682.37817	69.34	EV_set1_060319_050219_Frac3.raw
768039830																					vTVLFAGQHIAk	N-Term(iTRAQ4plex); K12(iTRAQ4plex)	Unique	1.065E4	6.632E3	4.454E4	8.271E3	0	0.00123	3.45			0	3	524.65515	48.87	EV_set1_060319_050219_Frac5.raw
768039830																					fnGTHIPGSPFk	N-Term(iTRAQ4plex); N2(Deamidated); K12(iTRAQ4plex)	Unique	2.387E3	3.066E3	1.555E4	6.576E3	0.002	0.08385	1.90			0	3	530.95563	49.20	NIOP_Eset1_031019_Frac6.raw
768039830																					fnGTHIPGSPFk	N-Term(iTRAQ4plex); N2(Deamidated); K12(iTRAQ4plex)	Unique	3.011E3	1.534E3	1.683E4	6.497E3	0	0.02206	2.14			0	3	530.95557	49.39	NIOP_Eset1_031019_Frac6.raw
768039830																					vTYTPmAPGSYLISIk	N-Term(iTRAQ4plex); M6(Oxidation); K16(iTRAQ4plex)	Unique	8.492E3	6.262E3	1.918E4	6.458E3	0.001	0.04531	1.96			0	3	682.37817	69.34	EV_set1_060319_050219_Frac3.raw
768039830																					nGHVGISFVPk	N-Term(iTRAQ4plex); N1(Deamidated); K11(iTRAQ4plex)	Unique	5.338E3	6.951E3	5.189E4	5.404E3	0	0.02392	2.02			0	3	481.94455	44.37	EV_set1_060319_050219_Frac5.raw
768039830																					iVGPSGAAVPck	N-Term(iTRAQ4plex); C11(Methylthio); K12(iTRAQ4plex)	Unique	3.940E3	3.959E3	2.078E4	5.038E3	0	0.022	2.31			0	3	478.26804	50.96	EV_set1_060319_050219_Frac4.raw
768039830																					aHVVPcFDASk	N-Term(iTRAQ4plex); C6(Methylthio); K11(iTRAQ4plex)	Unique	3.382E3	1.515E3	1.357E4	3.188E3	0.003	0.1094	1.41			0	3	503.25961	44.80	EV_set1_060319_050219_Frac5.raw
768039830																					iVGPSGAAVPck	N-Term(iTRAQ4plex); C11(Methylthio); K12(iTRAQ4plex)	Unique	1.278E3	3.324E3	1.075E4	2.926E3	0.006	0.1563	1.61			0	3	478.26804	50.99	EV_set1_060319_050219_Frac4.raw
768039830																					yGGPYHIGGSPFk	N-Term(iTRAQ4plex); K13(iTRAQ4plex)	Unique		9.220E3	5.144E4	1.286E4	0	0.01183	2.81			0	3	556.63019	42.09	NIOP_Eset1_031019_Frac6.raw
768039830																					vTYTPMAPGSYLISIk	N-Term(iTRAQ4plex); K16(iTRAQ4plex)	Unique		2.361E3	1.392E4	1.836E3	0	0.00955	2.51			0	3	677.04730	76.15	EV_set1_060319_050219_Frac3.raw

768039830																					yGGPYHIGGSPFk	N-Term(iTRAQ4plex); K13(iTRAQ4plex)	Unique		9.433E3	4.430E4	1.055E4	0	0.02065	2.49			0	3	556.63037	41.91	NIOP_Eset1_031019_Frac6.raw
768039830																					vTYTPMAPGSYLISIk	N-Term(iTRAQ4plex); K16(iTRAQ4plex)	Unique		2.461E3	7.959E3	3.339E3	0	0.03441	1.71			0	3	677.04730	76.15	EV_set1_060319_050219_Frac3.raw
960139558	210	ALAD	delta-aminolevulinic acid dehydratase isoform c	1	2	GBC vs Healthy	3.11	2.758	1.0E-01	0.748	8.7E-01	0.881	6.2E-01	3.207	0.827	27.8	32.3	82.0	46.8	137.9																	
960139558																					cVLIFGVPSR	N-Term(iTRAQ4plex); C1(Methylthio)	Unique	2.079E3	1.064E4	6.381E3	8.393E3	0.001	0.04484	2.56			0	2	640.85205	81.28	EV_set1_060319_050219_Frac3.raw
960139558																					cVLIFGVPSR	N-Term(iTRAQ4plex); C1(Methylthio)	Unique	1.774E3	4.109E3	9.919E3	4.958E3	0.003	0.1017	2.03			0	2	640.85223	81.35	EV_set1_060319_050219_Frac3.raw
1009287437	83854	ANGPTL6	angiopoietin-related protein 6	1	2	GBC vs Healthy	1.49	6.591	5.4E-02	1.697	1.2E-02	0.941	3.6E-01	7.173	1.755	121.6	6.2	16.6	157.6	10.3																	
1009287437																					fHQLDVk	N-Term(iTRAQ4plex); K7(iTRAQ4plex)	Unique	1.213E4	2.219E4	5.288E4	5.119E4	0.002	0.06793	2.20			0	3	392.23135	20.88	NIOP_Eset1_031019_Frac6.raw
1009287437																					fHQLDVk	N-Term(iTRAQ4plex); K7(iTRAQ4plex)	Unique	9.965E2	7.166E3	1.961E4	1.521E4	0.003	0.1065	1.83			0	3	392.23172	32.40	EV_set1_060319_050219_Frac5.raw
1034575756	83706	FERMT3	PREDICTED: fermitin family homolog 3 isoform X2	1	3	GBC vs XGC	1.96	1.583	2.3E-02	0.978	6.9E-02	0.366	1.6E-04	4.090	2.600	11.4	16.9	5.8	17.7	5.8																	
1034575756																					vVLAGGVAPALFR	N-Term(iTRAQ4plex)	Unique	3.427E3	5.800E3	2.037E4	6.606E3	0	0.00872	2.80			0	2	707.43842	73.60	EV_set1_060319_050219_Frac3.raw
1034575756																					vVLAGGVAPALFR	N-Term(iTRAQ4plex)	Unique	1.575E3	1.793E3	7.054E3	3.277E3	0	0.01372	2.77			0	3	471.96146	73.60	EV_set1_060319_050219_Frac3.raw
1034575756																					vVLAGGVAPALFR	N-Term(iTRAQ4plex)	Unique	1.015E3	1.416E3	5.359E3	1.807E3	0	0.00485	2.69			0	3	471.96130	73.59	EV_set1_060319_050219_Frac3.raw
1034589520	102	ADAM10	PREDICTED: disintegrin and metalloproteinase domain-containing protein 10 isoform X1	1	3	GBC vs XGC	1.78	1.196	1.4E-01	1.091	1.4E-01	0.319	1.6E-03	3.552	3.094	11.6	24.2	13.1	25.0	9.2																	
1034589520																					lPPPkPLPGTLk	N-Term(iTRAQ4plex); K5(iTRAQ4plex); K12(iTRAQ4plex)	Unique	2.646E4	2.253E4	9.537E4	5.099E4	0	0.02044	2.87			0	3	564.03748	39.83	NIOP_Eset1_031019_Frac6.raw
1034589520																					lPPPkPLPGTLk	N-Term(iTRAQ4plex); K5(iTRAQ4plex); K12(iTRAQ4plex)	Unique	1.978E4	1.873E4	9.076E4	2.665E4	0.002	0.08319	2.36			0	3	564.03748	39.35	NIOP_Eset1_031019_Frac6.raw
1034589520																					lPPPkPLPGTLk	N-Term(iTRAQ4plex); K5(iTRAQ4plex); K12(iTRAQ4plex)	Unique	1.275E4	1.534E4	6.907E4	1.858E4	0.002	0.08027	1.90			0	3	564.03815	40.54	NIOP_Eset1_031019_Frac6.raw
1034613548	339761	CYP27C1	PREDICTED: cytochrome P450 27C1 isoform X1	1	3	GBC vs Healthy	1.49	10.794	8.0E-03	1.026	4.0E-02	1.202	2.2E-01	8.978	0.936	499.1	10.4	7.3	534.7	8.0																	
1034613548																					iHEIQqk	N-Term(iTRAQ4plex); Q6(Deamidated); K7(iTRAQ4plex)	Unique	1.602E4	5.699E5	6.427E5	7.109E5	0.001	0.05429	3.06			0	3	395.56696	13.54	NIOP_Eset1_031019_Frac6.raw
1034613548																					iHEIQqk	N-Term(iTRAQ4plex); Q6(Deamidated); K7(iTRAQ4plex)	Unique	4.208E4	9.806E4	1.410E5	1.638E5	0	0.02881	3.06			0	3	395.56668	13.72	NIOP_Eset1_031019_Frac6.raw
1034613548																					iHEIQqk	N-Term(iTRAQ4plex); Q6(Deamidated); K7(iTRAQ4plex)	Unique		9.450E3	1.288E4	1.264E4	0.006	0.1603	1.44			0	3	395.56714	20.75	EV_set1_060319_050219_Frac5.raw
1034625758	7529	YWHAB	PREDICTED: 14-3-3 protein beta/alpha isoform X2	1	3	GBC vs XGC	7.55	0.964	5.4E-01	0.938	2.4E-01	0.233	3.7E-03	3.643	3.648	22.6	19.7	8.8	62.3	10.7																	
1034625758																					nLLSVAYk	N-Term(iTRAQ4plex); K8(iTRAQ4plex)	Unique	3.082E4	2.541E4	1.450E5	3.110E4	0	0.0069	3.41			0	2	598.36780	51.23	EV_set1_060319_050219_Frac3.raw
1034625758																					nLLSVAYk	N-Term(iTRAQ4plex); K8(iTRAQ4plex)	Unique	2.614E4	1.733E4	7.373E4	3.068E4	0.001	0.06545	2.65			0	2	598.36804	51.27	EV_set1_060319_050219_Frac3.raw
1034625758																					nLLSVAYk	N-Term(iTRAQ4plex); K8(iTRAQ4plex)	Unique	5.669E3	7.389E3	3.924E4	7.930E3	0.003	0.1109	1.70			0	3	399.24738	51.28	EV_set1_060319_050219_Frac3.raw
1034664657	23307	FKBP15	PREDICTED: FK506-binding protein 15 isoform X3	1	3	GBC vs XGC	1.31	1.249	6.3E-02	1.314	7.8E-02	2.458	3.5E-02	0.542	0.520	4.0	46.5	78.7	10.3	25.6																	
1034664657																					iLLYISqqqPVTVAR	N-Term(iTRAQ4plex); Q7(Deamidated); Q8(Deamidated); Q9(Deamidated)	Unique	2.215E4	1.911E4	1.448E4	3.277E4	0.002	0.09161	2.30			0	3	626.02313	45.38	EV_set1_060319_050219_Frac5.raw
1034664657																					iLLYISqqqPVTVAR	N-Term(iTRAQ4plex); Q7(Deamidated); Q8(Deamidated); Q9(Deamidated)	Unique	7.156E3	8.120E3	5.013E3	2.055E4	0.004	0.1292	1.93			0	3	626.02246	46.69	EV_set1_060319_050219_Frac5.raw
1034664657																					iLLYISqqqPVTVAR	N-Term(iTRAQ4plex); Q7(Deamidated); Q8(Deamidated); Q9(Deamidated)	Unique	1.137E4	1.359E4	1.220E4	1.728E4	0.007	0.1635	1.31			0	3	626.02313	45.39	EV_set1_060319_050219_Frac5.raw

Supplementary Table S5: List of plasma-derived EV proteins with altered levels in advanced stage GBC. A total of 57 EV proteins (≥2.0 fold change) were identified as detailed in Methods section. The Table describes the total number of proteins and peptides identified in mass spectrometry runs along with their quantity values.

Accession	Description	Gene Symbol	Gene ID	Protein name	Differential w.r.t. controls	Differential in advanced stage GBC	Σ# Peptides	Σ# PSMs	Fold change GBC stage IIIA vs Healthy R1: 116/114	Fold change GBC stage IIIA vs Healthy R2: 116/114	p value: GBC-3A vs Healthy	Fold change GBC stage IIIA vs GSD R1: 116/115	Fold change GBC stage IIIA vs GSD R2: 116/115	p value: GBC-3A vs GSD	Fold change GBC stage IVB vs Healthy R1: 117/114	Fold change GBC stage IVB vs Healthy R2: 117/114	p value: GBC-4B vs Healthy	Fold change GBC stage IVB vs GSD R1: 117/115	Fold change GBC stage IVB vs GSD R2: 117/115	p value: GBC-4B vs GSD	GBC stage IIIA vs GSD R1: 116/115 Variability [%]	GBC stage IIIA vs GSD R2: 116/115 Variability [%]	GBC stage IIIA vs Healthy R1: 116/114 Variability [%]	GBC stage IIIA vs Healthy R2: 116/114 Variability [%]	GBC stage IVB vs Healthy R1: 117/114 Variability [%]	GBC stage IVB vs Healthy R2: 117/114 Variability [%]	GBC stage IVB vs GSD R1: 117/115 Variability [%]	GBC stage IVB vs GSD R2: 117/115 Variability [%]	MW [kDa]	calc. pI	Accession	Sequence	# PSMs	Modifications	MH+ [Da]	R1: 116/114	R2: 116/114	R1: 116/115	R2: 116/115	R2: 117/114	R1: 117/114	R1: 117/115	R2: 117/115	R1: 116/114 Variability [%]	R2: 116/114 Variability [%]	R1: 117/115 Variability [%]	R2: 117/115 Variability [%]	R1: 116/115 Variability [%]	R2: 116/115 Variability [%]	R1: 117/114 Variability [%]	R2: 117/114 Variability [%]	q-Value	PEP	Xcorr R1	IonScore R1	Exp Value R1	Xcorr R2	IonScore R2	Exp Value R2	# Missed Cleavages
4502027	NP_000468.1	ALB	213	serum albumin preproprotein	GBC IVB/Healthy	IVB specific	45	3420	0.556	0.561	0.0E+00	1.004	0.961	4.6E-07	**0.459**	**0.469**	0.0E+00	0.830	0.763	0.0E+00	28.4	13.9	21.8	20.5	29.4	26.3	31.0	15.1	69.3	6.28	4502027																													
4502027																															4502027	AACLLPK	34	N-Term(iTRAQ4plex); C3(Methylthio); K7(iTRAQ4plex)	1049.60942	0.560	0.532	0.925	0.991	0.396	0.437	0.766	0.709	10.2	48.0	9.4	5.9	5.7	18.8	9.6	23.1	1.0E-03	2.2E-02	2.9	33	6.7E-02	3.0	34	5.6E-02	0
4502027																															4502027	AAFTECCQAADK	61	N-Term(iTRAQ4plex); C6(Methylthio); C7(Methylthio); K12(iTRAQ4plex)	1637.70659	0.545	0.606	1.278	1.234	0.537	0.492	1.130	1.082	8.3	10.3	15.2	14.1	23.4	11.1	8.2	11.9	0.0E+00	1.8E-09	5.5	71	1.0E-06	5.7	74	4.7E-07	0
4502027																															4502027	AAFTECCQAADK	3	N-Term(iTRAQ4plex); C6(Methylthio); C7(Methylthio); Q8(Deamidated); K12(iTRAQ4plex)	1638.69707	0.501	0.590	1.326	0.660	0.660	0.446	1.213	0.719									0.0E+00	1.6E-03	3.6	20	1.4E-01	1.8			0
4502027																															4502027	ADDKETCFAEEGK	122	N-Term(iTRAQ4plex); K4(iTRAQ4plex); C7(Methylthio); K13(iTRAQ4plex)	1920.90825	0.548	0.545	0.939	0.939	0.486	0.384	0.704	0.773	20.9	15.5	12.4	6.5	10.7	7.8	25.8	18.4	0.0E+00	1.5E-06	6.8	82	4.0E-07	7.2	59	7.6E-05	1
4502027																															4502027	AEFAEVSK	194	N-Term(iTRAQ4plex); K8(iTRAQ4plex)	1168.64690	0.577	0.611	0.937	0.958	0.502	0.447	0.777	0.768	19.7	18.9	11.8	13.1	8.3	10.6	32.6	30.6	0.0E+00	1.7E-06	4.0	66	3.1E-05	3.9	66	3.1E-05	0
4502027																															4502027	AVMDDFAAFVEK	279	N-Term(iTRAQ4plex); M3(Oxidation); K12(iTRAQ4plex)	1646.83708	0.591	0.547	0.793	0.896	0.458	0.459	0.616	0.741	28.6	19.6	22.3	18.1	16.8	13.8	27.8	31.8	0.0E+00	1.5E-06	4.9	88	1.5E-07	5.0	88	1.5E-07	0
4502027																															4502027	AVMDDFAAFVEK	90	N-Term(iTRAQ4plex); K12(iTRAQ4plex)	1630.84184	0.526	0.491	0.826	0.906	0.442	0.407	0.608	0.732	8.0	19.2	33.4	15.2	24.0	8.4	26.7	31.7	0.0E+00	5.3E-08	5.7	81	8.7E-07	5.5	94	4.4E-08	0
4502027																															4502027	CASIQK	1	N-Term(iTRAQ4plex); C1(Methylthio); K6(iTRAQ4plex)	983.52660		0.718		1.212	0.448			0.737									6.0E-03	5.5E-02					26	7.6E-02	0
4502027																															4502027	CCAAADPHECYAK	8	N-Term(iTRAQ4plex); C1(Methylthio); C2(Methylthio); C10(Methylthio); K13(iTRAQ4plex)	1807.70505	0.524	0.883	1.600	1.222	0.614	0.553	1.812	0.827	2.2		210.9		184.0		0.0		0.0E+00	1.5E-06	4.2	31	3.6E-03	3.1	29	5.5E-03	0
4502027																															4502027	CCTESLVNR	10	N-Term(iTRAQ4plex); C1(Methylthio); C2(Methylthio); N8(Deamidated)	1261.51750	0.696	0.552	1.764	1.288	0.473	0.720	1.910	1.076	4.3		11.1		8.3		8.6		0.0E+00	2.0E-06	3.2	26	1.7E-02	4.1	33	3.3E-03	0
4502027																															4502027	CCTESLVNR	49	N-Term(iTRAQ4plex); C1(Methylthio); C2(Methylthio)	1260.53349	0.563	0.545	2.497	1.161	0.448	0.587	2.705	0.922	16.5	10.7	54.8	20.6	60.9	23.5	10.4	20.9	0.0E+00	9.7E-06	3.9	36	2.2E-03	3.9	39	1.2E-03	0
4502027																															4502027	DDNPNLPR	318	N-Term(iTRAQ4plex)	1084.55217	0.620	0.587	0.838	0.885	0.491	0.488	0.713	0.745	36.9	25.8	19.2	14.6	16.2	13.7	40.3	31.9	0.0E+00	3.9E-03	3.5	45	2.0E-03	3.4	44	2.4E-03	0
4502027																															4502027	DDNPNLPR	34	N-Term(iTRAQ4plex); N5(Deamidated)	1085.53569	0.667	0.664	0.865	0.885	0.522	0.535	0.695	0.717	12.6	25.5	13.1	11.6	10.7	16.0	17.3	18.3	0.0E+00	3.0E-04	3.6	46	1.3E-03	3.5	55	2.0E-04	0
4502027																															4502027	DLGEENFK	153	N-Term(iTRAQ4plex); K8(iTRAQ4plex)	1239.64690	0.629	0.645	0.799	1.015	0.517	0.635	0.824	0.812	23.9	20.1	10.2	10.2	9.5	7.5	19.9	31.1	0.0E+00	2.1E-03	3.8	53	4.2E-04	3.9	47	2.0E-03	0
4502027																															4502027	DLGEENFK	40	N-Term(iTRAQ4plex); N6(Deamidated); K8(iTRAQ4plex)	1240.63249	0.656	0.637	0.719	1.024	0.541	0.610	0.679	0.784	11.7	8.3	7.2	4.5	14.1	4.8	13.3	11.4	0.0E+00	1.1E-04	3.7	41	7.7E-03	3.5	42	6.5E-03	0
4502027																															4502027	DVFLGMFLYEYAR	4	N-Term(iTRAQ4plex); M6(Oxidation)	1783.88567	0.456		1.510			0.306	1.039		9.4		6.8		0.4		3.0		0.0E+00	1.0E-04	4.2	55	2.7E-04				0
4502027																															4502027	ECCEKPLLEK	29	N-Term(iTRAQ4plex); C2(Methylthio); C3(Methylthio); K5(iTRAQ4plex); K10(iTRAQ4plex)	1715.85788	0.618	0.646	1.074	1.244	0.540	0.484	0.878	1.068	9.0	15.8	8.8	3.8	7.5	6.6	14.4	10.6	0.0E+00	9.7E-05	4.0	16	2.4E+00	4.2	38	1.7E-02	0
4502027																															4502027	EFNAETFTFHADICTLSEK	9	N-Term(iTRAQ4plex); C14(Methylthio); K19(iTRAQ4plex)	2537.19614	0.598	0.676	1.155	0.895	0.488	0.289	0.572	0.717	28.5	50.3	6.2	9.6	27.2	12.7	7.4	31.1	0.0E+00	4.6E-05	4.5	43	3.7E-03	5.3	41	6.4E-03	0
4502027																															4502027	ETCFAEEGK	48	N-Term(iTRAQ4plex); C3(Methylthio); K9(iTRAQ4plex)	1347.61980	0.541	0.540	1.132	1.016	0.407	0.524	1.136	0.683	18.1	16.6	6.9	15.1	9.6	7.2	6.9	30.9	0.0E+00	1.5E-06	4.0	62	1.5E-05	4.2	71	2.2E-06	0
4502027																															4502027	ETYGEMADCCAK	67	N-Term(iTRAQ4plex); M6(Oxidation); C9(Methylthio); C10(Methylthio); K12(iTRAQ4plex)	1716.67107	0.560	0.555	0.952	1.207	0.496	0.504	0.874	0.976	9.8	15.7	15.9	9.2	19.6	12.9	16.9	20.6	0.0E+00	1.5E-06	4.4	76	4.9E-08	4.9	98	3.0E-10	0
4502027																															4502027	ETYGEMADCCAK	60	N-Term(iTRAQ4plex); C9(Methylthio); C10(Methylthio); K12(iTRAQ4plex)	1700.67217	0.567	0.596	1.074	1.149	0.497	0.528	0.899	0.934	11.2	16.1	7.4	10.7	13.9	8.0	21.1	15.5	0.0E+00	1.6E-09	5.5	84	7.2E-09	5.6	89	3.0E-09	0
4502027																															4502027	FKDLGEENFK	7	N-Term(iTRAQ4plex); K2(iTRAQ4plex); N8(Deamidated); K10(iTRAQ4plex)	1659.89701	0.592	0.770	0.947	1.000	0.722	0.289	0.482	0.913	6.3		5.5		2.2		18.2		0.0E+00	2.5E-05	5.7	30	9.9E-02	4.8			1
4502027																															4502027	FKDLGEENFK	30	N-Term(iTRAQ4plex); K2(iTRAQ4plex); K10(iTRAQ4plex)	1658.91326	0.532	0.525	1.156	0.843	0.469	0.285	0.668	0.755	9.0	6.1	7.8	4.7	15.7	15.7	23.4	8.6	0.0E+00	4.6E-04	5.8	57	2.5E-04	6.0	56	2.5E-04	1
4502027																															4502027	FQNALLVR	4	N-Term(iTRAQ4plex); Q2(Deamidated); N3(Deamidated)	1106.63152	0.643	0.561	0.960	0.951	0.590	0.490	0.750	0.974									0.0E+00	3.5E-04	3.2	32	8.5E-02	3.3	36	3.1E-02	0
4502027																															4502027	FQNALLVR	62	N-Term(iTRAQ4plex); N3(Deamidated)	1105.64958	0.520	0.524	1.169	0.945	0.413	0.363	0.796	0.743	3.7	13.5	13.5	10.9	18.7	9.9	18.5	18.9	0.0E+00	3.1E-02	3.3	45	4.0E-03	3.6	43	5.4E-03	0
4502027																															4502027	FQNALLVR	76	N-Term(iTRAQ4plex)	1104.66667	0.482	0.500	2.128	0.966	0.353	0.357	1.599	0.675	9.6	6.2	17.9	8.0	26.0	6.7	20.5	8.6	0.0E+00	9.5E-03	3.6	48	1.8E-03	3.6	45	3.0E-03	0
4502027																															4502027	HPDYSVVLLLR	4	N-Term(iTRAQ4plex)	1455.84500	0.448	0.592	1.131	0.910	0.547	0.455	1.179	0.818									0.0E+00	1.2E-03	3.4	32	5.9E-02	2.7	27	2.1E-01	0
4502027																															4502027	HPYFYAPELLFFAK	7	N-Term(iTRAQ4plex); K14(iTRAQ4plex)	2031.09903	0.326		0.659			0.479	0.832		6.8		0.1		26.5		9.4		0.0E+00	4.0E-04	3.2	25	4.6E-01				0
4502027																															4502027	KLVAASQAALGL	2	N-Term(iTRAQ4plex); K1(iTRAQ4plex)	1429.90107	0.690		1.189			0.389	0.690										0.0E+00	8.3E-03	4.9	43	1.9E-03				1
4502027																															4502027	KQTALVELVK	29	N-Term(iTRAQ4plex); K1(iTRAQ4plex); K10(iTRAQ4plex)	1561.00797	0.619	0.595	1.103	0.980	0.592	0.340	0.692	0.854	15.8	16.2	13.6	15.3	10.6	4.0	18.7	23.5	0.0E+00	1.3E-02	4.7	45	4.7E-04	4.9	49	1.7E-04	1
4502027																															4502027	KVPQVSTPTLVEVSR	2	N-Term(iTRAQ4plex); K1(iTRAQ4plex); Q4(Deamidated)	1929.13126		0.593		0.897	0.588			0.867									0.0E+00	1.5E-06				6.1	61	3.7E-05	1
4502027																															4502027	KVPQVSTPTLVEVSR	86	N-Term(iTRAQ4plex); K1(iTRAQ4plex)	1928.14377	0.612	0.631	1.024	1.051	0.519	0.577	0.823	0.844	25.0	20.7	11.9	15.2	11.7	14.1	36.7	43.8	0.0E+00	1.5E-06	6.3	60	3.4E-05	5.9	76	8.6E-07	1
4502027																															4502027	KYLYEIAR	21	N-Term(iTRAQ4plex); K1(iTRAQ4plex)	1343.79572	0.562	0.527	0.925	0.898	0.489	0.361	0.597	0.783	8.6	7.9	8.2	6.2	7.5	6.0	15.9	21.5	0.0E+00	9.3E-03	4.1	32	6.0E-02	4.2	33	4.6E-02	1

4502027																														4502027	LCTVATLR	25	N-Term(iTRAQ4plex); C2(Methylthio)	1066.58818	0.492	0.475	1.785	0.955	0.349	0.375	1.354	0.693	7.2	18.0	27.5	6.2	28.9	5.5	19.7	11.2	0.0E+00	4.7E-02	3.4	33	3.1E-02	3.4	35	2.0E-02	0
4502027																														4502027	LDELRDEGK	12	N-Term(iTRAQ4plex); K9(iTRAQ4plex)	1362.74980	0.624	0.608	1.111	0.961	0.523	0.420	0.787	0.747	12.2	18.1	19.0	4.6	13.7	14.2	18.2	2.2	1.0E-03	2.7E-02	3.8	43	6.5E-03	3.8	33	6.1E-02	1
4502027																														4502027	LVAASQAALGL	88	N-Term(iTRAQ4plex)	1157.70317	0.643	0.625	0.948	0.941	0.548	0.512	0.957	0.765	5.5	37.8	37.3	16.3	41.7	16.8	46.5	43.2	0.0E+00	1.7E-03	3.0	42	3.7E-03	3.3	48	8.7E-04	0
4502027																														4502027	LVAASQAALGL	10	N-Term(iTRAQ4plex); Q6(Deamidated)	1158.68645	0.378	0.649	1.040	0.882	0.587	0.358	1.011	0.730		4.4		8.1		7.3		2.5	0.0E+00	1.1E-05	2.6	52	3.8E-04	3.0	68	9.3E-06	0
4502027																														4502027	LVNEVTEFAK	75	N-Term(iTRAQ4plex); N3(Deamidated); K10(iTRAQ4plex)	1438.80315	0.567	0.665	1.138	0.946	0.554	0.472	0.875	0.810	20.0	19.8	15.1	13.2	12.2	10.8	33.3	26.5	0.0E+00	1.5E-06	4.3	89	1.2E-07	4.4	85	3.7E-07	0
4502027																														4502027	LVNEVTEFAK	78	N-Term(iTRAQ4plex); K10(iTRAQ4plex)	1437.82207	0.651	0.548	1.291	0.902	0.440	0.459	0.979	0.707	9.3	20.9	11.8	8.6	9.4	8.9	9.1	19.6	0.0E+00	1.5E-06	4.7	90	1.1E-07	4.5	90	9.7E-08	0
4502027																														4502027	LVRPEVDVMCTAFHDN EETFLK	4	N-Term(iTRAQ4plex); C10(Methylthio); K22(iTRAQ4plex)	2927.43886	0.374		0.876			0.324	0.778		24.4		79.9		21.5		76.0		0.0E+00	4.3E-05	3.8	29	1.8E-01				0
4502027																														4502027	LVRPEVDVMCTAFHDN EETFLK	2	N-Term(iTRAQ4plex); M9(Oxidation); C10(Methylthio); K22(iTRAQ4plex)	2943.43081	0.458		1.467			0.218	0.718										0.0E+00	1.7E-03	2.2	13	6.4E+00				0
4502027																														4502027	LVTDLTK	85	N-Term(iTRAQ4plex); K7(iTRAQ4plex)	1077.67742	0.548	0.541	1.095	0.909	0.443	0.443	0.869	0.728	19.1	16.0	11.1	5.4	11.4	13.5	25.0	17.5	0.0E+00	3.6E-03	3.0	55	1.4E-04	2.9	55	1.2E-04	0
4502027																														4502027	NECFLQHK	60	N-Term(iTRAQ4plex); C3(Methylthio); K8(iTRAQ4plex)	1352.67888	0.526	0.534	1.583	0.954	0.413	0.434	1.309	0.783	6.9	16.7	19.8	8.1	19.4	13.3	10.4	19.5	0.0E+00	3.9E-02	3.6	39	7.9E-03	3.7	38	9.7E-03	0
4502027																														4502027	NECFLQHK	11	N-Term(iTRAQ4plex); C3(Methylthio); Q6(Deamidated); K8(iTRAQ4plex)	1353.65439	0.580	0.576	1.117	0.888	0.492	0.390	0.863	0.733	2.3	14.1	19.5	6.8	2.6	11.6	19.3	5.9	0.0E+00	5.8E-03	3.4	28	1.0E-01	2.2	20	6.1E-01	0
4502027																														4502027	NYAEAK	1	N-Term(iTRAQ4plex); N1(Deamidated); K6(iTRAQ4plex)	984.52379		0.656		0.876	0.595			0.773									6.0E-03	1.7E-01				2.3			0
4502027																														4502027	QNCELFEQLGEYK	22	N-Term(iTRAQ4plex); C3(Methylthio); K13(iTRAQ4plex)	1934.92534	0.595	0.596	1.285	0.960	0.510	0.444	1.024	0.790	7.5	12.8	16.2	16.6	3.7	27.0	16.0	18.3	0.0E+00	2.4E-09	6.1	76	1.5E-06	5.6	83	3.1E-07	0
4502027																														4502027	QNCELFEQLGEYK	7	N-Term(iTRAQ4plex); N2(Deamidated); C3(Methylthio); K13(iTRAQ4plex)	1935.91092		0.574		0.800	0.478			0.641		20.0		34.3		8.1		24.0	0.0E+00	1.5E-06				3.5	32	3.2E-02	0
4502027																														4502027	QTALVELVK	105	N-Term(iTRAQ4plex); K9(iTRAQ4plex)	1288.81096	0.621	0.606	1.170	0.990	0.460	0.518	1.038	0.720	16.4	22.5	10.7	8.3	16.4	9.3	17.4	23.2	0.0E+00	1.5E-06	4.6	64	1.9E-05	4.5	65	1.6E-05	0
4502027																														4502027	QTALVELVK	14	N-Term(iTRAQ4plex); Q1(Deamidated); K9(iTRAQ4plex)	1289.79350	0.533	0.547	1.346	0.959	0.407	0.449	1.126	0.694	4.2	15.5	22.8	1.3	11.7	1.7	11.4	15.0	0.0E+00	1.9E-04	4.3	65	1.7E-05	3.7	40	5.2E-03	0
4502027																														4502027	RHPDYSVVLLLR	4	N-Term(iTRAQ4plex)	1611.94626	0.743		0.864			0.782	0.934		37.1		18.3		24.0		31.1		0.0E+00	1.1E-04	5.8	58	1.4E-04				1
4502027																														4502027	RHPYFYAPELLFFAK	15	N-Term(iTRAQ4plex); K15(iTRAQ4plex)	2187.20340	0.459		1.278			0.326	0.812		18.7		35.8		39.0		21.5		0.0E+00	6.8E-03	4.8	28	2.1E-01				1
4502027																														4502027	RPCFSALEVDETYVPK	42	N-Term(iTRAQ4plex); C3(Methylthio); K16(iTRAQ4plex)	2188.10556	0.668	0.649	0.929	0.988	0.501	0.550	0.781	0.806	25.5	20.6	22.2	18.9	12.4	18.9	46.7	30.3	0.0E+00	7.0E-07	5.4	34	5.3E-02	5.4	39	1.7E-02	0
4502027																														4502027	SHCIAEVENDEMPADLP SLAADFVESK	11	N-Term(iTRAQ4plex); C3(Methylthio); K27(iTRAQ4plex)	3251.51181	0.542	0.501	1.174	0.884	0.530	0.410	0.912	0.911	46.5	22.2	7.3	21.0	35.0	27.4	29.9	26.2	0.0E+00	1.5E-11	7.7	78	1.2E-06	6.9	69	8.1E-06	0
4502027																														4502027	SHCIAEVENDEMPADLP SLAADFVESK	32	N-Term(iTRAQ4plex); C3(Methylthio); M12(Oxidation); K27(iTRAQ4plex)	3267.51186	0.515	0.563	1.007	0.913	0.511	0.515	0.830	0.716	42.5	19.8	28.8	33.7	19.7	20.9	31.2	9.9	0.0E+00	8.6E-16	7.7	79	1.1E-06	8.2	77	1.5E-06	0
4502027																														4502027	SHCIAEVENDEMPADLP SLAADFVESK	6	N-Term(iTRAQ4plex); C3(Methylthio); N9(Deamidated); M12(Oxidation); K27(iTRAQ4plex)	3268.51865	0.533		0.920			0.419	0.562		23.7		16.1		14.2		20.4		0.0E+00	3.1E-08	5.1	40	8.0E-03				0
4502027																														4502027	SLHTLFGDK	83	N-Term(iTRAQ4plex); K9(iTRAQ4plex)	1305.74016	0.501	0.486	0.792	0.927	0.439	0.448	0.767	0.753	18.5	22.2	11.6	11.7	10.7	15.7	19.6	15.6	0.0E+00	2.6E-04	3.6	60	1.4E-04	3.2	27	2.4E-01	0
4502027																														4502027	TCVADESAENCDK	39	N-Term(iTRAQ4plex); C2(Methylthio); N10(Deamidated); C11(Methylthio); K13(iTRAQ4plex)	1765.70268	0.569	0.515	1.252	1.026	0.476	0.460	1.153	0.864	23.7	21.3	7.3	8.1	14.6	17.5	14.4	17.4	0.0E+00	1.6E-07	5.7	101	3.7E-10	5.9	98	9.6E-10	0
4502027																														4502027	TCVADESAENCDK	132	N-Term(iTRAQ4plex); C2(Methylthio); C11(Methylthio); K13(iTRAQ4plex)	1764.71892	0.550	0.584	1.380	1.074	0.491	0.453	1.255	0.857	33.1	18.2	13.4	11.8	12.7	11.8	27.1	19.3	0.0E+00	2.2E-07	5.7	98	9.8E-10	6.1	97	1.1E-09	0
4502027																														4502027	TYETTLEK	174	N-Term(iTRAQ4plex); K8(iTRAQ4plex)	1272.69512	0.525	0.573	1.671	1.042	0.431	0.387	1.276	0.764	22.4	13.7	11.4	9.3	18.4	8.2	20.1	13.5	0.0E+00	4.3E-03	3.8	59	1.1E-04	3.8	59	1.0E-04	0
4502027																														4502027	VFDEFKPLVEEPQNLIK	6	N-Term(iTRAQ4plex); K6(iTRAQ4plex); N14(Deamidated); K17(iTRAQ4plex)	2478.38907	0.740	0.712	1.701	1.086	0.546	0.578	1.365	0.907		2.2		29.6		6.8		12.2	0.0E+00	1.5E-03	1.2			4.4	23	3.6E-01	0
4502027																														4502027	VFDEFKPLVEEPQNLIK	22	N-Term(iTRAQ4plex); K6(iTRAQ4plex); K17(iTRAQ4plex)	2477.40525	0.423	0.501	1.316	0.895	0.342	0.342	1.156	0.601	17.7	8.1	46.9	7.4	4.4	15.6	12.9	11.5	0.0E+00	4.5E-09	4.3	35	2.2E-02	6.9	34	2.7E-02	0
4502027																														4502027	VHTECCHGDLLECADD R	10	N-Term(iTRAQ4plex); C5(Methylthio); C6(Methylthio); C13(Methylthio)	2197.84110	0.537	0.630	1.268	1.062	0.620	0.575	1.392	1.018	11.6		18.0		24.5		16.4		0.0E+00	6.1E-07	5.4	40	5.4E-04	4.1	27	9.9E-03	0
4502027																														4502027	VPQVSTPTLVEVSR	63	N-Term(iTRAQ4plex)	1655.94744	0.527	0.528	1.079	0.932	0.397	0.492	0.982	0.681	11.7	15.8	12.5	11.1	13.2	10.3	20.1	18.5	0.0E+00	1.5E-06	4.9	64	4.5E-05	5.0	64	4.4E-05	0
4502027																														4502027	YICENQDSISSK	140	N-Term(iTRAQ4plex); C3(Methylthio); K12(iTRAQ4plex)	1720.81450	0.510	0.477	1.057	0.921	0.385	0.446	0.887	0.734	13.2	16.6	21.3	7.8	15.1	10.7	27.3	16.2	0.0E+00	1.5E-06	5.4	85	1.9E-07	5.3	85	2.1E-07	0
4502027																														4502027	YICENQDSISSK	76	N-Term(iTRAQ4plex); C3(Methylthio); N5(Deamidated); K12(iTRAQ4plex)	1721.79851	0.523	0.525	0.962	0.883	0.441	0.401	0.748	0.693	8.1	20.5	16.2	10.1	5.6	11.0	23.9	26.7	0.0E+00	1.5E-06	5.2	98	7.2E-09	5.1	93	2.4E-08	0
4502027																														4502027	YLYEIAR	23	N-Term(iTRAQ4plex)	1071.59624	0.496	0.471	1.087	0.901	0.381	0.405	0.915	0.746	14.1	5.6	4.1	7.8	7.6	9.4	19.6	19.4	3.0E-03	2.3E-02	2.7	26	2.7E-01	2.7	26	2.9E-01	0
4502337	NP_001176.1	AZGP1	563	zinc-alpha-2-glycoprotein precursor	GBC IVB/Healthy	IVB specific	6	21	0.675	**0.539**	2.4E-06	0.890	0.962	1.3E-02	**0.534**	**0.478**	5.4E-09	0.740	0.868	1.0E-07	12.2	7.4	10.3	10.5	5.4	4.1	11.8	1.0	34.2	6.05	4502337																												
4502337																														4502337	EIPAWVPFDPAAQITK	4	N-Term(iTRAQ4plex); K16(iTRAQ4plex)	2071.14914	0.483	0.539	0.957	1.012	0.478	0.312	0.635	0.874									0.0E+00	1.6E-05	3.3	39	1.2E-02	2.4	15	3.4E+00	0
4502337																														4502337	IDVHWTR	2	N-Term(iTRAQ4plex)	1070.58762		0.502		0.962	0.465			0.868									5.0E-03	3.8E-02				2.3	26	1.3E-01	0
4502337																														4502337	WEAEPVYVQR	7	N-Term(iTRAQ4plex)	1420.73491	0.711	0.655	0.821	0.898	0.534	0.550	0.723	0.714	3.5		13.8		0.4		3.8		0.0E+00	2.0E-02	2.3	19	1.1E+00	2.9	18	1.6E+00	0

4502337																															4502337	EDIFMETLK	2	N-Term(iTRAQ4plex); K9(iTRAQ4plex)	1413.75359	0.573		1.069			0.511	0.979										0.0E+00	2.3E-04	3.5	56	3.2E-04				0
4502337																															4502337	EDIFMETLK	2	N-Term(iTRAQ4plex); M5(Oxidation); K9(iTRAQ4plex)	1429.74956	0.718		1.064			0.532	0.809										0.0E+00	9.8E-04	2.5	45	2.8E-03				0
4502337																															4502337	AYLEEECPATLR	2	N-Term(iTRAQ4plex); C7(Methylthio)	1584.75298	0.608		0.831			0.530	0.744										0.0E+00	3.5E-06	4.5	57	1.3E-04				0
4502337																															4502337	DYIEFNK	2	N-Term(iTRAQ4plex); K7(iTRAQ4plex)	1216.64885	0.689		0.767			0.644	0.737										4.0E-03	6.3E-02	2.5	24	3.6E-01				0
4504073	NP_000398.1	GP1BB	2812	platelet glycoprotein Ib beta chain precursor	GBC IIIA/Healthy; GBC IVB vs Healthy	IIIA and IVB	4	10	**0.176**	**0.233**	3.2E-07	1.018	1.339	2.3E-01	**0.199**	**0.213**	1.7E-06	1.183	1.360	7.7E-01	49.5	5.3	2.5	49.1	22.2	27.9	74.4	25.1	21.7	9.31	4504073																													
4504073																															4504073	LSLTDPLVAER	3	N-Term(iTRAQ4plex)	1357.78508	0.173	0.123	0.742	1.325	0.177	0.172	0.756	1.856									0.0E+00	7.0E-03	3.6			3.2	37	1.5E-02	0
4504073																															4504073	AWLAGRPER	1	N-Term(iTRAQ4plex)	1199.67844		0.278		1.258	0.353			1.557									0.0E+00	5.2E-02				3.3			0
4504073																															4504073	LLPYLAEDELR	4	N-Term(iTRAQ4plex)	1475.82634		0.268		1.490	0.212			1.151		49.1		4.7		14.4		27.9	0.0E+00	1.5E-06				3.7	60	1.1E-04	0
4504073																															4504073	CVAPPALR	2	N-Term(iTRAQ4plex); C1(Methylthio)	1016.55339	0.179		1.396			0.231	1.851										2.0E-03	3.8E-02	2.5	25	2.1E-01				0
4504323	NP_003539.1	HIST2H4A	8370	histone H4	GBC IIIA/Healthy	IIIA specific	3	6	**2.895**	**2.194**	3.8E-04	1.361	1.233	1.1E-03	**2.214**	1.854	7.2E-03	1.069	1.015	2.3E-03	2.4		4.6		15.0		12.7		11.4	11.36	4504323																													
4504323																															4504323	DAVTYTEHAK	2	N-Term(iTRAQ4plex); K10(iTRAQ4plex)	1422.74942		2.194		1.233	1.854			1.015									0.0E+00	1.5E-06				3.5	29	1.6E-01	0
4504323																															4504323	ISGLIYEETR	2	N-Term(iTRAQ4plex)	1324.71709	2.987		1.384			2.448	1.165										0.0E+00	1.4E-03	3.7	41	9.0E-03				0
4504323																															4504323	VFLENVIR	2	N-Term(iTRAQ4plex)	1133.67912	2.806		1.339			2.002	0.981										0.0E+00	6.9E-03	2.9	24	3.8E-01				0
4505733	NP_002610.1	PF4	5196	platelet factor 4 precursor	GBC IVB/Healthy, GSD	IVB specific	4	32	1.504	1.349	7.8E-04	1.539	1.430	1.2E-04	**4.798**	**4.084**	1.9E-11	**5.055**	**4.680**	3.6E-11	14.3	33.0	9.4	36.2	25.8	65.1	43.7	35.4	10.8	8.62	4505733																													
4505733																															4505733	ICLDLQAPLYK	8	N-Term(iTRAQ4plex); C2(Methylthio); K11(iTRAQ4plex)	1610.88974	1.521	1.739	1.425	1.701	6.200	4.576	4.402	5.748		15.8		1.0		15.0		8.1	0.0E+00	5.1E-03	2.3	44	5.0E-03	4.1	65	3.5E-05	0
4505733																															4505733	HITSLEVIK	13	N-Term(iTRAQ4plex); K9(iTRAQ4plex)	1327.81804	1.552	1.347	1.686	1.808	4.009	5.193	6.005	5.392	6.6	33.3	16.4	23.4	5.4	4.2	13.8	65.4	0.0E+00	6.5E-04	3.8	53	2.4E-04	3.5	30	4.6E-02	0
4505733																															4505733	TTSQVRPR	4	N-Term(iTRAQ4plex)	1088.62909		1.351		1.581	4.160			4.619		9.7		3.9		2.7		19.3	0.0E+00	6.0E-03				2.4	18	1.1E+00	0
4505733																															4505733	AGPHCPTAQLIATLK	7	N-Term(iTRAQ4plex); C5(Methylthio); K15(iTRAQ4plex)	1855.01548	1.037	0.851	0.853	1.156	1.435	2.876	2.430	1.812		37.0		7.8		4.3		11.2	0.0E+00	1.5E-05	5.2	29	1.5E-01	2.7	19	1.6E+00	0
4505981	NP_002695.1	PPBP	5473	platelet basic protein preproprotein	GBC IIIA/Healthy	IIIA specific	2	6	**0.397**	**0.425**	1.1E-03	1.155	0.824	9.4E-01	0.545	0.586	2.8E-04	1.625	1.108	8.8E-01	4.0		9.9		12.7		6.7		13.9	8.79	4505981																													
4505981																															4505981	ICLDPDAPR	2	N-Term(iTRAQ4plex); C2(Methylthio)	1189.58464		0.425		0.824	0.586			1.108									0.0E+00	6.0E-04				3.2	29	5.2E-02	0
4505981																															4505981	NIQSLEVIGK	4	N-Term(iTRAQ4plex); K10(iTRAQ4plex)	1388.83513	0.397		1.155			0.545	1.625		9.9		6.7		4.0		12.7		0.0E+00	8.1E-06	4.4	78	1.5E-06				0
4506179	NP_002777.1	PSMA1	5682	proteasome subunit alpha type-1 isoform 2	GBC IVB/Healthy	IVB specific	3	16	1.794	1.610	1.7E-07	1.282	1.279	1.6E-03	**2.140**	**1.967**	1.1E-06	1.618	1.632	1.1E-02	26.3	14.3	14.5	32.3	12.8	38.6	17.5	11.2	29.5	6.61	4506179																													
4506179																															4506179	AQPAQPADEPAEK	12	N-Term(iTRAQ4plex); K13(iTRAQ4plex)	1639.85344	1.935	1.791	1.210	1.342	2.265	2.278	1.487	1.645	7.3	22.0	9.7	6.8	17.1	7.4	18.6	28.5	0.0E+00	1.5E-06	4.0	50	1.3E-03	4.0	55	4.8E-04	0
4506179																															4506179	ETLPAEQDLTTK	2	N-Term(iTRAQ4plex); K12(iTRAQ4plex)	1633.87772		1.147		1.012	1.530			1.314									1.0E-03	8.1E-03				2.2	24	5.3E-01	0
4506179																															4506179	LVSLIGSK	2	N-Term(iTRAQ4plex); K8(iTRAQ4plex)	1104.72185	1.664		3.078			1.917	3.641										0.0E+00	2.1E-03	3.5	68	3.0E-06				0
4506193	NP_002784.1	PSMB1	5689	proteasome subunit beta type-1	GBC IVB/Healthy	IVB specific	6	18	1.674	1.559	6.3E-07	1.064	1.168	2.6E-01	**2.154**	**1.955**	2.0E-05	1.416	1.573	5.7E-01	16.9	32.4	16.0	17.3	18.8	36.2	30.6	21.0	26.5	8.13	4506193																													
4506193																															4506193	DVYTGDALR	2	N-Term(iTRAQ4plex)	1153.59770		1.559		1.168	2.478			1.809									0.0E+00	1.4E-05				3.4	26	2.8E-01	0
4506193																															4506193	GAVYSFDPVGSYQR	8	N-Term(iTRAQ4plex)	1689.83489	1.782	1.581	0.980	1.261	2.279	2.282	1.166	1.770	17.3		2.4		9.4		36.1		0.0E+00	4.8E-05	4.3	61	5.4E-05	4.8	58	1.0E-04	0
4506193																															4506193	EETVSLR	2	N-Term(iTRAQ4plex)	977.53734		1.750		1.445	1.955			1.573									4.0E-03	1.5E-01				2.5	31	5.1E-02	0
4506193																															4506193	LSEGFSIHTR	2	N-Term(iTRAQ4plex)	1290.69193		1.089		0.933	1.413			1.179									0.0E+00	6.3E-06				3.7	19	1.1E+00	0
4506193																															4506193	NMQNVEHVPLSLDR	2	N-Term(iTRAQ4plex); M2(Oxidation)	1811.91812		1.119		0.789	1.383			0.950									0.0E+00	1.5E-06				4.1	36	2.8E-02	0
4506193																															4506193	DVFISAAER	2	N-Term(iTRAQ4plex)	1151.61565	1.573		1.296			2.033	1.719										0.0E+00	9.3E-04	3.3	28	2.1E-01				0
4506381	NP_002863.1	RAC2	5880	ras-related C3 botulinum toxin substrate 2	GBC IVB/Healthy	IVB specific	2	3	**0.434**	0.621	1.2E-01	0.703	1.206	7.0E-01	**0.440**	**0.533**	1.5E-02	0.731	1.008	2.5E-01									21.4	7.61	4506381																													
4506381																															4506381	TVFDEAIR	1	N-Term(iTRAQ4plex)	1094.59636		0.621		1.206	0.533			1.008									5.0E-03	1.6E-01				2.4			0
4506381																															4506381	LAPITYPQGLALAK	2	N-Term(iTRAQ4plex); K14(iTRAQ4plex)	1744.06455	0.434		0.703			0.440	0.731										0.0E+00	4.5E-03	2.3	17	5.4E-01				0
4507651	NP_003281.1	TPM4	7171	tropomyosin alpha-4 chain isoform Tpm4.2cy	GBC IVB/Healthy	IVB specific	5	20	0.582	0.806	4.7E-03	0.998	**2.352**	7.0E-03	**0.474**	**0.433**	4.6E-07	0.837	1.438	1.2E-01	41.4	40.7	66.1	5.5	34.2	18.0	42.0	14.9	28.5	4.69	4507651																													
4507651																															4507651	LVILEGELER	2	N-Term(iTRAQ4plex)	1314.77934		1.024		2.033	0.848			1.640									1.0E-03	6.8E-02				2.3	22	3.9E-01	0

4507651																															4507651	SLEAASEK	8	N-Term(iTRAQ4plex); K8(iTRAQ4plex)	1122.62883	1.025	0.816	1.447	2.722	0.399	0.724	1.050	1.508		3.7		3.2		29.4		10.1	0.0E+00	6.7E-03	2.4	40	9.4E-03	3.5	48	1.9E-03	0
4507651																															4507651	TIDDLEEK	4	N-Term(iTRAQ4plex); K8(iTRAQ4plex)	1250.67558	0.773	0.790	0.838	3.374	0.330	0.547	0.609	1.371									0.0E+00	2.0E-03	2.6	33	6.8E-02	3.8	48	1.9E-03	0
4507651																															4507651	AEGDVAALNR	4	N-Term(iTRAQ4plex)	1159.62053	0.438	0.384	0.694	1.006	0.470	0.411	0.668	1.200									0.0E+00	1.5E-06	3.2	46	2.1E-03	3.4	48	1.3E-03	0
4507651																															4507651	IQALQQQADEAEDR	2	N-Term(iTRAQ4plex)	1758.87432	0.343		1.188			0.350	1.243										0.0E+00	7.0E-06	4.3	62	5.8E-05				0
4507877	NP_003364.1	VCL	7414	vinculin isoform VCL	GBC IVB/Healthy	IVB specific	15	39	0.595	0.630	2.4E-12	0.740	0.936	2.6E-03	**0.428**	**0.461**	5.0E-20	0.590	0.657	7.3E-17	18.4	10.6	5.7	13.9	9.4	26.8	15.2	21.1	116.6	6.09	4507877																													
4507877																															4507877	DYLIDGSR	3	N-Term(iTRAQ4plex)	1082.56182	0.609	0.574	0.934	0.912	0.525	0.433	0.682	0.812									0.0E+00	1.7E-02	2.8	21	7.3E-01		20	8.0E-01	0
4507877																															4507877	GALAEAR	2	N-Term(iTRAQ4plex)	831.48210		0.669		0.962	0.574			0.803									4.0E-03	1.4E-01				2.5	28	9.5E-02	0
4507877																															4507877	AVAGNISDPGLQK	2	N-Term(iTRAQ4plex); K13(iTRAQ4plex)	1557.88786		0.576		0.813	0.550			0.756									0.0E+00	1.5E-06				3.4	62	6.9E-05	0
4507877																															4507877	GVGQAAIR	4	N-Term(iTRAQ4plex)	915.55028	0.617	0.641	0.722	0.939	0.502	0.428	0.514	0.717									0.0E+00	3.7E-03	3.0	44	1.6E-03	2.4	21	3.3E-01	0
4507877																															4507877	ALASIDSK	4	N-Term(iTRAQ4plex); K8(iTRAQ4plex)	1092.65093	0.536	0.843	0.654	1.324	0.429	0.466	0.584	0.657									0.0E+00	1.9E-06	3.4	47	1.8E-03	3.1	68	1.7E-05	0
4507877																															4507877	AASDELSK	4	N-Term(iTRAQ4plex); K8(iTRAQ4plex)	1108.61174		0.637		0.922	0.437			0.617		1.6		24.5		2.3		25.2	0.0E+00	2.4E-05				3.2	53	4.3E-04	0
4507877																															4507877	STVEGIQASVK	2	N-Term(iTRAQ4plex); K11(iTRAQ4plex)	1406.81147		0.747		1.013	0.461			0.608									0.0E+00	8.4E-04				3.1	33	6.3E-02	0
4507877																															4507877	LGATAEK	2	N-Term(iTRAQ4plex); K7(iTRAQ4plex)	977.59056		0.533		1.005	0.319			0.586									2.0E-03	1.0E-01				2.7	38	2.4E-02	0
4507877																															4507877	AAAVGTANK	2	N-Term(iTRAQ4plex); K9(iTRAQ4plex)	1090.65105		0.506		0.848	0.259			0.423									0.0E+00	1.5E-06				2.7	55	5.1E-04	0
4507877																															4507877	DPSASPGDAGEQAIR	4	N-Term(iTRAQ4plex)	1614.78606	1.229	0.315	1.321	0.432	0.284	0.554	0.612	0.380									0.0E+00	1.5E-06	3.2	31	4.9E-02	3.4	67	1.4E-05	0
4507877																															4507877	TNLLQVCER	2	N-Term(iTRAQ4plex); C7(Methylthio)	1265.64775	0.595		1.051			0.402	0.729										0.0E+00	7.0E-03	3.5	24	3.1E-01				0
4507877																															4507877	SFLDSGYR	2	N-Term(iTRAQ4plex)	1088.55193	0.499		0.715			0.403	0.592										0.0E+00	5.2E-03	3.5	31	4.1E-02				0
4507877																															4507877	SLGEISALTSK	2	N-Term(iTRAQ4plex); K11(iTRAQ4plex)	1393.81830	0.525		0.740			0.408	0.590										0.0E+00	8.7E-04	3.0	28	1.7E-01				0
4507877																															4507877	AQQVSQGLDVLTAK	2	N-Term(iTRAQ4plex); K14(iTRAQ4plex)	1745.99979	0.618		0.657			0.488	0.533										0.0E+00	1.4E-04	5.2	49	1.1E-03				0
4507877																															4507877	ELTPQVVSAAR	2	N-Term(iTRAQ4plex)	1314.75054	0.581		0.866			0.339	0.519										0.0E+00	4.8E-03	3.1	38	1.5E-02				0
4758606	NP_004508.1	ILK	3611	integrin-linked protein kinase isoform 1	GBC IIIA/Healthy	IIIA specific	3	5	**0.502**	**0.474**	2.1E-02	0.827	1.277	7.1E-01	0.825	0.747	4.7E-02	1.397	**1.962**	2.8E-01		5.9		35.9		16.6		24.6	51.4	8.07	4758606																													
4758606																															4758606	HSGIDFK	1	N-Term(iTRAQ4plex); K7(iTRAQ4plex)	1091.61078		0.375		1.329	0.669			2.310									5.0E-03	1.7E-01				1.8			0
4758606																															4758606	DFNEECPR	2	N-Term(iTRAQ4plex); C6(Methylthio)	1199.49895		0.599		1.227	0.835			1.666									0.0E+00	2.0E-04				2.6	29	7.4E-03	0
4758606																															4758606	EGNAVAVR	2	N-Term(iTRAQ4plex)	959.54143	0.502		0.827			0.825	1.397										3.0E-03	5.1E-02	2.4	24	3.2E-01				0
4826762	NP_005134.1	HP	3240	haptoglobin isoform 1 preproprotein	GBC IIIA/Healthy	IIIA specific	11	824	**0.468**	**0.479**	1.2E-295	1.161	1.028	1.9E-02	0.636	0.677	1.2E-288	1.681	1.434	1.7E-03	31.5	15.1	23.1	28.6	15.3	14.6	42.6	28.9	45.2	6.58	4826762																													
4826762																															4826762	FTDHLK	2	N-Term(iTRAQ4plex); K6(iTRAQ4plex)	1048.60389		0.404		1.241	0.654			1.956		7.3		0.1		12.3		5.1	2.0E-03	9.7E-02				2.3			0
4826762																															4826762	YVMLPVADQDQCIR	30	N-Term(iTRAQ4plex); C12(Methylthio)	1840.88408	0.381	0.401	1.303	1.077	0.682	0.575	1.407	1.787	34.4	17.2	8.8	21.0	3.0	11.2	7.3	8.7	0.0E+00	1.5E-06	4.6	66	2.2E-05	4.4	68	1.3E-05	0
4826762																															4826762	TEGDGVYTLNNEK	40	N-Term(iTRAQ4plex); N10(Deamidated); N11(Deamidated); K13(iTRAQ4plex)	1729.84209	0.472	0.455	1.302	1.043	0.667	0.599	1.699	1.565	18.7	20.0	25.2	21.6	13.6	17.4	5.5	8.8	0.0E+00	7.5E-12	6.2	101	5.3E-09	6.1	101	5.3E-09	0
4826762																															4826762	TEGDGVYTLNNEK	178	N-Term(iTRAQ4plex); K13(iTRAQ4plex)	1727.87261	0.452	0.445	1.359	1.094	0.638	0.723	2.221	1.471	17.6	24.1	22.1	24.5	17.2	13.7	9.7	18.2	0.0E+00	1.5E-08	6.4	87	1.8E-07	6.2	87	2.0E-07	0
4826762																															4826762	VGYVSGWGR	43	N-Term(iTRAQ4plex)	1124.59905	0.451	0.424	1.118	0.947	0.671	0.566	1.527	1.456	28.7	25.7	34.8	16.0	19.1	9.5	15.3	11.0	0.0E+00	2.7E-05	3.3	35	1.8E-02	3.2	38	9.0E-03	0
4826762																															4826762	TEGDGVYTLNNEK	211	N-Term(iTRAQ4plex); N10(Deamidated); K13(iTRAQ4plex)	1728.85661	0.443	0.468	1.205	1.043	0.676	0.606	1.826	1.449	24.2	21.9	32.7	23.4	27.8	16.7	19.2	12.9	0.0E+00	1.5E-08	6.2	97	1.7E-08	6.2	102	5.9E-09	0
4826762																															4826762	HYEGSTVPEK	130	N-Term(iTRAQ4plex); K10(iTRAQ4plex)	1434.75005	0.432	0.483	1.091	1.019	0.654	0.626	1.681	1.416	30.9	30.9	35.3	26.4	21.8	13.7	11.3	12.8	0.0E+00	1.5E-06	3.6	51	7.6E-04	3.6	54	3.5E-04	0
4826762																															4826762	VTSIQDWVQK	50	N-Term(iTRAQ4plex); K10(iTRAQ4plex)	1491.84197	0.532	0.543	1.254	1.026	0.737	0.698	1.684	1.307	12.9	13.2	14.0	19.2	15.1	9.9	22.4	9.5	0.0E+00	1.5E-06	4.9	83	5.1E-07	4.8	88	1.6E-07	0
4826762																															4826762	YVMLPVADQDQCIR	75	N-Term(iTRAQ4plex); M3(Oxidation); C12(Methylthio)	1856.88425	0.489	0.595	0.868	1.021	0.760	0.775	1.460	1.280	51.2	53.8	37.1	44.1	20.0	16.3	32.5	18.1	0.0E+00	1.5E-06	4.6	56	1.8E-04	4.5	65	2.1E-05	0
4826762																															4826762	DYAEVGR	45	N-Term(iTRAQ4plex)	953.48180	0.512	0.505	0.739	0.879	0.652	0.641	0.901	1.086	24.4	26.6	29.3	19.9	11.7	15.9	15.0	9.1	1.0E-03	3.1E-02	3.1	30	9.0E-02	2.9	30	9.0E-02	0
4826762																															4826762	SPVGVQPILNEHTFCAGMSK	4	N-Term(iTRAQ4plex); C15(Methylthio); M18(Oxidation); K20(iTRAQ4plex)	2465.21646	0.423	0.587	0.910	0.834	0.573	0.456	1.008	0.793									0.0E+00	1.7E-05	4.4	46	3.5E-03	2.4	11	1.1E+01	0
4826762																															4826762	LPECEADDGCPKPPEIAHGYVEHSVR	3	N-Term(iTRAQ4plex); C4(Methylthio); C10(Methylthio); K12(iTRAQ4plex)	3228.48218	0.395		1.021			0.759	2.011		7.3		7.7		15.4		15.1		0.0E+00	2.0E-05	4.2	18	1.0E+00				0

4826762																															4826762	SPVGVQPILNEHTFCAGMSK	2	N-Term(iTRAQ4plex); Q6(Deamidated); N10(Deamidated); C15(Methylthio); K20(iTRAQ4plex)	2451.21842	0.411		0.810			0.659	1.333										0.0E+00	1.4E-02	3.5	30	1.2E-01				0
4826762																															4826762	SPVGVQPILNEHTFCAGMSK	2	N-Term(iTRAQ4plex); C15(Methylthio); K20(iTRAQ4plex)	2449.23905	0.600		1.106			0.592	1.119										0.0E+00	1.5E-05	5.4	33	6.3E-02				0
4826762																															4826762	VTSIQDWVQK	2	N-Term(iTRAQ4plex); Q9(Deamidated); K10(iTRAQ4plex)	1492.82773	0.634		0.927			0.659	0.989										0.0E+00	1.3E-02	2.8	22	7.5E-01				0
4826762																															4826762	SPVGVQPILNEHTFCAGMSK	2	N-Term(iTRAQ4plex); N10(Deamidated); C15(Methylthio); K20(iTRAQ4plex)	2450.20987	0.354		0.868			0.381	0.960										0.0E+00	4.4E-07	6.5	55	3.9E-04				0
4826762																															4826762	SPVGVQPILNEHTFCAGMSK	2	N-Term(iTRAQ4plex); N10(Deamidated); C15(Methylthio); M18(Oxidation); K20(iTRAQ4plex)	2466.20395	0.673		0.912			0.652	0.908										0.0E+00	8.0E-05	4.5	34	5.0E-02				0
4826762																															4826762	VVLHPNYSQVDIGLIK	2	N-Term(iTRAQ4plex); K16(iTRAQ4plex)	2083.22220	0.547		0.608			0.631	0.721										0.0E+00	1.3E-04	4.0	19	4.4E-01				0
4826762																															4826762	HYEGSTVPEKK	1	N-Term(iTRAQ4plex); K10(iTRAQ4plex); K11(iTRAQ4plex)	1706.94631	0.485		0.794			0.341	0.573										8.0E-03	1.2E-01	3.7						1
4826898	NP_005013.1	PFN1	5216	profilin-1	GBC IIIA/Healthy; GBC IVB vs Healthy	IIIA and IVB	2	11	**0.386**	**0.304**	7.8E-04	0.677	0.960	1.1E-01	**0.472**	**0.338**	6.1E-04	0.866	1.042	3.6E-02	3.2	8.9	32.9	16.3	26.6	10.8	16.5	3.4	15.0	8.27	4826898																													
4826898																															4826898	DSPSVWAAVPGK	9	N-Term(iTRAQ4plex); K12(iTRAQ4plex)	1501.83013	0.373	0.304	0.662	0.960	0.338	0.464	0.882	1.042	57.7	16.3	33.6	3.4	0.0	8.9	50.5	10.8	0.0E+00	1.5E-06	3.3	38	1.8E-02	3.9	54	4.2E-04	0
4826898																															4826898	SSFYVNGLTLGGQK	2	N-Term(iTRAQ4plex); N6(Deamidated); K14(iTRAQ4plex)	1759.95151	0.400		0.692			0.479	0.851										0.0E+00	4.9E-04	3.6	33	6.3E-02				0
5031635	NP_005498.1	CFL1	1072	cofilin-1	GBC IIIA/Healthy; GBC IVB vs Healthy	IIIA and IVB	2	5	**0.393**	**0.518**	7.3E-04	0.850	1.144	7.3E-01	**0.452**	**0.426**	9.8E-05	1.004	0.916	2.3E-02		13.2		5.0		22.2		3.8	18.5	8.09	5031635																													
5031635																															5031635	SSTPEEVK	3	N-Term(iTRAQ4plex); K8(iTRAQ4plex)	1164.64043		0.518		1.144	0.426			0.916		5.0		3.8		13.2		22.2	2.0E-03	1.1E-01				2.2	31	9.4E-02	0
5031635																															5031635	NIILEEGK	2	N-Term(iTRAQ4plex); K8(iTRAQ4plex)	1203.72087	0.393		0.850			0.452	1.004										4.0E-03	7.0E-02	1.4	18	1.9E+00				0
10800138	NP_066407.1	HIST1H2BD	3017	histone H2B type 1-D	GBC IIIA/Healthy	IIIA specific	2	10	**5.113**	**2.431**	1.5E-05	1.645	1.364	1.7E-04	**3.430**	1.805	1.3E-02	1.133	1.161	1.3E-02	2.2	2.8	26.7	32.7	29.7	25.4	0.6	22.1	13.9	10.32	10800138																													
10800138																															10800138	LLLPGELAK	8	N-Term(iTRAQ4plex); K9(iTRAQ4plex)	1241.80486	5.113	2.294	1.645	1.567	1.877	3.430	1.133	1.250	26.7	42.2	0.6	11.0	2.2	20.8	29.7	31.6	0.0E+00	2.1E-06	3.2	65	9.8E-06	3.1	58	5.3E-05	0
10800138																															10800138	QVHPDTGISSK	2	N-Term(iTRAQ4plex); K11(iTRAQ4plex)	1456.79908		2.431		1.339	1.805			0.968									0.0E+00	1.5E-06				4.4	48	2.3E-03	0
10800144	NP_066544.1	HIST1H2AJ	8331	histone cluster 1, H2aj	GBC IIIA/Healthy	IIIA specific	2	18	**2.399**	**2.693**	1.9E-12	1.568	1.344	4.0E-06	1.802	**2.006**	8.0E-08	1.159	0.994	5.9E-04	16.9	14.5	11.7	21.0	6.3	21.8	23.9	17.2	13.9	10.89	10800144																													
10800144																															10800144	NDEELNK	11	N-Term(iTRAQ4plex); K7(iTRAQ4plex)	1149.59697	2.308	2.780	1.620	1.356	2.132	1.748	1.278	1.013	11.8	25.6	18.3	32.9	9.7	13.5	3.6	19.4	0.0E+00	1.6E-02	2.9	48	1.3E-03	3.2	49	1.2E-03	0
10800144																															10800144	AGLQFPVGR	7	N-Term(iTRAQ4plex)	1088.63298	2.494	2.609	0.942	1.332	1.888	1.983	0.769	0.976		18.2		12.3		15.7		24.1	0.0E+00	1.1E-02	2.3			3.3	37	1.6E-02	0
11761629	NP_068657.1	FGA	2243	fibrinogen alpha chain isoform alpha precursor	GBC IVB/Healthy	IVB specific	31	4824	1.669	1.636	0.0E+00	1.075	1.152	6.7E-154	**2.020**	**2.143**	0.0E+00	1.321	1.457	6.1E-04	25.4	15.5	21.0	23.8	26.6	26.7	30.3	19.2	69.7	8.06	11761629																													
11761629																															11761629	EVDLKDYEDQQK	68	N-Term(iTRAQ4plex); K5(iTRAQ4plex); K12(iTRAQ4plex)	1942.01218	1.941	1.731	0.954	1.022	2.999	1.511	0.793	1.759	12.7	16.4	11.0	16.7	10.2	8.4	13.3	37.7	0.0E+00	4.8E-05	6.2	54	5.3E-04	6.4	66	3.0E-05	1
11761629																															11761629	AQLVDMK	55	N-Term(iTRAQ4plex); M6(Oxidation); K7(iTRAQ4plex)	1108.62773	1.831	1.926	0.833	1.551	2.502	2.100	0.982	1.700	9.8	50.5	11.5	8.0	7.4	15.4	24.8	35.2	1.0E-03	1.2E-02	2.3	32	4.8E-02	2.5	32	5.2E-02	0
11761629																															11761629	MADEAGSEADHEGTHSTK	69	N-Term(iTRAQ4plex); K18(iTRAQ4plex)	2160.97025	1.654	1.786	1.176	1.312	2.253	1.943	1.408	1.659	9.7	14.5	18.7	17.2	14.9	12.9	14.4	21.5	0.0E+00	2.7E-13	6.7	56	1.0E-04	7.3	91	2.2E-08	0
11761629																															11761629	NPSSAGSWNSGSSGPGSTGNR	194	N-Term(iTRAQ4plex)	2107.95342	1.839	1.830	1.761	1.237	2.538	2.353	2.322	1.652	20.9	17.5	27.7	16.2	21.8	13.1	16.4	16.8	0.0E+00	3.1E-09	7.2	129	4.2E-12	6.9	132	2.2E-12	0
11761629																															11761629	GDSTFESK	219	N-Term(iTRAQ4plex); K8(iTRAQ4plex)	1158.58806	1.655	1.630	1.091	1.215	2.276	2.059	1.364	1.621	16.4	25.2	15.9	21.6	12.8	17.2	24.7	33.9	0.0E+00	7.1E-04	3.7	60	4.0E-05	3.7	49	5.2E-04	0
11761629																															11761629	QHLPLIK	259	N-Term(iTRAQ4plex); K7(iTRAQ4plex)	1136.73941	1.728	1.602	1.351	1.185	2.112	2.059	1.655	1.582	17.2	17.1	15.7	13.9	14.3	14.3	22.2	19.0	0.0E+00	2.3E-02	3.0	50	3.0E-04	2.9	44	1.3E-03	0
11761629																															11761629	GSESGIFTNTK	140	N-Term(iTRAQ4plex); K11(iTRAQ4plex)	1428.75713	1.710	1.628	1.058	1.174	2.327	2.216	1.437	1.582	12.5	34.5	19.2	25.2	15.4	19.4	13.6	34.3	0.0E+00	1.5E-06	5.9	73	6.7E-06	5.7	76	3.2E-06	0
11761629																															11761629	EYHTEK	1	N-Term(iTRAQ4plex); K6(iTRAQ4plex)	1094.57132		1.655		1.132	2.349			1.566									9.0E-03	7.7E-02					14	2.7E+00	0
11761629																															11761629	TVIGPDGHK	325	N-Term(iTRAQ4plex); K9(iTRAQ4plex)	1211.69914	1.455	1.624	1.117	1.193	2.205	1.822	1.449	1.542	26.6	26.6	25.5	17.9	21.4	13.3	32.7	26.5	0.0E+00	2.9E-04	4.9	67	1.9E-05	4.9	63	5.2E-05	0
11761629																															11761629	GSESGIFTNTK	78	N-Term(iTRAQ4plex); N9(Deamidated); K11(iTRAQ4plex)	1429.73870	1.876	1.611	1.010	1.148	2.260	2.131	1.226	1.539	25.8	25.7	16.3	8.1	8.5	10.5	27.0	19.8	0.0E+00	1.5E-06	4.8	60	8.7E-05	5.4	61	7.5E-05	0
11761629																															11761629	NSLFEYQK	95	N-Term(iTRAQ4plex); K8(iTRAQ4plex)	1316.70854	1.927	1.803	2.295	1.205	2.284	2.482	2.903	1.531	27.3	17.2	30.0	10.5	30.5	6.4	29.4	22.1	0.0E+00	8.1E-04	3.9	54	4.2E-04	4.0	59	1.2E-04	0
11761629																															11761629	DYEDQQK	152	N-Term(iTRAQ4plex); K7(iTRAQ4plex)	1213.59465	1.672	1.885	1.155	1.259	2.299	2.152	1.541	1.524	17.1	15.4	16.3	7.7	10.2	10.6	23.3	13.5	0.0E+00	6.2E-03	3.5	38	8.1E-03	3.5	39	7.6E-03	0
11761629																															11761629	QFTSSTSYNR	104	N-Term(iTRAQ4plex)	1334.64714	1.865	1.895	2.127	1.293	2.270	2.386	2.738	1.522	11.1	11.9	21.6	8.3	25.8	8.7	10.6	9.4	0.0E+00	1.5E-06	4.2	42	3.2E-03	4.5	45	1.9E-03	0
11761629																															11761629	NSLFEYQK	82	N-Term(iTRAQ4plex); N1(Deamidated); K8(iTRAQ4plex)	1317.69280	1.957	1.755	1.031	1.174	2.343	2.175	1.109	1.520	41.7	19.8	16.5	8.3	25.7	10.2	35.2	24.0	0.0E+00	1.6E-06	4.1	67	1.9E-05	4.1	52	5.5E-04	0
11761629																															11761629	QLEQVIAK	101	N-Term(iTRAQ4plex); K8(iTRAQ4plex)	1216.74944	1.891	1.702	1.400	1.231	2.256	2.170	1.809	1.520	18.5	20.8	25.5	13.5	15.4	9.0	20.9	25.7	0.0E+00	3.3E-05	4.0	53	2.9E-04	3.8	53	3.7E-04	0
11761629																															11761629	GDFSSANNR	262	N-Term(iTRAQ4plex)	1111.52458	1.672	1.598	0.956	1.121	2.190	2.144	1.243	1.518	9.3	23.1	13.4	15.1	9.3	16.3	13.3	25.5	0.0E+00	2.8E-04	3.9	52	1.7E-04	3.8	49	3.5E-04	0
11761629																															11761629	DSDWPFCSDEDWNYK	64	N-Term(iTRAQ4plex); C7(Methylthio); K15(iTRAQ4plex)	2240.91875	1.914	1.841	1.093	1.171	2.546	2.296	1.339	1.518	30.0	21.9	15.4	22.0	37.1	24.8	11.1	24.2	0.0E+00	1.5E-06	4.7	54	3.6E-05	4.6	57	1.7E-05	0

11761629																															11761629	VTSGSTTTTR	147	N-Term(iTRAQ4plex)	1154.61418	1.799	1.850	2.790	1.245	2.297	2.447	3.764	1.516	6.3	9.0	38.4	7.0	28.0	9.1	10.7	12.4	0.0E+00	7.0E-04	3.9	38	1.4E-02	4.0	38	1.3E-02	0
11761629																															11761629	VPPEWK	72	N-Term(iTRAQ4plex); K6(iTRAQ4plex)	1043.61235	1.796	1.688	1.109	1.206	2.151	2.225	1.399	1.509	13.7	19.0	7.3	12.0	9.9	10.6	13.1	21.3	1.0E-03	2.6E-02		43	3.7E-03		43	4.3E-03	0
11761629																															11761629	NPSSAGSWNSGSSGPG STGNR	277	N-Term(iTRAQ4plex); N9(Deamidated)	2108.93682	1.747	1.704	1.508	1.181	2.176	2.180	1.949	1.504	14.7	15.1	22.6	18.1	17.7	14.3	20.7	18.1	0.0E+00	2.2E-10	7.1	126	7.0E-12	7.2	132	2.3E-12	0
11761629																															11761629	QFTSSTSYNR	75	N-Term(iTRAQ4plex); N9(Deamidated)	1335.63152	1.759	1.756	1.610	1.196	2.271	2.405	2.139	1.504	6.0	14.8	12.1	8.7	6.4	8.6	6.4	13.0	0.0E+00	1.9E-02	4.2	45	1.4E-03	4.5	52	2.7E-04	0
11761629																															11761629	AQLVDMK	25	N-Term(iTRAQ4plex); K7(iTRAQ4plex)	1092.63274	1.872	1.597	1.009	1.322	1.874	2.147	1.115	1.503	17.8	19.0	4.7	15.3	3.6	7.0	5.7	19.3	0.0E+00	3.2E-03	2.9	45	3.2E-03	2.9	45	3.0E-03	0
11761629																															11761629	EVVTSEDGSDCPEAMD LGTLSGIGTLDGFR	64	N-Term(iTRAQ4plex); C11(Methylthio); M15(Oxidation)	3277.47104	2.509	1.860	1.161	1.202	2.257	2.100	0.998	1.476		21.7		35.2		36.9		40.4	0.0E+00	1.4E-20	6.0	85	1.7E-07	6.5	93	1.7E-08	0
11761629																															11761629	GGSTSYGTGSETESPR	1	N-Term(iTRAQ4plex); Y6(iTRAQ4plex)	1860.87979		1.901		1.501	1.912			1.470									0.0E+00	1.5E-06					17	9.9E-01	0
11761629																															11761629	ALTDMPQMR	28	N-Term(iTRAQ4plex); M5(Oxidation); M8(Oxidation)	1238.59861	1.729	1.448	0.926	1.053	1.904	2.189	1.222	1.408	15.2	19.3	6.8	19.1	8.7	13.7	13.3	34.3	1.0E-03	1.4E-02	1.9	33	2.6E-02	2.1	33	2.6E-02	0
11761629																															11761629	ALTDMPQMR	72	N-Term(iTRAQ4plex); M5(Oxidation)	1222.60271	1.617	1.662	0.973	1.103	2.134	1.913	1.160	1.388	13.7	12.0	10.8	8.0	10.4	6.7	21.1	12.2	0.0E+00	4.7E-02	3.0	41	4.2E-03	2.7	42	3.9E-03	0
11761629																															11761629	HPDEAAFFDTASTGK	75	N-Term(iTRAQ4plex); K15(iTRAQ4plex)	1881.92082	1.693	1.470	1.032	1.203	1.754	2.211	1.334	1.387	27.3	19.5	15.2	19.4	14.8	11.3	29.2	27.6	0.0E+00	4.7E-09	5.5	105	2.8E-09	5.5	106	2.3E-09	0
11761629																															11761629	GDFSSANNR	176	N-Term(iTRAQ4plex); N8(Deamidated)	1112.51067	1.403	1.466	0.884	1.082	1.914	1.642	1.051	1.383	16.4	29.1	13.0	16.8	9.4	15.1	31.0	35.5	0.0E+00	3.2E-06	4.4	58	3.1E-05	4.4	49	2.9E-04	0
11761629																															11761629	DSDWPFCSDEDWNYK	10	N-Term(iTRAQ4plex); C7(Methylthio); N13(Deamidated); K15(iTRAQ4plex)	2241.90067	1.693	1.273	0.836	1.054	1.712	1.948	0.920	1.380	22.2		28.5		47.3		22.5		0.0E+00	1.0E-02	2.0	21	5.8E-02	1.5			0
11761629																															11761629	MELERPGGNEITR	77	N-Term(iTRAQ4plex); M1(Oxidation); N9(Deamidated)	1662.82523	1.521	1.451	1.023	1.064	1.943	1.738	1.114	1.377	17.4	25.5	7.7	11.6	13.7	11.6	13.5	34.9	0.0E+00	8.2E-03	3.5	37	1.5E-02	3.8	32	4.6E-02	0
11761629																															11761629	GLIDEVNQDFTNR	144	N-Term(iTRAQ4plex)	1664.83660	1.647	1.540	0.978	1.047	2.013	2.132	1.237	1.368	22.1	25.6	34.4	18.0	13.0	12.4	20.5	28.5	0.0E+00	1.7E-08	5.3	77	2.3E-06	5.4	79	1.5E-06	0
11761629																															11761629	GGSTSYGTGSETESPR	365	N-Term(iTRAQ4plex)	1716.78154	1.765	1.602	0.938	1.094	1.982	2.061	1.113	1.350	25.2	30.5	23.5	25.8	14.4	21.4	34.3	33.8	0.0E+00	1.5E-06	6.7	92	1.9E-08	6.9	100	3.8E-09	0
11761629																															11761629	GDFSSANNRDNTYNR	12	N-Term(iTRAQ4plex); N8(Deamidated)	1875.82614	1.308	1.162	0.755	0.886	1.811	1.832	1.085	1.330	25.7	3.1	33.8	1.7	4.2	12.0	3.4	7.4	0.0E+00	2.5E-03	3.7	29	3.5E-02	4.8	29	3.8E-02	1
11761629																															11761629	MELERPGGNEITR	128	N-Term(iTRAQ4plex); M1(Oxidation)	1661.83976	1.520	1.370	1.103	1.022	1.795	1.596	1.190	1.321	25.2	15.5	14.0	21.2	13.3	13.6	22.3	22.8	0.0E+00	2.6E-06	3.7	36	2.1E-02	3.6	36	2.6E-02	0
11761629																															11761629	ALTDMPQMR	51	N-Term(iTRAQ4plex)	1206.60845	1.663	1.504	1.047	1.041	1.992	2.019	1.268	1.308	29.4	22.5	7.3	20.0	14.0	14.5	29.5	25.1	0.0E+00	1.8E-03	3.8	39	8.5E-03	3.7	37	1.4E-02	0
11761629																															11761629	MELERPGGNEITR	50	N-Term(iTRAQ4plex); N9(Deamidated)	1646.82950	1.482	1.463	1.039	1.033	1.959	1.636	1.104	1.287	14.6	10.8	13.1	10.7	14.0	6.5	24.2	8.7	0.0E+00	1.5E-06	4.8	44	3.5E-03	4.9	42	5.0E-03	0
11761629																															11761629	VQHIQLLQK	37	N-Term(iTRAQ4plex); K9(iTRAQ4plex)	1394.87143	1.828	1.834	1.199	1.236	1.855	2.243	1.449	1.281	13.3	5.6	14.6	16.5	12.2	18.4	19.7	21.6	0.0E+00	6.0E-03	4.1	45	6.8E-04	4.3	45	7.7E-04	0
11761629																															11761629	MELERPGGNEITR	47	N-Term(iTRAQ4plex)	1645.84477	1.264	1.310	1.202	0.991	1.782	1.456	1.267	1.263	52.6	11.3	10.1	13.7	5.6	14.1	52.2	18.8	0.0E+00	1.5E-06	5.0	38	1.2E-02	4.9	50	8.8E-04	0
11761629																															11761629	MADEAGSEADHEGTH STK	85	N-Term(iTRAQ4plex); M1(Oxidation); K18(iTRAQ4plex)	2176.96255	1.408	1.342	1.034	1.085	1.787	1.644	1.195	1.258	15.5	34.7	29.5	26.0	24.1	19.4	21.9	34.4	0.0E+00	2.5E-09	5.9	58	5.8E-05	6.3	65	9.8E-06	0
11761629																															11761629	GLIDEVNQDFTNR	96	N-Term(iTRAQ4plex); Q8(Deamidated)	1665.82175	1.360	1.393	0.872	1.028	1.710	1.858	1.013	1.236	17.7	21.8	16.9	15.4	16.9	13.4	23.6	30.7	0.0E+00	3.5E-05	5.5	84	4.4E-07	5.4	71	8.2E-06	0
11761629																															11761629	GDFSSANNR	24	N-Term(iTRAQ4plex); N7(Deamidated); N8(Deamidated)	1113.49578	1.168	1.472	0.788	1.105	1.848	1.121	0.744	1.234	14.0	17.9	22.4	8.2	6.7	8.4	29.9	20.7	0.0E+00	2.2E-04	3.9	51	9.5E-05	4.0	49	1.2E-04	0
11761629																															11761629	NPSSAGSWNSGSSGPG STGNR	168	N-Term(iTRAQ4plex); N9(Deamidated); N20(Deamidated)	2109.92143	1.561	1.596	1.167	1.071	1.862	1.909	1.457	1.198	16.2	21.3	36.1	23.9	36.0	16.5	25.0	22.2	0.0E+00	6.5E-09	6.3	132	1.3E-12	7.0	129	2.1E-12	0
11761629																															11761629	MKPVPDLVPGNFK	86	N-Term(iTRAQ4plex); M1(Oxidation); K2(iTRAQ4plex); K13(iTRAQ4plex)	1890.08969	1.571	1.269	1.381	1.059	1.379	1.721	1.727	1.182	33.5	12.6	34.1	13.7	30.0	10.2	45.7	15.8	0.0E+00	1.5E-06	4.2	33	4.0E-02	3.6	24	2.9E-01	0
11761629																															11761629	MKPVPDLVPGNFK	22	M1(Oxidation); N-Term(iTRAQ4plex); K2(iTRAQ4plex); N11(Deamidated); K13(iTRAQ4plex)	1891.07444	1.701	1.294	1.180	1.029	1.488	1.690	1.443	1.170	10.0	14.9	14.1	7.4	8.4	6.1	16.0	14.9	0.0E+00	4.3E-05	3.7	29	1.1E-01	3.9	29	1.4E-01	0
11761629																															11761629	AQLVDMK	2	N-Term(iTRAQ4plex); Q2(Deamidated); K7(iTRAQ4plex)	1093.62016		1.385		1.203	1.369			1.158									2.0E-03	2.3E-02				3.0	40	1.0E-02	0
11761629																															11761629	GDFSSANNRDNTYNR	8	N-Term(iTRAQ4plex); N7(Deamidated); N8(Deamidated)	1876.81913	0.955	1.547	0.682	0.746	2.061	0.903	0.662	1.093		24.1		12.3		1.4		30.0	0.0E+00	7.7E-05	3.1	28	3.1E-02	4.9	33	1.1E-02	1
11761629																															11761629	MKPVPDLVPGNFK	21	N-Term(iTRAQ4plex); K2(iTRAQ4plex); N11(Deamidated); K13(iTRAQ4plex)	1875.07944	1.679	1.290	1.117	1.076	1.281	1.803	1.247	1.084	19.8	32.8	11.4	8.7	8.9	6.0	21.5	31.1	0.0E+00	1.9E-06	4.2	38	1.2E-02	4.0	40	8.2E-03	0
11761629																															11761629	DYEDQQK	2	N-Term(iTRAQ4plex); Q5(Deamidated); K7(iTRAQ4plex)	1214.57549		1.280		1.030	1.377			1.079									1.0E-03	7.6E-02				2.5	20	4.3E-01	0
11761629																															11761629	ESSSHHPGIAEFPSR	46	N-Term(iTRAQ4plex)	1781.86138	1.241	1.098	0.764	0.944	1.449	1.431	1.051	1.050	23.4	10.7	16.0	25.3	24.8	22.9	32.8	19.0	0.0E+00	2.9E-06	4.8	67	2.0E-05	4.6	34	4.5E-02	0
11761629																															11761629	MKPVPDLVPGNFK	15	N-Term(iTRAQ4plex); K2(iTRAQ4plex); K13(iTRAQ4plex)	1874.09012	1.810	1.599	1.380	1.108	1.466	2.308	1.692	0.989	16.2		21.3		4.3		15.9		0.0E+00	3.8E-04	4.1	29	7.1E-02	3.3	14	2.3E+00	0
11761629																															11761629	QFTSSTSYNRGDSTFES K	8	N-Term(iTRAQ4plex); N9(Deamidated); K18(iTRAQ4plex)	2331.09757	1.594	1.266	1.424	0.755	1.689	1.920	1.761	0.982	1.0	23.3	5.6	11.3	10.6	10.4	17.3	24.2	0.0E+00	5.2E-02	5.0	28	1.3E-01	3.5	22	4.9E-01	1
11761629																															11761629	DSHSLTTNIMEILR	14	N-Term(iTRAQ4plex); M10(Oxidation)	1789.92221	1.240	0.969	0.890	0.796	1.016	1.364	0.980	0.826	3.8	23.7	28.5	9.6	3.5	1.0	36.6	11.0	0.0E+00	1.5E-06	4.5	60	1.1E-04	4.5	37	2.4E-02	0
11761629																															11761629	EVVTSEDGSDCPEAMD LGTLSGIGTLDGFR	7	N-Term(iTRAQ4plex); C11(Methylthio)	3261.46909	1.761	1.155	0.866	0.695	1.100	2.772	1.401	0.645	26.4		18.5		2.0		9.6		0.0E+00	3.4E-11	6.9	102	2.9E-09	3.2	27	9.8E-02	0
11761629																															11761629	LKNSLFEYQK	6	N-Term(iTRAQ4plex); K2(iTRAQ4plex); N3(Deamidated); K10(iTRAQ4plex)	1702.97403	1.754		1.426			2.234	1.865		11.1		3.1		4.9		9.3		0.0E+00	2.3E-04	5.5	50	1.1E-03				1

11761629																															11761629	TFPGFFSPMLGEFVSETESR	12	N-Term(iTRAQ4plex); M9(Oxidation)	2425.15068	1.650		1.634			1.734	1.726		5.6		3.6		5.3		8.5		0.0E+00	2.5E-06	6.0	78	1.2E-06				0
11761629																															11761629	ALTDMPQMR	1	N-Term(iTRAQ4plex); Q7(Deamidated)	1207.58794	1.671		1.110			1.401	0.955										7.0E-03	1.0E-01	2.7						0
20149498	NP_000137.2	FTL	2512	ferritin light chain	GBC IVB/GSD	IVB specific	3	5	0.947	1.563	1.9E-01	0.568	**0.465**	2.0E-02	0.745	1.159	2.9E-01	**0.459**	**0.336**	1.0E-02		20.7		30.1		29.5		21.3	20.0	5.78	20149498																													
20149498																															20149498	LNQALLDLHALGSAR	2	N-Term(iTRAQ4plex)	1735.99289		1.281		0.534	0.954			0.387									0.0E+00	1.5E-06				4.3	29	1.4E-01	0
20149498																															20149498	LGGPEAGLGEYLFER	1	N-Term(iTRAQ4plex)	1751.91838		1.907		0.405	1.407			0.291									0.0E+00	2.8E-02				4.0			0
20149498																															20149498	KPAEDEWGK	2	N-Term(iTRAQ4plex); K1(iTRAQ4plex); K9(iTRAQ4plex)	1491.81031	0.947		0.568			0.745	0.459										0.0E+00	1.4E-02	3.0	21	9.0E-01				0
23110942	NP_002781.2	PSMA5	5686	proteasome subunit alpha type-5 isoform 1	GBC IVB/Healthy	IVB specific	3	10	1.660	1.694	2.2E-05	1.361	1.284	2.5E-04	**2.105**	**2.204**	8.9E-05	1.723	1.627	6.3E-03	17.8	32.5	2.0	50.1	6.1	45.1	13.6	27.9	26.4	4.79	23110942																													
23110942																															23110942	EELEEVIK	6	N-Term(iTRAQ4plex); K8(iTRAQ4plex)	1276.72344	1.649	2.331	1.282	1.590	2.946	2.062	1.647	1.957	1.0		6.8		8.8		3.1		0.0E+00	1.5E-04	4.1	40	1.0E-02	3.9	42	6.4E-03	0
23110942																															23110942	SSLIILK	2	N-Term(iTRAQ4plex); K7(iTRAQ4plex)	1061.71794	2.407	1.694	1.555	1.284	2.204	3.086	2.047	1.627									4.0E-03	6.0E-02	2.6			1.9			0
23110942																															23110942	ITSPLMEPSSIEK	2	N-Term(iTRAQ4plex); M6(Oxidation); K13(iTRAQ4plex)	1735.93375		1.157		1.030	1.399			1.214									0.0E+00	2.9E-06				4.0	24	5.7E-01	0
29550838	NP_057632.2	GOLM1	51280	Golgi membrane protein 1	GBC IVB/Healthy	IVB specific	5	15	0.565	0.550	9.1E-06	0.914	0.887	1.1E-01	**0.346**	**0.400**	1.6E-08	0.687	0.634	2.8E-06	0.7	7.7	47.4	0.7	4.1	17.6	22.8	11.0	45.3	4.97	29550838																													
29550838																															29550838	EETNEIQVVNEEPQR	2	N-Term(iTRAQ4plex)	1957.95792		0.592		0.842	0.658			0.912									0.0E+00	1.5E-06				3.5	42	5.5E-03	0
29550838																															29550838	IQSSHNFQLESVNK	2	N-Term(iTRAQ4plex); K14(iTRAQ4plex)	1919.03318		0.508		0.828	0.400			0.634									0.0E+00	1.5E-06				5.0	27	3.2E-01	0
29550838																															29550838	EQVVEDRPVGGR	6	N-Term(iTRAQ4plex)	1484.79609	0.766	0.549	0.910	0.909	0.380	0.563	0.687	0.613		0.3		6.0		3.6		10.0	0.0E+00	1.6E-06	3.0	18	2.0E+00	4.2	26	3.4E-01	0
29550838																															29550838	QAALAGNDR	1	N-Term(iTRAQ4plex)	1059.56963		0.550		1.141	0.114			0.230									1.0E-03	5.8E-02				2.9			0
29550838																															29550838	QAALAGNDR	2	N-Term(iTRAQ4plex); N7(Deamidated)	1060.55242	0.386		0.914			0.346	0.840										0.0E+00	6.6E-03	3.3	50	4.0E-04				0
29550838																															29550838	DQLVIPDGQEEEQEAAGEGR	2	N-Term(iTRAQ4plex)	2314.09531	0.565		0.966			0.336	0.590										0.0E+00	2.4E-06	5.4	48	1.5E-03				0
31377806	NP_002635.2	PIGR	5284	polymeric immunoglobulin receptor precursor	GBC IVB/Healthy, GSD	IVB specific	21	197	1.635	1.717	5.6E-80	1.774	1.783	3.3E-57	**2.833**	**3.034**	2.5E-73	**2.963**	**3.177**	5.1E-70	44.0	32.5	23.1	29.0	28.3	35.1	42.6	37.5	83.2	5.74	31377806																													
31377806																															31377806	IIEGEPNLK	19	N-Term(iTRAQ4plex); K9(iTRAQ4plex)	1300.77141	1.786	2.073	1.859	2.452	3.591	2.761	3.021	3.988	12.4	10.3	4.4	10.0	11.9	8.6	10.8	14.8	0.0E+00	1.5E-06	3.9	52	4.7E-04	3.5	65	2.5E-05	0
31377806																															31377806	FSSYEK	2	N-Term(iTRAQ4plex); K6(iTRAQ4plex)	1048.55535		1.959		2.119	3.561			3.751		69.2		68.8		74.5		63.6	8.0E-03	6.8E-02					34	2.7E-02	0
31377806																															31377806	AFVNCDENSR	8	N-Term(iTRAQ4plex); C5(Methylthio); N8(Deamidated)	1345.56474	1.512	2.111	1.476	2.159	3.763	2.358	2.365	3.748	8.6	23.2	16.7	29.8	27.3	28.7	1.6	24.3	0.0E+00	1.5E-06	3.7	42	4.2E-04	3.6	49	6.7E-05	0
31377806																															31377806	ILLNPQDK	18	N-Term(iTRAQ4plex); K8(iTRAQ4plex)	1228.74956	2.178	2.011	1.989	2.142	3.816	3.432	3.218	3.641	25.0	21.3	33.2	11.7	36.6	12.8	31.6	12.0	0.0E+00	5.2E-03	3.4	43	1.5E-03	3.4	41	2.5E-03	0
31377806																															31377806	ADAAPDEK	19	N-Term(iTRAQ4plex); K8(iTRAQ4plex)	1104.57536	1.915	1.699	1.540	1.842	3.300	3.281	2.939	3.579	3.7	27.1	6.7	20.9	32.6	15.2	15.2	31.3	0.0E+00	2.2E-05	2.8	46	2.3E-03	3.2	55	3.2E-04	0
31377806																															31377806	CGLGINSR	6	N-Term(iTRAQ4plex); C1(Methylthio); N6(Deamidated)	1010.48827	1.563	1.757	2.476	2.038	3.041	2.336	3.800	3.436	4.3		4.6		8.9		9.2		0.0E+00	2.2E-04	3.2	28	4.9E-02	2.9	31	2.3E-02	0
31377806																															31377806	WNNTGCQALPSQDEGPSK	4	N-Term(iTRAQ4plex); C6(Methylthio); K18(iTRAQ4plex)	2266.04660	1.791	1.651	1.521	1.635	3.432	2.585	2.254	3.310									0.0E+00	1.8E-12	5.5	48	8.7E-04	5.6	67	9.9E-06	0
31377806																															31377806	NADLQVLKPEPELVYEDLR	2	N-Term(iTRAQ4plex); Q5(Deamidated); K8(iTRAQ4plex)	2530.37148		1.251		2.001	2.100			3.273									0.0E+00	1.5E-06				2.0	16	3.2E+00	0
31377806																															31377806	TTVEIK	2	N-Term(iTRAQ4plex); K6(iTRAQ4plex)	978.60655	1.568	1.779	1.531	2.200	2.697	3.133	3.142	3.248									9.0E-03	2.1E-01		39	4.5E-03	2.5			0
31377806																															31377806	CGLGINSR	9	N-Term(iTRAQ4plex); C1(Methylthio)	1009.50432	1.994	1.635	5.126	1.924	2.827	3.638	9.604	3.242	11.5	22.3	33.3	36.4	25.9	40.9	18.6	18.1	0.0E+00	8.6E-03	3.1	34	1.8E-02	2.9	33	2.3E-02	0
31377806																															31377806	AFVNCDENSR	8	N-Term(iTRAQ4plex); C5(Methylthio)	1344.58037	1.884	2.264	1.447	1.898	3.945	3.375	2.662	3.220	2.2	0.7	11.4	5.1	8.3	4.9	0.8	0.9	0.0E+00	1.5E-06	3.8	47	2.3E-04	3.8	49	1.4E-04	0
31377806																															31377806	VLDSGFR	14	N-Term(iTRAQ4plex)	937.52172	1.572	1.537	2.004	1.695	3.028	2.809	3.459	3.114	13.8	6.5	20.1	35.5	16.9	7.3	11.8	21.0	2.0E-03	8.2E-02	3.1	29	1.4E-01	2.9	27	2.2E-01	0
31377806																															31377806	NADLQVLKPEPELVYEDLR	13	N-Term(iTRAQ4plex); K8(iTRAQ4plex)	2529.38100	1.629	1.733	1.810	1.887	2.994	3.252	3.709	3.088	34.9	10.3	41.1	13.7	67.0	20.9	11.8	3.1	0.0E+00	1.3E-09	4.6	36	2.8E-02	5.5	38	1.7E-02	0
31377806																															31377806	QSSGENCDVVVNTLGK	12	N-Term(iTRAQ4plex); C7(Methylthio); K16(iTRAQ4plex)	1983.96933	1.623	2.063	1.855	1.731	3.483	2.442	2.867	2.707		8.8		39.7		23.2		16.9	0.0E+00	3.9E-09	4.0	37	1.9E-02	5.4	71	8.3E-06	0
31377806																															31377806	LSDAGQYLCQAGDDSNSNK	6	N-Term(iTRAQ4plex); C9(Methylthio); K19(iTRAQ4plex)	2320.04422	1.768	1.515	1.618	1.604	2.460	3.166	2.975	2.537	12.4		43.5		14.1		15.3		0.0E+00	7.7E-08	5.7	57	7.8E-05	6.1	53	1.9E-04	0
31377806																															31377806	VYTVDLGR	24	N-Term(iTRAQ4plex)	1066.60234	1.744	1.630	2.141	1.688	2.473	2.734	3.314	2.508	16.1	29.6	67.6	48.1	41.8	27.3	34.0	35.0	0.0E+00	4.5E-02	3.5	33	5.4E-02	3.5	33	6.0E-02	0
31377806																															31377806	LFAEEK	3	N-Term(iTRAQ4plex); K6(iTRAQ4plex)	1024.58916	1.380	1.528	1.603	1.494	2.607	1.952	2.327	2.483		10.5		1.7		6.0		2.7	5.0E-03	4.0E-02		37	1.7E-02		37	1.1E-02	0
31377806																															31377806	YWCLWEGAQNGR	5	N-Term(iTRAQ4plex); C3(Methylthio); N10(Deamidated)	1673.73747	1.029	1.543	1.297	1.572	2.258	2.500	3.237	2.239		64.7		96.5		39.2		136.3	0.0E+00	8.0E-04	1.5			2.8	45	6.6E-04	0
31377806																															31377806	SPTVVK	3	N-Term(iTRAQ4plex); K6(iTRAQ4plex)	918.58611		1.463		1.354	2.524			2.007		14.9		3.7		4.8		6.7	4.0E-03	2.4E-02					42	8.2E-03	0

31377806																															31377806	LSDAGQYLCQAGDDSNSK	2	N-Term(iTRAQ4plex); C9(Methylthio); N16(Deamidated); K19(iTRAQ4plex)	2321.02201		2.224		1.700	2.522			1.877									0.0E+00	1.5E-06				3.4	46	8.3E-04	0
31377806																															31377806	DGSFSVVITGLR	3	N-Term(iTRAQ4plex)	1394.77397	1.641	1.206	1.979	1.043	1.738	2.071	2.565	1.463									0.0E+00	4.1E-04	2.4			3.3	32	7.0E-02	0
31377806																															31377806	TVTINCPFK	4	N-Term(iTRAQ4plex); C6(Methylthio); K9(iTRAQ4plex)	1356.72576	2.219		2.701			3.798	4.747		2.2		7.2		10.6		5.6		0.0E+00	1.8E-04	3.6	55	4.4E-04				0
31377806																															31377806	ADEGWYWCGVK	4	N-Term(iTRAQ4plex); C8(Methylthio); K11(iTRAQ4plex)	1647.75102	1.915		2.001			3.235	3.470		98.6		59.3		97.2		60.4		0.0E+00	2.2E-04	2.6	46	7.3E-04				0
31377806																															31377806	QSSGENCDVVVNTLGK	1	N-Term(iTRAQ4plex); N6(Deamidated); C7(Methylthio); K16(iTRAQ4plex)	1984.95554	1.402		1.258			1.700	1.567										1.0E-03	2.3E-02	2.2						0
31377806																															31377806	GVAGGSVAVLCPYNR	4	N-Term(iTRAQ4plex); C11(Methylthio)	1652.83756	1.142		0.934			1.586	1.332		33.1		45.2		36.7		41.4		0.0E+00	2.1E-06	4.9	50	1.2E-03				0
31377806																															31377806	GGCITLISSEGYVSSK	2	N-Term(iTRAQ4plex); C3(Methylthio); K16(iTRAQ4plex)	1934.98093	1.123		0.995			1.171	1.066										0.0E+00	2.3E-04	2.7	27	2.3E-01				0
32130518	NP_000474.2	APOC2	344	apolipoprotein C-II precursor	GBC IIIA/GSD	IIIA specific	3	81	1.295	1.134	1.7E-11	**2.052**	**2.375**	2.7E-41	0.743	0.700	9.0E-28	1.236	1.349	8.3E-02	19.2	15.6	15.6	15.0	9.5	16.9	19.9	12.4	11.3	4.72	32130518																													
32130518																															32130518	TYLPAVDEK	30	N-Term(iTRAQ4plex); K9(iTRAQ4plex)	1323.73943	1.271	1.159	2.157	2.371	0.717	0.712	1.376	1.388	5.6	9.1	9.1	8.9	1.4	22.2	3.3	15.3	0.0E+00	1.5E-06	3.4	59	1.6E-04	3.7	61	8.4E-05	0
32130518																															32130518	TAAQNLYEK	4	N-Term(iTRAQ4plex); N5(Deamidated); K9(iTRAQ4plex)	1326.71453		1.165		2.160	0.743			1.343		28.4		4.2		15.8		7.9	0.0E+00	2.2E-03				3.5	50	1.3E-03	0
32130518																															32130518	TAAQNLYEK	28	N-Term(iTRAQ4plex); K9(iTRAQ4plex)	1325.73137	1.441	1.105	3.415	2.390	0.631	0.791	1.926	1.315		18.2		11.5		9.2		21.3	0.0E+00	1.5E-06	4.4	54	4.6E-04	4.5	58	2.1E-04	0
32130518																															32130518	ESLSSYWESAK	19	N-Term(iTRAQ4plex); K11(iTRAQ4plex)	1574.79534	1.250	1.160	1.759	1.632	0.764	0.740	1.120	1.124	18.8	5.6	14.7	9.7	38.1	31.2	12.0	16.0	0.0E+00	2.6E-05	5.2	57	1.8E-04	3.4	32	5.2E-02	0
40217843	NP_000086.2	COMP	1311	cartilage oligomeric matrix protein precursor	GBC IIIA/GSD; GBC IVB vs GSD	IIIA and IVB	6	19	0.783	0.852	5.0E-02	**0.447**	**0.412**	6.0E-08	0.767	0.703	4.4E-07	**0.414**	**0.331**	8.0E-09	44.5	21.2	23.9	5.2	16.4	22.1	37.5	50.9	82.8	4.60	40217843																													
40217843																															40217843	SSTGPGEQLR	3	N-Term(iTRAQ4plex)	1175.61528	0.969	0.823	0.538	0.474	0.814	0.814	0.464	0.457									4.0E-03	6.9E-02	3.0	48	1.6E-03	3.3			0
40217843																															40217843	ELQETNAALQDVR	6	N-Term(iTRAQ4plex)	1630.85015	0.849	0.882	0.346	0.357	0.607	0.639	0.267	0.239	30.0		57.9		33.1		54.4		0.0E+00	1.5E-06	5.4	74	4.5E-06	5.1	57	2.4E-04	0
40217843																															40217843	NQADNCPR	2	N-Term(iTRAQ4plex); C6(Methylthio)	1107.48027	0.780		0.620			0.755	0.617										0.0E+00	5.3E-03	2.6	18	1.5E-01				0
40217843																															40217843	LVPNPGQEDADR	2	N-Term(iTRAQ4plex)	1454.73320	0.669		0.465			0.812	0.580										0.0E+00	1.5E-02	3.2	44	3.2E-03				0
40217843																															40217843	NALWHTGDTESQVR	2	N-Term(iTRAQ4plex)	1757.85935	0.918		0.683			0.583	0.445										1.0E-03	2.4E-02	2.7	11	6.0E+00				0
40217843																															40217843	DTDLDGFPDEK	4	N-Term(iTRAQ4plex); K11(iTRAQ4plex)	1539.74016	0.699		0.317			0.686	0.320		17.8		15.8		14.5		19.2		0.0E+00	6.1E-05	3.8	59	7.1E-05				0
45580688	NP_000578.2	C7	730	complement component C7 precursor	GBC IVB/Healthy	IVB specific	16	79	1.866	**2.010**	1.8E-33	0.983	1.077	2.4E-02	**2.109**	**2.458**	5.0E-19	1.158	1.225	2.0E-07	10.2	17.8	16.8	31.2	25.4	31.9	32.9	18.6	93.5	6.48	45580688																													
45580688																															45580688	EQTMSECEAGALR	4	N-Term(iTRAQ4plex); M4(Oxidation); C7(Methylthio)	1630.69829	1.669	2.519	0.911	1.459	2.830	1.836	1.030	1.597									0.0E+00	1.4E-05	2.8	64	7.4E-06	2.6	51	1.5E-04	0
45580688																															45580688	QNDFNSVEEK	2	N-Term(iTRAQ4plex); N2(Deamidated); K10(iTRAQ4plex)	1498.72539		1.378		1.028	1.965			1.428									2.0E-03	9.5E-02				1.8	20	5.9E-01	0
45580688																															45580688	EVPCASVK	8	N-Term(iTRAQ4plex); C4(Methylthio); K8(iTRAQ4plex)	1166.61431	1.870	2.207	1.097	1.173	2.632	1.821	1.097	1.362	16.5	19.9	18.8	0.6	7.2	0.2	28.3	19.5	0.0E+00	3.1E-02	2.8	46	2.1E-03	2.9	43	3.5E-03	0
45580688																															45580688	VLFYVDSEK	4	N-Term(iTRAQ4plex); K9(iTRAQ4plex)	1387.77178		2.729		1.221	3.041			1.325		4.2		13.9		19.1		9.2	0.0E+00	1.5E-06				3.3	59	1.3E-04	0
45580688																															45580688	CDAESSK	2	N-Term(iTRAQ4plex); C1(Methylthio); K7(iTRAQ4plex)	1073.48516		2.642		1.214	2.813			1.259									1.0E-03	1.4E-02				2.4	19	1.7E-01	0
45580688																															45580688	SFGGQCR	3	N-Term(iTRAQ4plex); C6(Methylthio)	944.41911	1.834	2.010	1.201	1.113	2.313	2.046	1.375	1.247									5.0E-03	1.6E-01	2.7			2.7	17	3.0E-01	0
45580688																															45580688	ECNNPPPSGGGR	18	N-Term(iTRAQ4plex); C2(Methylthio)	1374.60112	1.708	2.005	1.147	1.077	2.458	2.484	1.512	1.242	28.5	5.3	7.3	35.6	26.6	15.4	15.0	31.9	0.0E+00	4.0E-04	3.1	41	1.1E-03	3.1	35	4.2E-03	0
45580688																															45580688	QNDFNSVEEK	2	N-Term(iTRAQ4plex); K10(iTRAQ4plex)	1497.74211		2.199		1.075	2.573			1.225									0.0E+00	1.5E-06				4.1	50	8.2E-04	0
45580688																															45580688	EQTMSECEAGALR	2	N-Term(iTRAQ4plex); C7(Methylthio)	1614.71062		1.751		0.997	2.121			1.176									0.0E+00	2.7E-02				3.3	27	5.0E-02	0
45580688																															45580688	AASGTQNNVLR	4	N-Term(iTRAQ4plex)	1274.69390		1.775		0.945	2.159			1.119		76.8		20.2		38.8		54.1	0.0E+00	2.4E-04				5.1	64	3.8E-05	0
45580688																															45580688	SLVCNGDSDCDEDSADEDR	4	N-Term(iTRAQ4plex); C4(Methylthio); N5(Deamidated); C10(Methylthio)	2281.79473		2.270		1.122	2.275			1.095		51.5		36.1		25.4		64.0	0.0E+00	1.5E-06				4.6	57	2.2E-06	0
45580688																															45580688	DSCTLPASAEK	10	N-Term(iTRAQ4plex); C3(Methylthio); K11(iTRAQ4plex)	1455.70244	2.047	2.054	0.956	1.047	2.507	2.389	1.145	1.079	14.3	18.0	2.1	6.9	1.8	14.2	14.0	3.6	0.0E+00	1.1E-04	4.0	55	1.3E-04	2.8	35	1.4E-02	0
45580688																															45580688	GCPTEEGCGER	4	N-Term(iTRAQ4plex); C2(Methylthio); C8(Methylthio)	1373.50823		1.404		1.018	1.358			0.959		30.3		37.3		26.5		41.2	0.0E+00	4.8E-02				2.1	24	1.1E-02	0
45580688																															45580688	DGFVQDEGTMFPVGK	4	N-Term(iTRAQ4plex); M10(Oxidation); K15(iTRAQ4plex)	1930.95444	2.948	0.990	0.950	0.843	1.108	2.278	0.753	0.919									0.0E+00	3.3E-05	2.3	20	7.2E-01	2.1	25	2.8E-01	0
45580688																															45580688	ELENALK	2	N-Term(iTRAQ4plex); K7(iTRAQ4plex)	1104.65019	1.917		1.115			1.935	1.155										1.0E-03	1.9E-02	3.3	50	9.2E-04				0
45580688																															45580688	LIDQYGTHYLQSGSLGGEYR	2	N-Term(iTRAQ4plex); Q11(Deamidated)	2402.18589	1.873		0.998			1.543	0.844										0.0E+00	2.1E-06	5.3	52	8.4E-04				0
45580688																															45580688	SCVGETTESTQCEDEELEHLR	2	N-Term(iTRAQ4plex); C2(Methylthio); C12(Methylthio)	2631.07614	1.885		0.808			1.773	0.780										0.0E+00	1.4E-07	5.3	41	7.3E-04				0

45580688																															45580688	ELSHLPSLYDYSAYR	2	N-Term(iTRAQ4plex)	1957.97788	1.411		0.928			1.147	0.775										0.0E+00	3.2E-05	2.8	20	1.1E+00				0
47078292	NP_000203.2	ITGB3	3690	integrin beta-3 precursor	GBC IIIA/Healthy	IIIA specific	11	80	**0.451**	**0.485**	1.2E-24	0.932	0.982	2.0E-01	**0.510**	0.558	5.9E-27	1.021	1.125	3.9E-09	22.3	26.3	24.8	29.4	19.0	37.0	25.9	22.6	87.0	5.24	47078292																													
47078292																															47078292	HVLTLTDQVTR	2	N-Term(iTRAQ4plex)	1426.81427		0.728		1.169	0.828			1.295									0.0E+00	1.5E-05				2.3	19	1.4E+00	0
47078292																															47078292	WDTANNPLYK	10	N-Term(iTRAQ4plex); K10(iTRAQ4plex)	1509.80351	0.404	0.529	0.988	1.265	0.556	0.492	1.224	1.294	9.7	22.4	1.1	15.7	6.5	7.0	5.1	0.5	0.0E+00	1.1E-04	4.3	45	4.6E-03	3.9	40	1.3E-02	0
47078292																															47078292	FQYYEDSSGK	8	N-Term(iTRAQ4plex); K10(iTRAQ4plex)	1511.72869	0.340	0.399	0.959	0.982	0.434	0.405	1.172	1.245		4.6		8.4		19.9		3.2	0.0E+00	3.1E-05	4.0	74	2.3E-06	3.5	70	6.4E-06	0
47078292																															47078292	NEDDCVVR	4	N-Term(iTRAQ4plex); C5(Methylthio)	1139.50041	0.447	0.413	1.167	1.027	0.510	0.518	1.391	1.234									0.0E+00	1.4E-02	3.6	24	3.2E-02	3.5	26	1.3E-02	0
47078292																															47078292	DEIESVK	4	N-Term(iTRAQ4plex); K7(iTRAQ4plex)	1107.61565	0.573	0.490	0.997	1.140	0.517	0.511	0.914	1.172		10.9		6.1		7.3		12.2	0.0E+00	4.1E-03		33	5.9E-02	3.2	38	2.2E-02	0
47078292																															47078292	GSGDSSQVTQVSPQR	20	N-Term(iTRAQ4plex)	1676.83245	0.402	0.419	0.919	0.935	0.481	0.428	0.857	1.090	21.2	86.0	15.9	28.0	35.3	24.3	9.5	49.1	0.0E+00	1.6E-06	5.1	87	2.1E-07	4.8	54	4.4E-04	0
47078292																															47078292	EATSTFTNITYR	10	N-Term(iTRAQ4plex)	1547.78557	0.525	0.509	0.830	0.899	0.602	0.569	0.941	1.036	2.8	5.9	1.7	10.1	8.7	0.8	4.0	5.0	0.0E+00	5.8E-04	4.8	61	7.1E-05	5.2	58	1.6E-04	0
47078292																															47078292	EGQPVCSQR	7	N-Term(iTRAQ4plex); C6(Methylthio)	1193.55376	0.514	0.593	0.761	0.851	0.729	0.578	0.879	1.019	3.4	40.6	32.6	24.0	26.1	22.5	2.7	42.2	0.0E+00	3.4E-03	2.6	32	1.2E-02	2.4	20	2.2E-01	0
47078292																															47078292	GSGDSSQVTQVSPQR	4	N-Term(iTRAQ4plex); Q10(Deamidated)	1677.82805	1.144	0.343	1.251	0.799	0.421	1.121	1.259	0.954									0.0E+00	1.6E-03	3.7	11	7.3E+00	4.7	69	1.1E-05	0
47078292																															47078292	DNCAPESIEFPVSEAR	6	N-Term(iTRAQ4plex); C3(Methylthio)	1953.88474	0.459	0.576	1.003	0.961	0.577	0.428	0.960	0.938	57.9		16.1		32.8		39.2		0.0E+00	3.1E-06	5.2	83	2.1E-07	3.3	30	3.8E-02	0
47078292																															47078292	GALHDENTCNR	2	N-Term(iTRAQ4plex); C9(Methylthio)	1419.62317		0.549		0.653	0.710			0.823									0.0E+00	1.6E-06				3.5	19	1.5E-01	0
47078292																															47078292	DAPEGGFDAIMQATVCDEK	2	N-Term(iTRAQ4plex); C16(Methylthio); K19(iTRAQ4plex)	2331.05381		0.676		1.459	0.385			0.810									0.0E+00	1.7E-04				2.8	21	3.9E-01	0
47078292																															47078292	DAPEGGFDAIMQATVCDEK	1	N-Term(iTRAQ4plex); M11(Oxidation); Q12(Deamidated); C16(Methylthio); K19(iTRAQ4plex)	2348.03922	0.425		0.779			0.355	0.668										0.0E+00	1.2E-02	2.3						0
48255966	NP_006750.3	UGP2	7360	UTP--glucose-1-phosphate uridylyltransferase isoform a	GBC IIIA/Healthy; GBC IVB vs Healthy	IIIA and IVB	10	36	**3.137**	**3.091**	1.5E-25	1.125	1.216	1.3E-05	**2.266**	**2.309**	1.8E-11	0.844	0.840	1.1E-14	17.4	12.6	38.9	46.1	47.5	33.1	27.0	15.1	56.9	8.15	48255966																													
48255966																															48255966	EFPTVPLVK	12	N-Term(iTRAQ4plex); K9(iTRAQ4plex)	1317.79900	3.801	2.966	1.013	1.322	2.370	2.349	0.750	0.982	39.4	15.5	2.9	2.7	1.6	8.5	10.7	8.0	0.0E+00	1.2E-02	3.5	64	3.6E-05	3.0	42	5.5E-03	0
48255966																															48255966	LVEIAQVPK	2	N-Term(iTRAQ4plex); K9(iTRAQ4plex)	1284.81113		4.208		1.434	2.921			0.970									0.0E+00	5.8E-04				2.8	34	8.6E-03	0
48255966																															48255966	AHVDEFK	1	N-Term(iTRAQ4plex); K7(iTRAQ4plex)	1133.61893		2.254		1.046	1.858			0.840									7.0E-03	1.9E-01				1.6			0
48255966																															48255966	SFENSLGINVPR	6	N-Term(iTRAQ4plex)	1476.79277	2.291	3.952	1.390	1.179	2.791	1.659	1.034	0.811		37.7		13.5		5.2		28.7	0.0E+00	1.9E-07	3.9	23	5.5E-01	4.6	74	4.3E-06	0
48255966																															48255966	IVSGNLR	2	N-Term(iTRAQ4plex)	902.55254	1.673	2.299	1.297	1.216	1.571	1.170	0.932	0.809									5.0E-03	1.7E-01	2.5			2.7			0
48255966																															48255966	GPSVDWGK	6	N-Term(iTRAQ4plex); K8(iTRAQ4plex)	1133.61833	3.117	3.074	0.943	1.118	2.191	2.800	0.870	0.776	10.5		22.6		8.8		20.9		0.0E+00	2.6E-02	2.8	65	2.5E-05	3.2	44	3.7E-03	0
48255966																															48255966	FVQDLSK	3	N-Term(iTRAQ4plex); K7(iTRAQ4plex)	1124.65422	6.937	4.946	1.744	1.078	3.654	4.604	1.189	0.776									5.0E-03	7.4E-02	2.9	41	4.9E-03	2.6			0
48255966																															48255966	IYTFNQSR	1	N-Term(iTRAQ4plex)	1172.61785		3.483		1.402	1.936			0.759									6.0E-03	5.4E-02					19	9.9E-01	0
48255966																															48255966	IQRPPEDSIQPYEK	1	N-Term(iTRAQ4plex); K14(iTRAQ4plex)	1988.06687	1.942		1.132			1.683	1.007										0.0E+00	1.5E-02	4.3						0
48255966																															48255966	GGTLTQYEGK	2	N-Term(iTRAQ4plex); K10(iTRAQ4plex)	1341.72356	3.704		1.154			2.149	0.688										0.0E+00	2.6E-04	3.3	44	3.5E-03				0
55770842	NP_000558.2	CRP	1401	C-reactive protein precursor	GBC IIIA/Healthy, GSD; GBC IVB/Healthy, GSD	IIIA and IVB	4	59	**3.684**	**2.672**	1.1E-31	**4.241**	**2.629**	5.0E-26	**4.699**	**3.340**	1.0E-25	**4.874**	**3.325**	1.3E-21	53.3	67.8	39.4	46.7	41.0	62.0	37.0	85.6	25.0	5.63	55770842																													
55770842																															55770842	AFVFPK	3	N-Term(iTRAQ4plex); K6(iTRAQ4plex)	996.61254	4.640	3.663	5.551	4.176	5.156	5.880	7.222	5.724	23.6		33.5		27.3		29.8		4.0E-03	2.4E-02		41	7.4E-03		45	3.0E-03	0
55770842																															55770842	ESDTSYVSLK	50	N-Term(iTRAQ4plex); K10(iTRAQ4plex)	1416.74626	3.548	2.634	4.187	2.249	3.263	4.362	5.065	2.934	40.0	42.9	38.5	91.8	45.6	75.7	46.0	53.8	0.0E+00	1.5E-06	4.8	73	5.7E-06	4.6	73	5.3E-06	0
55770842																															55770842	GYSIFSYATK	2	N-Term(iTRAQ4plex); K10(iTRAQ4plex)	1424.76579	3.365		2.934			4.571	4.092										0.0E+00	1.3E-04	4.4	45	4.3E-03				0
55770842																															55770842	YEVQGEVFTKPQLWP	4	N-Term(iTRAQ4plex); K10(iTRAQ4plex)	2109.12613	4.672		3.451			4.826	3.661		54.4		38.0		33.4		49.3		0.0E+00	5.3E-04	3.3	23	6.8E-01				0
56682959	NP_002023.2	FTH1	2495	ferritin heavy chain	GBC IVB/GSD	IVB specific	3	8	1.637	1.540	1.2E-02	0.695	**0.473**	4.5E-03	1.143	1.485	8.4E-01	**0.498**	**0.444**	1.9E-03	51.9	4.3	7.1	21.0	7.5	3.9	52.3	12.7	21.2	5.55	56682959																													
56682959																															56682959	QNYHQDSEAAINR	4	N-Term(iTRAQ4plex)	1689.80527	1.717	1.338	0.966	0.459	1.447	1.202	0.694	0.484									0.0E+00	1.5E-06	3.3	26	1.1E-01	3.4	34	2.0E-02	0
56682959																															56682959	MGAPESGLAEYLFDK	2	N-Term(iTRAQ4plex); M1(Oxidation); K15(iTRAQ4plex)	1931.96427		1.772		0.487	1.524			0.408									0.0E+00	1.5E-06				3.8	39	1.6E-02	0
56682959																															56682959	ELGDHVTNLR	2	N-Term(iTRAQ4plex)	1297.69672	1.561		0.500			1.086	0.358										0.0E+00	2.9E-03	3.8	23	5.8E-01				0
62362414	NP_005148.2	ABL1	25	tyrosine-protein kinase ABL1 isoform a	GBC IVB/Healthy	IVB specific	1	5	0.660	0.614	4.5E-03	0.726	0.657	4.1E-03	**0.484**	**0.542**	5.7E-04	0.547	0.590	4.8E-04		0.6		11.8		4.9		13.0	122.8	8.66	62362414																													
62362414																															62362414	LENNLR	5	N-Term(iTRAQ4plex); N3(Deamidated)	903.50121	0.660	0.614	0.726	0.657	0.542	0.484	0.547	0.590		11.8		13.0		0.6		4.9	4.0E-03	1.4E-01		31	6.8E-02	2.7	31	7.0E-02	0

70906437	NP_000500.2	FGG	2266	fibrinogen gamma chain isoform gamma-A precursor	GBC IVB/Healthy	IVB specific	27	1866	1.661	1.633	0.0E+00	1.232	1.168	1.1E-138	**2.045**	**2.099**	0.0E+00	1.534	1.441	3.2E-24	27.7	13.6	23.4	23.0	29.5	27.7	30.7	18.7	49.5	6.09	70906437																													
70906437																															70906437	VAQLEAQCQEPCKDTVQIHDITGK	2	N-Term(iTRAQ4plex); C8(Methylthio); C12(Methylthio); K13(iTRAQ4plex); K24(iTRAQ4plex)	3178.56850		1.416		0.898	3.304			2.040									0.0E+00	1.5E-06				7.6	31	1.1E-01	1
70906437																															70906437	DNCCILDER	44	N-Term(iTRAQ4plex); C3(Methylthio); C4(Methylthio)	1316.52300	1.656	1.909	1.337	1.489	2.501	2.490	1.980	2.019	13.9	4.8	25.0	9.6	5.8	15.7	19.8	12.9	0.0E+00	1.5E-06	3.7	43	2.2E-04	3.6	46	1.0E-04	0
70906437																															70906437	DNCCILDER	14	N-Term(iTRAQ4plex); N2(Deamidated); C3(Methylthio); C4(Methylthio)	1317.50652	1.947	1.773	1.282	1.512	2.399	2.767	2.045	1.993	11.8		36.2		35.3		12.6		0.0E+00	1.0E-02	3.3	44	1.5E-04	3.1	30	4.6E-03	0
70906437																															70906437	VAQLEAQCQEPCK	36	N-Term(iTRAQ4plex); C8(Methylthio); C12(Methylthio); K13(iTRAQ4plex)	1826.85222	2.110	2.252	2.385	1.505	2.910	3.287	3.699	1.822	23.1	14.8	8.5	7.6	3.4	5.0	28.6	7.4	0.0E+00	2.0E-08	5.6	66	1.3E-05	5.2	66	1.3E-05	0
70906437																															70906437	AIQLTYNPDESSKPNMIDAATLK	6	N-Term(iTRAQ4plex); K13(iTRAQ4plex); N15(Deamidated); M16(Oxidation); K23(iTRAQ4plex)	2969.56679	1.345	1.339	0.939	1.215	2.006	1.271	0.911	1.772		18.8		21.8		5.4		2.6	0.0E+00	1.5E-06	2.3	24	6.2E-01	3.7	40	1.1E-02	0
70906437																															70906437	VGPEADKYR	14	N-Term(iTRAQ4plex); K7(iTRAQ4plex)	1322.73099	1.798	1.482	1.601	1.032	2.338	1.290	1.194	1.665	9.6	21.4	7.8	2.3	11.1	9.2	0.5	31.4	0.0E+00	2.6E-03	4.3	25	4.2E-01	4.4	29	1.4E-01	1
70906437																															70906437	RLDGSVDFK	28	N-Term(iTRAQ4plex); K9(iTRAQ4plex)	1324.74497	1.439	1.921	0.962	1.220	2.658	1.791	1.192	1.660	27.5	7.1	18.8	3.3	12.5	3.4	31.9	14.7	0.0E+00	2.9E-03	3.9	27	2.7E-01	4.0	24	5.8E-01	1
70906437																															70906437	KNWIQYK	3	N-Term(iTRAQ4plex); K1(iTRAQ4plex); K7(iTRAQ4plex)	1411.84256		1.925		1.246	2.598			1.639		25.1		14.8		17.8		22.1	5.0E-03	4.2E-02				3.6	22	4.7E-01	1
70906437																															70906437	LDGSVDFK	83	N-Term(iTRAQ4plex); K8(iTRAQ4plex)	1168.64507	1.783	1.862	1.977	1.308	2.471	2.525	2.951	1.635	22.1	15.4	22.1	18.0	19.8	11.8	17.9	19.6	0.0E+00	6.0E-05	3.6	50	1.0E-03	3.6	50	1.0E-03	0
70906437																															70906437	MLEEIMK	16	N-Term(iTRAQ4plex); K7(iTRAQ4plex)	1181.65227	2.532	1.886	1.749	1.257	2.570	3.079	2.179	1.614	6.0	6.2	1.3	5.9	0.4	0.2	8.1	20.9	1.0E-03	6.6E-02	3.2	39	1.3E-02	3.3	37	1.8E-02	0
70906437																															70906437	MLEEIMK	14	N-Term(iTRAQ4plex); M6(Oxidation); K7(iTRAQ4plex)	1197.64568	2.019	1.863	1.592	1.220	2.465	2.509	2.178	1.601	11.0	10.1	3.8	15.9	3.1	3.2	5.8	17.9	2.0E-03	2.2E-02	2.0	33	4.7E-02	2.6	34	3.9E-02	0
70906437																															70906437	NWIQYK	3	N-Term(iTRAQ4plex); K6(iTRAQ4plex)	1139.64466		1.796		1.159	2.395			1.564		5.7		12.6		11.3		22.8	7.0E-03	6.5E-02					31	9.9E-02	0
70906437																															70906437	DCQDIANK	158	N-Term(iTRAQ4plex); C2(Methylthio); K8(iTRAQ4plex)	1240.58977	1.668	1.716	1.096	1.177	2.238	2.159	1.413	1.558	31.2	29.7	20.4	14.9	14.9	11.6	35.8	33.1	0.0E+00	2.7E-04	4.0	45	8.2E-04	4.0	45	8.1E-04	0
70906437																															70906437	CHAGHLNGVYYQGGTYSK	43	N-Term(iTRAQ4plex); C1(Methylthio); N7(Deamidated); K18(iTRAQ4plex)	2290.06400	1.341	1.239	1.388	0.799	2.455	1.662	1.766	1.541	15.9		28.6		23.6		20.5		0.0E+00	2.1E-07	7.4	58	1.0E-04	3.9	28	1.0E-01	0
70906437																															70906437	YLQEIYNSNNQK	68	N-Term(iTRAQ4plex); K12(iTRAQ4plex)	1801.93425	1.864	1.765	1.981	1.194	2.255	2.277	2.683	1.516	32.6	16.2	39.9	13.3	28.3	15.3	45.5	16.6	0.0E+00	1.5E-06	5.3	64	4.7E-05	5.3	68	1.7E-05	0
70906437																															70906437	DCQDIANK	31	N-Term(iTRAQ4plex); C2(Methylthio); Q3(Deamidated); K8(iTRAQ4plex)	1241.57317	1.591	1.802	0.989	1.213	2.420	2.015	1.245	1.511	21.9	4.5	21.9	12.0	17.2	5.3	27.2	7.3	0.0E+00	1.9E-02	3.6	49	2.0E-04	3.8	55	4.3E-05	0
70906437																															70906437	EGFGHLSPTGTTEFWLGNEK	15	N-Term(iTRAQ4plex); N18(Deamidated); K20(iTRAQ4plex)	2496.22849	1.223	1.736	0.643	1.246	1.956	1.913	1.020	1.511	4.6	5.2	9.9	19.1	0.3	8.6	11.6	35.1	0.0E+00	2.8E-06	6.4	36	3.4E-02	6.8	42	7.4E-03	0
70906437																															70906437	VGPEADK	31	N-Term(iTRAQ4plex); K7(iTRAQ4plex)	1003.56627	1.661	1.766	1.442	1.287	2.100	2.172	1.866	1.497	19.7	14.8	8.3	10.9	1.6	8.7	15.1	18.8	0.0E+00	1.2E-02	2.8	55	2.0E-04	2.8	55	1.8E-04	0
70906437																															70906437	AIQLTYNPDESSKPNMIDAATLK	22	N-Term(iTRAQ4plex); K13(iTRAQ4plex); K23(iTRAQ4plex)	2952.56870	1.948	1.624	0.997	1.055	2.439	1.710	0.836	1.493	26.5	25.0	19.6	29.8	4.3	12.5	14.6	38.4	0.0E+00	1.0E-09	8.4	71	9.6E-06	5.2	37	2.0E-02	0
70906437																															70906437	LTYAYFAGGDAGDAFDGFDFGDDPSDK	13	N-Term(iTRAQ4plex); K27(iTRAQ4plex)	3122.38034		1.823		1.282	2.306			1.480		27.3		22.4		8.5		20.3	0.0E+00	7.3E-18	1.0			5.4	89	4.3E-08	0
70906437																															70906437	AIQLTYNPDESSKPNMIDAATLK	32	N-Term(iTRAQ4plex); K13(iTRAQ4plex); M16(Oxidation); K23(iTRAQ4plex)	2968.56681	1.775	1.508	1.028	1.030	2.213	1.537	0.924	1.475	19.0	16.7	22.2	5.8	10.4	16.5	19.2	5.9	0.0E+00	5.1E-05	5.5	44	4.7E-03	4.1	40	1.1E-02	0
70906437																															70906437	FEGNCAEQDGSGWWMNK	8	N-Term(iTRAQ4plex); C5(Methylthio); M15(Oxidation); K17(iTRAQ4plex)	2308.96653	1.709	1.886	1.863	1.188	2.383	2.678	2.998	1.463	52.4		10.4		2.4		30.3		0.0E+00	9.0E-07	5.4	54	4.4E-05	5.3	53	5.5E-05	0
70906437																															70906437	MLEEIMK	19	N-Term(iTRAQ4plex); M1(Oxidation); M6(Oxidation); K7(iTRAQ4plex)	1213.64141	1.934	1.595	1.652	1.170	2.071	2.231	1.950	1.462	25.0	9.1	9.4	12.4	9.3	14.5	21.1	10.6	4.0E-03	1.4E-01		44	3.2E-03	1.8	44	3.6E-03	0
70906437																															70906437	TSTADYAMFK	65	N-Term(iTRAQ4plex); K10(iTRAQ4plex)	1422.71770	2.214	1.645	1.716	1.175	1.947	2.581	2.059	1.433	8.8	25.8	1.1	15.5	4.0	12.1	9.6	34.3	0.0E+00	1.5E-06	4.0	67	1.8E-05	4.3	67	1.7E-05	0
70906437																															70906437	ASTPNGYDNGIIWATWK	20	N-Term(iTRAQ4plex); N5(Deamidated); N9(Deamidated); K17(iTRAQ4plex)	2184.09307	2.068	1.737	0.844	1.251	2.020	2.108	0.914	1.427	16.1	22.8	12.1	18.3	8.6	20.4	25.8	45.3	0.0E+00	1.7E-06	4.5	45	3.6E-03	4.7	53	7.0E-04	0
70906437																															70906437	DTVQIHDITGK	172	N-Term(iTRAQ4plex); K11(iTRAQ4plex)	1514.84087	1.538	1.610	1.024	1.192	2.017	2.101	1.416	1.416	34.7	21.0	24.1	21.4	9.9	15.0	38.2	24.2	0.0E+00	1.5E-06	5.3	82	7.5E-07	4.9	82	7.4E-07	0
70906437																															70906437	YEASILTHDSSIR	1	N-Term(iTRAQ4plex); Y1(iTRAQ4plex)	1779.94790		1.903		1.351	2.030			1.404									0.0E+00	4.2E-05					15	4.6E+00	0
70906437																															70906437	FFTSHNGMQFSTWDNDNDK	8	N-Term(iTRAQ4plex); N6(Deamidated); M8(Oxidation); K19(iTRAQ4plex)	2596.12973	1.500	1.777	1.670	1.339	1.908	2.584	2.954	1.400	38.7	35.3	5.1	24.1	10.1	28.2	33.3	31.1	0.0E+00	4.2E-08	6.0	49	3.7E-04	6.0	54	1.4E-04	0
70906437																															70906437	VELEDWNGR	27	N-Term(iTRAQ4plex)	1261.62798	1.600	1.538	1.209	1.235	1.832	1.781	1.446	1.395	9.3	14.8	36.6	12.3	20.2	14.1	26.3	8.6	0.0E+00	4.7E-06	3.1	34	2.6E-02	3.5	35	2.1E-02	0
70906437																															70906437	VELEDWNGR	121	N-Term(iTRAQ4plex); N7(Deamidated)	1262.61284	1.643	1.627	1.042	1.136	2.102	2.050	1.336	1.390	16.0	23.0	6.2	9.6	9.5	8.2	13.5	22.2	0.0E+00	2.2E-06	3.6	48	7.5E-04	3.8	50	4.0E-04	0
70906437																															70906437	DTVQIHDITGK	2	N-Term(iTRAQ4plex); Q4(Deamidated); K11(iTRAQ4plex)	1515.82175		1.638		1.145	2.040			1.389									0.0E+00	1.6E-06				4.3	40	1.2E-02	0
70906437																															70906437	YEASILTHDSSIR	97	N-Term(iTRAQ4plex)	1635.84526	1.455	1.523	1.145	1.060	1.986	1.880	1.784	1.386	22.2	18.0	21.7	16.0	29.1	11.8	46.1	35.2	0.0E+00	1.5E-06	5.1	65	3.8E-05	5.8	70	1.4E-05	0
70906437																															70906437	EGFGHLSPTGTTEFWLGNEK	29	N-Term(iTRAQ4plex); K20(iTRAQ4plex)	2495.23954	1.409	1.377	0.628	1.117	2.029	2.186	1.112	1.379	49.5	26.2	16.9	29.4	12.5	20.7	43.8	39.3	0.0E+00	1.8E-09	7.8	60	1.3E-04	6.5	50	1.3E-03	0
70906437																															70906437	YLQEIYNSNNQK	143	N-Term(iTRAQ4plex); N7(Deamidated); K12(iTRAQ4plex)	1802.91716	1.732	1.576	1.648	1.113	2.084	1.994	1.935	1.342	17.9	24.8	31.2	16.3	16.3	13.0	27.7	23.2	0.0E+00	1.5E-06	4.9	73	5.9E-06	5.2	75	3.4E-06	0
70906437																															70906437	LTIGEGQQHHLGGAK	222	N-Term(iTRAQ4plex); K15(iTRAQ4plex)	1834.01662	1.612	1.521	1.208	1.167	1.785	1.900	1.450	1.328	16.8	23.1	12.7	24.7	8.9	19.0	23.4	35.5	0.0E+00	4.5E-06	6.3	57	2.8E-04	6.7	74	5.6E-06	0
70906437																															70906437	ASTPNGYDNGIIWATWK	8	N-Term(iTRAQ4plex); N5(Deamidated); K17(iTRAQ4plex)	2183.10056	1.344	2.557	0.845	1.137	3.055	1.582	0.951	1.323	33.3		21.0		47.5		15.5		0.0E+00	3.8E-03	3.9	31	1.0E-01	3.2			0

70906437																															70906437	TSTADYAMFK	151	N-Term(iTRAQ4plex); M8(Oxidation); K10(iTRAQ4plex)	1438.71318	1.606	1.528	1.456	1.122	1.859	1.769	1.762	1.264	21.0	21.6	15.5	12.2	8.2	11.5	25.1	20.9	0.0E+00	8.6E-05	3.2	77	1.2E-06	3.5	77	1.1E-06	0
70906437																															70906437	FEGNCAEQDGSGWWMNK	6	N-Term(iTRAQ4plex); C5(Methylthio); K17(iTRAQ4plex)	2292.97208	1.936	1.786	1.703	1.226	1.886	3.315	2.993	1.261	6.3		36.7		18.6		10.8		0.0E+00	7.4E-10	5.0	58	2.5E-05	5.7	62	1.1E-05	0
70906437																															70906437	YLQEIYNSNNQK	64	N-Term(iTRAQ4plex); N7(Deamidated); N9(Deamidated); K12(iTRAQ4plex)	1803.89873	1.911	1.505	1.523	1.149	1.742	1.886	1.561	1.248	5.8	22.8	15.6	24.1	9.3	16.4	11.1	28.3	0.0E+00	1.5E-06	4.2	66	2.3E-05	4.8	58	1.5E-04	0
70906437																															70906437	VAQLEAQCQEPCK	6	N-Term(iTRAQ4plex); Q7(Deamidated); C8(Methylthio); C12(Methylthio); K13(iTRAQ4plex)	1827.82988	1.695		1.423			2.793	2.298		7.2		29.9		9.0		2.2		0.0E+00	4.9E-03	3.9	25	1.1E-01				0
70906437																															70906437	FFTSHNGMQFSTWDNDNDK	2	N-Term(iTRAQ4plex); N6(Deamidated); M8(Oxidation); N15(Deamidated); K19(iTRAQ4plex)	2597.11356	1.457		1.445			2.127	2.166										0.0E+00	1.6E-05	4.9	43	1.0E-03				0
70906437																															70906437	QSGLYFIKPLK	4	N-Term(iTRAQ4plex); K8(iTRAQ4plex); K11(iTRAQ4plex)	1726.06235	1.994		1.967			1.912	1.937		0.0		2.0		6.3		4.3		0.0E+00	1.5E-05	5.0	47	4.5E-04				0
70906437																															70906437	EGFGHLSPTGTTEFWLGNEKIHLISTQSAIPYALR	1	N-Term(iTRAQ4plex); N18(Deamidated); K20(iTRAQ4plex); Q27(Deamidated)	4161.19306	0.899		5.011			0.261	1.496										0.0E+00	4.9E-04	4.2						1
70906437																															70906437	CHAGHLNGVYYQGGTYSK	2	N-Term(iTRAQ4plex); C1(Methylthio); K18(iTRAQ4plex)	2289.07583	1.298		1.198			1.518	1.438										0.0E+00	1.3E-04	3.8	10	8.1E+00				0
70906437																															70906437	IHLISTQSAIPYALR	2	N-Term(iTRAQ4plex)	1827.06699	1.633		1.692			1.167	1.241										0.0E+00	3.8E-05	4.9	45	1.9E-03				0
70906437																															70906437	AIQLTYNPDESSKPNMIDAATLK	2	N-Term(iTRAQ4plex); K13(iTRAQ4plex); N15(Deamidated); K23(iTRAQ4plex)	2953.56093	2.058		1.091			2.170	1.181										0.0E+00	2.7E-07	4.9	40	1.1E-02				0
70906437																															70906437	AIQLTYNPDESSKPNMIDAATLK	2	N-Term(iTRAQ4plex); N7(Deamidated); K13(iTRAQ4plex); N15(Deamidated); M16(Oxidation); K23(iTRAQ4plex)	2970.55166	1.216		0.903			1.179	0.899										0.0E+00	2.5E-06	3.1	34	5.9E-02				0
70906437																															70906437	AIQLTYNPDESSKPNMIDAATLK	3	N-Term(iTRAQ4plex); N7(Deamidated); K13(iTRAQ4plex); N15(Deamidated); K23(iTRAQ4plex)	2954.56753	1.996		0.931			1.688	0.809		51.9		5.3		29.6		25.6		0.0E+00	6.2E-07	5.6	18	1.9E+00				0
70906437																															70906437	EGFGHLSPTGTTEFWLGNEKIHLISTQSAIPYALR	3	N-Term(iTRAQ4plex); K20(iTRAQ4plex)	4159.19790	1.606		2.468			0.247	0.390		6.7		50.9		8.9		33.3		0.0E+00	3.1E-05	6.5	23	3.5E-01				1
88758615	NP_000410.2	ITGA2B	3674	integrin alpha-IIb preproprotein	GBC IIIA/Healthy; GBC IVB vs Healthy	IIIA and IVB	12	47	**0.445**	**0.432**	2.2E-16	0.911	0.904	1.8E-02	**0.485**	**0.449**	8.8E-17	0.970	1.030	1.2E-05	11.9	26.0	14.2	44.9	23.9	29.3	19.3	23.7	113.3	5.38	88758615																													
88758615																															88758615	NVGSQTLQTFK	4	N-Term(iTRAQ4plex); K11(iTRAQ4plex)	1510.84709	0.457	0.256	1.448	1.006	0.320	0.487	1.586	1.222									0.0E+00	1.5E-06	4.1	33	6.8E-02	5.4	68	2.0E-05	0
88758615																															88758615	IVLLDVPVR	3	N-Term(iTRAQ4plex)	1167.75981	0.441	0.370	0.873	1.020	0.449	0.483	0.981	1.206									0.0E+00	4.8E-03	2.8			3.4	29	1.5E-02	0
88758615																															88758615	ALSNVEGFER	4	N-Term(iTRAQ4plex)	1265.65886	0.387	0.470	0.896	0.878	0.630	0.378	0.899	1.148									0.0E+00	7.8E-04	4.1	53	5.6E-04	3.8	34	3.8E-02	0
88758615																															88758615	EQNSLDSWGPK	4	N-Term(iTRAQ4plex); K11(iTRAQ4plex)	1548.79411		0.537		1.139	0.550			1.137		20.8		18.7		23.8		15.8	0.0E+00	1.6E-03				3.4	46	2.2E-03	0
88758615																															88758615	TPVGSCFLAQPESGR	6	N-Term(iTRAQ4plex); C6(Methylthio)	1738.83757	0.357	0.473	0.718	0.691	0.713	0.427	0.880	1.015	36.5		12.9		35.5		62.0		0.0E+00	1.4E-06	4.7	99	1.0E-08	2.6	41	6.0E-03	0
88758615																															88758615	DGYNDIAVAAPYGGPSGR	7	N-Term(iTRAQ4plex)	1923.93804	0.902	0.273	0.986	0.760	0.436	0.794	0.891	0.993		20.8		54.4		25.4		24.5	0.0E+00	1.5E-06	2.1	22	6.1E-01	5.4	60	1.1E-04	0
88758615																															88758615	AEAQVELR	6	N-Term(iTRAQ4plex)	1059.59355	0.456	0.483	0.917	1.014	0.473	0.412	0.852	0.967		26.7		9.4		17.2		18.8	0.0E+00	1.7E-05	2.5	34	3.8E-02	3.2	44	4.2E-03	0
88758615																															88758615	NRPPLEEDDEEGE	3	N-Term(iTRAQ4plex)	1672.73589		0.368		0.895	0.396			0.937		23.9		42.8		37.4		18.8	3.0E-03	1.3E-01				3.8	47	2.5E-04	0
88758615																															88758615	FGSAIAPLGDLDR	2	N-Term(iTRAQ4plex)	1475.79411	0.319		1.095			0.401	1.415										0.0E+00	1.2E-05	4.5	67	1.6E-05				0
88758615																															88758615	VAIVVGAPR	2	N-Term(iTRAQ4plex)	1025.66069	0.449		1.099			0.526	1.319										0.0E+00	1.9E-03	2.7	27	1.3E-02				0
88758615																															88758615	SCVLPQTK	2	N-Term(iTRAQ4plex); C2(Methylthio); K8(iTRAQ4plex)	1209.66496	0.500		0.935			0.567	1.090										0.0E+00	9.4E-03	3.2	54	4.8E-04				0
88758615																															88758615	GEAQVWTQLLR	4	N-Term(iTRAQ4plex)	1444.80474	0.376		0.607			0.469	0.777		14.0		55.6		51.1		10.3		0.0E+00	2.4E-05	4.1	63	5.6E-05				0
89191868	NP_000543.2	VWF	7450	von Willebrand factor preproprotein	GBC IVB/GSD	IVB specific	49	511	0.926	0.910	9.9E-04	1.261	1.367	6.0E-54	1.443	1.516	6.3E-27	**2.039**	**2.218**	8.0E-98	28.8	20.6	12.8	17.2	17.3	18.9	38.1	32.4	309.1	5.49	89191868																													
89191868																															89191868	GEYFWEK	6	N-Term(iTRAQ4plex); K7(iTRAQ4plex)	1246.63384	1.050	1.025	1.322	1.797	2.137	2.445	3.159	3.651		9.8		2.6		3.6		3.5	0.0E+00	1.1E-03	3.2	26	1.9E-01	3.4	36	2.0E-02	0
89191868																															89191868	GDSQSSWK	10	N-Term(iTRAQ4plex); K8(iTRAQ4plex)	1182.60039	1.118	0.936	1.103	1.613	1.853	1.821	1.845	3.119		5.1		16.3		15.9		2.7	0.0E+00	1.8E-06	3.3	53	3.2E-04	3.6	54	2.9E-04	0
89191868																															89191868	EAPDLVLQR	17	N-Term(iTRAQ4plex)	1184.67295	0.711	0.804	0.939	1.593	1.623	1.116	1.518	2.982	0.4	14.8	41.3	2.1	8.3	2.1	31.8	7.0	0.0E+00	8.4E-03	4.0	40	8.2E-03	3.8	33	4.9E-02	0
89191868																															89191868	LPGDIQVVPIGVGPNANVQELER	4	N-Term(iTRAQ4plex)	2558.39957	1.164	0.833	1.197	1.416	1.761	1.451	1.532	2.917									0.0E+00	1.0E-08	5.6	79	1.4E-06	5.8	69	1.1E-05	0
89191868																															89191868	EYAPGETVK	44	N-Term(iTRAQ4plex); K9(iTRAQ4plex)	1281.69426	0.895	0.882	1.314	1.507	1.607	1.458	2.102	2.789	6.7	11.3	16.0	13.4	13.7	10.3	6.5	20.4	0.0E+00	1.5E-06	3.4	47	2.1E-03	3.6	48	1.8E-03	0
89191868																															89191868	ILAGPAGDSNVVK	22	N-Term(iTRAQ4plex); K13(iTRAQ4plex)	1528.89409	1.006	1.173	1.914	1.566	1.951	1.730	3.212	2.775	11.2	13.5	10.0	15.9	7.5	13.8	5.7	25.6	0.0E+00	1.9E-04	5.6	102	5.7E-09	5.6	102	5.4E-09	0
89191868																															89191868	VKEEVFIQQR	14	N-Term(iTRAQ4plex); K2(iTRAQ4plex)	1563.91257	0.877	0.976	1.220	1.429	1.619	1.402	2.038	2.627	8.7	0.7	20.2	39.6	8.6	35.4	5.4	10.8	0.0E+00	5.8E-05	5.1	42	4.5E-03	5.3	36	1.7E-02	1
89191868																															89191868	ILTSDVFQDCNK	6	N-Term(iTRAQ4plex); C10(Methylthio); K12(iTRAQ4plex)	1716.85393	0.617	1.031	1.352	1.556	1.760	1.327	2.988	2.587		12.4		25.4		25.7		12.1	0.0E+00	1.5E-06	5.0	70	1.1E-05	4.8	74	3.6E-06	0
89191868																															89191868	AFVLSSVDELEQQR	24	N-Term(iTRAQ4plex)	1764.93035	0.934	1.033	0.915	1.488	1.623	1.489	1.482	2.526	13.2	14.6	47.7	20.6	35.7	12.8	14.2	27.0	0.0E+00	4.2E-05	5.3	76	3.4E-06	5.4	71	1.1E-05	0
89191868																															89191868	TATLCPQSCEER	4	N-Term(iTRAQ4plex); C5(Methylthio); C9(Methylthio)	1573.65996	0.768	1.015	2.287	1.365	1.875	1.770	5.409	2.456									0.0E+00	6.5E-05	4.3	43	6.9E-04	4.4	40	1.3E-03	0

89191868																													89191868	IDRPEASR	8	N-Term(iTRAQ4plex)	1087.59748		0.882		1.552	1.639			2.452		9.7		14.9		19.6		22.0	0.0E+00	3.7E-02				3.1	17	1.8E+00	0
89191868																													89191868	VTVFPIGIGDR	14	N-Term(iTRAQ4plex)	1317.76457	1.030	0.756	1.173	1.338	1.413	1.720	1.994	2.435	19.3	1.7	15.5	1.5	19.1	0.7	11.2	2.6	0.0E+00	1.5E-06	3.5	30	1.0E-01	3.8	49	1.3E-03	0
89191868																													89191868	YDAAQLR	25	N-Term(iTRAQ4plex)	980.52849	0.809	0.823	1.015	1.351	1.452	1.299	1.519	2.416	0.4	10.3	5.9	24.6	20.5	15.7	13.2	12.2	2.0E-03	9.3E-02	2.8	23	3.2E-01	2.8	29	8.5E-02	0
89191868																													89191868	VSSQCADTR	23	N-Term(iTRAQ4plex); C5(Methylthio)	1156.52251	0.976	0.912	1.632	1.361	1.605	1.492	2.561	2.344	3.9	13.4	11.6	26.0	3.1	13.7	3.9	7.8	0.0E+00	1.7E-04	3.4	37	1.8E-03	3.4	38	1.4E-03	0
89191868																													89191868	AEGLECTK	10	N-Term(iTRAQ4plex); C6(Methylthio); K8(iTRAQ4plex)	1184.59062	1.073	0.982	2.404	1.734	1.354	1.136	2.613	2.329	9.2	2.3	41.3	2.7	29.7	12.5	19.9	1.9	0.0E+00	5.5E-03	3.0	36	1.2E-02	2.8	21	4.6E-01	0
89191868																													89191868	ICMDEDGNEK	7	N-Term(iTRAQ4plex); C2(Methylthio); M3(Oxidation); K10(iTRAQ4plex)	1503.64177	1.074	1.045	1.599	1.566	1.580	1.322	1.684	2.306	0.6		18.1		2.9		30.1		0.0E+00	1.2E-04	2.2	41	6.1E-04	2.5	44	2.9E-04	0
89191868																													89191868	GCSHPSVK	2	N-Term(iTRAQ4plex); C2(Methylthio); K8(iTRAQ4plex)	1148.57654		0.974		1.327	1.737			2.305									1.0E-03	1.0E-02				2.4	12	3.1E+00	0
89191868																													89191868	LASQVK	4	N-Term(iTRAQ4plex); K6(iTRAQ4plex)	933.59899	1.056	1.070	1.285	1.681	1.502	1.403	1.752	2.299	13.8		20.9		7.8		0.9		3.0E-03	1.2E-01		30	4.5E-02	2.0	23	2.0E-01	0
89191868																													89191868	ICMDEDGNEK	4	N-Term(iTRAQ4plex); C2(Methylthio); K10(iTRAQ4plex)	1487.64275	1.005	1.145	1.261	1.402	1.910	1.564	2.014	2.277									0.0E+00	1.8E-03	4.4	41	8.5E-04	3.8	53	5.1E-05	0
89191868																													89191868	GGQIMTLK	1	N-Term(iTRAQ4plex); M5(Oxidation); K8(iTRAQ4plex)	1151.67143		1.150		1.746	1.539			2.275									4.0E-03	2.5E-02					28	2.0E-01	0
89191868																													89191868	ENGYECEWR	6	N-Term(iTRAQ4plex); N2(Deamidated); C6(Methylthio)	1376.53630	1.001	0.980	1.277	1.341	1.686	1.417	1.856	2.246	0.3		8.8		9.2		0.0		0.0E+00	1.5E-06	3.8	34	8.3E-04	3.9	34	8.1E-04	0
89191868																													89191868	YAGSQVASTSEVLK	20	N-Term(iTRAQ4plex); K14(iTRAQ4plex)	1727.94267	0.900	1.091	1.582	1.670	1.640	1.448	2.614	2.227	17.6	13.5	14.6	20.4	9.2	6.1	13.0	14.1	0.0E+00	1.9E-09	5.9	77	3.2E-06	6.1	83	6.7E-07	0
89191868																													89191868	AFVVDMMER	2	N-Term(iTRAQ4plex); M6(Oxidation); M7(Oxidation)	1273.60198		0.962		1.312	1.657			2.200									0.0E+00	8.8E-04				2.3	29	3.5E-02	0
89191868																													89191868	DGTVTTDWK	30	N-Term(iTRAQ4plex); K9(iTRAQ4plex)	1310.68425	1.020	0.876	1.349	1.311	1.590	1.524	2.342	2.162	10.3	24.3	23.5	15.9	19.8	15.3	9.1	20.5	0.0E+00	1.6E-06	3.9	48	1.6E-03	3.9	49	1.3E-03	0
89191868																													89191868	EENNTGECCGR	1	N-Term(iTRAQ4plex); C8(Methylthio); C9(Methylthio)	1447.51641		0.736		1.303	1.247			2.149									1.0E-03	7.6E-02				2.0			0
89191868																													89191868	EGGPSQIGDALGFAVR	15	N-Term(iTRAQ4plex)	1717.90312	0.922	0.974	1.160	1.366	1.650	1.184	1.466	2.116	14.2	7.6	63.5	27.7	38.7	23.2	14.8	17.1	0.0E+00	7.8E-10	6.6	108	2.2E-09	5.5	78	2.5E-06	0
89191868																													89191868	EEVFIQQR	6	N-Term(iTRAQ4plex)	1192.64641	0.895	0.909	1.065	1.459	1.353	1.850	2.260	2.115		10.9		9.1		6.2		8.0	0.0E+00	7.5E-04	3.5	32	5.9E-02	3.5	34	3.9E-02	0
89191868																													89191868	LSEAEFEVLK	9	N-Term(iTRAQ4plex); K10(iTRAQ4plex)	1452.82097	1.055	1.041	1.978	1.375	1.722	1.817	3.497	2.079		2.1		3.9		9.7		4.8	0.0E+00	1.0E-04	3.7	48	2.3E-03	4.4	67	2.6E-05	0
89191868																													89191868	SVGSQWASPENPCLINECVR	1	N-Term(iTRAQ4plex); N11(Deamidated); C13(Methylthio); N16(Deamidated); C18(Methylthio)	2427.08091		0.821		1.260	1.368			2.043									4.0E-03	1.4E-01				1.8			0
89191868																													89191868	AFVVDMMER	6	N-Term(iTRAQ4plex); M6(Oxidation)	1257.60881	0.802	0.964	1.106	1.334	1.508	1.241	1.760	2.032		15.9		32.3		28.2		19.8	0.0E+00	4.2E-06	1.8	34	1.9E-02	2.3	29	5.6E-02	0
89191868																													89191868	QTMVDSSCR	6	N-Term(iTRAQ4plex); C8(Methylthio)	1216.52373		0.826		1.197	1.416			2.000		25.2		9.5		10.0		27.1	0.0E+00	5.6E-03				3.0	37	2.5E-03	0
89191868																													89191868	LVCPADNLR	10	N-Term(iTRAQ4plex); C3(Methylthio)	1190.61479	0.943	0.750	1.397	1.158	1.327	1.620	2.464	1.994	0.3	0.5	6.7	15.3	0.1	2.4	5.0	17.3	0.0E+00	4.9E-03	2.9	37	1.6E-02	2.9	27	1.5E-01	0
89191868																													89191868	EQAPNLVYMVTGNPASDEIK	4	N-Term(iTRAQ4plex); M9(Oxidation); K20(iTRAQ4plex)	2480.26114	1.029	0.745	1.107	1.008	1.499	1.480	1.636	1.976									0.0E+00	4.5E-08	5.0	52	9.2E-04	3.8	42	9.5E-03	0
89191868																													89191868	IEDLPTMVTLGNSFLHK	2	N-Term(iTRAQ4plex); M7(Oxidation); K17(iTRAQ4plex)	2219.19577		0.622		0.998	1.264			1.976									0.0E+00	1.5E-06				3.8	36	3.3E-02	0
89191868																													89191868	QTMVDSSCR	4	N-Term(iTRAQ4plex); M3(Oxidation); C8(Methylthio)	1232.52165	0.790	1.032	1.038	1.195	1.718	1.322	1.784	1.938									0.0E+00	1.5E-02	2.1	34	1.5E-03	2.2	25	1.5E-02	0
89191868																													89191868	TNTGLALR	7	N-Term(iTRAQ4plex)	989.58678		0.850		1.245	1.346			1.934		8.4		25.6		7.2		14.3	1.0E-03	1.3E-02				3.1	38	5.7E-03	0
89191868																													89191868	EQAPNLVYMVTGNPASDEIK	18	N-Term(iTRAQ4plex); K20(iTRAQ4plex)	2464.26175	0.840	0.808	0.897	1.345	1.410	1.105	1.210	1.895	5.0	11.2	121.5	33.9	51.1	21.8	55.8	30.1	0.0E+00	2.6E-14	6.0	47	3.3E-03	5.9	62	1.0E-04	0
89191868																													89191868	AFVVDMMER	10	N-Term(iTRAQ4plex)	1241.61528	0.904	0.825	1.132	1.111	1.433	1.381	1.851	1.880	11.5	18.3	4.0	33.0	2.7	25.2	5.3	26.0	0.0E+00	1.5E-06	3.5	30	5.6E-02	3.4	38	9.3E-03	0
89191868																													89191868	GGQIMTLK	8	N-Term(iTRAQ4plex); K8(iTRAQ4plex)	1135.67632	0.935	1.014	1.222	1.315	1.507	2.293	3.077	1.862		1.3		52.0		49.2		4.5	0.0E+00	4.1E-05	3.3	35	3.3E-02	3.5	36	2.2E-02	0
89191868																													89191868	CLAEGGK	8	N-Term(iTRAQ4plex); C1(Methylthio); K7(iTRAQ4plex)	1011.52104	0.885	0.968	2.549	1.480	1.226	1.268	3.749	1.833	11.2	20.2	42.2	23.3	30.4	22.3	0.4	22.7	1.0E-03	1.5E-02	2.8	44	2.2E-03	2.7	41	4.2E-03	0
89191868																													89191868	EFMEEVIQR	8	N-Term(iTRAQ4plex)	1324.66875	0.992	0.717	1.159	1.287	1.042	1.625	1.945	1.822	3.2		39.6		35.1		2.9		0.0E+00	1.5E-06	4.1	45	2.1E-03	4.0	36	2.1E-02	0
89191868																													89191868	YLSDHSFLVSQGDR	20	N-Term(iTRAQ4plex)	1767.88274	0.876	0.912	0.961	1.092	1.413	1.380	1.449	1.813	13.1	5.2	26.1	43.4	12.1	40.0	15.0	0.2	0.0E+00	1.5E-06	4.9	39	1.4E-02	4.8	52	7.2E-04	0
89191868																													89191868	ICMDEDGNEK	2	N-Term(iTRAQ4plex); C2(Methylthio); M3(Oxidation); N8(Deamidated); K10(iTRAQ4plex)	1504.62187		0.782		1.019	1.365			1.733									0.0E+00	9.5E-06				1.8	39	1.2E-03	0
89191868																													89191868	ILAGPAGDSNVVK	5	N-Term(iTRAQ4plex); N10(Deamidated); K13(iTRAQ4plex)	1529.88042		0.840		1.145	1.293			1.674		5.4		17.6		7.4		13.8	0.0E+00	1.2E-07				4.6	105	3.0E-09	0
89191868																													89191868	EFMEEVIQR	11	N-Term(iTRAQ4plex); M3(Oxidation)	1340.66374	0.879	0.885	1.217	1.315	0.940	1.292	1.848	1.639	0.8	5.1	30.0	7.7	19.5	6.1	0.3	5.2	0.0E+00	7.5E-04	2.7	31	6.1E-02	1.9	39	1.1E-02	0
89191868																													89191868	SKEFMEEVIQR	2	N-Term(iTRAQ4plex); K2(iTRAQ4plex); M5(Oxidation)	1699.89420		0.861		0.972	1.483			1.630									0.0E+00	2.1E-04				3.5	33	6.1E-02	1
89191868																													89191868	SVGSQWASPENPCLINECVR	2	N-Term(iTRAQ4plex); C13(Methylthio); C18(Methylthio)	2425.08414		1.055		1.269	1.198			1.403									0.0E+00	1.5E-06				4.5	32	2.4E-02	0

89191868																															89191868	RLPGDIQVVPIGVGPNANVQELER	3	N-Term(iTRAQ4plex)	2714.51969	0.926	0.968	1.119	1.119	1.229	1.037	1.288	1.382									0.0E+00	2.2E-06	6.7	61	5.4E-05	3.6			1
89191868																															89191868	GLRPSCPNSQSPVK	5	N-Term(iTRAQ4plex); C6(Methylthio); K14(iTRAQ4plex)	1803.94590	0.796	0.985	0.801	1.029	1.344	0.946	0.978	1.367	13.8		22.0		17.1		18.6		0.0E+00	1.5E-06	4.7	13	6.2E+00	4.6	21	9.5E-01	0
89191868																															89191868	CLPSACEVVTGSPR	1	N-Term(iTRAQ4plex); C1(Methylthio); C6(Methylthio)	1654.75444		1.123		1.243	1.235			1.332									3.0E-03	1.3E-01				1.4			0
89191868																															89191868	VTGCPPFDEHK	2	N-Term(iTRAQ4plex); C4(Methylthio); K11(iTRAQ4plex)	1563.75565		0.927		0.948	1.301			1.295									0.0E+00	2.7E-02				3.3	14	2.8E+00	0
89191868																															89191868	EQDLEVILHNGACSPGAR	3	N-Term(iTRAQ4plex); N10(Deamidated); C13(Methylthio)	2099.99704	0.992	0.725	0.850	0.830	1.152	0.911	0.802	1.284									0.0E+00	3.6E-05	4.1	44	3.1E-03	1.8			0
89191868																															89191868	SGFTYVLHEGECCGR	4	N-Term(iTRAQ4plex); C12(Methylthio); C13(Methylthio)	1893.78897	1.033	0.899	1.049	1.033	0.975	1.344	1.402	1.092									0.0E+00	2.8E-05	3.7	28	2.0E-02	3.3	11	9.7E-01	0
89191868																															89191868	DETHFEVVESGR	6	N-Term(iTRAQ4plex)	1548.73789	0.870	0.932	0.851	0.861	1.142	1.088	1.092	1.027		16.0		8.1		24.5		0.1	0.0E+00	1.5E-06	4.2	28	8.8E-02	4.4	36	1.3E-02	0
89191868																															89191868	EQAPNLVYMVTGNPASDEIK	1	N-Term(iTRAQ4plex); M9(Oxidation); N13(Deamidated); K20(iTRAQ4plex)	2481.24973		0.708		0.634	0.967			0.844									0.0E+00	7.8E-03				3.1			0
89191868																															89191868	LPGDIQVVPIGVGPNANVQELER	2	N-Term(iTRAQ4plex); N15(Deamidated)	2559.38761		1.086		0.894	0.811			0.650									0.0E+00	1.5E-06				4.9	32	6.6E-02	0
89191868																															89191868	LCSGFVR	1	N-Term(iTRAQ4plex); C2(Methylthio)	971.49211	0.894		4.510			1.548	8.015										9.0E-03	1.0E-01		20	4.3E-01				0
89191868																															89191868	VEETCGCR	3	N-Term(iTRAQ4plex); C5(Methylthio); C7(Methylthio)	1132.43877	0.820		1.722			1.617	3.487		5.4		47.9		37.3		4.0		0.0E+00	1.3E-02	2.8	17	2.0E-02				0
89191868																															89191868	TTCNPCPLGYK	3	N-Term(iTRAQ4plex); C3(Methylthio); C6(Methylthio); K11(iTRAQ4plex)	1576.72515	0.717		1.045			2.150	3.215		51.4		164.1		104.5		89.6		0.0E+00	1.7E-06	4.0	70	3.7E-06				0
89191868																															89191868	SLSCRPPMVK	2	N-Term(iTRAQ4plex); C4(Methylthio); K10(iTRAQ4plex)	1451.77915	0.854		1.533			1.484	2.736										0.0E+00	1.1E-02	4.3	22	6.0E-01				0
89191868																															89191868	APTCGLCEVAR	2	N-Term(iTRAQ4plex); C4(Methylthio); C7(Methylthio)	1355.60735	1.012		1.418			1.785	2.568										0.0E+00	6.4E-03	3.1	25	8.4E-02				0
89191868																															89191868	LVCPADNLR	1	N-Term(iTRAQ4plex); C3(Methylthio); N7(Deamidated)	1191.59990	0.974		1.192			1.425	1.792										4.0E-03	6.0E-02	2.5						0
93141226	NP_003390.4	XPNPEP2	7512	xaa-Pro aminopeptidase 2 precursor	GBC IIIA/Healthy, GSD	IIIA specific	2	5	**3.244**	**2.736**	2.6E-04	**2.850**	**2.925**	2.4E-04	**2.116**	1.771	1.9E-02	1.908	1.844	3.3E-02		28.2		18.0		7.0		16.9	75.6	6.04	93141226																													
93141226																															93141226	LIFPAATSGR	4	N-Term(iTRAQ4plex)	1176.68547		2.736		2.925	1.771			1.844		18.0		16.9		28.2		7.0	0.0E+00	1.3E-04				2.8	19	1.7E+00	0
93141226																															93141226	VGPELQR	1	N-Term(iTRAQ4plex)	942.55071	3.244		2.850			2.116	1.908										8.0E-03	1.1E-01	2.5						0
105990532	NP_000375.2	APOB	338	apolipoprotein B-100 precursor	GBC IIIA/GSD	IIIA specific	136	841	1.271	1.263	1.1E-167	**2.344**	**2.342**	3.4E-291	0.988	1.056	2.0E-99	**1.988**	1.922	1.1E-58	51.8	46.3	16.6	19.3	17.0	20.1	48.0	42.9	515.2	7.05	105990532																													
105990532																															105990532	SISAALEHK	2	N-Term(iTRAQ4plex); K9(iTRAQ4plex)	1243.72611		1.682		5.232	1.612			4.882									0.0E+00	1.5E-06				4.3	35	2.5E-02	0
105990532																															105990532	SEYQADYESLR	12	N-Term(iTRAQ4plex)	1504.70549	1.644	1.647	3.410	4.748	1.552	1.245	2.988	4.358	4.5	12.7	13.5	50.3	12.4	64.6	0.8	1.1	0.0E+00	6.2E-04	4.6	54	1.4E-04	5.1	58	5.6E-05	0
105990532																															105990532	ESDEETQIK	5	N-Term(iTRAQ4plex); K9(iTRAQ4plex)	1366.69682	1.142	1.462	2.724	5.362	1.165	0.802	1.966	4.160	7.3		51.7		45.3		12.9		0.0E+00	1.5E-06	3.6	39	1.1E-02	4.4	44	4.1E-03	0
105990532																															105990532	FPEVDVLTK	8	N-Term(iTRAQ4plex); K9(iTRAQ4plex)	1335.77751	1.477	1.680	3.648	5.499	1.165	1.129	2.615	3.714	10.0		2.7		20.8		2.8		0.0E+00	6.0E-04	3.6	51	1.0E-03	3.6	54	5.0E-04	0
105990532																															105990532	GAYQNNEIK	4	N-Term(iTRAQ4plex); K9(iTRAQ4plex)	1324.71135	1.815	2.016	3.509	3.774	2.009	1.565	3.106	3.662									0.0E+00	1.5E-06	4.3	50	1.3E-03	4.2	39	1.7E-02	0
105990532																															105990532	AQNLYQELLTQEGQASFQGLK	4	N-Term(iTRAQ4plex); K21(iTRAQ4plex)	2654.40653	0.746	1.722	1.806	5.362	1.194	0.736	1.829	3.622									0.0E+00	1.5E-14	3.3	29	1.5E-01	5.5	75	3.6E-06	0
105990532																															105990532	VSQEGLK	7	N-Term(iTRAQ4plex); K7(iTRAQ4plex)	1048.62627	1.618	1.862	2.580	3.949	1.677	1.417	2.321	3.464	18.5	12.6	58.8	6.3	64.3	8.5	14.0	2.2	1.0E-03	8.0E-02	2.9	38	1.7E-02	2.8	38	2.3E-02	0
105990532																															105990532	VSSFYAK	6	N-Term(iTRAQ4plex); K7(iTRAQ4plex)	1089.61980	1.879	1.840	2.813	4.292	1.488	1.310	2.012	3.381		2.9		33.6		23.4		6.7	0.0E+00	6.5E-03	2.6	54	4.1E-04	3.0	54	3.4E-04	0
105990532																															105990532	AAIQALR	8	N-Term(iTRAQ4plex)	886.56047	1.164	1.193	2.208	4.258	0.973	0.989	1.925	3.380	4.2	0.2	6.5	11.2	8.9	10.1	6.6	0.9	1.0E-03	1.1E-02	2.8	31	1.1E-02	2.8	33	8.2E-03	0
105990532																															105990532	INDVLEHVK	4	N-Term(iTRAQ4plex); K9(iTRAQ4plex)	1354.79459	1.169	1.434	2.950	4.400	1.122	0.975	2.525	3.354									0.0E+00	1.5E-06	3.4	23	5.1E-01	3.9	35	3.3E-02	0
105990532																															105990532	LDFSSQADLR	8	N-Term(iTRAQ4plex)	1295.67192	1.245	1.413	2.274	3.102	1.546	1.171	2.173	3.304	1.0		3.4		25.3		14.6		0.0E+00	8.6E-04	4.4	42	6.2E-03	3.8	42	6.2E-03	0
105990532																															105990532	NIILPVYDK	12	N-Term(iTRAQ4plex); K9(iTRAQ4plex)	1362.82439	1.425	1.499	4.107	4.260	1.190	1.034	3.059	3.296	8.1	2.2	19.4	21.0	10.5	21.2	17.0	2.0	0.0E+00	2.4E-04	3.4	72	3.8E-06	3.2	62	4.2E-05	0
105990532																															105990532	LVELAHQYK	6	N-Term(iTRAQ4plex); K9(iTRAQ4plex)	1388.81586	1.304	1.111	3.100	4.117	0.897	1.015	2.477	3.235	0.9		30.8		41.4		8.8		0.0E+00	1.5E-06	4.0	31	7.5E-02	4.2	33	4.9E-02	0
105990532																															105990532	SLWDFLK	2	N-Term(iTRAQ4plex); K7(iTRAQ4plex)	1196.69341		1.356		4.511	0.981			3.179									0.0E+00	5.1E-02				3.0	38	1.6E-02	0
105990532																															105990532	DFSLWEK	10	N-Term(iTRAQ4plex); K7(iTRAQ4plex)	1212.65129	1.436	1.449	3.193	3.894	1.192	1.084	2.220	3.120	12.6	2.5	0.9	5.2	15.7	16.6	0.9	8.9	0.0E+00	6.0E-03	3.3	30	8.7E-02	3.1	34	3.8E-02	0
105990532																															105990532	TLADLTLLDSPIK	4	N-Term(iTRAQ4plex); K13(iTRAQ4plex)	1688.00957		1.637		3.768	1.390			3.118		8.2		1.5		11.2		1.4	0.0E+00	1.1E-06				4.9	85	2.1E-07	0
105990532																															105990532	NFATSNK	4	N-Term(iTRAQ4plex); K7(iTRAQ4plex)	1069.58928	1.391	1.510	6.204	3.756	1.276	1.230	5.632	3.090									4.0E-03	6.1E-02	2.7	36	2.2E-02	2.3	30	1.0E-01	0
105990532																															105990532	GVISIPR	2	N-Term(iTRAQ4plex)	885.56438		1.386		3.131	1.395			3.068									4.0E-03	2.5E-02				2.9	34	2.3E-03	0

105990532																															105990532	FLDMLIK	1	N-Term(iTRAQ4plex); M4(Oxidation); K7(iTRAQ4plex)	1183.70171		1.389		4.635	0.909			2.956									7.0E-03	6.4E-02					21	6.6E-01	0
105990532																															105990532	QTEATMTFK	8	N-Term(iTRAQ4plex); K9(iTRAQ4plex)	1344.70940	1.364	1.400	1.834	3.557	1.191	0.961	1.327	2.946		11.1		0.1		22.0		11.3	0.0E+00	1.5E-06	3.2	52	5.4E-04	3.7	76	2.1E-06	0
105990532																															105990532	ALVDTLK	8	N-Term(iTRAQ4plex); K7(iTRAQ4plex)	1047.66643	1.139	1.609	2.408	4.086	1.188	0.919	1.996	2.937	15.1	27.9	32.3	17.3	47.2	25.5	1.7	19.7	0.0E+00	6.2E-03	3.2	45	1.3E-03	3.3	44	2.3E-03	0
105990532																															105990532	QSFDLSVK	4	N-Term(iTRAQ4plex); K8(iTRAQ4plex)	1211.68608	1.533	1.624	2.987	3.509	1.385	1.352	2.706	2.914									0.0E+00	4.4E-06	3.4	45	2.6E-03	3.6	49	9.4E-04	0
105990532																															105990532	LGNNPVSK	9	N-Term(iTRAQ4plex); K8(iTRAQ4plex)	1116.66289	1.615	1.687	2.262	3.493	1.507	1.249	1.797	2.870	3.2	8.5	1.2	41.3	6.1	40.0	4.1	0.7	0.0E+00	1.6E-05	3.1	54	2.9E-04	3.7	51	5.6E-04	0
105990532																															105990532	NSEEFAAAMSR	2	N-Term(iTRAQ4plex)	1356.63371		1.538		3.586	1.262			2.866									0.0E+00	1.5E-06				4.9	58	3.8E-05	0
105990532																															105990532	LAIPEGK	4	N-Term(iTRAQ4plex); K7(iTRAQ4plex)	1015.63848	1.310	1.518	2.899	3.866	1.145	1.019	2.316	2.842	20.7		0.9		6.0		13.6		1.0E-03	3.0E-02	2.6	43	1.7E-03		42	3.6E-03	0
105990532																															105990532	HVAEAICK	2	N-Term(iTRAQ4plex); C7(Methylthio); K8(iTRAQ4plex)	1204.64060		1.470		3.008	1.423			2.836									3.0E-03	2.3E-02				2.4	24	3.8E-01	0
105990532																															105990532	FFGEGTK	7	N-Term(iTRAQ4plex); K7(iTRAQ4plex)	1073.58806	1.363	1.432	5.385	4.084	1.091	1.036	4.200	2.813	0.9	11.1	8.9	16.2	13.2	3.8	3.3	1.3	3.0E-03	1.3E-01		50	8.2E-04	2.5	34	3.3E-02	0
105990532																															105990532	GAYQNNEIK	4	N-Term(iTRAQ4plex); N5(Deamidated); K9(iTRAQ4plex)	1325.69451		1.828		2.170	2.407			2.782		7.8		10.1		32.5		13.9	0.0E+00	3.9E-02				2.7	41	1.0E-02	0
105990532																															105990532	FVTQAEGAK	12	N-Term(iTRAQ4plex); K9(iTRAQ4plex)	1238.69939	1.198	1.630	2.382	3.410	1.297	1.013	1.917	2.643	11.3	6.8	26.0	6.3	30.5	9.4	6.0	3.7	0.0E+00	1.5E-06	3.7	66	3.6E-05	3.9	69	1.5E-05	0
105990532																															105990532	FLDSNIK	3	N-Term(iTRAQ4plex); K7(iTRAQ4plex)	1124.65703	1.718	1.485	2.714	2.873	1.374	1.549	2.511	2.588									5.0E-03	4.7E-02	2.4			2.6	25	3.8E-01	0
105990532																															105990532	LVGFIDDAVK	4	N-Term(iTRAQ4plex); K10(iTRAQ4plex)	1364.80486	1.271	1.452	2.559	3.609	1.067	0.964	1.993	2.583									0.0E+00	1.5E-06	3.5	48	1.4E-03	4.0	72	5.6E-06	0
105990532																															105990532	EAQEVFK	11	N-Term(iTRAQ4plex); K7(iTRAQ4plex)	1138.63542	1.534	1.580	3.078	3.445	1.175	1.242	2.558	2.578	3.5	15.2	13.0	12.5	15.9	18.3	0.7	17.0	0.0E+00	4.6E-02	3.3	43	3.7E-03	3.4	43	3.9E-03	0
105990532																															105990532	LHVAGNLK	4	N-Term(iTRAQ4plex); K8(iTRAQ4plex)	1139.71387	1.373	1.361	3.031	2.388	1.507	1.326	3.006	2.574									0.0E+00	1.0E-02	3.2	29	5.6E-02	1.9	22	3.7E-01	0
105990532																															105990532	QVFLYPEK	6	N-Term(iTRAQ4plex); K8(iTRAQ4plex)	1311.75688	1.062	1.479	1.679	3.819	1.004	0.957	1.553	2.526	12.9		31.2		32.1		12.1		0.0E+00	6.4E-05	3.0	35	3.2E-02	3.1	46	3.1E-03	0
105990532																															105990532	NSEEFAAAMSR	4	N-Term(iTRAQ4plex); M9(Oxidation)	1372.63103	1.182	1.372	3.370	2.898	1.222	0.628	1.840	2.513									0.0E+00	1.4E-03	3.1	58	3.0E-05	2.5	51	1.5E-04	0
105990532																															105990532	TPALHFK	3	N-Term(iTRAQ4plex); K7(iTRAQ4plex)	1101.66822	1.237	1.276	3.403	3.336	0.985	1.081	3.052	2.507									0.0E+00	5.0E-03	2.7	30	6.8E-02		26	1.5E-01	0
105990532																															105990532	QGFFPDSVNK	16	N-Term(iTRAQ4plex); K10(iTRAQ4plex)	1426.75859	1.236	1.492	2.999	3.275	1.137	0.973	2.371	2.429	15.2	4.0	67.6	13.8	72.8	17.6	10.5	0.3	0.0E+00	1.7E-03	4.2	65	3.0E-05	4.2	65	3.1E-05	0
105990532																															105990532	QAEAVLK	4	N-Term(iTRAQ4plex); K7(iTRAQ4plex)	1046.64678		1.359		3.092	1.082			2.396		2.5		33.5		30.8		5.0	2.0E-03	2.1E-02				3.0	43	5.5E-03	0
105990532																															105990532	DSYDLHDLK	2	N-Term(iTRAQ4plex); K9(iTRAQ4plex)	1393.72153		1.287		3.049	1.023			2.361									0.0E+00	2.3E-03				3.2	23	6.1E-01	0
105990532																															105990532	VSALLTPAEQTGTWK	4	N-Term(iTRAQ4plex); K15(iTRAQ4plex)	1890.06131	1.138	0.954	1.190	2.510	0.912	1.426	1.531	2.338									0.0E+00	3.0E-03	2.3	13	5.2E+00	2.2	14	3.5E+00	0
105990532																															105990532	ENFAGEATLQR	6	N-Term(iTRAQ4plex)	1379.70464		1.436		2.822	1.216			2.327		10.9		41.0		37.5		14.1	0.0E+00	1.5E-06				4.2	51	7.6E-04	0
105990532																															105990532	LSNVLQQVK	4	N-Term(iTRAQ4plex); K9(iTRAQ4plex)	1316.81572	1.264	1.136	3.476	2.991	0.901	0.875	2.473	2.312									0.0E+00	3.1E-04	4.1	54	2.8E-04	3.6	50	6.9E-04	0
105990532																															105990532	LNELSFK	4	N-Term(iTRAQ4plex); K7(iTRAQ4plex)	1138.67217	1.165	1.709	2.323	3.436	1.176	1.045	2.140	2.302									1.0E-03	1.3E-02	3.0	43	4.4E-03	2.9	47	1.8E-03	0
105990532																															105990532	EIFNMAR	6	N-Term(iTRAQ4plex)	1024.53923	1.068	1.141	1.769	2.803	0.930	0.973	1.655	2.226		5.6		26.4		38.4		5.5	2.0E-03	1.1E-01	2.8	27	1.0E-01	3.1	33	2.5E-02	0
105990532																															105990532	LNGEIQALELPQK	8	N-Term(iTRAQ4plex); N2(Deamidated); K13(iTRAQ4plex)	1741.99236	1.283	1.218	2.884	2.973	0.962	0.973	2.246	2.206		15.4		33.9		12.7		18.1	0.0E+00	1.5E-06	3.9	36	2.5E-02	4.7	50	9.8E-04	0
105990532																															105990532	IISDYHQQFR	5	N-Term(iTRAQ4plex)	1450.75660	1.192	1.181	1.492	2.358	1.133	0.985	1.267	2.204		4.1		16.2		33.6		12.6	0.0E+00	1.5E-06	4.3	37	1.9E-02	4.1	38	1.4E-02	0
105990532																															105990532	VSTAFVYTK	4	N-Term(iTRAQ4plex); K9(iTRAQ4plex)	1303.75176	1.722	1.579	3.360	3.150	1.128	1.153	2.311	2.193									0.0E+00	1.1E-04	4.1	65	3.8E-05	3.3	53	6.1E-04	0
105990532																															105990532	IEIPLPFGGK	6	N-Term(iTRAQ4plex); K10(iTRAQ4plex)	1358.82959		1.286		3.278	1.127			2.170		12.8		27.5		16.4		33.2	0.0E+00	9.9E-03				3.4	59	5.2E-05	0
105990532																															105990532	LSLPDFK	6	N-Term(iTRAQ4plex); K7(iTRAQ4plex)	1107.66631	1.359	1.470	2.989	2.403	1.338	1.310	2.958	2.130	5.8		9.6		33.8		17.6		0.0E+00	8.9E-03	2.6	56	2.3E-04	2.2	35	2.7E-02	0
105990532																															105990532	LTISEQNIQR	9	N-Term(iTRAQ4plex)	1345.75542	1.082	1.138	1.952	3.017	0.928	0.841	1.558	2.124	14.9	12.1	31.4	18.3	36.5	16.8	10.2	0.1	0.0E+00	3.2E-03	3.8	30	9.9E-02	4.3	44	4.2E-03	0
105990532																															105990532	SPSQADINK	22	N-Term(iTRAQ4plex); K9(iTRAQ4plex)	1247.68523	1.260	1.177	2.000	2.489	0.947	1.044	1.529	2.119	1.2	11.9	26.5	36.6	17.2	26.1	11.1	14.9	0.0E+00	1.9E-06	3.7	63	4.6E-05	3.7	66	2.4E-05	0
105990532																															105990532	LQDFSDQLSDYYEK	6	N-Term(iTRAQ4plex); K14(iTRAQ4plex)	2038.98503	0.989	1.181	2.344	3.014	0.849	1.432	3.484	2.109		5.0		43.6		31.3		16.2	0.0E+00	5.6E-03	2.1	14	4.1E+00	3.6	38	1.4E-02	0
105990532																															105990532	TSQCTLK	4	N-Term(iTRAQ4plex); C4(Methylthio); K7(iTRAQ4plex)	1114.58574	1.032	1.499	1.663	2.342	1.379	1.047	1.732	2.098									2.0E-03	3.6E-02	2.5	31	4.1E-02	2.5	24	2.0E-01	0
105990532																															105990532	AASGTTGTYQEWK	12	N-Term(iTRAQ4plex); K13(iTRAQ4plex)	1687.85600	1.296	0.988	4.258	2.731	0.934	0.971	3.277	2.087		2.2		30.0		24.3		16.8	0.0E+00	7.7E-07	6.7	79	1.4E-06	5.4	80	1.1E-06	0
105990532																															105990532	ESQLPTVMDFR	6	N-Term(iTRAQ4plex); M8(Oxidation)	1482.73699	1.332	1.163	1.897	2.494	0.999	0.844	1.235	2.086		4.9		3.3		15.8		7.5	0.0E+00	2.6E-02	1.6	42	5.6E-03	2.8	39	9.9E-03	0

105990532																												105990532	FQFPGKPGIYTR	2	N-Term(iTRAQ4plex); K6(iTRAQ4plex)	1698.95707		1.151		2.501	0.978			2.070									0.0E+00	1.6E-06				3.7	23	5.0E-01	0
105990532																												105990532	MYQMDIQQELQR	8	N-Term(iTRAQ4plex)	1726.84148	1.096	1.194	2.246	2.344	1.081	0.944	1.925	2.067	0.6		18.1		69.7		5.1		0.0E+00	1.5E-06	3.6	55	2.3E-04	4.4	67	1.3E-05	0
105990532																												105990532	QTEATMTFK	2	N-Term(iTRAQ4plex); M6(Oxidation); K9(iTRAQ4plex)	1360.70366		1.532		2.710	1.186			2.044									2.0E-03	9.4E-02				2.0	42	5.1E-03	0
105990532																												105990532	ATGVLYDYVNK	12	N-Term(iTRAQ4plex); K11(iTRAQ4plex)	1530.84270	1.431	1.311	3.101	2.372	1.112	1.115	2.398	2.030	7.2	1.0	32.3	4.9	17.8	16.7	2.1	1.3	0.0E+00	1.6E-02	4.9	67	2.5E-05	4.8	64	4.2E-05	0
105990532																												105990532	MYQMDIQQELQR	2	N-Term(iTRAQ4plex); M4(Oxidation)	1742.83635		0.959		2.059	0.959			2.007									0.0E+00	5.2E-06				3.0	41	5.8E-03	0
105990532																												105990532	LTLDIQNK	6	N-Term(iTRAQ4plex); K8(iTRAQ4plex)	1232.74834	1.509	1.625	3.454	2.343	1.427	1.212	2.848	2.003	9.7		30.8		37.4		3.6		0.0E+00	3.4E-02	3.6	47	1.6E-03	3.3	46	1.9E-03	0
105990532																												105990532	VTQEFHMK	4	N-Term(iTRAQ4plex); K8(iTRAQ4plex)	1307.70407		1.306		2.627	1.021			2.001		20.1		57.2		80.6		2.4	0.0E+00	4.9E-02				3.3	29	1.5E-01	0
105990532																												105990532	IGVELTGR	5	N-Term(iTRAQ4plex)	988.59130	1.522	1.376	3.431	2.266	1.213	1.152	2.666	1.945		3.5		5.3		20.0		11.0	0.0E+00	9.2E-04	3.1	37	1.4E-02	3.0	32	4.7E-02	0
105990532																												105990532	YYELEEK	1	N-Term(iTRAQ4plex); K7(iTRAQ4plex)	1261.65911		1.189		2.127	1.101			1.917									9.0E-03	8.2E-02					31	7.3E-02	0
105990532																												105990532	QIDDIDVR	8	N-Term(iTRAQ4plex)	1117.60112	1.157	1.404	2.230	2.190	1.260	0.962	1.791	1.915	5.4		18.7		0.3		9.4		0.0E+00	2.1E-03	3.1	39	1.5E-02	3.0	41	9.6E-03	0
105990532																												105990532	DLGQCDR	8	N-Term(iTRAQ4plex); C5(Methylthio)	996.43773	1.117	1.205	1.724	2.537	0.929	0.884	1.401	1.906	1.3	7.3	14.5	0.4	7.6	0.8	3.5	7.7	3.0E-03	5.5E-02	3.0	25	2.5E-02	2.8	27	8.1E-03	0
105990532																												105990532	NLQNNAEWVYQGAIR	10	N-Term(iTRAQ4plex)	1919.98083	1.221	1.207	1.783	2.617	0.898	1.060	1.453	1.897	4.1	1.2	95.7	26.9	102.0	19.8	7.0	5.7	0.0E+00	1.6E-06	5.2	39	1.6E-02	5.9	71	1.0E-05	0
105990532																												105990532	GMALFGEGK	4	N-Term(iTRAQ4plex); K9(iTRAQ4plex)	1197.65508	1.119	1.026	1.041	1.965	0.999	0.988	0.945	1.864									0.0E+00	2.5E-03	3.1	31	7.2E-02	1.4	12	6.3E+00	0
105990532																												105990532	DNVFDGLVR	8	N-Term(iTRAQ4plex)	1178.62944	1.232	1.170	2.574	2.395	0.998	0.924	1.982	1.860		3.6		4.4		14.5		4.0	0.0E+00	1.7E-06	3.6	50	1.0E-03	3.7	52	7.1E-04	0
105990532																												105990532	AGHIAWTSSGK	4	N-Term(iTRAQ4plex); K11(iTRAQ4plex)	1402.76914	1.165	1.426	0.937	1.952	1.387	1.225	1.012	1.850									0.0E+00	1.5E-06	2.0	22	6.5E-01	3.9	40	1.2E-02	0
105990532																												105990532	EVGTVLSQVYSK	6	N-Term(iTRAQ4plex); K12(iTRAQ4plex)	1597.90519	1.299	1.286	2.439	2.354	1.033	0.989	1.906	1.842		14.9		11.7		23.5		3.4	0.0E+00	1.5E-06	3.7	34	3.7E-02	3.6	38	1.7E-02	0
105990532																												105990532	ETLEDTR	9	N-Term(iTRAQ4plex)	1007.51360	1.215	1.235	2.020	2.212	1.052	0.921	1.572	1.819	1.4	1.4	15.8	8.1	15.5	10.0	1.7	3.3	2.0E-03	9.5E-02	3.0	32	3.1E-02	2.7	28	6.1E-02	0
105990532																												105990532	VEDIPLAR	10	N-Term(iTRAQ4plex)	1056.61772	1.405	1.312	3.473	2.445	0.998	1.031	2.618	1.811	11.4	13.6	70.3	24.3	73.2	22.2	9.2	15.6	0.0E+00	2.3E-02	3.0	33	2.9E-02	3.2	34	1.9E-02	0
105990532																												105990532	VPQTDMTFR	11	N-Term(iTRAQ4plex)	1238.63396	1.047	1.163	1.412	2.169	0.993	0.861	1.198	1.804	6.4	10.9	9.7	1.4	20.0	2.1	3.1	14.5	0.0E+00	1.2E-02	2.6	41	7.2E-03	2.7	42	5.1E-03	0
105990532																												105990532	NNALDFVTK	2	N-Term(iTRAQ4plex); N2(Deamidated); K9(iTRAQ4plex)	1310.72197		1.173		2.323	0.924			1.782									0.0E+00	1.5E-06				3.6	66	3.2E-05	0
105990532																												105990532	LNGESNLR	6	N-Term(iTRAQ4plex); N2(Deamidated)	1047.55705	1.244	1.337	1.711	1.902	1.279	1.079	1.523	1.772	5.3		14.9		32.8		11.9		0.0E+00	3.5E-02	2.6	29	1.0E-01	3.1	27	1.9E-01	0
105990532																												105990532	NNALDFVTK	14	N-Term(iTRAQ4plex); K9(iTRAQ4plex)	1309.73674	1.492	1.212	4.504	2.050	1.021	1.098	3.404	1.765	10.2	13.2	41.8	18.3	32.9	2.3	2.0	7.1	0.0E+00	7.5E-05	4.1	72	1.1E-05	4.1	71	1.1E-05	0
105990532																												105990532	SGSSTASWIQNVDTK	9	N-Term(iTRAQ4plex); K15(iTRAQ4plex)	1868.96758	1.089	1.193	2.215	1.965	1.091	0.912	1.903	1.759		16.9		20.0		23.9		28.8	0.0E+00	1.2E-06	3.8	40	1.4E-02	5.7	51	1.1E-03	0
105990532																												105990532	TEHGSEMLFFGNAIEGK	2	N-Term(iTRAQ4plex); M7(Oxidation); K17(iTRAQ4plex)	2171.07016		1.373		1.827	1.356			1.757									0.0E+00	3.4E-07				5.7	34	5.1E-02	0
105990532																												105990532	GISTSAASPAVGTVGM DMDEDDDFSK	12	N-Term(iTRAQ4plex); M16(Oxidation); M18(Oxidation); K26(iTRAQ4plex)	2923.31046	1.473	1.696	2.034	2.058	1.281	1.296	1.838	1.736	27.3	25.4	59.6	18.4	141.8	15.6	23.3	47.6	0.0E+00	3.0E-07	5.6	63	2.4E-05	5.1	49	4.9E-04	0
105990532																												105990532	TEVIPPLIENR	14	N-Term(iTRAQ4plex)	1424.82524	1.187	1.220	2.197	2.251	1.011	0.967	1.756	1.704	2.7	8.5	18.7	5.0	16.0	37.2	2.3	3.3	0.0E+00	8.5E-06	3.8	49	1.7E-03	3.9	56	3.1E-04	0
105990532																												105990532	SVSLPSLDPASAK	10	N-Term(iTRAQ4plex); K13(iTRAQ4plex)	1559.88994	1.283	1.199	2.652	2.161	0.969	0.917	2.087	1.701	0.3	6.5	54.7	14.0	60.5	15.3	9.0	7.8	0.0E+00	1.5E-06	4.3	71	8.8E-06	3.9	61	9.1E-05	0
105990532																												105990532	IHSGSFQSQVELSNDQE K	4	N-Term(iTRAQ4plex); K18(iTRAQ4plex)	2321.15677		1.172		1.893	1.052			1.655		16.4		43.4		42.6		17.1	0.0E+00	5.4E-07				7.3	37	2.7E-02	0
105990532																												105990532	QHIEAIDVR	4	N-Term(iTRAQ4plex)	1224.68177	1.174	1.018	1.213	1.856	0.923	0.967	1.025	1.639									0.0E+00	1.5E-06	2.9	31	1.1E-01	3.0	41	1.1E-02	0
105990532																												105990532	NTLELSNGVIVK	2	N-Term(iTRAQ4plex); N7(Deamidated); K12(iTRAQ4plex)	1575.92026		1.107		1.640	1.121			1.619									0.0E+00	1.5E-06				3.9	42	7.0E-03	0
105990532																												105990532	ALYWVNGQVPDGVSK	4	N-Term(iTRAQ4plex); N6(Deamidated); K15(iTRAQ4plex)	1922.03281	1.254	1.246	2.240	1.323	1.550	0.864	1.584	1.604									0.0E+00	1.5E-06	4.2	28	1.9E-01	3.7	32	7.5E-02	0
105990532																												105990532	AVSMPSFSILGSDVR	12	N-Term(iTRAQ4plex); M4(Oxidation)	1725.89702	0.942	1.162	2.184	1.934	0.983	0.534	1.272	1.603	23.0	2.5	92.5	47.4	10.9	37.0	75.0	9.1	0.0E+00	1.5E-06	2.5	53	5.1E-04	4.0	89	1.5E-07	0
105990532																												105990532	TLQELK	1	N-Term(iTRAQ4plex); K6(iTRAQ4plex)	1019.63646		1.105		1.962	0.899			1.554									9.0E-03	2.1E-01				2.3			0
105990532																												105990532	ANLFNK	2	N-Term(iTRAQ4plex); K6(iTRAQ4plex)	994.59685	1.615	1.127	3.488	1.936	0.925	1.098	2.434	1.547									5.0E-03	4.0E-02		43	4.7E-03		38	8.8E-03	0
105990532																												105990532	KYTYNYEAESSSGVPG TADSR	6	N-Term(iTRAQ4plex); K1(iTRAQ4plex)	2570.22580	1.344	1.005	1.772	1.427	1.108	0.965	1.306	1.531	9.9		79.5		136.6		23.1		0.0E+00	3.3E-08	6.9	61	6.9E-05	5.1	53	4.7E-04	1
105990532																												105990532	SLHMYANR	4	N-Term(iTRAQ4plex); M4(Oxidation)	1151.57544		1.132		1.748	1.018			1.531		1.6		2.6		0.5		0.5	0.0E+00	5.3E-03				2.2	21	6.7E-01	0
105990532																												105990532	LPYTIITTPPLK	10	N-Term(iTRAQ4plex); K12(iTRAQ4plex)	1645.02317	1.190	0.842	3.093	1.651	0.856	0.860	2.295	1.527	3.8	1.4	1.2	8.1	7.7	18.2	5.1	11.5	0.0E+00	4.9E-04	4.0	52	1.3E-04	3.3	25	7.5E-02	0

105990532																															105990532	GNVATEISTER	15	N-Term(iTRAQ4plex)	1320.69219	1.260	1.193	1.598	2.219	0.891	1.156	1.418	1.524	13.7	8.5	23.9	51.4	14.8	42.7	4.2	1.0	0.0E+00	1.3E-05	4.3	38	1.9E-02	4.2	60	1.0E-04	0
105990532																															105990532	SLHMYANR	6	N-Term(iTRAQ4plex)	1135.58118		0.999		1.486	1.005			1.520		1.8		6.6		57.1		4.6	0.0E+00	6.0E-03				3.0	21	5.8E-01	0
105990532																															105990532	EFQVPTFTIPK	8	N-Term(iTRAQ4plex); K11(iTRAQ4plex)	1594.90537	1.350	1.032	4.372	1.890	0.939	0.908	3.021	1.512		3.1		5.6		18.4		14.3	0.0E+00	5.2E-03	2.8	28	1.8E-01	2.5	16	2.1E+00	0
105990532																															105990532	YGMVAQVTQTLK	6	N-Term(iTRAQ4plex); M3(Oxidation); K12(iTRAQ4plex)	1642.91399		1.102		1.802	1.124			1.511		4.8		9.7		30.5		16.4	0.0E+00	1.5E-06				4.0	56	3.4E-04	0
105990532																															105990532	KGNVATEISTER	10	N-Term(iTRAQ4plex); K1(iTRAQ4plex)	1592.88242	1.131	1.221	1.187	1.320	1.425	0.919	0.953	1.501	3.3		6.0		12.4		9.0		0.0E+00	2.9E-05	5.2	42	8.0E-03	4.8	43	6.4E-03	1
105990532																															105990532	TFIEDVNK	4	N-Term(iTRAQ4plex); K8(iTRAQ4plex)	1253.69780	1.679	1.224	4.270	2.139	0.860	1.271	3.319	1.463									0.0E+00	1.4E-03	3.8	55	3.6E-04	2.3	29	1.6E-01	0
105990532																															105990532	VIGNMGQTMEQLTPELK	6	N-Term(iTRAQ4plex); M5(Oxidation); M9(Oxidation); K17(iTRAQ4plex)	2209.14658	1.165	1.055	1.640	1.661	0.942	0.836	1.210	1.443		24.4		9.7		24.8		9.4	0.0E+00	1.5E-06	3.8	30	1.4E-01	3.5	43	7.8E-03	0
105990532																															105990532	SQAIATK	20	N-Term(iTRAQ4plex); K7(iTRAQ4plex)	1006.61449	1.418	1.396	3.326	2.087	0.973	0.967	2.210	1.437	1.5	19.2	7.7	15.9	12.5	31.8	1.7	15.8	0.0E+00	1.4E-02	3.1	49	4.0E-04	3.3	46	1.4E-03	0
105990532																															105990532	ALVEQGFTVPEIK	11	N-Term(iTRAQ4plex); K13(iTRAQ4plex)	1718.99468	1.265	1.134	2.651	1.556	1.140	0.936	2.015	1.434	20.8	3.5	11.4	53.2	21.5	66.8	10.8	12.2	0.0E+00	1.5E-06	4.8	55	2.9E-04	4.5	56	2.1E-04	0
105990532																															105990532	VPQTDMTFR	5	N-Term(iTRAQ4plex); M6(Oxidation)	1254.62786	1.126	1.086	1.872	1.778	0.889	0.837	1.556	1.419	12.7		55.2		71.9		5.1		1.0E-03	3.0E-02		35	2.0E-02		30	6.6E-02	0
105990532																															105990532	GTYGLSCQR	2	N-Term(iTRAQ4plex); C7(Methylthio)	1174.54717		1.281		1.751	1.065			1.418									0.0E+00	1.8E-06				3.2	23	7.1E-02	0
105990532																															105990532	VLLDQLGTTISFER	2	N-Term(iTRAQ4plex)	1735.97392		1.194		1.851	0.922			1.392									0.0E+00	8.9E-06				3.4	32	9.3E-02	0
105990532																															105990532	VIGNMGQTMEQLTPELK	3	N-Term(iTRAQ4plex); M5(Oxidation); K17(iTRAQ4plex)	2193.15287	1.183	1.146	1.081	1.683	0.959	0.904	0.848	1.370									0.0E+00	3.4E-06	2.7			3.0	34	5.4E-02	0
105990532																															105990532	ATLYALSHAVNNYHK	2	N-Term(iTRAQ4plex); N11(Deamidated); K15(iTRAQ4plex)	1991.06521		1.151		1.390	1.138			1.338									0.0E+00	1.5E-06				4.7	32	9.4E-02	0
105990532																															105990532	LDVTTSIGR	8	N-Term(iTRAQ4plex)	1105.63469	1.055	1.260	2.162	1.335	1.253	0.816	1.718	1.293	31.8	5.3	40.9	18.1	52.6	16.8	21.1	6.6	0.0E+00	7.1E-03	3.2	37	2.4E-02	2.3	32	8.2E-02	0
105990532																															105990532	AVSMPSFSILGSDVR	1	N-Term(iTRAQ4plex)	1709.90935		1.822		1.463	1.615			1.263									4.0E-03	1.5E-01				1.5			0
105990532																															105990532	SPAFTDLHLR	10	N-Term(iTRAQ4plex)	1300.71439	1.121	1.279	1.685	1.239	1.302	1.004	1.473	1.228	23.3	19.8	5.1	18.5	12.8	3.6	17.4	35.3	0.0E+00	9.1E-03	4.6	64	4.4E-05	4.4	42	6.5E-03	0
105990532																															105990532	GISTSAASPAVGTVGMDMDEDDDFSK	2	N-Term(iTRAQ4plex); K26(iTRAQ4plex)	2891.31717		1.340		2.324	0.712			1.202									0.0E+00	2.4E-09				4.8	59	7.2E-05	0
105990532																															105990532	KLTISEQNIQR	4	N-Term(iTRAQ4plex); K1(iTRAQ4plex)	1617.95560	1.028	1.054	1.200	1.434	0.872	0.717	0.860	1.156									0.0E+00	4.7E-06	5.3	42	5.0E-03	4.6	23	3.8E-01	1
105990532																															105990532	ESQLPTVMDFR	12	N-Term(iTRAQ4plex)	1466.74309	1.361	1.233	2.016	1.762	1.140	0.918	1.357	1.135	8.9	6.3	6.1	7.8	6.6	68.3	5.5	5.9	0.0E+00	9.4E-04	3.7	48	1.3E-03	3.7	39	1.2E-02	0
105990532																															105990532	TQFNNNEYSQDLDAYNTK	12	N-Term(iTRAQ4plex); K18(iTRAQ4plex)	2453.14725	1.319	0.956	2.306	1.273	0.852	1.235	2.217	1.115	32.7	13.8	18.6	24.7	37.5	18.9	14.1	3.2	0.0E+00	4.8E-08	6.3	72	5.4E-06	3.9	36	2.1E-02	0
105990532																															105990532	TQFNNNEYSQDLDAYNTK	6	N-Term(iTRAQ4plex); Q10(Deamidated); K18(iTRAQ4plex)	2454.15067		1.065		0.902	1.254			1.034		29.8		28.9		19.5		26.9	0.0E+00	4.7E-02				3.2	48	9.9E-04	0
105990532																															105990532	VQGVEFSHR	6	N-Term(iTRAQ4plex)	1202.63999	0.906	1.024	1.123	0.867	1.209	0.939	1.196	0.997	2.8		9.7		9.6		2.9		0.0E+00	4.1E-02	3.5	33	4.2E-02	3.2	26	2.1E-01	0
105990532																															105990532	SVSDGIAALDLNAVANK	2	N-Term(iTRAQ4plex); K17(iTRAQ4plex)	1946.08866		0.751		0.919	0.832			0.992									0.0E+00	1.5E-06				3.6	37	2.9E-02	0
105990532																															105990532	GISTSAASPAVGTVGMDMDEDDDFSK	6	N-Term(iTRAQ4plex); M16(Oxidation); K26(iTRAQ4plex)	2907.31968		1.267		1.549	0.809			0.963		9.7		38.4		8.2		40.0	0.0E+00	1.5E-06	2.9	21	4.2E-01	4.1	35	2.0E-02	0
105990532																															105990532	MGLAFESTK	6	N-Term(iTRAQ4plex); K9(iTRAQ4plex)	1271.69182	0.952	1.037	1.136	1.041	0.902	0.680	0.832	0.882		46.9		15.6		13.6		49.1	0.0E+00	3.1E-03	3.2	39	1.1E-02	3.2	40	9.8E-03	0
105990532																															105990532	MGLAFESTK	7	N-Term(iTRAQ4plex); M1(Oxidation); K9(iTRAQ4plex)	1287.68620	1.203	0.840	2.957	1.121	0.661	0.874	2.206	0.859	10.7	88.2	34.1	253.3	36.2	278.2	8.8	79.8	0.0E+00	2.1E-05	2.9	60	9.4E-05	2.9	54	3.7E-04	0
105990532																															105990532	YTYNYEAESSSGVPGTADSR	8	N-Term(iTRAQ4plex)	2298.03545	1.219	1.104	6.116	1.366	0.672	1.249	6.436	0.810		17.5		46.7		41.5		75.1	0.0E+00	4.4E-05	4.8	43	1.6E-03	2.7	21	3.2E-01	0
105990532																															105990532	VNWEEEAASGLLTSLK	3	N-Term(iTRAQ4plex); K16(iTRAQ4plex)	2035.09732		1.262		1.296	0.697			0.696									0.0E+00	1.5E-06				3.0	22	9.2E-01	0
105990532																															105990532	LDNIYSSDK	2	N-Term(iTRAQ4plex); K9(iTRAQ4plex)	1342.70891	1.870		6.598			1.729	6.262										0.0E+00	1.3E-03	3.6	51	9.0E-04				0
105990532																															105990532	IAELSATAQEIIK	2	N-Term(iTRAQ4plex); K13(iTRAQ4plex)	1674.98801	1.440		8.233			0.919	5.393										0.0E+00	4.1E-05	4.3	36	1.5E-02				0
105990532																															105990532	VIGTLK	1	N-Term(iTRAQ4plex); K6(iTRAQ4plex)	918.62334	1.566		5.019			1.506	4.954										9.0E-03	1.1E-01		43	7.9E-04				0
105990532																															105990532	INPLALK	2	N-Term(iTRAQ4plex); K7(iTRAQ4plex)	1056.70207	1.765		4.505			1.531	4.012										3.0E-03	4.9E-02	3.0	34	2.1E-03				0
105990532																															105990532	IDDIWNLEVK	2	N-Term(iTRAQ4plex); K10(iTRAQ4plex)	1532.85918	1.539		4.097			1.328	3.629										0.0E+00	2.8E-04	4.0	40	1.1E-02				0
105990532																															105990532	NLLVALK	2	N-Term(iTRAQ4plex); K7(iTRAQ4plex)	1058.71868	1.395		3.124			1.477	3.394										0.0E+00	4.9E-03	3.1	62	8.8E-06				0
105990532																															105990532	TGISPLALIK	2	N-Term(iTRAQ4plex); K10(iTRAQ4plex)	1300.84723	0.897		3.677			0.794	3.341										0.0E+00	4.8E-03	2.7	23	6.3E-02				0
105990532																															105990532	QSWSVCK	2	N-Term(iTRAQ4plex); C6(Methylthio); K7(iTRAQ4plex)	1171.58660	1.273		4.167			0.940	3.160										4.0E-03	6.0E-02	3.0	35	1.4E-02				0

105990532																															105990532	LFLEETK	4	N-Term(iTRAQ4plex); K7(iTRAQ4plex)	1167.68767	1.422		3.660			1.030	2.722		10.1		30.4		32.9		7.7		1.0E-03	3.0E-02	2.9	45	2.3E-03				0
105990532																															105990532	VLVDHFGYTK	2	N-Term(iTRAQ4plex); K10(iTRAQ4plex)	1466.82575	1.304		3.181			0.979	2.452										0.0E+00	5.1E-05	4.0	51	9.0E-04				0
105990532																															105990532	NPNGYSFSIPVK	4	N-Term(iTRAQ4plex); N3(Deamidated); K12(iTRAQ4plex)	1611.86150	1.435		3.166			0.990	2.244		6.7		17.8		15.3		9.1		0.0E+00	2.7E-05	4.8	67	3.0E-05				0
105990532																															105990532	SEILAHWSPAK	2	N-Term(iTRAQ4plex); K11(iTRAQ4plex)	1526.85953	1.192		1.976			1.259	2.142										0.0E+00	2.2E-04	3.4	33	4.4E-02				0
105990532																															105990532	MTSNFPVDLSDYPK	4	N-Term(iTRAQ4plex); M1(Oxidation); K14(iTRAQ4plex)	1917.94864	1.287		2.349			1.127	2.111		7.5		62.7		63.1		7.2		0.0E+00	4.9E-04	3.4	34	4.7E-02				0
105990532																															105990532	HINIDQFVR	2	N-Term(iTRAQ4plex)	1285.71430	1.008		2.430			0.817	2.021										0.0E+00	3.8E-04	3.3	40	1.0E-02				0
105990532																															105990532	SVGFHLPSR	4	N-Term(iTRAQ4plex)	1143.63755	1.216		2.320			1.020	1.999		8.1		17.1		17.6		7.6		0.0E+00	1.2E-03	3.1	40	7.6E-03				0
105990532																															105990532	IVQILPWEQNEQVK	3	N-Term(iTRAQ4plex); K14(iTRAQ4plex)	2012.14414	1.338		2.475			0.999	1.897		11.7		40.1		43.2		8.9		0.0E+00	4.7E-05	3.0	32	5.1E-02				0
105990532																															105990532	YEDGTLSLTSTSDLQSGIIK	2	N-Term(iTRAQ4plex); Q15(Deamidated); K20(iTRAQ4plex)	2417.27597	1.759		2.199			1.470	1.887										0.0E+00	1.2E-05	5.2	30	1.2E-01				0
105990532																															105990532	WNFYYSPQSSPDK	4	N-Term(iTRAQ4plex); K13(iTRAQ4plex)	1906.92241	1.448		2.113			1.197	1.794		4.1		26.0		11.1		10.5		0.0E+00	1.1E-04	3.7	29	1.1E-01				0
105990532																															105990532	FIIPSPK	2	N-Term(iTRAQ4plex); K7(iTRAQ4plex)	1089.69109	1.392		2.172			1.111	1.781										6.0E-03	8.2E-02	1.9	13	3.5E+00				0
105990532																															105990532	EVYGFNPEGK	4	N-Term(iTRAQ4plex); K10(iTRAQ4plex)	1427.74175	1.337		2.295			1.008	1.776		0.9		9.6		8.0		0.6		0.0E+00	7.6E-05	4.2	50	9.0E-04				0
105990532																															105990532	VAWHYDEEK	1	N-Term(iTRAQ4plex); K9(iTRAQ4plex)	1464.73853	0.779		1.394			0.942	1.732										7.0E-03	9.9E-02	2.3						0
105990532																															105990532	SNTVASLHTEK	2	N-Term(iTRAQ4plex); K11(iTRAQ4plex)	1474.80970	1.040		1.745			0.970	1.670										1.0E-03	2.4E-02	3.8	17	1.8E+00				0
105990532																															105990532	ITENDIQIALDDAK	2	N-Term(iTRAQ4plex); K14(iTRAQ4plex)	1847.00090	0.965		1.372			0.999	1.458										0.0E+00	7.2E-03	2.4	40	1.5E-02				0
105990532																															105990532	YGMVAQVTQTLK	2	N-Term(iTRAQ4plex); K12(iTRAQ4plex)	1626.91806	1.417		1.718			1.141	1.421										0.0E+00	9.2E-05	3.7	34	4.4E-02				0
105990532																															105990532	SVSDGIAALDLNAVANK	2	N-Term(iTRAQ4plex); N12(Deamidated); K17(iTRAQ4plex)	1947.05619	1.094		1.486			0.882	1.230										0.0E+00	9.3E-04	3.8	29	1.6E-01				0
105990532																															105990532	YEGLQEWEGK	2	N-Term(iTRAQ4plex); K10(iTRAQ4plex)	1526.77239	1.200		1.286			1.093	1.203										0.0E+00	8.4E-03	3.5	12	6.1E+00				0
105990532																															105990532	DFSAEYEEDGKYEGLQEWEGK	2	N-Term(iTRAQ4plex); K11(iTRAQ4plex); K21(iTRAQ4plex)	2941.38564	0.965		1.933			0.564	1.159										0.0E+00	2.9E-04	4.5	22	5.7E-01				1
105990532																															105990532	YEVDQQIQVLMDK	2	N-Term(iTRAQ4plex); M11(Oxidation); K13(iTRAQ4plex)	1913.00553	0.988		1.145			0.924	1.100										0.0E+00	9.0E-04	2.1	24	5.7E-01				0
105990532																															105990532	LALWGEHTGQLYSK	2	N-Term(iTRAQ4plex); K14(iTRAQ4plex)	1891.03269	1.286		0.850			1.427	0.969										0.0E+00	2.2E-03	3.2	21	1.2E+00				0
105990532																															105990532	YNALDLTNNGK	1	N-Term(iTRAQ4plex); N9(Deamidated); K11(iTRAQ4plex)	1511.79544	0.789		0.926			0.765	0.921										1.0E-03	1.9E-02	2.5						0
105990532																															105990532	NLTDFAEQYSIQDWAK	2	N-Term(iTRAQ4plex); Q8(Deamidated); K16(iTRAQ4plex)	2218.10685	1.174		0.899			0.818	0.643										0.0E+00	3.5E-04	3.8	31	1.2E-01				0
116292750	NP_005568.2	LPA	4018	apolipoprotein(a) precursor	GBC IVB/Healthy	IVB specific	8	51	0.609	0.617	5.1E-13	1.850	**2.151**	2.1E-12	**0.398**	**0.438**	3.3E-20	1.476	1.917	3.2E-01	57.8	84.6	14.3	19.8	24.0	20.1	53.4	44.5	226.4	6.07	116292750																													
116292750																															116292750	GTYSTTVTGR	16	N-Term(iTRAQ4plex)	1186.62200	0.610	0.604	4.543	4.967	0.395	0.372	2.827	3.288	6.0	7.1	67.1	16.8	63.3	26.1	5.9	12.9	0.0E+00	1.9E-06	3.8	52	5.2E-04	3.7	40	1.0E-02	0
116292750																															116292750	GSFSTTVTGR	10	N-Term(iTRAQ4plex)	1156.61113	0.554	0.656	1.488	3.460	0.417	0.461	1.162	2.144	6.0	29.3	21.2	4.9	32.0	6.7	19.4	17.3	0.0E+00	1.5E-06	3.7	46	1.8E-03	3.9	53	3.9E-04	0
116292750																															116292750	NPDAVAAPYCYTR	4	N-Term(iTRAQ4plex); C10(Methylthio)	1630.74932	0.678	0.432	1.596	2.151	0.395	0.326	0.787	1.917									0.0E+00	1.5E-06	4.1	33	1.8E-02	4.8	41	2.5E-03	0
116292750																															116292750	GTLSTTITGR	4	N-Term(iTRAQ4plex)	1150.65337		0.538		1.881	0.434			1.478		11.7		2.2		15.3		1.3	0.0E+00	1.5E-06				3.8	45	3.5E-03	0
116292750																															116292750	GISSTTVTGR	4	N-Term(iTRAQ4plex)	1122.62554	0.687	0.656	1.634	1.573	0.558	0.583	1.424	1.303									0.0E+00	5.6E-03	3.8	43	5.0E-03	3.6	43	4.7E-03	0
116292750																															116292750	GTFSTTVTGR	7	N-Term(iTRAQ4plex)	1170.62358	0.595	0.741	1.185	1.204	0.745	0.500	1.022	1.178	8.1	20.8	54.4	30.3	37.9	12.6	6.2	3.5	0.0E+00	1.5E-06	3.5	44	3.5E-03	2.5	46	1.7E-03	0
116292750																															116292750	TTEYYPNGGLTR	5	N-Term(iTRAQ4plex); N7(Deamidated)	1516.74077	0.528	0.746	2.774	1.491	0.504	0.322	1.737	0.981	16.0		104.7		132.8		31.4		0.0E+00	2.7E-03	3.6	27	1.2E-01	2.3			0
116292750																															116292750	TPENYPNAGLTR	1	N-Term(iTRAQ4plex)	1476.75957	0.699		2.591			0.397	1.512										9.0E-03	1.3E-01	2.6						0
116734717	NP_000469.3	ALPL	249	alkaline phosphatase, tissue-nonspecific isozyme isoform 1 preproprotein	GBC IIIA/GSD; GBC IVB vs GSD	IIIA and IVB	5	10	1.500	**3.324**	6.7E-05	**1.951**	**2.292**	1.3E-04	1.830	**3.463**	1.8E-03	**2.444**	**2.213**	4.4E-03		66.5		46.6		50.0		91.9	57.3	6.67	116734717																													
116734717																															116734717	TDVEYESDEK	1	N-Term(iTRAQ4plex); K10(iTRAQ4plex)	1502.71086		4.468		3.686	5.205			4.182									1.0E-03	6.4E-02				2.6			0
116734717																															116734717	DQAQETLK	2	N-Term(iTRAQ4plex); K8(iTRAQ4plex)	1220.66997		4.018		3.226	4.302			3.365									0.0E+00	3.6E-04				3.5	33	8.8E-02	0
116734717																															116734717	SVGIVTTTR	2	N-Term(iTRAQ4plex)	1077.63774		2.751		2.356	2.788			2.325									0.0E+00	1.4E-05				3.2	30	8.3E-02	0
116734717																															116734717	ANEGTVGVSAATER	3	N-Term(iTRAQ4plex)	1505.76775		2.453		1.671	2.321			1.540		111.4		49.1		44.8		118.1	0.0E+00	2.2E-03				3.1	40	9.3E-03	0

116734717																															116734717	DIDVIMGGGR	1	N-Term(iTRAQ4plex); M6(Oxidation)	1192.60918		1.037		0.948	1.213			1.080									3.0E-03	2.3E-02					35	1.9E-02	0
116734717																															116734717	DIDVIMGGGR	1	N-Term(iTRAQ4plex)	1176.61528	1.500		1.951			1.830	2.444										4.0E-03	6.7E-02	1.5						0
156627571	NP_003095.2	SORD	6652	sorbitol dehydrogenase	GBC IIIA/Healthy; GBC IVB vs Healthy	IIIA and IVB	5	9	**3.827**	**2.953**	4.2E-05	0.939	1.123	2.6E-01	**3.597**	**2.171**	5.2E-03	0.906	0.912	2.5E-03		24.8		54.2		57.9		5.2	38.3	7.97	156627571																													
156627571																															156627571	ALEAFETFK	2	N-Term(iTRAQ4plex); K9(iTRAQ4plex)	1343.74578		3.366		1.095	3.049			0.966									0.0E+00	2.1E-06				3.9	41	8.0E-03	0
156627571																															156627571	LENYPIPEPGPNEVLLR	2	N-Term(iTRAQ4plex)	2094.13823		5.135		1.153	4.224			0.923									0.0E+00	1.5E-06				3.4	29	1.6E-01	0
156627571																															156627571	ENDEFCK	1	N-Term(iTRAQ4plex); C6(Methylthio); K7(iTRAQ4plex)	1218.53581		2.591		1.622	1.476			0.900									2.0E-03	1.1E-01				2.3			0
156627571																															156627571	ESPQEIAR	2	N-Term(iTRAQ4plex)	1073.56963		1.689		0.829	1.546			0.739									3.0E-03	2.3E-02				2.7	30	4.3E-02	0
156627571																															156627571	VAIEPGAPR	2	N-Term(iTRAQ4plex)	1053.61736	3.827		0.939			3.597	0.906										0.0E+00	3.2E-03	2.6	37	1.2E-02				0
157266300	NP_001141.2	ANPEP	290	aminopeptidase N precursor	GBC IIIA/Healthy, GSD; GBC IVB/Healthy, GSD	IIIA and IVB	23	132	**3.938**	**4.561**	1.2E-79	**2.840**	**3.783**	3.4E-68	**3.492**	**4.399**	4.1E-63	**2.811**	**3.775**	1.9E-40	51.3	41.7	50.5	41.9	55.3	38.9	47.9	43.8	109.5	5.48	157266300																													
157266300																															157266300	SIQLPTTVR	3	N-Term(iTRAQ4plex)	1158.69695	3.517	6.186	2.808	6.290	5.832	3.509	2.877	5.775	20.4		1.2		10.0		11.5		0.0E+00	1.6E-03	3.3			3.2			0
157266300																															157266300	GFYISK	4	N-Term(iTRAQ4plex); K6(iTRAQ4plex)	1002.58592	4.439	6.620	3.620	6.038	5.662	3.613	3.025	5.030		3.4		19.8		17.4		1.0	6.0E-03	5.7E-02		42	4.9E-03	2.5	39	1.1E-02	0
157266300																															157266300	VNYDEENWR	6	N-Term(iTRAQ4plex)	1368.63030	3.532	6.218	3.502	5.558	5.621	2.927	2.980	4.893	5.3		42.5		41.3		6.4		0.0E+00	1.5E-06	3.8	42	1.8E-03	4.3	51	2.4E-04	0
157266300																															157266300	SFPCFDEPAMK	4	N-Term(iTRAQ4plex); C4(Methylthio); K11(iTRAQ4plex)	1605.73821	8.033	7.776	6.144	5.064	7.235	7.145	5.611	4.589									0.0E+00	2.2E-05	3.7	50	2.4E-04	3.8	49	3.4E-04	0
157266300																															157266300	SFPCFDEPAMK	11	N-Term(iTRAQ4plex); C4(Methylthio); M10(Oxidation); K11(iTRAQ4plex)	1621.72954	2.937	4.832	2.708	4.042	5.241	2.858	2.705	4.270	24.0	43.2	18.5	17.3	22.0	23.3	23.3	36.7	0.0E+00	8.3E-03	3.2	67	4.5E-06	3.0	28	3.7E-02	0
157266300																															157266300	IQTQLQR	5	N-Term(iTRAQ4plex)	1030.61321	4.464	4.807	5.128	4.499	4.537	4.457	5.257	4.188	11.3	28.4	38.7	0.9	45.0	2.7	5.7	30.3	2.0E-03	4.4E-02	3.0			3.0			0
157266300																															157266300	SEYMEGNVR	1	N-Term(iTRAQ4plex); M4(Oxidation)	1244.56987		4.251		3.796	4.695			4.083									5.0E-03	4.6E-02					35	6.0E-03	0
157266300																															157266300	SEYMEGNVR	10	N-Term(iTRAQ4plex)	1228.57561	3.605	4.319	3.175	4.007	4.265	3.347	3.027	3.854	1.6	108.1	14.0	46.7	3.7	37.5	11.9	123.2	0.0E+00	6.9E-04	3.6	38	4.9E-03	3.6	43	1.5E-03	0
157266300																															157266300	DNEETGFGSGTR	16	N-Term(iTRAQ4plex)	1413.63725	7.918	5.827	2.070	4.267	5.295	7.636	2.078	3.776	28.6	16.2	54.8	27.8	57.4	33.8	30.7	32.3	0.0E+00	1.5E-06	4.6	67	5.0E-06	4.7	68	4.0E-06	0
157266300																															157266300	ENSLLFDPLSSSSSNK	8	N-Term(iTRAQ4plex); K16(iTRAQ4plex)	2013.03862	4.807	5.243	4.082	3.704	5.380	4.083	3.561	3.702		17.4		12.9		7.2		23.2	0.0E+00	3.2E-08	4.0	56	4.0E-04	5.0	57	2.8E-04	0
157266300																															157266300	SEVYGPMK	8	N-Term(iTRAQ4plex); K8(iTRAQ4plex)	1198.63762	2.247	3.361	2.140	3.217	3.474	2.024	1.980	3.238	35.6	50.3	6.5	76.7	10.5	64.0	31.4	61.8	0.0E+00	5.0E-03	3.1	38	1.3E-02	2.8	35	2.9E-02	0
157266300																															157266300	GVGGSQPPDIDK	10	N-Term(iTRAQ4plex); K12(iTRAQ4plex)	1457.78044	1.617	3.197	1.333	3.314	2.703	1.793	1.518	2.780	0.6	11.0	8.0	43.6	3.2	46.7	4.2	22.3	0.0E+00	4.8E-04	3.1	28	1.6E-01	3.8	57	2.4E-04	0
157266300																															157266300	VVATTQMQAADAR	7	N-Term(iTRAQ4plex); M7(Oxidation)	1521.77983	5.385	4.491	4.121	2.988	3.911	6.685	5.253	2.780	92.6	7.7	270.7	13.8	203.7	16.4	123.6	4.4	1.0E-03	1.1E-02		25	3.2E-01	1.5	54	4.2E-04	0
157266300																															157266300	DHSAIPVINR	2	N-Term(iTRAQ4plex)	1265.70940		2.321		2.427	2.561			2.608									0.0E+00	1.5E-06				3.7	36	3.7E-02	0
157266300																															157266300	LPNTLKPDSYR	4	N-Term(iTRAQ4plex); K6(iTRAQ4plex)	1591.90573		3.199		3.267	2.382			2.369		73.7		81.9		116.4		47.3	0.0E+00	2.4E-06				3.4	29	1.2E-01	0
157266300																															157266300	QASNGVLIR	2	N-Term(iTRAQ4plex); N4(Deamidated)	1102.63335	3.938	3.424	3.408	2.229	3.214	2.541	2.258	2.038									1.0E-03	3.3E-02	3.6			2.5			0
157266300																															157266300	KDNEETGFGSGTR	6	N-Term(iTRAQ4plex); K1(iTRAQ4plex)	1685.82950	2.100	2.723	1.387	1.898	2.875	1.781	1.208	1.952	26.6		19.1		5.0		41.7		0.0E+00	2.5E-03	4.0	33	4.4E-02	4.0	29	1.1E-01	1
157266300																															157266300	SEVYGPMK	2	N-Term(iTRAQ4plex); M7(Oxidation); K8(iTRAQ4plex)	1214.63188		4.141		2.211	3.687			1.917									2.0E-03	2.0E-02				1.9	33	3.1E-02	0
157266300																															157266300	VVATTQMQAADAR	2	N-Term(iTRAQ4plex)	1505.79143	5.404		6.017			5.361	6.128										0.0E+00	3.9E-03	2.5	30	1.3E-01				0
157266300																															157266300	ELWILNR	3	N-Term(iTRAQ4plex)	1087.63725	4.666		3.869			4.665	3.972		54.0		54.8		60.0		49.0		1.0E-03	3.4E-02	2.8	27	1.7E-01				0
157266300																															157266300	ALEQALEK	4	N-Term(iTRAQ4plex); K8(iTRAQ4plex)	1189.70207	5.072		3.455			5.393	3.771		17.7		34.5		39.8		12.8		0.0E+00	7.2E-03	4.1	51	4.6E-04				0
157266300																															157266300	DLMVLNDVYR	2	N-Term(iTRAQ4plex); M3(Oxidation)	1397.72148	4.597		3.538			4.680	3.699										0.0E+00	1.8E-03	3.1	40	9.4E-03				0
157266300																															157266300	GLYVFK	2	N-Term(iTRAQ4plex); K6(iTRAQ4plex)	1014.62212	5.187		3.495			4.689	3.245		18.0		1.3		0.5		17.2		8.0E-03	1.0E-01		24	2.1E-01				0
157266300																															157266300	DLTALSNMLPK	2	N-Term(iTRAQ4plex); M8(Oxidation); K11(iTRAQ4plex)	1506.84391	3.251		2.840			3.257	2.922										0.0E+00	2.1E-04	3.3	35	3.5E-02				0
157266300																															157266300	QQQDYWLIDVR	2	N-Term(iTRAQ4plex)	1607.82573	7.081		4.785			3.443	2.389										0.0E+00	5.4E-05	4.6	46	2.5E-03				0
157266300																															157266300	AALACSK	2	N-Term(iTRAQ4plex); C5(Methylthio); K7(iTRAQ4plex)	997.54271	2.535		2.780			1.838	2.070										1.0E-03	2.3E-02	3.0	38	6.9E-03				0
157266300																															157266300	DLMVLNDVYR	2	N-Term(iTRAQ4plex); N6(Deamidated)	1382.71001	1.453		1.821			1.423	1.831										0.0E+00	3.0E-04	3.0	39	1.0E-02				0
157266300																															157266300	QWMENPNNNPIHPNLR	2	N-Term(iTRAQ4plex); M3(Oxidation)	2134.03305	1.692		1.317			1.567	1.252										0.0E+00	1.0E-04	3.5	31	7.7E-02				0

157266337	NP_001704.2	BHMT	635	betaine--homocysteine S-methyltransferase 1	GBC IIIA/Healthy; GBC IVB vs GSD	IIIA and IVB	3	11	**7.272**	**3.818**	1.3E-05	1.549	1.371	6.2E-03	**2.283**	1.604	4.6E-01	**0.499**	**0.537**	9.1E-08	33.3	2.3	225.5	13.8	127.8	4.2	3.6	7.3	45.0	7.03	157266337																													
157266337																															157266337	EAYNLGVR	4	N-Term(iTRAQ4plex)	1065.57964	2.937	3.818	1.244	1.392	1.650	1.176	0.511	0.586									0.0E+00	1.1E-02	2.9	45	4.7E-03	3.1	35	4.3E-02	0
157266337																															157266337	AIAEELAPER	5	N-Term(iTRAQ4plex)	1242.68071		3.283		1.336	1.321			0.524		37.3		3.6		3.8		29.3	0.0E+00	4.1E-03	3.6			4.1	48	1.6E-03	0
157266337																															157266337	ISGQEVNEAACDIAR	2	N-Term(iTRAQ4plex); C11(Methylthio)	1765.83171	18.001		1.928			4.434	0.488										0.0E+00	1.9E-05	4.5	71	5.0E-06				0
183076548	NP_001116847.1	HIST2H3D	653604	histone H3.2	GBC IIIA/Healthy	IIIA specific	3	12	**2.010**	**3.521**	2.7E-05	1.410	**2.049**	9.7E-04	1.321	**2.137**	5.8E-02	1.013	1.099	5.2E-03	30.8	22.4	30.5	50.1	18.3	33.8	14.3	18.7	15.4	11.27	183076548																													
183076548																															183076548	EIAQDFK	5	N-Term(iTRAQ4plex); K7(iTRAQ4plex)	1138.63371	1.842	4.083	1.179	2.030	2.569	1.627	1.069	1.244		78.5		18.6		23.9		27.8	0.0E+00	4.6E-02	2.4			3.0	33	4.4E-02	0
183076548																															183076548	STELLIR	5	N-Term(iTRAQ4plex)	975.59685	2.458	3.521	1.834	2.049	1.711	1.321	1.012	0.970	17.0		20.6		12.6		9.1		3.0E-03	1.2E-01	3.2	25	9.5E-02	3.0	30	2.8E-02	0
183076548																															183076548	YRPGTVALR	2	N-Term(iTRAQ4plex)	1176.69806	1.322		1.122			1.100	0.959										0.0E+00	1.2E-03	3.6	32	6.6E-02				0
188497671	NP_110381.2	SAA2	6289	serum amyloid A-2 protein isoform a precursor	GBC IVB/Healthy	IVB specific	2	9	1.536	1.393	3.6E-02	1.058	1.872	3.3E-01	**2.590**	**2.015**	4.9E-02	1.740	**2.637**	1.7E-01	36.3	59.4	7.6	39.6	5.6	98.1	51.4	128.9	13.5	9.14	188497671																													
188497671																															188497671	GAEDSLADQAANK	7	N-Term(iTRAQ4plex); K13(iTRAQ4plex)	1577.80149	1.536	1.802	1.058	2.712	3.506	2.590	1.740	5.140	7.6		51.4		36.3		5.6		0.0E+00	1.5E-06	3.9	34	5.0E-02	5.0	37	2.2E-02	0
188497671																															188497671	GPGGAWAAEVISNAR	2	N-Term(iTRAQ4plex)	1599.83658		1.077		1.292	1.158			1.353									0.0E+00	1.5E-06				4.9	50	1.2E-03	0
221316614	NP_004416.2	ECM1	1893	extracellular matrix protein 1 isoform 1 precursor	GBC IVB/Healthy	IVB specific	11	58	1.333	1.226	8.7E-13	1.046	1.093	7.0E-01	**2.035**	**1.993**	1.9E-16	1.584	1.523	2.2E-03	19.5	11.5	12.7	19.3	18.4	20.2	24.8	12.1	60.6	6.71	221316614																													
221316614																															221316614	LLPAQLPAEK	12	N-Term(iTRAQ4plex); K10(iTRAQ4plex)	1367.84990	1.530	1.340	1.136	0.995	2.298	2.314	1.764	1.693	9.1	10.8	10.2	2.8	7.0	1.1	12.3	9.0	0.0E+00	8.5E-05	3.6	55	1.8E-04	3.5	49	7.3E-04	0
221316614																															221316614	DECFAR	1	N-Term(iTRAQ4plex); C3(Methylthio)	930.39379		1.312		1.113	2.046			1.690									8.0E-03	7.2E-02					20	5.8E-02	0
221316614																															221316614	AWEDTLDK	8	N-Term(iTRAQ4plex); K8(iTRAQ4plex)	1265.65972	1.470	0.993	0.936	1.129	1.442	2.210	1.444	1.596	4.5	32.1	3.7	20.0	2.8	3.1	5.4	50.8	0.0E+00	9.0E-04	3.6	65	3.1E-05	3.4	47	1.9E-03	0
221316614																															221316614	DILTIDIGR	4	N-Term(iTRAQ4plex)	1159.68022	1.120	1.051	0.562	0.794	2.069	1.792	0.924	1.523									0.0E+00	7.0E-06	1.9	24	2.3E-01	3.1	41	4.3E-03	0
221316614																															221316614	NVALVSGDTENAK	2	N-Term(iTRAQ4plex); N11(Deamidated); K13(iTRAQ4plex)	1606.85417		1.410		1.203	1.793			1.489									0.0E+00	4.9E-06				2.8	50	1.5E-03	0
221316614																															221316614	NLPATDPLQR	7	N-Term(iTRAQ4plex)	1268.70903	1.268	1.224	1.201	1.083	1.704	1.938	1.885	1.469	3.3	4.1	23.4	7.9	19.9	12.8	0.1	25.1	0.0E+00	1.4E-03	2.9	26	3.2E-01	3.3	42	7.8E-03	0
221316614																															221316614	QGETLNFLEIGYSR	2	N-Term(iTRAQ4plex)	1770.91362		1.123		1.025	1.651			1.468									0.0E+00	1.5E-06				4.1	42	8.7E-03	0
221316614																															221316614	ELPSLQHPNEQK	4	N-Term(iTRAQ4plex); K12(iTRAQ4plex)	1707.93143	1.198	1.169	1.041	1.034	1.694	1.341	1.196	1.459									0.0E+00	3.7E-04	4.1	32	9.5E-02	3.8	22	8.3E-01	0
221316614																															221316614	EVGPPLPQEAVPLQK	9	N-Term(iTRAQ4plex); K15(iTRAQ4plex)	1890.09464	1.435	1.513	1.023	1.121	1.969	2.113	1.546	1.443	22.6	20.6	42.2	4.0	34.1	3.2	15.1	22.0	0.0E+00	2.5E-05	3.5	41	6.5E-03	3.5	28	1.2E-01	0
221316614																															221316614	LVWEEAMSR	3	N-Term(iTRAQ4plex); M7(Oxidation)	1280.64592	1.213	1.024	0.979	0.956	1.156	1.539	1.276	1.052									3.0E-03	4.7E-02	2.2	22	5.0E-01		26	1.6E-01	0
221316614																															221316614	LVWEEAMSR	4	N-Term(iTRAQ4plex)	1264.64824	1.477		1.184			2.237	1.842		13.6		0.4		6.9		21.1		0.0E+00	1.1E-03	3.2	30	7.4E-02				0
221316614																															221316614	LTFINDLCGPR	2	N-Term(iTRAQ4plex); N5(Deamidated); C8(Methylthio)	1439.70830	1.318		0.628			3.174	1.554										0.0E+00	8.0E-03	2.5	25	2.0E-01				0
316659409	NP_001186883.1	ACTG1	71	actin, cytoplasmic 2	GBC IIIA/Healthy; GBC IVB vs Healthy	IIIA and IVB	4	24	**0.428**	**0.462**	9.6E-06	0.731	0.891	4.1E-02	**0.441**	**0.420**	5.9E-07	0.692	0.980	4.2E-05	16.9	18.9	25.0	57.7	40.5	53.0	34.3	14.1	41.8	5.48	316659409																													
316659409																															316659409	QEYDESGPSIVHR	2	N-Term(iTRAQ4plex)	1660.80375		1.330		1.113	1.326			1.081									0.0E+00	1.5E-06				3.7	33	4.0E-02	0
316659409																															316659409	GYSFTTTAER	12	N-Term(iTRAQ4plex)	1276.63152	0.401	0.266	0.660	0.891	0.300	0.359	0.582	0.980	20.9	17.2	8.3	0.1	9.3	16.2	22.0	1.1	0.0E+00	1.4E-03	4.0	43	2.5E-03	4.0	51	3.7E-04	0
316659409																															316659409	VAPEEHPVLLTEAPLNPK	9	N-Term(iTRAQ4plex); K18(iTRAQ4plex)	2242.26999	0.496	0.501	0.774	0.787	0.490	0.555	0.890	0.894	22.0	24.3	3.9	2.4	8.6	0.9	35.2	48.3	0.0E+00	1.5E-06	4.0	12	3.9E+00	4.2	33	3.4E-02	0
316659409																															316659409	DLYANTVLSGGTTMYPGIADR	1	N-Term(iTRAQ4plex)	2359.17752	1.038		1.311			0.869	1.128										4.0E-03	5.7E-02	1.4						0
325651886	NP_001191742.1	NT5E	4907	5'-nucleotidase isoform 2 preproprotein	GBC IIIA/Healthy, GSD; GBC IVB/Healthy, GSD	IIIA and IVB	4	17	**5.117**	**5.847**	8.0E-12	**4.867**	**3.307**	2.0E-08	**6.071**	**7.703**	9.0E-09	**5.762**	**4.244**	4.1E-07	40.5	115.3	28.8	107.9	45.4	118.9	36.1	103.7	57.9	6.98	325651886																													
325651886																															325651886	LEQTSEDSSK	9	N-Term(iTRAQ4plex); K10(iTRAQ4plex)	1411.71770	5.202	10.278	6.141	6.759	13.234	6.008	6.879	8.476	61.3	4.2	17.6	24.3	9.0	6.1	100.4	13.7	0.0E+00	3.5E-02	4.1	77	1.7E-06	3.4	72	5.0E-06	0
325651886																															325651886	YPFIVTSDDGR	2	N-Term(iTRAQ4plex)	1413.71343		5.847		3.307	7.703			4.244									0.0E+00	5.3E-03				2.8	55	2.5E-04	0
325651886																															325651886	VPSYDPLK	4	N-Term(iTRAQ4plex); K8(iTRAQ4plex)	1206.69853		2.705		1.952	3.114			2.189		14.9		12.7		13.9		13.7	0.0E+00	5.2E-03				2.3	41	8.6E-03	0
325651886																															325651886	LDVLCTK	2	N-Term(iTRAQ4plex); C5(Methylthio); K7(iTRAQ4plex)	1125.62554	5.034		3.858			6.135	4.827										1.0E-03	1.8E-02	2.7	42	4.6E-03				0
354459356	NP_001238851.1	RAP1B	5908	ras-related protein Rap-1b isoform 4	GBC IIIA/Healthy; GBC IVB vs Healthy	IIIA and IVB	3	12	**0.251**	**0.289**	1.5E-07	0.978	0.849	8.7E-01	**0.287**	**0.319**	2.7E-06	1.114	0.956	1.6E-02	37.1	20.0	20.1	38.5	24.6	21.1	32.5	26.8	15.3	8.13	354459356																													
354459356																															354459356	CDLEDER	5	N-Term(iTRAQ4plex); C1(Methylthio)	1069.44390	0.606	0.268	2.152	0.917	0.318	0.394	1.437	1.058		50.8		15.0		11.5		21.8	1.0E-03	5.9E-02	2.4			2.8	32	2.9E-03	0
354459356																															354459356	YDPTIEDSYR	3	N-Term(iTRAQ4plex)	1402.65593	0.238	0.289	0.884	0.743	0.319	0.244	0.927	0.801									0.0E+00	2.0E-03	3.5	67	6.8E-06	2.4			0

354459356																															354459356	LVVLGSGGVGK	4	N-Term(iTRAQ4plex); K11(iTRAQ4plex)	1273.81328	0.231		0.849			0.287	1.082		20.1		32.5		37.1		24.5		0.0E+00	5.3E-04	3.9	55	1.1E-04				0
530369972	XP_005246428.1	DPP4	1803	PREDICTED: dipeptidyl peptidase 4 isoform X1	GBC IIIA/Healthy; GBC IVB vs Healthy	IIIA and IVB	2	4	**2.179**	**2.625**	9.5E-03	1.786	**2.475**	8.2E-02	**1.981**	**2.880**	1.2E-01	1.667	**2.645**	1.7E-01									88.1	6.11	530369972																													
530369972																															530369972	VLEDNSALDK	2	N-Term(iTRAQ4plex); K10(iTRAQ4plex)	1391.76213		2.625		2.475	2.880			2.645									0.0E+00	1.5E-06				4.1	76	3.2E-06	0
530369972																															530369972	AGAVNPTVK	2	N-Term(iTRAQ4plex); K9(iTRAQ4plex)	1144.69573	2.179		1.786			1.981	1.667										0.0E+00	7.1E-04	3.7	36	1.6E-02				0
530380541	XP_005268499.1	NDST1	3340	PREDICTED: bifunctional heparan sulfate N-deacetylase/N-sulfotransferase 1 isoform X8	GBC IVB/Healthy	IVB specific	1	7	**0.521**	0.905	8.5E-03	0.886	1.075	6.8E-01	**0.309**	**0.517**	3.0E-04	0.552	0.599	2.9E-05	4.8	38.8	6.8	6.7	8.1	35.6	6.8	3.9	54.6	8.75	530380541																													
530380541																															530380541	AAALLPK	7	N-Term(iTRAQ4plex); K7(iTRAQ4plex)	971.65038	0.521	0.905	0.886	1.075	0.517	0.309	0.552	0.599	6.8	6.7	6.8	3.9	4.8	38.8	8.1	35.6	2.0E-03	2.0E-02	3.1	43	1.5E-03	3.1	44	3.5E-04	0
557786227	NP_001273579.1	ADH1B	125	alcohol dehydrogenase 1B isoform 2	GBC IIIA/Healthy	IIIA specific	1	4	**2.504**	**4.070**	1.5E-01	1.870	**4.502**	1.0E-01	1.323	1.203	9.7E-01	1.015	1.296	6.3E-01									35.4	7.87	557786227																													
557786227																															557786227	GAVYGGFK	4	N-Term(iTRAQ4plex); K8(iTRAQ4plex)	1086.61772	2.504	4.070	1.870	4.502	1.203	1.323	1.015	1.296									0.0E+00	2.4E-06	3.1	38	1.1E-02	3.6	53	3.8E-04	0
572152963	NP_001275762.1	GGT1	2678	gamma-glutamyltranspeptidase 1 precursor	GBC IIIA/Healthy, GSD; GBC IVB/Healthy, GSD	IIIA and IVB	5	21	**3.235**	**5.060**	5.3E-12	**3.172**	**5.947**	5.9E-12	**2.687**	**4.302**	2.7E-09	**2.754**	**5.018**	1.1E-07	54.8	42.9	33.4	119.8	42.9	85.9	66.3	35.0	61.4	7.12	572152963																													
572152963																															572152963	ESVESPEQK	11	N-Term(iTRAQ4plex); K9(iTRAQ4plex)	1320.69023	4.218	5.060	7.431	7.624	4.302	3.354	6.068	5.642	23.8	71.1	38.8	16.8	46.7	4.3	16.6	35.4	0.0E+00	1.5E-06	3.3	42	7.7E-03	3.2	61	9.4E-05	0
572152963																															572152963	LFQPSIQLAR	2	N-Term(iTRAQ4plex)	1316.78081		9.561		5.947	8.283			5.018									0.0E+00	3.4E-02				2.8	30	7.3E-02	0
572152963																															572152963	AAVAADAK	4	N-Term(iTRAQ4plex); K8(iTRAQ4plex)	1004.59789	1.982	2.234	2.784	3.049	1.914	1.887	2.721	2.544									0.0E+00	3.6E-03	3.3	45	3.2E-03	3.2	40	1.1E-02	0
572152963																															572152963	FVDVTEVVR	2	N-Term(iTRAQ4plex)	1207.67949	3.756		3.276			3.112	2.787										0.0E+00	1.1E-03	3.6	38	1.7E-02				0
572152963																															572152963	GLAAALENK	2	N-Term(iTRAQ4plex); K9(iTRAQ4plex)	1174.70391	2.786		1.809			1.847	1.231										0.0E+00	1.8E-04	4.1	61	5.9E-05				0
768039830	XP_011529433.1	FLNA	2316	PREDICTED: filamin-A isoform X5	GBC IIIA/Healthy; GBC IVB vs Healthy	IIIA and IVB	21	65	**0.458**	**0.469**	9.0E-16	0.862	1.079	4.6E-01	**0.508**	**0.481**	1.0E-17	1.057	1.201	2.8E-03	17.2	14.4	30.6	39.6	24.7	24.9	6.1	20.6	273.5	6.09	768039830																													
768039830																															768039830	VTGDDSMR	2	N-Term(iTRAQ4plex)	1024.48711		0.488		1.248	0.654			1.630									2.0E-03	1.0E-01				2.0	17	6.8E-01	0
768039830																															768039830	AEISFEDR	5	N-Term(iTRAQ4plex)	1110.55632	0.238	0.287	0.767	1.198	0.376	0.306	1.014	1.528		23.9		6.7		16.2		33.9	0.0E+00	2.2E-02	2.2			3.2	40	5.9E-03	0
768039830																															768039830	LTVSSLQESGLK	2	N-Term(iTRAQ4plex); K12(iTRAQ4plex)	1549.90788		0.329		1.504	0.318			1.412									0.0E+00	1.5E-06				4.3	69	1.0E-05	0
768039830																															768039830	AVPTGDASK	6	N-Term(iTRAQ4plex); K9(iTRAQ4plex)	1133.64446	0.458	0.364	0.967	1.110	0.462	0.508	1.101	1.369		28.8		23.8		3.6		1.1	0.0E+00	1.4E-05	2.6	33	5.9E-02	2.5	51	9.5E-04	0
768039830																															768039830	AGQSAAGAAPGGGVDTR	4	N-Term(iTRAQ4plex)	1586.80010		0.750		1.277	0.803			1.332		80.4		2.5		20.7		56.5	0.0E+00	1.5E-06				4.0	63	4.0E-05	0
768039830																															768039830	AEAGVPAEFSIWTR	4	N-Term(iTRAQ4plex)	1677.87749	0.252	0.219	1.009	1.359	0.216	0.265	1.089	1.304									0.0E+00	9.2E-05	3.6	50	1.4E-03	3.5	29	1.8E-01	0
768039830																															768039830	AFGPGLQGGSAGSPAR	8	N-Term(iTRAQ4plex)	1573.82610	0.375	0.488	0.949	0.869	0.720	0.465	1.208	1.265		3.3		21.5		23.9		62.1	0.0E+00	1.6E-04	5.3	87	2.0E-07	4.5	87	1.8E-07	0
768039830																															768039830	VAQPTITDNK	7	N-Term(iTRAQ4plex); K10(iTRAQ4plex)	1374.78655	0.360	0.396	0.981	1.189	0.430	0.370	1.035	1.257	73.4	7.4	57.7	6.9	52.7	5.4	67.9	8.9	0.0E+00	3.3E-02	3.3	77	2.1E-06	2.5	43	5.3E-03	0
768039830																															768039830	YGGDEIPFSPYR	4	N-Term(iTRAQ4plex)	1544.75334	0.303	0.358	1.038	0.976	0.452	0.458	1.612	1.201									0.0E+00	7.4E-03	3.5	43	3.5E-03	4.1	41	5.1E-03	0
768039830																															768039830	FNEEHIPDSPFVVPVASPSGDAR	2	N-Term(iTRAQ4plex)	2611.29636		0.367		0.983	0.430			1.121									0.0E+00	1.5E-06				5.0	38	2.1E-02	0
768039830																															768039830	VLPTHDASK	1	N-Term(iTRAQ4plex); K9(iTRAQ4plex)	1255.72812		0.594		0.923	0.737			1.115									5.0E-03	4.4E-02					12	6.8E+00	0
768039830																															768039830	IQQNTFTR	2	N-Term(iTRAQ4plex)	1151.62956		0.390		1.031	0.431			1.108									1.0E-03	7.5E-02				2.0	13	5.1E+00	0
768039830																															768039830	TPCEEILVK	2	N-Term(iTRAQ4plex); C3(Methylthio); K9(iTRAQ4plex)	1365.74687		0.472		1.055	0.473			1.029									2.0E-03	9.3E-02				2.9	23	6.9E-01	0
768039830																															768039830	YNEQHVPGSPFTAR	2	N-Term(iTRAQ4plex)	1746.87082		0.634		1.064	0.624			1.020									0.0E+00	1.5E-06				3.4	34	4.6E-02	0
768039830																															768039830	GLVEPVDVVDNADGTQTVNYVPSR	3	N-Term(iTRAQ4plex)	2688.35483	0.995	1.113	0.818	1.025	1.116	0.689	0.582	1.002									0.0E+00	5.9E-06	3.8	44	6.2E-03	2.0			0
768039830																															768039830	VNQPASFAVSLNGAK	1	N-Term(iTRAQ4plex); N12(Deamidated); K15(iTRAQ4plex)	1791.98966		0.678		1.031	0.631			0.935									2.0E-03	9.0E-02				3.4			0
768039830																															768039830	NGHVGISFVPK	2	N-Term(iTRAQ4plex); N1(Deamidated); K11(iTRAQ4plex)	1443.82587		0.580		1.035	0.531			0.923									0.0E+00	5.1E-06				3.7	22	6.9E-01	0
768039830																															768039830	GAGTGGLGLAVEGPSEAK	2	N-Term(iTRAQ4plex); K18(iTRAQ4plex)	1859.01615		1.085		1.782	0.525			0.841									0.0E+00	1.5E-06				3.4	40	1.3E-02	0
768039830																															768039830	AWGPGLEGGVVGK	2	N-Term(iTRAQ4plex); K13(iTRAQ4plex)	1514.86045	0.545		0.846			0.663	1.057										0.0E+00	1.2E-02	3.2	18	1.4E+00				0
768039830																															768039830	FVPAEMGTHTVSVK	2	N-Term(iTRAQ4plex); K14(iTRAQ4plex)	1790.97690	0.500		0.854			0.598	1.049										1.0E-03	3.0E-02	3.0	19	1.8E+00				0
768039830																															768039830	AEFTVETR	2	N-Term(iTRAQ4plex)	1096.57646	0.491		0.862			0.569	1.025										0.0E+00	7.9E-03	3.1	17	1.2E+00				0
807201183	NP_001293141.1	THBS4	7060	thrombospondin-4 isoform b	GBC IIIA/GSD; GBC IVB vs Healthy	IIIA and IVB	3	30	1.628	1.607	1.7E-13	**2.070**	**2.691**	2.4E-17	**0.514**	**0.485**	5.5E-20	0.713	0.837	5.5E-08	27.3	21.1	14.8	14.1	35.2	19.9	24.9	22.9	95.9	4.63	807201183																													

807201183																														
807201183																														
807201183																														
807201183																														
1034609212	XP_016882689.1	VASP	7408	PREDICTED: vasodilator-stimulated phosphoprotein isoform X3	GBC IVB/Healthy	IVB specific	3	12	0.592	0.921	2.9E-02	0.790	1.022	9.7E-02	**0.481**	**0.524**	4.5E-06	0.683	0.566	1.4E-05	14.8	10.1	29.1	0.3	9.4	9.5	31.1	0.3	39.5	8.94
1034609212																														
1034609212																														
1034609212																														
1034630670	XP_016861016.1	TFG	10342	PREDICTED: protein TFG isoform X2	GBC IIIA/GSD; GBC IVB vs GSD	IIIA and IVB	2	7	0.924	0.974	9.6E-01	**3.123**	**3.554**	8.7E-04	0.974	1.016	2.7E-02	**3.380**	**3.612**	6.8E-03	47.9	88.1	3.4	17.7	9.2	20.7	33.8	83.6	43.0	5.17
1034630670																														
1034630670																														
1034630893	XP_016861103.1	ALDH1L1	10840	PREDICTED: cytosolic 10-formyltetrahydrofolate dehydrogenase isoform X3	GBC IIIA/Healthy	IIIA specific	4	8	**2.101**	**2.292**	2.1E-03	0.986	1.406	1.1E-01	1.422	1.175	6.2E-01	0.689	0.701	1.5E-03	7.0	17.0	0.9	38.6	19.3	18.1	0.4	2.4	90.5	5.88
1034630893																														
1034630893																														
1034630893																														
1034630893																														
1034661451	XP_016869299.1	YWHAZ	7534	PREDICTED: 14-3-3 protein zeta/delta isoform X1	GBC IIIA/Healthy; GBC IVB vs Healthy	IIIA and IVB	3	14	**0.271**	**0.272**	6.2E-07	0.671	1.195	3.6E-01	**0.416**	**0.245**	4.0E-07	0.821	1.168	7.5E-02	23.3	1.5	26.5	52.3	5.2	28.9	44.5	20.4	27.7	4.79
1034661451																														
1034661451																														
1034661451																														
1034674387	XP_016885034.1	MSN	4478	PREDICTED: moesin isoform X3	GBC IIIA/Healthy; GBC IVB vs Healthy	IIIA and IVB	5	17	**0.489**	**0.416**	1.6E-06	1.133	1.377	6.1E-02	**0.508**	**0.413**	2.8E-07	1.207	1.224	3.3E-01	45.0	13.2	44.9	10.7	42.1	18.5	47.8	23.7	66.6	6.16
1034674387																														
1034674387																														
1034674387																														
1034674387																														
1034674387																														

807201183	GVQCTDSR	6	N-Term(iTRAQ4plex); C4(Methylthio)	1055.47490	1.730	1.485	1.940	2.691	0.543	0.586	0.675	0.958	6.0	2.3	23.0	30.6	3.5	7.2	20.3	25.5	0.0E+00	9.4E-03	3.1	34	3.9E-03	3.2	41	8.9E-04	0
807201183	NILDNCPK	7	N-Term(iTRAQ4plex); C6(Methylthio); K8(iTRAQ4plex)	1250.64592	1.733	1.942	2.864	3.683	0.502	0.685	1.163	0.927	19.2	1.6	4.9	5.5	43.9	10.6	28.4	14.5	0.0E+00	2.2E-06	3.4	55	2.9E-04	3.6	55	2.8E-04	0
807201183	YVPNSGQEDADR	5	N-Term(iTRAQ4plex); N4(Deamidated)	1495.67974	1.522	1.537	2.602	2.183	0.542	0.403	0.708	0.750		7.4		34.6		14.8		42.7	0.0E+00	1.4E-02	2.4	35	8.1E-03	2.5	42	1.7E-03	0
807201183	YVPNSGQEDADR	12	N-Term(iTRAQ4plex)	1494.69512	1.595	1.812	1.978	2.594	0.466	0.413	0.625	0.650	22.4	18.8	21.0	12.9	42.2	20.2	9.7	14.3	0.0E+00	4.8E-03	3.3	58	6.7E-05	2.8	71	3.9E-06	0
1034609212																													
1034609212	VPAQSESVR	6	N-Term(iTRAQ4plex)	1116.61589	0.721	0.921	0.790	1.022	0.524	0.481	0.542	0.566		0.3		0.3		10.1		9.5	0.0E+00	8.2E-04	2.5	30	5.8E-02	2.3	33	4.8E-02	0
1034609212	SSSSVTTSETQPCTPSSSDYSDLQR	4	N-Term(iTRAQ4plex); C13(Methylthio)	2840.23386	0.489		0.566			0.705	0.838										0.0E+00	2.0E-04	4.1	45	1.1E-03	3.4	34	1.2E-02	0
1034609212	QVWGLNFGSK	2	N-Term(iTRAQ4plex); K10(iTRAQ4plex)	1423.79546	0.592		0.872			0.452	0.683										0.0E+00	3.1E-04	3.3	57	2.2E-04				0
1034630670																													
1034630670	NQDEINK	6	N-Term(iTRAQ4plex); K7(iTRAQ4plex)	1148.61943	0.924	0.865	3.123	5.925	0.885	0.974	3.380	5.903	3.4		33.8		47.9		9.2		1.0E-03	7.9E-02	3.4	43	4.8E-03	2.9	48	1.4E-03	0
1034630670	AQLGEDIR	1	N-Term(iTRAQ4plex)	1045.57768		1.096		2.131	1.167			2.211									2.0E-03	1.0E-01				2.6			0
1034630893																													
1034630893	ALYVSDK	4	N-Term(iTRAQ4plex); K7(iTRAQ4plex)	1083.62822		2.292		1.406	1.175			0.701		38.6		2.4		17.0		18.1	0.0E+00	3.4E-03				2.5	38	1.1E-02	0
1034630893	FLFPEGIK	2	N-Term(iTRAQ4plex); K8(iTRAQ4plex)	1238.73955	18.669		2.413			5.206	0.691										0.0E+00	1.6E-02	2.7	47	1.9E-03				0
1034630893	LSDHPDVR	1	N-Term(iTRAQ4plex)	1082.57215	2.089		0.986			1.422	0.689										3.0E-03	5.5E-02	1.6						0
1034630893	GQALPDVVAK	1	N-Term(iTRAQ4plex); K10(iTRAQ4plex)	1285.77005	2.101		0.940			1.250	0.574										4.0E-03	5.4E-02		14	2.0E+00				0
1034661451																													
1034661451	NELVQK	2	N-Term(iTRAQ4plex); K6(iTRAQ4plex)	1018.61992		0.362		1.208	0.381			1.237		44.8		8.5		3.1		38.8	5.0E-03	3.5E-02					39	1.7E-02	0
1034661451	SVTEQGAELSNEER	9	N-Term(iTRAQ4plex)	1692.81938	0.255	0.195	0.513	1.195	0.236	0.299	0.616	1.019	30.1	24.7	2.5	30.4	24.3	0.4	60.6	5.6	0.0E+00	4.3E-09	5.2	42	4.3E-03	5.6	82	4.5E-07	0
1034661451	GIVDQSQQAYQEAFEISK	3	N-Term(iTRAQ4plex); K18(iTRAQ4plex)	2329.19309	0.280		0.784			0.416	1.193		26.5		15.6		7.2		3.4		0.0E+00	7.9E-07	4.4	31	1.1E-01				0
1034674387																													
1034674387	AQMVQEDLEK	2	N-Term(iTRAQ4plex); K10(iTRAQ4plex)	1478.78118		0.369		1.505	0.413			1.640									0.0E+00	1.5E-06				3.8	57	2.1E-04	0
1034674387	EALLQASR	4	N-Term(iTRAQ4plex)	1031.59953	0.653	0.418	0.848	1.385	0.444	0.667	0.889	1.433									0.0E+00	6.1E-03	2.8	22	3.7E-01	2.9	31	7.2E-02	0
1034674387	AQQELEEQTR	6	N-Term(iTRAQ4plex)	1375.70073	0.366	0.392	1.513	1.412	0.357	0.387	1.640	1.252		9.0		6.2		3.7		1.0	0.0E+00	1.5E-06	4.5	59	9.2E-05	4.3	63	4.1E-05	0
1034674387	QIEEQTK	1	N-Term(iTRAQ4plex); K7(iTRAQ4plex)	1163.65849		0.409		1.252	0.411			1.224									5.0E-03	1.6E-01				2.5			0
1034674387	ALELEQER	4	N-Term(iTRAQ4plex)	1131.61064		0.462		0.999	0.483			1.019		4.8		2.7		2.3		5.1	0.0E+00	2.8E-02				3.4	29	1.0E-01	0

Supplementary Table S6

Localization of 86 non-redundant plasma-derived EV proteins with altered levels in GBC using STRING database

Cellular component			
**GO-term**	**Description**	**Count in gene set**	**False discovery rate**
GO:0005576	Extracellular region	51 of 2505	1.06E-21
GO:0031982	Vesicle	46 of 2318	1.32E-18
GO:0030141	Secretory granule	30 of 828	1.04E-17
GO:0060205	Cytoplasmic vesicle lumen	22 of 340	1.52E-17
GO:0031410	Cytoplasmic vesicles	43 of 2226	5.13E-17

Supplementary Table S7

The concentration of NT5E, ANPEP and MME in GBC cases and controls as determined by quantitative ELISA. This analysis is performed using the clinical samples used for the discovery experiment (proteomic analysis) and an independent cohort **(see Supplementary Table S2).**

Gene Symbol	Healthy	GSD	XGC	Early stage	Advanced stage	
				(I and II)	III	IV
	ng/ml	ng/ml	ng/ml	ng/ml	ng/ml	ng/ml
	(Mean value ± SEM)			(Mean value ± SEM)		
NT5E	1.820 ± 0.8900	2.261 ± 1.040	0.4757 ± 0.2095	4.204 ± 1.608	9.968 ± 2.734	14.06 ± 2.250
ANPEP	555.4 ± 90.08	934.7 ± 281.8	1078 ± 426.0	2524 ± 876.0	2566 ± 696.1	4096 ± 631.6
MME	0.1331 ± 0.06060	0.1120 ± 0.04547	0.1192 ± 0.03901	0.6658 ± 0.1821	ND	ND

ND- Not determined

SEM- Standard Error of the mean

Supplementary Table S8:

Statistical analysis of quantitative ELISA data showing sensitivity and specificity of NT5E, ANPEP and MME for detection of GBC. (A) The statistical analysis performed using samples from the discovery study (proteomics study) as available and (B) an independent cohort (C) Combined cohort (discovery + independent cohort). The details of the samples are shown in **Supplementary Table S2.**

(A) Verification using Discovery Samples

	Cut off (ng/ml)	% Sensitivity	% Specificity	AUC	P value
**NT5E**					
Advanced stage GBC vs Controls	> 6.190	42.11	100	0.7197	0.01901
	> 4.136	42.11	95		
**ANPEP**					
Advanced stage GBC vs Controls	> 3413	26.32	100	0.7013	0.03165
	> 1582	47.37	95		
**MME**					
Early stage GBC vs Controls	> 0.8412	50	100	0.5625	0.7055
	> 0.3707	50	93.75		

(B) Verification using an independent cohort of samples

	Cut off (ng/ml)	% Sensitivity	% Specificity	AUC	P value
**NT5E**					
Early stage GBC vs Controls	> 18.02	7.69	96	0.6185	0.2362
	> 5.786	30.77	92		
Advanced stage GBC vs Controls	> 18.54	34.78	100	0.9252	<0.0001
	> 18.04	34.78	96		
GBC (Early and Advanced) vs Controls	> 18.54	22.22	100	0.8144	<0.0001
	> 18.02	25	96		
**ANPEP**					
Early stage GBC vs Controls	> 4423	23.08	100	0.5615	0.5383
	> 2021	46.15	92		
Advanced stage GBC vs Controls	> 4185	56.52	100	0.8678	<0.0001
	> 3786	60.87	96		
GBC (Early and Advanced) vs Controls	> 4185	44.44	100	0.7572	0.0006
	> 3786	47.22	96		
**MME**					
Early stage GBC vs Controls	> 0.4434	66.67	100	0.7333	0.08647
	> 0.3059	66.67	90		

(C) Verification using the combined cohort (Discovery + Independent cohort)

	Cut off (ng/ml)	% Sensitivity	% Specificity	AUC	P value
**NT5E**					
Early stage GBC vs Controls	> 18.02	7.69	97.78	0.5829	0.3659
	> 6.008	30.77	95.56		
Advanced stage GBC vs Controls	> 18.54	28.57	100	0.8354	0.0001
	> 18.04	28.57	97.78		
GBC (Early and Advanced) vs Controls	> 18.54	21.82	100	0.7758	<0.0001
	> 18.02	23.64	97.78		
**ANPEP**					
Early stage GBC vs Controls	>4423	23.08	100	0.5863	0.3464
	>3965	46.15	97.78		
Advanced stage GBC vs Controls	> 4185	40.48	100	0.7873	<0.0001
	> 3786	42.86	97.78		
GBC (Early and Advanced) vs Controls	>4185	36.36	100	0.7398	<0.0001
	>3786	38.18	97.78		
**MME**					
Early stage GBC vs Controls	> 0.5337	61.54	100	0.6827	0.06983
	> 0.4010	61.54	95.83		

AUC > 0.7 is considered significant

The p values <0.05 was considered significant.
